# Nickel Meets Aryl Thianthrenium Salts: Ni(I)-Catalyzed Halogenation of Arenes

**DOI:** 10.1021/jacs.3c02611

**Published:** 2023-05-01

**Authors:** Shengyang Ni, Jiyao Yan, Srija Tewari, Edward J. Reijerse, Tobias Ritter, Josep Cornella

**Affiliations:** † Max-Planck-Institut für Kohlenforschung, Kaiser-Wilhelm-Platz 1, Mülheim an der Ruhr 45470, Germany; § Max Planck Institut for Chemical Energy Conversion, Stiftstrasse 34-36, Mülheim an der Ruhr 45470, Germany

## Abstract

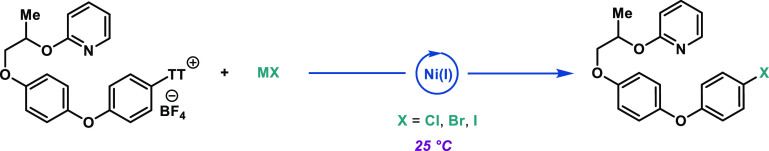

Herein, a regioselective, late-stage two-step arene halogenation method is reported. We propose how unusual Ni(I)/(III) catalysis is enabled by a combination of aryl thianthrenium and Ni redox properties that is hitherto unachieved with other (pseudo)halides. The catalyst is accessed in situ from inexpensive NiCl_2_·6(H_2_O) and zinc without the need of supporting ligands.

Direct C–H thianthrenation has emerged as a synthetic tool for functionalization of aromatic rings.^1,2^ It proceeds under mild conditions and tolerates a wide variety of functional groups and thereby addresses one of the most challenging aspects of C–H functionalization: control of the regio- and chemoselectivity.^3^ Aryl thianthrenium salts have been shown to be excellent one-electron electrophiles in various photochemical and SET (single electron transfer)-type transformations reported to date.^1,2^ However, despite the wealth of examples, processes that involve SET reactions of thianthrenium salts using nickel as catalyst are still elusive.^4^ Here, we report a Ni(I)-catalyzed cross-coupling of arylthianthrenium salts to afford aryl halides (Cl, Br, and I). Because thianthrenation proceeds regio- and chemoselectively at a late stage, halides that would not readily be accessible by late-stage halogenation can be accessed with potential applications in selective radiolabeling, access of pharmaceutical candidates, or organometallic chemistry through halogen/metal exchange.^5^ The proposed redox cycle fundamentally differs from previously reported halogenation reactions^6−8^ and may be a consequence of the productive interplay of thianthrenium and nickel featuring distinct redox properties, which enables a smooth and practical reaction at room temperature with a broad substrate scope. The conceptual advance to realize an unusual but straightforward redox cycle is promising for the development of additional thianthrenium chemistry with nickel. Different to the commonly proposed mechansims,^9,10^ Nocera et al.^11^ has recently shown that a bipyridine-Ni catalyst system can engage in C–N and C–O bond forming reactions *under thermal conditions*, invoking a self-sustained Ni(I)/Ni(III) process, thus avoiding the combination with photo-^9^ or electrocatalysis.^10^ However, the process is limited to electron-deficient aryl bromides, due to the general reluctance to C–X cleavage of electron-rich substrates using Ni(I) complexes with diamines as ligands.^12^ Due to the low bond dissociation energy of the C(sp^2^)–S bond in aryl thianthrenium salts upon reduction, they are excellent candidates to engage in one-electron processes regardless of their substitution pattern.^1,2^ While halogenation of arylthianthrenium salts has been reported, stoichiometric copper salts and irradiation are required.^1a,1e^ Conventional arene halogenation relies on the use of electrophilic halogenating reagents, such as X_2_ or element–X-type reagents such as N-bromosuccinimide (NBS) (Figure 1), which commonly results in low regioselectivity, particularly for unbiased substrates.^13^ Although recent years have witnessed an increasing number of electrophilic halogenating reagents, direct late stage C(sp^2^)–X bond formation still remains a synthetic challenge.^14^ Interexchanges of X groups in aromatic Finkelstein-type reactions have been reported,^15,16^ but they all require the synthesis of the parent aryl halide as starting material. In order to provide a broad and robust method, herein, we report on a thermal Ni(I)-catalyzed halogenation of arylthianthrenium salts with broadly available, nucleophilic halide sources. The catalytic system is simple, featuring one of the least expensive sources of Ni, NiCl_2_·(H_2_O)_6_, and zinc metal for in situ reduction. The protocol is robust, scalable, and broad in scope, as exemplified by the late stage halogenation of densely functionalized molecules, without the need for photocatalysis or electrochemistry.

**Figure 1:**
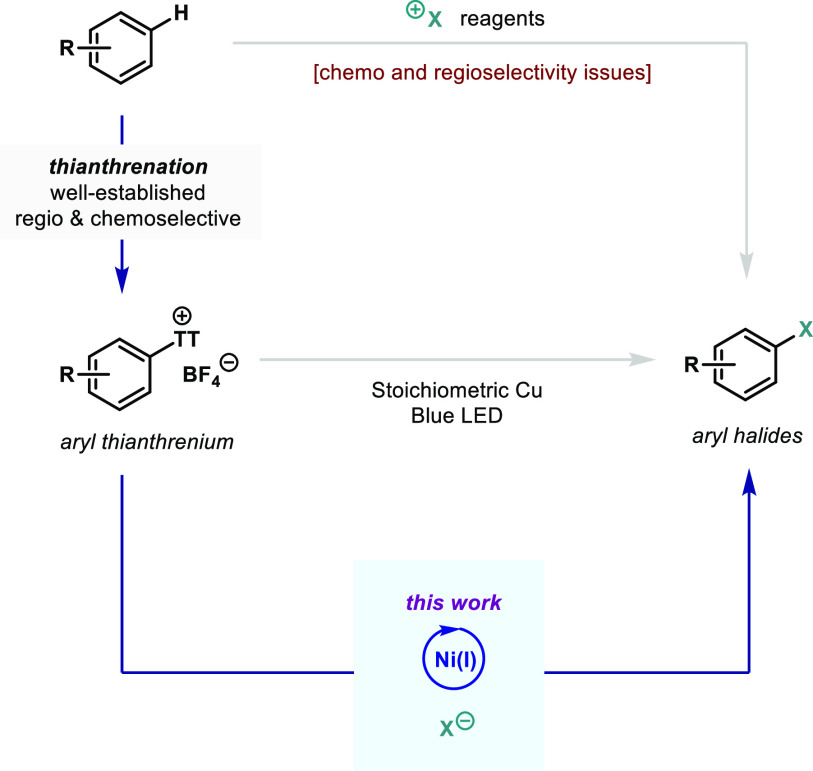
Halogenation of arenes: state-of-the-art.

Catalytic nickel chloride hexahydrate combined with zinc in the presence of sodium iodide suffices to convert thianthrenium salt **1** into the corresponding iodinated product **2** at 25 °C (Scheme 1A, entry 1). Notice that this protocol uses common NaI as halogen source and does not require the presence of additional supporting ligands for the metal center.^17^ During the optimization, it was found that dimethylacetamide (DMA) was a crucial solvent for the reaction to proceed; DMF inhibited the reactivity (entry 2). All the elements in the catalytic system are necessary for the reaction to proceed; omission of either Ni or Zn did not lead to any detectable formation of **2** (entries 3 and 4). In addition to simple Ni(II) salt as catalyst, Ni(1,4-cyclooctadiene (COD))_2_ or Ni(^tBu^stb)_3_ can be used as well (entry 5), and both Ni(COD)_2_ and NiCl_2_/Zn afforded excellent yields of **3** on 5 mmol scale (entries 1 and 5). When using undistilled “wet” DMA, the reaction can still occur, albeit the yield halts at 73% (entry 6). However, the inert atmosphere was crucial for the reaction, and only a trace amount of **2** was detected if the reaction is run open-air (entry 7). Based on precedents in ligandless Ni catalysis,^18^ the need for a reducing agent when using a simple Ni(II) salt points to the possible generation of Ni(I) as the active species. Along these lines, when using Wilke-Morandi’s well-defined Ni(I) complex Ni(COD)(OAr*) without Zn,^19^**2** was also obtained in high yields (entry 8). Catalytic amounts of Ni(0) precursors are able to catalyze the reaction, presumably via the in situ formation of Ni(I) species through initial oxidation by the arylthianthrenium salt (entry 6 and vide infra). Larger amounts of Ni(0) led to a linear decrease of yield, ultimately reaching only trace amounts of **2** with 1.0 equiv of Ni(COD)_2_ (Scheme 1B, top). However, when the reaction with 100 mol % Ni(COD)_2_ is diluted to 0.02 M in DMA, a 41% yield of **2** is obtained, thus suggesting that aggregations of Ni(0) deactivate the Ni catalyst at high concentrations (see the Supporting Information). When one-electron oxidant FeCp_2_BAr^F^ was added to the reaction with 1.0 equiv of Ni(COD)_2_ prior to the addition of the thianthrenium salt, reactivity was restored and product **2** was obtained in 75% yield, supporting that one full equivalent of Ni(I) is able to sustain the reactivity (Scheme 1B, bottom). Electron paramagnetic resonance (EPR) analysis of an equimolar mixture of Ni(COD)_2_ and FeCp_2_BAr^F^ in toluene:THF revealed the formation of a Ni(I) species consistent with cationic [Ni(COD)_2_]^+^ (Scheme 1C).^20^ Addition of **1** and NaI to this mixture led to a rapid color change from light yellow to red, and 75% of **2** was obtained. Moreover, in situ EPR analysis during the reaction at different reaction times clearly indicates the presence of one paramagnetic Ni species distinct from the cationic Ni(I) made from Ni(COD)_2_ and FeCp_2_BAr^F^. This species appears at high concentration and slowly fades away as the reaction progresses. The signal is analogous to the one obtained by Reisman while monitoring the Ni-catalyzed triflate-iodide exchange reaction of enol ethers (see the Supporting Information for details).^21^ Although insights on the exact structure of the active Ni(I) catalyst are still elusive given that it evaded isolation, the in situ EPR experiments further support the Ni(I) hypothesis (see the Supporting Information for details). Based on the above experiments, we speculate that the reaction mechanism proceeds through the initial formation of the L–Ni(I)–X species **A** (Scheme 1D), where L is COD or DMA, with appropriate redox potential for oxidative addition to the aryl-TT salt. After initial SET from Ni(I) to the cationic thianthrenium salt, mesolytic cleavage of the C–S bond would lead to the formation of an Ar radical, which prior to escaping the solvent cage in **B**, undergoes oxidative ligation to the Ni(II) species, to afford Ni(III) intermediate **C**. Reductive elimination from **C** leads to the formation of the C–halogen bond and presumably **D**, which after rapid anion metathesis regenerates the active species **A**. It should be pointed out that the mechanism in Scheme 1, although plausible, remains purely speculative at present. The ability to undergo oxidative addition to a “naked” Ni(I) compound, even for electron-rich arenes, distinguishes the arylthianthrenium salts from aryl-halides and other pseudohalides and underscores the utility of arylthianthreniums beyond conventional cross-coupling precursors.^11,12^

**Scheme 1:**
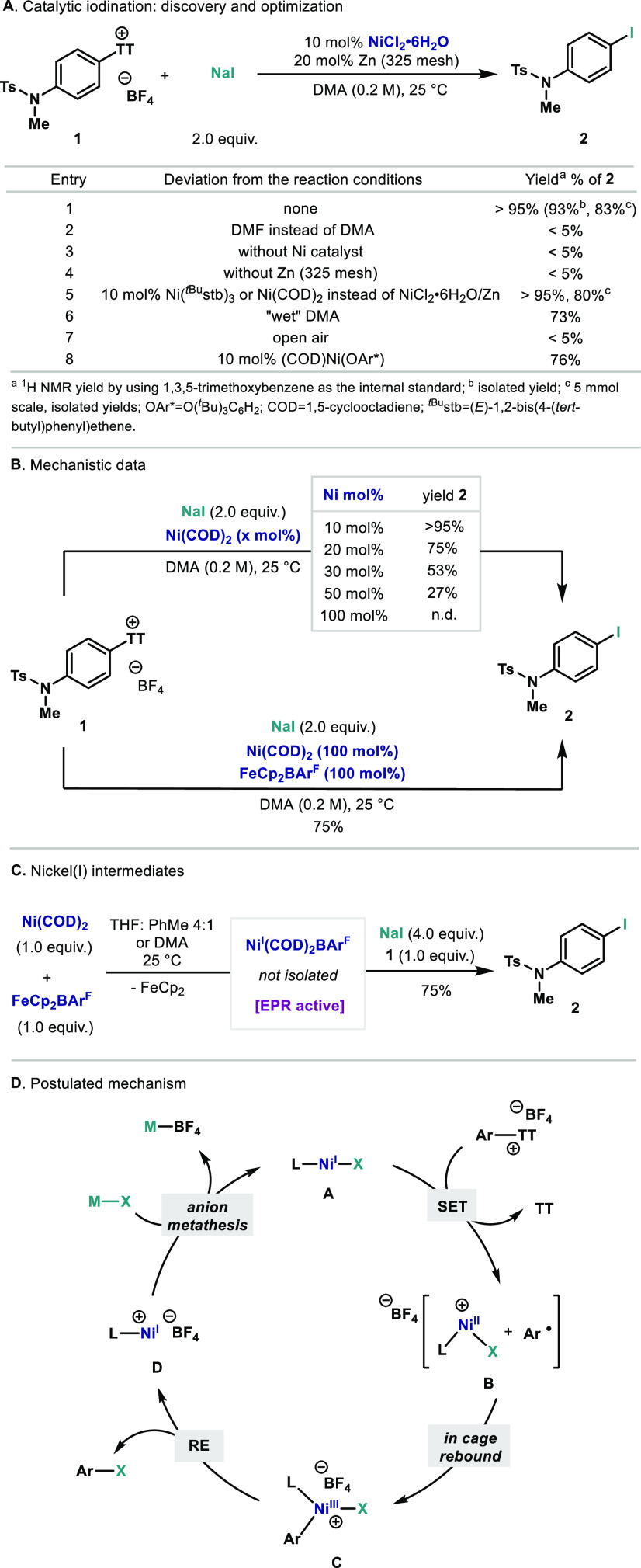
(A) Discovery and Optimization; (B) Influence of the Catalyst Loading and Oxidants; (C) Evidence for the Formation of Ni(I) Intermediates; (D) Proposed Mechanism of the Ni-Catalyzed Halogenation of Arylthianthrenium Salts

Sodium iodide could successfully be swapped for NaBr or tetrabutylammonium chloride leading to the corresponding aryl bromide (**3**) and chloride (**4**), respectively, in excellent yields (Scheme 2). Attempts to extend the scope to fluorination in this manner were thus far unsuccessful. Other MX salts have also been tested employing the optimized conditions to probe the extent of additional C–X coupling reactions: salts such as tetrabutylammonium difluorotriphenylsilicate (TBAT), tris(dimethylamino)sulfonium difluorotrimethylsilicate (TASF), KF, and NaF (for C–F) or KOAc and PhCO_2_Na (for C–O bonds) did not afford the corresponding products, and the starting material remained unreacted. It is important to note that *one single, simple catalytic system* allows for the synthesis of a triad of halogenated arenes by simply selecting the desired halide salt.

**Scheme 2:**
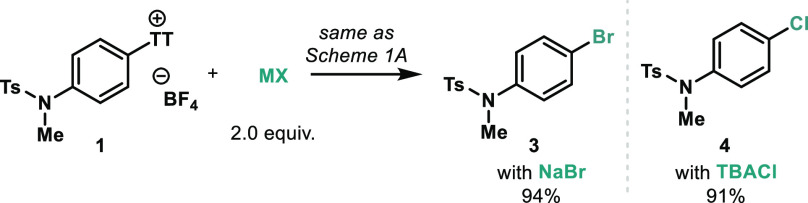
Expansion of the Protocol to the Corresponding Bromination and Chlorination

As shown in Scheme 3, both simple (**2**–**37**) and complex functionalized small molecules (**38**–**50**) can successfully be halogenated. A main feature of this protocol is the mild conditions, which permit tolerance of a wide range of functionalities, namely, sulfonamides, amides, imides, ethers, esters, halogens, nitriles, carbamates, hydroxyl, trifluoromethyl, ketones, biaryl, aldehydes, alkyl- and arylhalides, unsaturated heterocycles, amines, or encumbered olefin among others. It is also important to mention that halogenation occurs smoothly regardless of the substitution pattern: *ortho*- (**37**, **39**, **40**, **49**–**50**), *meta*- (**31**, **47**), and *para*-substituted arenes (**2**–**25**, **29**, **30**, **32**, **34**–**36**, **41**–**43**) are all smoothly converted into the corresponding aryl halides. Arenes with free amines react with the thianthrene-*S*-oxide and lead to the iminothianthrenes, which have been used in allylic amination.^22^ It is worth pointing out that, due to the electrophilic nature of the thianthrenation step, substrates bearing electron-releasing groups are more suitable for this process. As thianthrenation is not only restricted to aromatic C–H bonds, we have attempted the halogenation of alkenyl thianthrenium salts derived from simple olefins.^23^ Although terminal alkenylthianthrenium salts remained unreactive (see the Supporting Information), internal alkenylthianthrenium salts were smoothly converted into iodinated (**51**, **53**–**55**) or brominated (**52**, **56**) products successfully. It is worth pointing out that the stereochemistry of the starting alkenylthianthrenium salts were preserved during the course of the reaction (**51**, **52**, and **54**). Chlorination falls beyond the scope of the protocol, leaving the starting material unreacted. In order to benchmark our protocol with other C–H halogenation strategies, the synthesis of compounds **36** and **46** was attempted with state-of-the-art electrophilic iodination methods and resulted in either regioselectivity issues or low yields (see the Supporting Information). It is noteworthy that this Ni-catalyzed platform levels the functional group tolerance of the thianthrenation step, thus accommodating a broad range of functional groups.

**Scheme 3:**
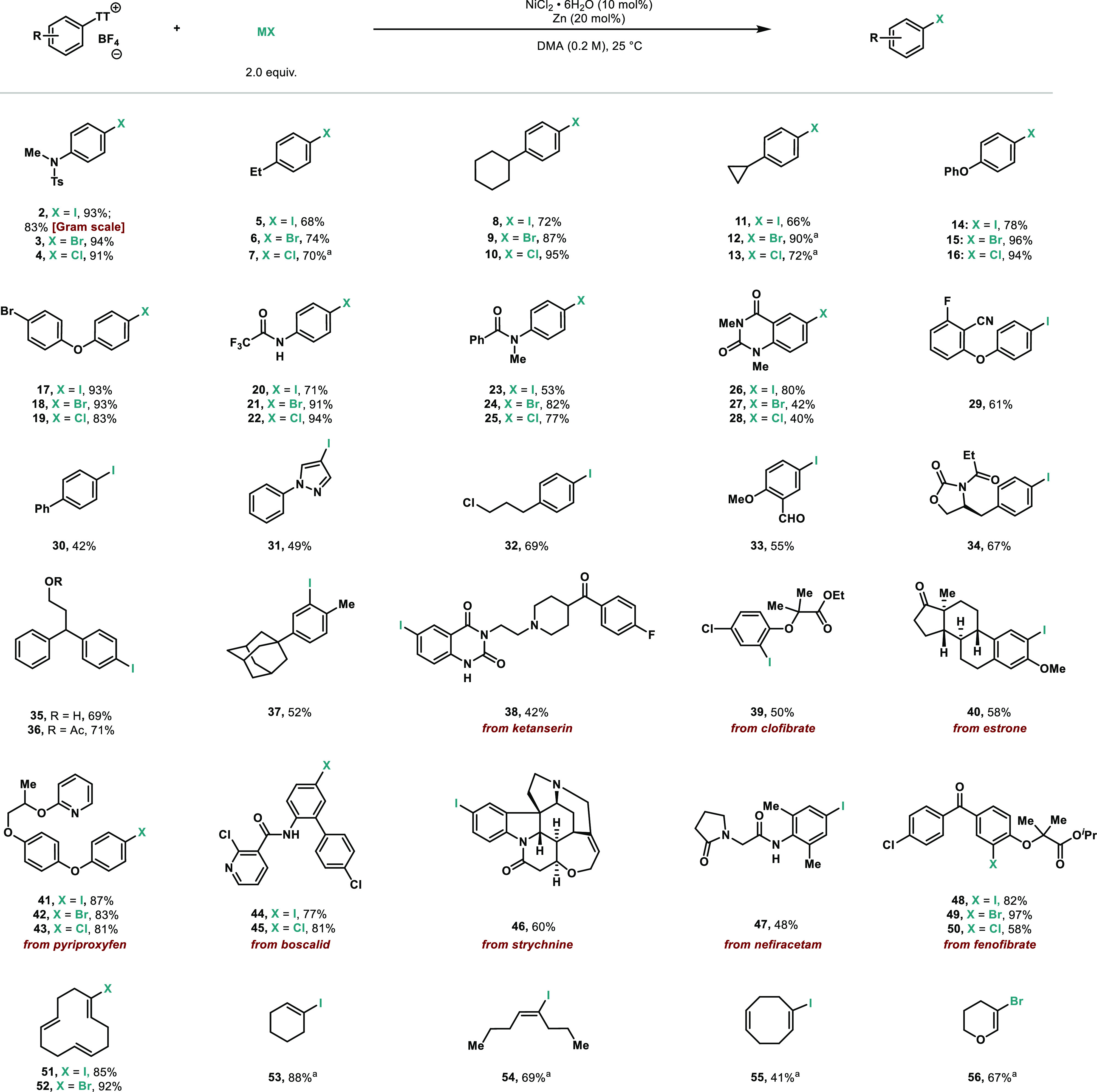
Scope of Halogenation of Aryl Thianthrenium Salts Yields of isolated products are indicated in each case. Standard reaction conditions: thianthrenium salt (1.0 equiv), NiCl_2_·6H_2_O (10 mol %), Zn (325 mesh) (20 mol %), halogenation source (NaI, NaBr, or TBACl) (2.0 equiv), DMA (0.2 M), 25 °C, 1–16 h. ^a1^H NMR yield by using 1,3,5-trimethoxybenzene as the internal standard.

In order to highlight the valuable formation of aryl halides, we provide two examples where the formation of the aryl halide can be telescoped in a two-step procedure: a reductive coupling with N_2_O to produce phenols (**58**, 51% yield) (Scheme 4A)^24^ and C–C bond formation with redox-active esters (**60**, 58% yield) (Scheme 4B).^25^

**Scheme 4:**
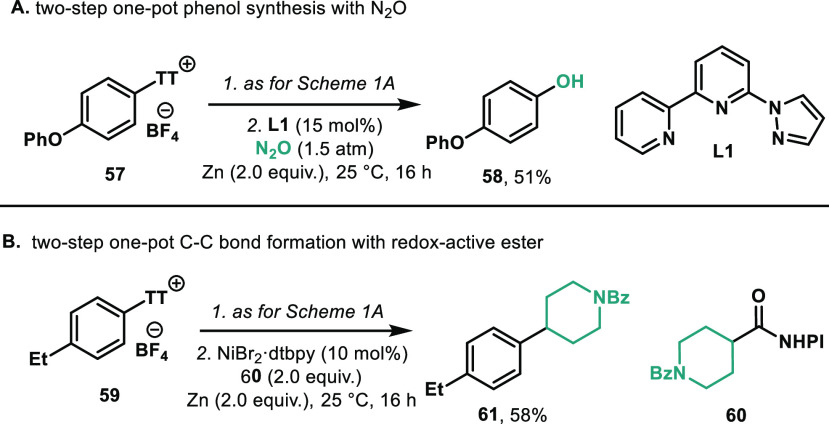
Two-Step One-Pot Transformation

The halogenation protocol presented here highlights that thermal Ni redox catalysis without the need of supporting ligands is within reach with suitably activated electrophilic aryl counterparts. Our laboratories are currently exploring these possibilities further.

## Supporting Information Available

Experimental procedures and analytical data (^1^H, ^13^C, and ^19^F NMR, HRMS) for new compounds (PDF)
