# Supplementary material for: Nickel Meets Aryl Thianthrenium Salts: Ni(I)-Catalyzed Halogenation of Arenes
Source: J Am Chem Soc. 2023 May 1;145(18):9988–93. doi: 10.1021/jacs.3c02611 (PMC10176483; doi:10.1021/jacs.3c02611)

Supporting Information

Nickel meets Aryl Thianthrenium Salts: Ni(I)-catalyzed Halogenation of Arenes

Shengyang Ni,[1] Jiyao Yan,[1] Srija Tewari,[1] Edward J. Reijerse,[2] Tobias Ritter,*[1] Josep Cornella*[1]

[1] Max-Planck-Institut für Kohlenforschung, Kaiser-Wilhelm-Platz 1, Mülheim an der Ruhr, 45470, Germany

[2] Max Planck Institute for Chemical Energy Conversion, Stiftstrasse 34-36, Mülheim an der Ruhr, 45470, Germany

Table of Contents

1 General Methods ... 2
2 Optimization of the reaction parameters ... 3
2.1 Nickel catalysts ... 3
2.2 Zinc reducing agent ... 3
2.3 Other reductants ... 4
2.4 Solvents ... 4
2.5 Reaction setup with “zero-precaution” ... 4
2.6 Cl sources ... 5
2.7 Attempts to halogenate terminal alkenylthianthrenium salt ... 5
2.8 Increasing the catalyst loading ... 6
3 UV-vis analysis ... 8
4 EPR analysis ... 10
5 Competition and radical trap experiments ... 13
6 Results of fluorination and other MX salts ... 14
7 Iodination of Arenes using reported methods ... 16
8 Preliminary kinetic studies ... 17
9 List of 2-step procedure table ... 19
10 Procedure for catalytic halogenation of aryl thianthrenium salts (with Ni^{II}+*Zn*) (Procedure A) ... 20
11 Procedure for catalytic halogenation of aryl thianthrenium salts (with Ni^{0}) (Procedure B) ... 22
12 Characterization Data ... 23
13 References ... 56
14 NMR Spectra ... 59

1 General Methods

Instruments

GC-MS (FID): GC-MS-QP2010 equipped (Shimadzu Europe Analytical Instruments). ESI-MS: ESQ 3000 (Bruker). Accurate mass determinations: Bruker APEX III FT-MS (7 T magnet) or MAT 95 (Finnigan). Melting points were measured with an EZ-Melt Automated Melting Point Apparatus from Standford Research Systems. NMR spectra were recorded using a Bruker AVIIIHD 300 MHz, Bruker AVneo 500 MHz or Bruker AVneo 600 MHz NMR spectrometer. The chemical shifts (δ) are given in ppm and were measured relative to solvent residual peak as an internal standard. For ^{1}H NMR: $CDCl_3$, δ 7.26; $(CD_3)_2(SO)$, δ 2.50. For ^{13}C NMR: $CDCl_3$, δ 77.16; $(CD_3)_2(SO)$, δ 39.52. The data is being reported as (s = singlet, d = doublet, t = triplet, q = quartet, quint = quintet, m = multiplet or unresolved br s = broad signal, coupling constant(s) in Hz, integration, interpretation). All UV-Vis measurements were recorded on a Shimadzu UV-Vis Spectrophotometer UV-2600 with a temperature controller using a screw-top quartz cuvette (Hellma Fluorescence quartz cuvette, 10 × 4 mm, 1.4 mL). Absorbance was monitored between 300 and 700 nm with a path length of 1 cm using a solution of DMA as a background solution. EPR was recorded on a Bruker Elexsys E500 CW EPR spectrometer equipped with an ER4116DM resonator and an Oxford ESR900 cryostat at 15 K.

Chemicals

Unless otherwise stated, all manipulations were performed using Schlenk techniques under dry argon in heatgun-dried glassware. Unless otherwise noted, all reagents were obtained from commercial suppliers and used without further purification. Anhydrous DMA (250 mL, 99.8%), NaI (anhydrous, free-flowing, Redi-Dri™, ReagentPlus®, ≥99%), NaBr (ACS reagent, ≥99.0%) and Tetrabutylammonium chloride (≥97.0% (NT)) were purchased from Sigma-Aldrich, stored directly in the glovebox, and use as received. Zinc powder (325 mesh, 99.9% (metal basis)) was purchased from Alfa Aesar, stored directly in the glovebox, and used as received without further activation. Aryl thianthrenium salts **1** were prepared according to procedures reported in the literature[1].

2 Optimization of the reaction parameters

2.1 Nickel catalysts

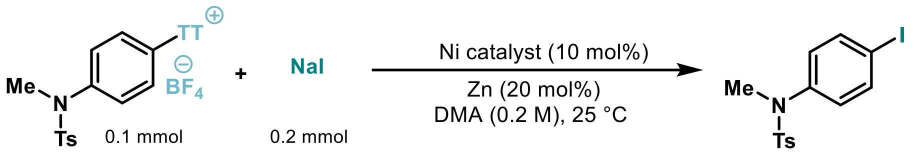

Nickel catalyst	yield[a] %
NiI_2	> 95%
$NiBr_2$•glyme	> 95%
$NiBr_2$•diglyme	> 95%
$NiCl_2$•6H_2O	> 95%
Ni(acac)$_2$	trace

[a] Yields were determined by crude ^{1}H NMR using 1,3,5-trimethoxybenzene as internal standard.

2.2 Zinc reducing agent

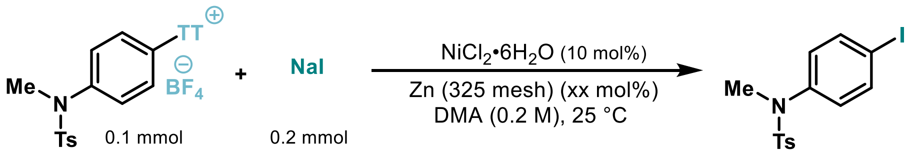

Zn	yield[a] %
10 mol%	> 95%
20 mol%	> 95%
40 mol%	48%
50 mol%	29%
100 mol%	trace
200 mol%	trace

[a] Yields were determined by crude ^{1}H NMR using 1,3,5-trimethoxybenzene as internal standard.

2.3 Other reductants

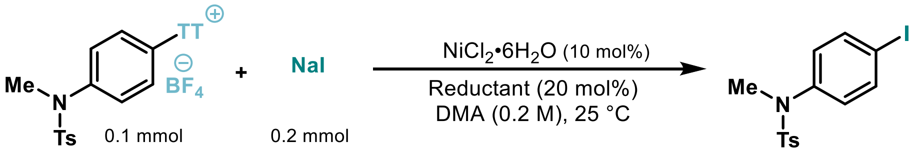

Reductant	yield[a] %
Zn 325 mesh	> 95%
Zn 140 - 325 mesh	78%
Mn	85%

[a] Yields were determined by crude ^{1}H NMR using 1,3,5-trimethoxybenzene as internal standard.

2.4 Solvents

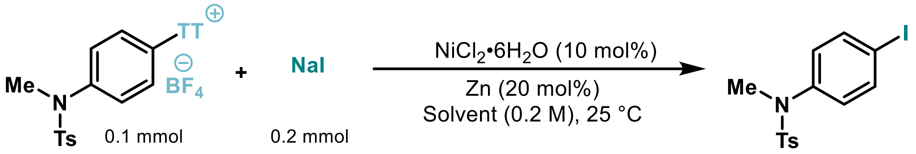

Solvent	yield[a] %
DMA	> 95%
DMF	trace
DMSO	trace
THF	62%
MeCN	45%

[a] Yields were determined by crude ^{1}H NMR using 1,3,5-trimethoxybenzene as internal standard.

2.5 Reaction setup with “zero-precaution”

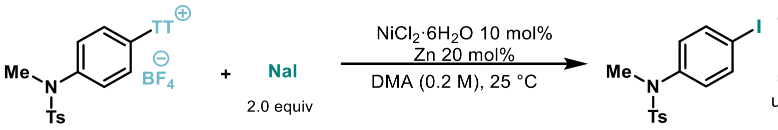

reaction conditions	^{1}H NMR yield%
under air	trace, SM remained
using the "wet" DMA for NaI	73%
using the "wet" DMA for NaBr	78%
using the "wet" DMA for TBACl	72%
bench NaI	> 95%

Yields were determined by crude ^{1}H NMR using 1,3,5-trimethoxybenzene as internal standard.

2.6 Cl sources

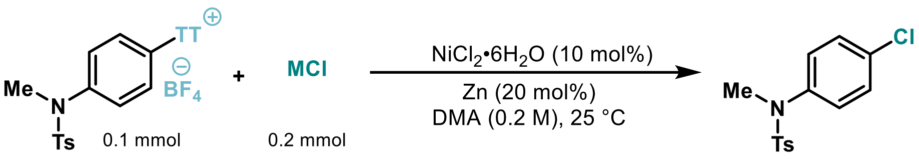

MCl	yield[a] %
NaCl	trace
TBACl	> 95%
$MgCl_2$	45%
LiCl	trace

[a] Yields were determined by crude ^1H NMR using 1,3,5-trimethoxybenzene as internal standard.

2.7 Attempts to halogenate terminal alkenylthianthrenium salt

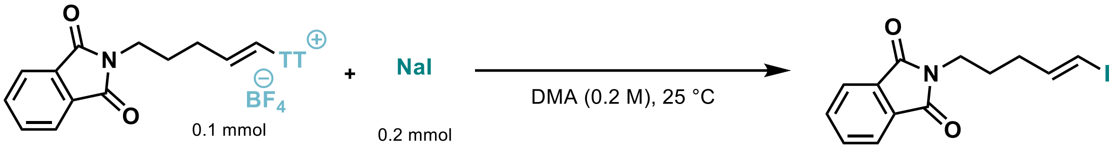

Condition	yield[a] %
$NiCl_2$•$6H_2O$ (10 mol%), Zn (20 mol%)	ND, SM remained
$Ni(COD)_2$	ND, SM remained

2.8 Increasing the catalyst loading

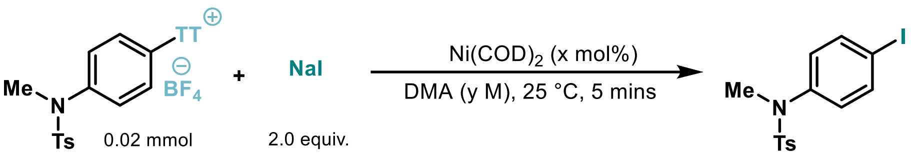

$Ni(COD)_2$ (x mol%)	DMA (y M)	yield[a] %
10 mol%	0.2 M	> 95 %
20 mol%	0.2 M	75 %
30 mol%	0.2 M	53 %
50 mol%	0.2 M	27 %
100 mol%	0.2 M	n.d.
100 mol%	0.02 M	41%

[a] Yields were determined by crude ^1H NMR using 1,3,5-trimethoxybenzene as internal standard.

Note: When the reaction with 100 mol% of $Ni(COD)_2$ is carried out under more diluted conditions, (x 10 times, 0.02 M in DMA) slight recovery of the reactivity is observed with 41% of the iodinated product obtained. The remaining of the material is protodesulfonation of the TT salt. At present, we speculate that when concentrations of Ni(0) are high, fast aggregation and precipitation of catalytically inactive species ensue. Indeed, dark-grey precipitate is observed at high concentrations of Ni(0) thus pointing to aggregation phenomena.

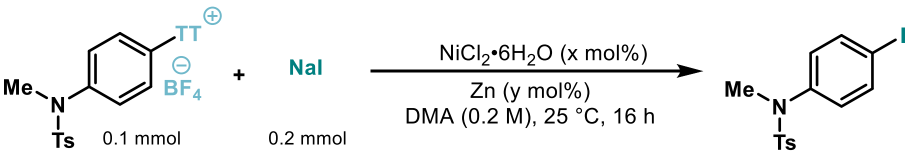

$NiCl_2 \cdot 6H_2O$ (x mol%)	Zn (y mol%)	yield[a] %
30 mol%	20 mol%	67 %
50 mol%	20 mol%	52 %
100 mol%	20 mol%	46 %
30 mol%	60 mol%	27 %
50 mol%	100 mol%	< 5%
100 mol%	200 mol%	< 5%

[a] Yields were determined by crude ^1H NMR using 1,3,5-trimethoxybenzene as internal standard.

Note: Similarly to $Ni(COD)_2$, increasing the $NiCl_2 \cdot 6H_2O$ concentration led to a gradual decrease in yield. When the ratio of Ni and Zn is kept constant, a substantial decrease in yield is observed

but this time, protodethianthrenation is observed as the major byproduct. This points at a negative effect of Zn with the TT salt.

3 UV-vis analysis

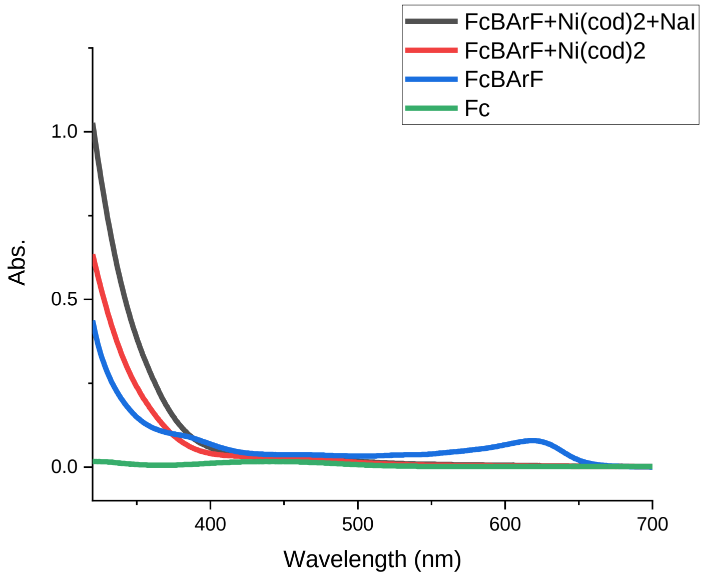

In a glovebox, a 20 mL vial equipped with a magnetic stir bar was charged with ferrocenium tetrakis[3,5-bis(trifluoromethyl)phenyl]borate ($FcBAr^F$, 21.0 mg, 20.0 µmol) and DMA (5.0 mL, 4.0 mM) (solution A). Then, 30 µL of solution A were transferred to the cuvette and diluted with 970 µL of DMA. The cuvette was sealed and taken out of the glovebox, placed in the spectrometer and the absorption spectrum of $FcBAr^F$ (0.12 mM) was acquired **(blue line)**.

(blue line) The solution has significant absorption from 320 to 400 nm and from 500 to 650 nm.

In a glovebox, $Ni(COD)_2$ (5.5 mg, 0.020 mmol, 4.0 mM) was added to solution A. The resulting suspension was stirred until $Ni(COD)_2$ was completely dissolved to form solution B. Then, 30 µL of this solution B were transferred to a clean cuvette and diluted with 970 µL of DMA. The cuvette was sealed and taken out of the glovebox, placed in the spectrometer and the absorption spectrum of the mixture of $FcBAr^F$ (0.12 mM) + $Ni(COD)_2$ (0.12 mM) was acquired **(red line)**.

(red line) The solution has absorption from 320 to 400 nm whereas the characteristic ferrocenium absorption from 500 to 650 nm disappears. Weak absorption of ferrocene from 400 to 500 nm can also be observed.

In a glovebox, NaI (12.0 mg, 80.0 mmol, 0.16 mM) was added to solution B. The resulting suspension was stirred until NaI was completely dissolved to form solution C. Then, 30 µL of solution C were transferred to a cuvette and diluted with 970 µL of DMA. The cuvette was sealed, taken out of the glovebox, placed in the spectrometer and the absorption spectrum of the mixture of $FcBAr^F$ (0.12 mM) + $Ni(COD)_2$ (0.12 mM) + NaI (0.48 mM) was acquired **(black line)**.

(black line) The solution has similar absorption pattern as solution B. An absorption of *d*-*d* transition of a possible Ni(I) species is not detected.

In a glovebox, ferrocene (Fc, 3.7 mg, 0.020 mmol) was dissolved in DMA (5.0 mL, 4.0 mM). Then, 30 µL of this solution were transferred to a cuvette and diluted with 970 µL of DMA. The cuvette was sealed and taken out of the glovebox, placed in the spectrometer and the absorption spectrum of Fc (0.12 mM) was acquired **(green line)**.

(green line) Weak absorption of ferrocene from 400-500 nm is observed.

4 EPR analysis

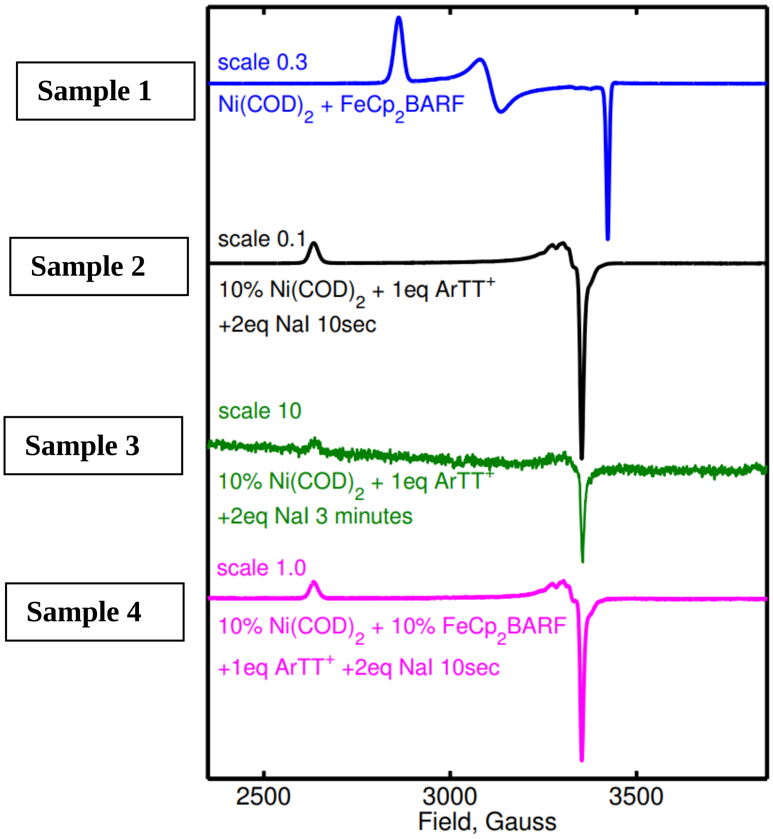

Sample 1:

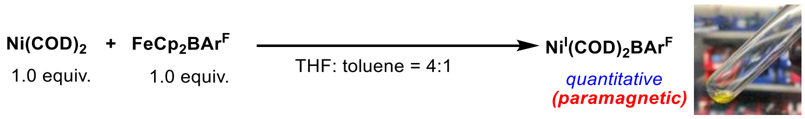

An oven-dried culture tube and a 4.0 mm EPR tube were brought into an argon-filled glovebox. The culture tube was charged with $Ni(COD)_2$ (2.8 mg, 10 µmol, 1.0 equiv.) and $FeCp_2BAr^F$ (10.5 mg, 10.0 µmol, 1.00 equiv.). The solids were dissolved in dry and degassed THF (400 µL) and toluene (100 µL). After 20 s, the solution was transferred to the EPR tube. Then, outside the glovebox, the sample was frozen to 77 K with liquid nitrogen and introduced into the EPR instrument. An intense signal consistent with a nickel(I) similar to the reported one[2] was detected.

Sample 2:

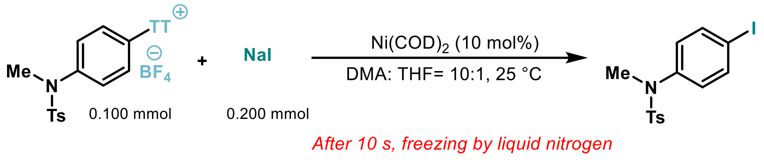

Preparation of the Ni(COD)$_2$ solution in THF: An oven-dried culture tube was brought into an argon-filled glovebox. The tube was charged with Ni(COD)$_2$ (2.8 mg, 10 µmol, 1.0 equiv.), and dissolved with dry and degassed THF (400 µL).

NOTE: The Ni(COD)$_2$ solution was prepared and used directly.

An oven-dried culture tube and a 5.0 mm EPR tube were brought into an argon-filled glovebox. The tube was charged with aryl thianthrenium salt **1** (56.3 mg, 0.100 mmol, 1.00 equiv.), NaI (30.0 mg, 0.200 mmol, 2.00 equiv.) and dissolved in DMA (400 µL). The resulting solution was transferred into the EPR tube and taken outside the glovebox. Then, the stock solution of Ni(COD)$_2$ (40 µL, 25 mM) was added into the EPR tube, and after ***10 s***, the sample was frozen to 77 K with liquid nitrogen and introduced in the EPR instrument; one paramagnetic Ni species distinct from the putative cationic Ni(I) from sample 1 could be observed.

Sample 3:

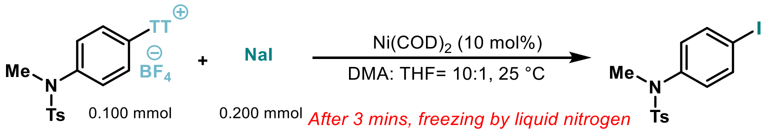

Preparation of the Ni(COD)$_2$ solution in THF: An oven-dried culture tube was brought into an argon-filled glovebox. The tube was charged with Ni(COD)$_2$ (2.8 mg, 0.10 mmol, 1.0 equiv.) and dissolved with dry and degassed THF (400 µL).

NOTE: The Ni(COD)$_2$ solution was prepared and used directly.

An oven-dried culture tube and 5.0 mm EPR tube were brought into an argon-filled glovebox. The tube was charged with aryl thianthrenium salt **1** (56.3 mg, 0.100 mmol, 1.00 equiv.) and NaI (30.0 mg, 0.200 mmol, 2.00 equiv.), and dissolved with DMA (400 µL). The solution was transferred

into the EPR tube and taken outside the glovebox. Then, the stock solution of $Ni(COD)_2$ (40 µL, 25 mM) was added. After ***3 mins***, the tube was frozen to 77 K with liquid nitrogen and introduced into the EPR instrument. *Ca*. 1% of the paramagnetic signal observed in sample 2 was obtained.

Sample 4:

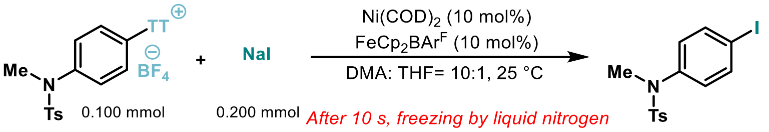

Preparation of the $Ni(COD)_2$ / $FeCp_2BAr^F$ solution in THF: An oven-dried culture tube was brought into an argon-filled glovebox. The tube was charged with $Ni(COD)_2$ (2.8 mg, 10 µmol, 1.0 equiv.), $FeCp_2BAr^F$ (10.5 mg, 10.0 µmol, 1.00 equiv.) and dissolved with dry and degassed THF (400 µL).

NOTE: the solution was prepared and used directly.

An oven-dried culture tube and 5.0 mm EPR tube were brought into an argon-filled glovebox. The tube was charged with aryl thianthrenium salt **1** (56.3 mg, 0.100 mmol, 1.00 equiv.), NaI (30.0 mg, 0.200 mmol, 2.00 equiv.) and dissolved with DMA (400 µL).The solution was transferred into the EPR tube and taken outside the glovebox. Then, the stock solution of $Ni(COD)_2$ /$FeCp_2Bar^F$ (40 µL, 25 mM) was added. After ***10 s***, the EPR tube was frozen to 77 K with liquid nitrogen and introduced into the EPR instrument. A paramagnetic signal to that in sample 2 and 3 was observed, which suggest similar species in catalysis.

5 Competition and radical trap experiments

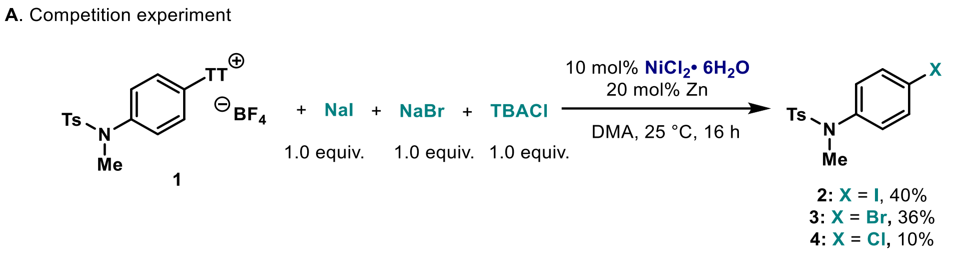

A culture tube with a Teflon-coated stir bar was brought into an argon-filled glovebox, and charged with aryl thianthrenium salt **1** (56.3 mg, 0.100 mmol, 1.00 equiv.), $NiCl_2$•$6H_2O$ (2.4 mg, 0.010 mmol, 10 mol%), Zn (1.3 mg, 0.020 mmol, 20 mol%), NaI (15.0 mg, 0.100 mmol, 1.00 equiv.), NaBr (10.2 mg, 0.100 mmol, 1.00 equiv.) and TBACl (27.8 mg, 0.100 mmol, 1.00 equiv.). Then, outside the glovebox DMA (0.5 mL) was added and the mixture stirred for 16 h at 25 °C. After this time, the mixture was diluted with MTBE, washed with brine, and dried over Na_2SO_4. Upon filtration, the organic layer was concentrated under reduced pressure (water bath at 40 °C). Yields were determined by crude ^{1}H NMR using 1,3,5-trimethoxybenzene as internal standard.

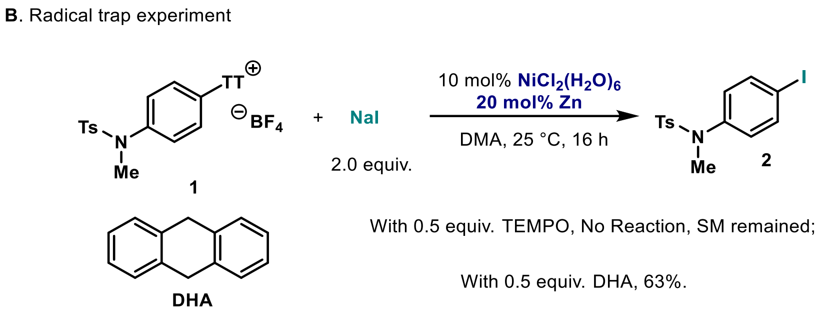

A culture tube with a Teflon-coated stir bar was brought into an argon-filled glovebox, and charged with aryl thianthrenium salt **1** (56.3 mg, 0.100 mmol, 1.00 equiv.), $NiCl_2$•$6H_2O$ (2.4 mg, 0.010 mmol, 10 mol%), Zn (1.3 mg, 0.020 mmol, 20 mol%), NaI (30.0 mg, 0.200 mmol, 2.00 equiv.) and *TEMPO (7.8 mg, 0.050 mmol, 0.50 equiv.) or DHA (9.0 mg, 0.050 mmol, 0.50 equiv.)*. Then, outside the glovebox DMA (0.5 mL) was added and the mixture stirred for 16 h at 25 °C. After this time, the mixture was diluted with MTBE, washed with brine, and dried over Na_2SO_4. Upon filtration, the organic layer was concentrated under reduced pressure (water bath at 40 °C). Yields were determined by crude ^{1}H NMR using 1,3,5-trimethoxybenzene as internal standard.

6 Results of fluorination and other MX salts

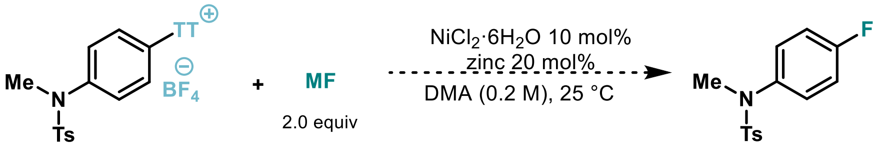

MF	Results
TBAT	SM remained
TASF	SM remained
ZnF2	SM remained
KF	SM remained
NaF	SM remained

NOTE: After the reaction, the starting material remained and no product is observed.

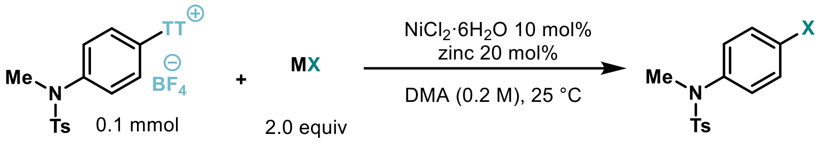

MX	coupling product
KOAc	ND, the SM remained
$PhCO_2Na$	ND, the SM remained
$PhSO_2Na$	ND, the SM remained
TsONa	ND, the SM remained
PhSNa	> 95%, without $NiCl_2 \cdot 6H_2O$ and zinc, in dark

NOTE: After the reaction, the starting material remained and no product is observed.

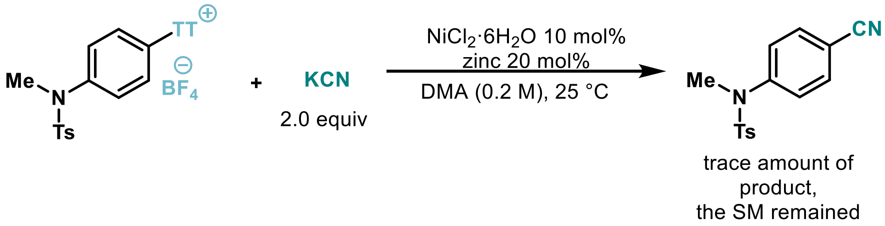

NOTE: After the reaction, the starting material remained, trace amount of product detected by GC-MS and HRMS.

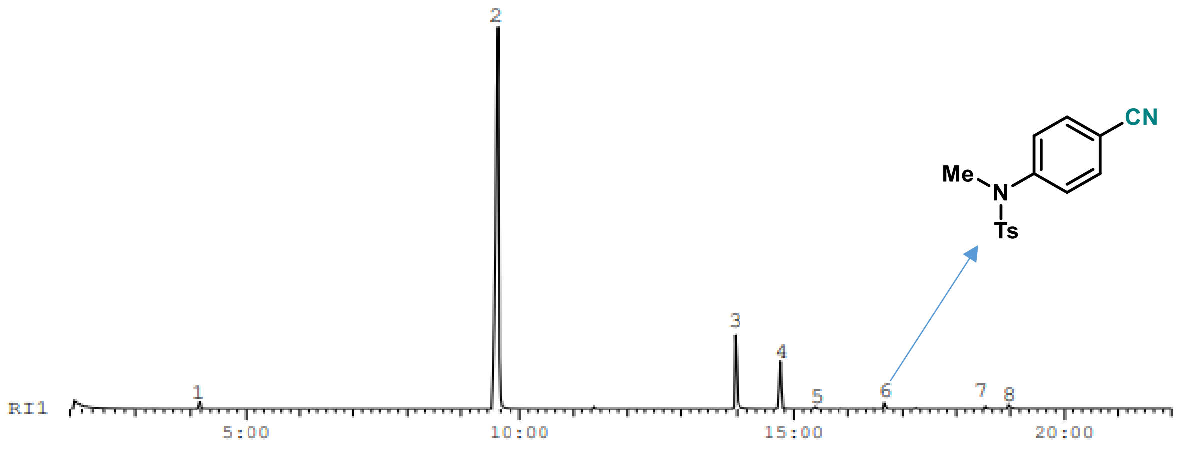

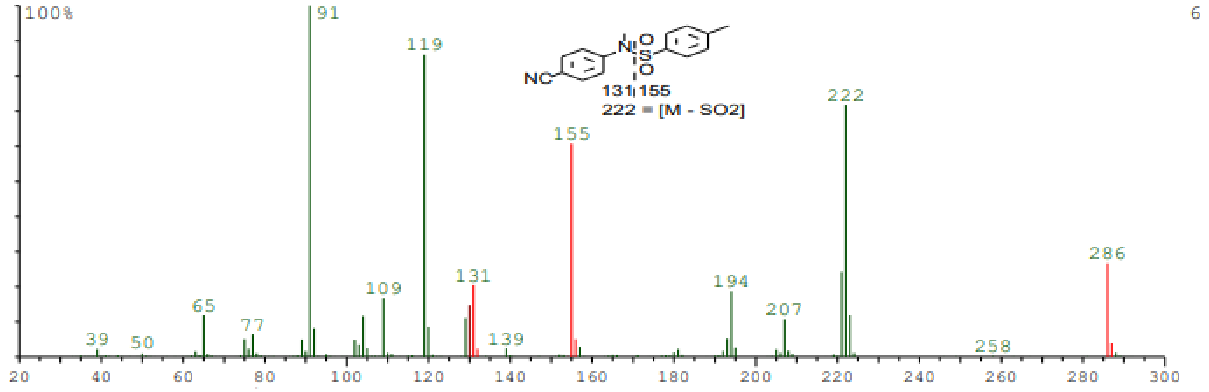

Peak 6

Mass to be matched (m/z): 286.077220 Charge: 1

Mass Tolerance: ±0.005000
Restriction of atom numbers:

C	H	N	O	S
1-110	1-100	2-2	1-5	1-1

Number of calculated Formulas: 1

Formula	Diff.(ppm)	theor. m/z
C15 H14 N2 O2 S1	-0.60	286.077050

23.01.2023
File: E41235a-00.raw
Analyse: NIS-NA-206-01
COP: Shengyang Ni

Messung:	GC-MS
Ionisierung:	GC-EI
Spektrometer:	QExactiveGC
Säule:	MS 85 ZB-5MSplus
Länge:	30
Temp.:	35-15-320-3
GC-Nr.:	-
MS-Nr.:	
Auswerter:	Margold (2242)

.

7 Iodination of Arenes using reported methods

The data was retrieved from a previous report from one of us. [3]

	NIS, $AgNTf_2$, DCM, 4 h	NIS, HFIP, 16 h, 28 °C	NH_4I, H_2SO_4, DMSO, EtOAc, 16 h, 60 °C	NIS, CSA, IPr·HCl, Dioxane, 24 h, 28 °C
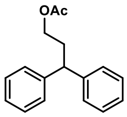	2 isomers[a]	Conversion < 10%<br>2 isomers[a]	no reaction[a]	no reaction[a]
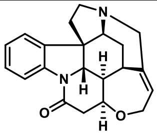	no reaction	Conversion < 5%<br>>1 isomer	no reaction	Conversion < 5%<br>>1 isomer

[a] 3 d reaction time.

8 Preliminary kinetic studies

We attempted various kinetic studies during the course of the investigation. However, we faced several hurdles. Since the optimal catalytic system of Ni(II) salts in combination with Zn resulted in an insoluble mixture, we performed the studies using $Ni(COD)_2$. When using 10 mol% $Ni(COD)_2$ at 25 °C, the reaction already afforded 45% yield after 10 seconds, and reached completion after 50 seconds. In an attempt to slow down the reaction, we attempted the monitoring of the reaction using 1 mol% $Ni(COD)_2$ at 0 °C. However, the reaction was completed after 3 minutes, which corresponded to the acquisition of 1 sole ^{1}H NMR spectra. Further lowering the temperature resulted troublesome due to the freezing point of DMA (–20 °C). As depicted in the optimization table, other solvents such as DMF did not afford any product. Further lowering the catalyst loading did not afford any product, even after prolonged reaction times.

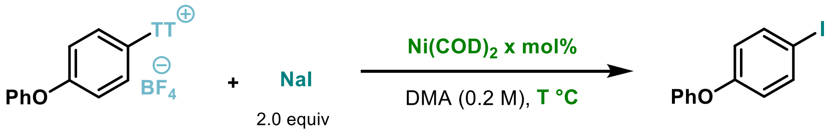

reaction conditions	yield%[a]
$Ni(COD)_2$ 10 mol%, 10 s, 25 °C	46%
$Ni(COD)_2$ 10 mol%, 3 min, 25 °C	91%
$Ni(COD)_2$ 1 mol%, 3 min, 0 °C	88%
$Ni(COD)_2$ 0.1 mol%, 3 min, 25 °C	trace

[a] Yield determined by GC by using 1,3,5-trimethoxybenzene as the internal standard.

We have also attempted to use UV-vis at a low concentrations to monitor the disappearance of the starting material (aryl TT salt) and identify the initiation stage. Again here however, due to the rapid reaction rate (even under diluted conditions), we were unable to identify an induction period of the reaction. (see below)

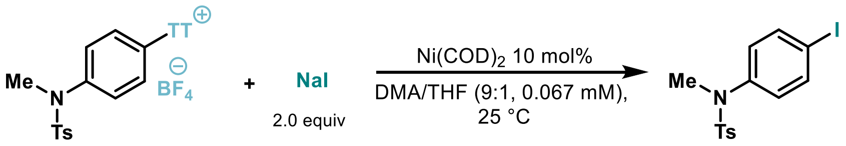

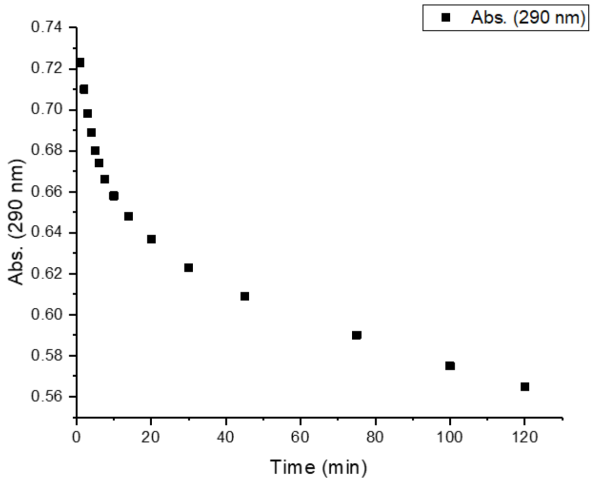
Abs. (290 nm)
0.74
0.72
0.70
0.68
0.66
0.64
0.62
0.60
0.58
0.56
Abs. (290 nm)
0
20
40
60
80
100
120
Time (min)

9 List of 2-step procedure table

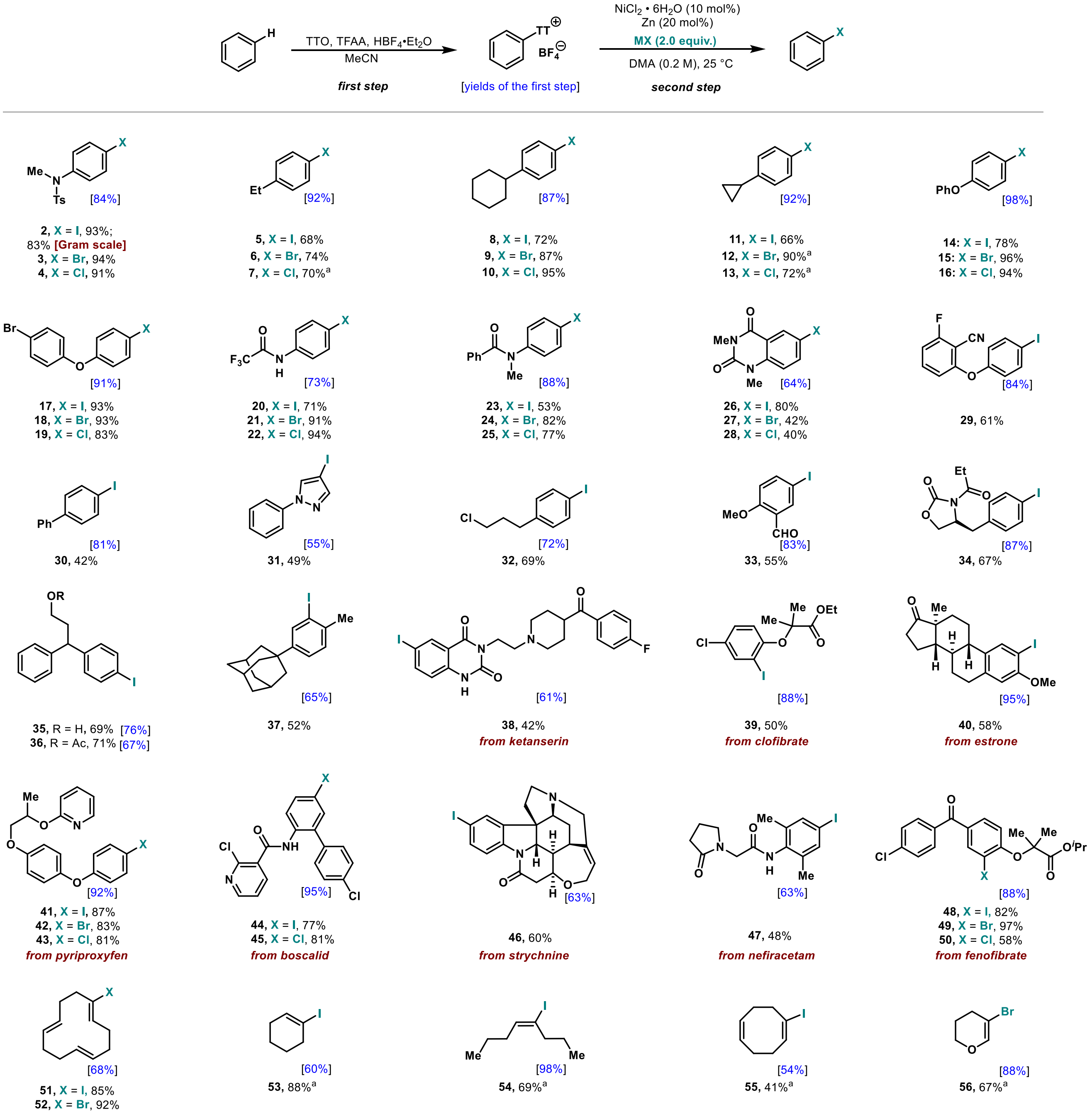

The preparation and yields of aryl thianthrenium salts was according to the literature.[1]

10 Procedure for catalytic halogenation of aryl thianthrenium salts (with *Ni^{II}+Zn*) (Procedure A)

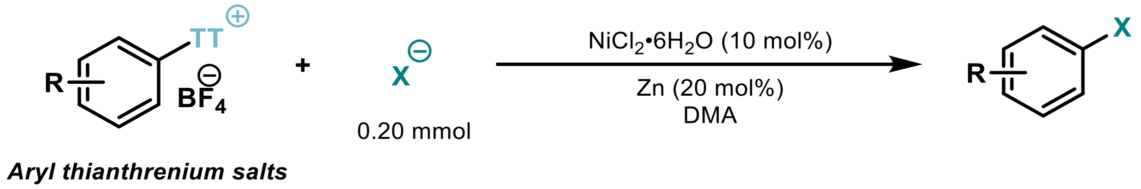

A culture tube equipped with a Teflon-coated stir bar was charged with aryl thianthrenium salt (0.100 mmol, 1.00 equiv.), $NiCl_2$•$6H_2O$ (2.4±0.2 mg, 0.01 mmol, 10 mol%), Zn (1.3±0.2 mg, 0.02 mmol, 20 mol%) and the halogen source [*for iodination, NaI* (30.0±0.5 mg, 0.20 mmol, 2.0 equiv.)*; for bromination, NaBr* (20.2±0.5 mg, 0.20 mmol, 2.0 equiv.)*; for chlorination, TBACl* (55.6±0.5 mg, 0.20 mmol, 2.0 equiv.)]. The tube was evacuated and backfilled with argon (three times). Then, DMA (0.5 mL) was added under Ar. The mixture was stirred for 16 h at 25 °C. After this time, the mixture was diluted with MTBE, washed with brine, and dried over Na_2SO_4. Upon filtration, the organic layer was concentrated under reduced pressure (water bath at 40 °C) and purified by flash column chromatography (silica gel) or preparative TLC (pTLC) to afford the desired product.

Note: Slight deviations (±0.2 mg) on the weight of $NiCl_2$•$6H_2O$ and Zn have no effect on the yields of the reaction. It is worth mentioning that purification by pTLC is a standard procedure form the practitioner and does not result from tedious purifications.

The procedure described above does not use a glovebox throughout the set up. However, for commodity due to reagents/solvent storage, we have performed the exploration of the scope using inert atmosphere and glovebox. We have performed control experiments for 3 different compounds and found that that the yields are not affected if the reaction is set up inside or outside of the glovebox.

	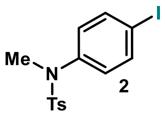 2	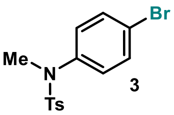 3	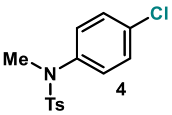 4
	^{1}H NMR yield[a] of 2	^{1}H NMR yield[a] of 3	^{1}H NMR yield[a] of 4
inside glovebox	> 95%	> 95%	> 95%
outside glovebox	> 95%	> 95%	> 95%

[a] Yields were determined by crude ^{1}H NMR using 1,3,5-trimethoxybenzene as internal standard.

11 Procedure for catalytic halogenation of aryl thianthrenium salts (with *Ni⁰*) (Procedure B)

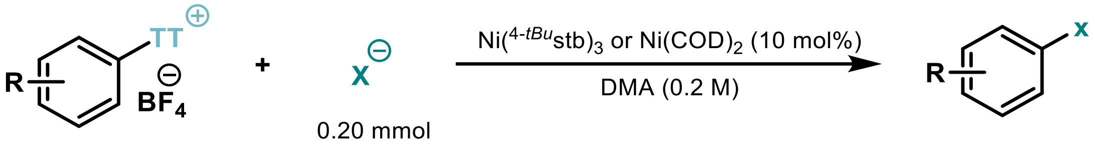

In an argon-filled glovebox, a culture tube equipped with a Teflon-coated stir bar was charged with aryl thianthrenium salt (0.100 mmol, 1.00 equiv.), $Ni(^{4\text{-}tBu}stb)_3$ (10.1±0.2 mg, 0.010 mmol, 10 mol%) *or* $Ni(COD)_2$ (2.8±0.2 mg, 0.01 mmol, 10 mol%) and the halogen source [*for iodination, NaI* (30.0±0.5 mg, 0.20 mmol, 2.0 equiv.)*; for bromination, NaBr* (20.2±0.5 mg, 0.20 mmol, 2.0 equiv.)*; for chlorination, TBACl* (55.6±0.5 mg, 0.20 mmol, 2.0 equiv.)]. Then, DMA (0.5 mL) was added. Outside the glovebox, the mixture was stirred for 16 h at 25 °C. After this time, the mixture was diluted with MTBE, washed with brine, and dried over Na_2SO_4. Upon filtration, the organic layer was concentrated under reduced pressure (water bath at 40 °C) and purified by flash column chromatography (silica gel) or preparative TLC (pTLC) to afford the desired product.

Note: Slight deviations (±0.2 mg) on the weight of $Ni(^{4\text{-}tBu}stb)_3$ or $Ni(COD)_2$ have no effect on the yields of the reaction.

12 Characterization Data

Compound 2

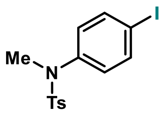

Following **Procedure A** on 0.100 mmol scale. Purification by pTLC (20:1 hexanes:EtOAc) afforded 36.0 mg (93%) of the title compound **2**.

Following **Procedure A** on 5.00 mmol scale. Purification by flash column chromatography (silica gel, 10:1 hexanes:EtOAc) afforded 1.61 g (83%) of the title compound **2**.

Following **Procedure B** on 5.00 mmol scale by using Ni(COD)$_2$. Purification by flash column chromatography (silica gel, 10:1 hexanes:EtOAc) afforded 1.55 g (80%) of the title compound **2**.

Physical State: colorless oil.

R_f = 0.30 (10:1 hexanes:EtOAc).

^{1}H NMR (300 MHz, CDCl$_3$, 298 K) δ 7.66 – 7.57 (m, 2H), 7.48 – 7.38 (m, 2H), 7.29 – 7.20 (m, 2H), 6.90 – 6.80 (m, 2H), 3.12 (s, 3H), 2.42 (s, 3H).
^{13}C NMR (75 MHz, CDCl$_3$, 298 K) δ 143.9, 141.6, 138.1, 133.3, 129.6, 128.5, 128.0, 92.4, 37.9, 21.7.
HRMS (ESI-TOF): calc'd for $C_{14}H_{14}I_1N_1O_2S_1Na_1$ [M+Na]$^+$: 409.9682, found: 409.9685; deviation: –0.6 ppm.

Compound 3

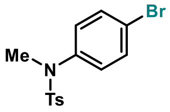

Following **Procedure A** on 0.100 mmol scale. Purification by pTLC (20:1 hexanes:EtOAc) afforded 31.9 mg (94%) of the title compound **3**.

Physical State: white solid.

m.p.: 71 – 73 ºC.

R_f = 0.32 (10:1 hexanes:EtOAc).

^{1}H NMR (300 MHz, CDCl$_3$, 298 K) δ 7.35 (dd, *J* = 8.6, 2.4 Hz, 4H), 7.22 – 7.13 (m, 2H), 6.91 (d, *J* = 8.7 Hz, 2H), 3.06 (s, 3H), 2.35 (s, 3H).
^{13}C NMR (75 MHz, CDCl$_3$, 298 K) δ 144.0, 140.9, 133.3, 132.1, 129.6, 128.3, 128.0, 121.1, 38.1, 21.7.
HRMS (EI-TOF): calc'd for $C_{14}H_{14}N_1O_2S_1Br_1$ $[M]^+$: 338.9923, found: 338.9926; deviation: –0.8 ppm.

Compound 4

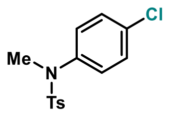

Following **Procedure A** on 0.100 mmol scale. Purification by pTLC (20:1 hexanes:EtOAc) afforded 26.8 mg (91%) of the title compound **4**.

Physical State: light yellow oil.

R_f = 0.29 (10:1 hexanes:EtOAc).

^{1}H NMR (300 MHz, CDCl$_3$, 298 K) δ 7.47 – 7.37 (m, 2H), 7.30 – 7.21 (m, 4H), 7.07 – 7.00 (m, 2H), 3.13 (s, 3H), 2.42 (s, 3H).
^{13}C NMR (75 MHz, CDCl$_3$, 298 K) δ 143.9, 140.3, 133.3, 133.1, 129.6, 129.1, 128.0, 128.0, 38.1, 21.7.
HRMS (EI-TOF): calc'd for $C_{14}H_{14}N_1O_2S_1Cl_1$ $[M]^+$: 295.0428, found: 295.0432; deviation: –1.4 ppm.

Compound 5

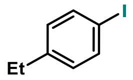

Following **Procedure A** on 0.100 mmol scale. Purification by pTLC (hexanes) afforded 15.7 mg (68%) of the title compound **5**.

Physical State: colorless oil.

R_f = 0.64 (hexanes).

^{1}H NMR (300 MHz, CDCl$_3$, 298 K) δ 7.60 (d, *J* = 8.3 Hz, 2H), 6.96 (d, *J* = 8.4 Hz, 2H), 2.60 (q, *J* = 7.6 Hz, 2H), 1.22 (t, *J* = 7.9 Hz, 3H).
Spectral data is in accordance with previous report.[4]

Compound 6

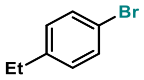

Following **Procedure A** on 0.100 mmol scale. Purification by pTLC (hexanes) afforded 13.6 mg (74%) of the title compound **6**.

Physical State: colorless oil.

R_f = 0.67 (hexanes).

^{1}H NMR (300 MHz, CDCl$_3$, 298 K) δ 7.47 – 7.35 (m, 2H), 7.13 – 7.04 (m, 2H), 2.61 (q, *J* = 7.6 Hz, 2H), 1.23 (t, *J* = 7.6 Hz, 3H).
Spectral data is in accordance with previous report.[5]

Compound 7

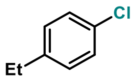

Following **Procedure A** on 0.100 mmol scale. Due to the compound being volatile, the yield (70 %) is determined by ^{1}H NMR using 1,3,5-trimethoxybenzene as the internal standard.

Physical State: colorless oil.

R_f = 0.67 (hexanes).

^{1}**H NMR (300 MHz, CDCl$_3$, 298 K)** δ 7.27 – 7.19 (m, 2H), 7.15 – 7.07 (m, 2H), 2.61 (q, *J* = 7.6 Hz, 2H), 1.21 (t, *J* = 7.6 Hz, 2H).

Spectral data is in accordance with previous report.[6]

Compound 8

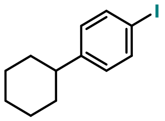

Following **Procedure A** on 0.100 mmol scale. Purification by pTLC (hexanes) afforded 20.6 mg (72%) of the title compound **8**.

Physical State: colorless oil.

R_f = 0.60 (hexanes).

^{1}**H NMR (300 MHz, CDCl$_3$, 298 K)** δ 7.63 – 7.55 (m, 2H), 7.02 – 6.90 (m, 2H), 2.53 – 2.35 (m, 1H), 1.94 – 1.68 (m, 5H), 1.50 – 1.26 (m, 5H).
^{13}**C NMR (75 MHz, CDCl$_3$, 298 K)** δ 147.9, 137.4, 129.1, 90.8, 44.3, 34.4, 26.9, 26.2.
HRMS (EI-TOF): calc'd for $C_{12}H_{15}I_1$ $[M]^+$: 286.0213, found: 286.0217; deviation: –1.4 ppm.

Compound 9

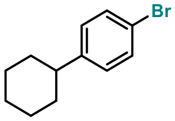

Following **Procedure A** on 0.100 mmol scale. Purification by pTLC (hexanes) afforded 20.7 mg (87%) of the title compound **9**.

Physical State: colorless oil.

R_f = 0.66 (hexanes).

^{1}**H NMR (300 MHz, CDCl$_3$, 298 K)** δ 7.46 – 7.35 (m, 2H), 7.15 – 7.02 (m, 2H), 2.56 – 2.35 (m, 1H), 1.94 – 1.68 (m, 5H), 1.49 – 1.16 (m, 5H).

^{13}C NMR (75 MHz, $CDCl_3$, 298 K) δ 147.2, 131.4, 128.8, 119.5, 44.2, 34.5, 26.9, 26.2.
Spectral data is in accordance with previous report.[7]

Compound 10

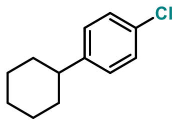

Following **Procedure A** on 0.100 mmol scale. Purification by pTLC (hexanes) afforded 18.4 mg (95%) of the title compound **10**.

Physical State: colorless oil.

R_f = 0.68 (hexanes).

^{1}H NMR (300 MHz, $CDCl_3$, 298 K) δ 7.28 – 7.20 (m, 2H), 7.17 – 7.08 (m, 2H), 2.55 – 2.37 (m, 1H), 1.90 – 1.67 (m, 5H), 1.47 – 1.16 (m, 5H).
^{13}C NMR (75 MHz, $CDCl_3$, 298 K) δ 146.7, 131.4, 128.5, 128.3, 44.1, 34.6, 27.0, 26.2.
HRMS (EI-TOF): calc'd for $C_{12}H_{15}Cl_1$ $[M]^+$: 194.0857, found: 194.0859; deviation: –1.4 ppm.

Compound 11

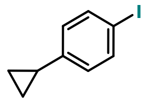

Following **Procedure A** on 0.100 mmol scale. Purification by pTLC (hexanes) afforded 16.0 mg (66%) of the title compound **11**.

Physical State: colorless oil.

R_f = 0.65 (hexanes).

^{1}H NMR (300 MHz, $CDCl_3$, 298 K) δ 7.59 – 7.50 (m, 2H), 6.85 – 6.77 (m, 2H), 1.91 – 1.76 (m, 1H), 1.04 – 0.91 (m, 2H), 0.70 – 0.58 (m, 2H).
^{13}C NMR (75 MHz, $CDCl_3$, 298 K) δ 144.0, 137.3, 128.0, 90.0, 15.2, 9.5.
Spectral data is in accordance with previous report.[8]

Compound 12

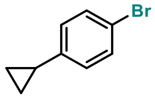

Following **Procedure A** on 0.100 mmol scale. Due to the compound being volatile, the yield (90 %) was determined by ^{1}H NMR using 1,3,5-trimethoxybenzene as the internal standard.

Physical State: colorless oil.

R_f = 0.65 (hexanes).

^{1}H NMR (300 MHz, $CDCl_3$, 298 K) δ 7.39 – 7.32 (m, 2H), 6.97 – 6.90 (m, 2H), 1.92 – 1.78 (m, 1H), 1.02 – 0.93 (m, 2H), 0.71 – 0.60 (m, 2H).
Spectral data is in accordance with previous report.[9]

Compound 13

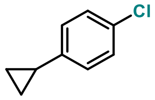

Following **Procedure A** on 0.100 mmol scale. Due to the compound being volatile, the yield (72%) was determined by ^{1}H NMR using 1,3,5-trimethoxybenzene as the internal standard.

Physical State: colorless oil.

R_f = 0.65 (hexanes).

^{1}H NMR (300 MHz, $CDCl_3$, 298 K) δ 7.25 – 7.20 (m, 2H), 7.05 – 6.96 (m, 2H), 1.94 – 1.81 (m, 1H), 1.02 – 0.93 (m, 2H), 0.70 – 0.59 (m, 2H).
Spectral data is in accordance with previous report.[8]

Compound 14

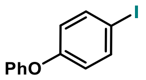

Following **Procedure A** on 0.100 mmol scale. Purification by pTLC (hexanes) afforded 23.1 mg (78%) of the title compound **14**.

Physical State: white solid.

m.p.: 105 – 107 ºC.

R_f = 0.25 (hexanes).

^{1}H NMR (300 MHz, CDCl$_3$, 298 K) δ 7.67 – 7.56 (m, 2H), 7.40 – 7.28 (m, 2H), 7.17 – 7.09 (m, 1H), 7.05 – 6.97 (m, 2H), 6.82 – 6.73 (m, 2H).
^{13}C NMR (75 MHz, CDCl$_3$, 298 K) δ 157.6, 156.7, 138.8, 130.0, 123.9, 121.0, 119.3, 86.0.
Spectral data is in accordance with previous report.[9]

Compound 15

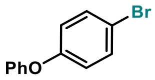

Following **Procedure A** on 0.100 mmol scale. Purification by pTLC (hexanes) afforded 23.8 mg (96%) of the title compound **15**.

Physical State: colorless oil.

R_f = 0.31 (hexanes).

^{1}H NMR (300 MHz, CDCl$_3$, 298 K) δ 7.48 – 7.40 (m, 2H), 7.39 – 7.31 (m, 2H), 7.18 – 7.08 (m, 1H), 7.05 – 6.96 (m, 2H), 6.93 – 6.85 (m, 2H).
Spectral data is in accordance with previous report.[10]

Compound 16

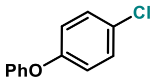

Following **Procedure A** on 0.100 mmol scale. Purification by pTLC (hexanes) afforded 19.2 mg (94%) of the title compound **16**.

Physical State: white solid.

m.p.: 133 – 135 ºC.

R_f = 0.31 (hexanes).

^{1}H NMR (300 MHz, $CDCl_3$, 298 K) δ 7.40 – 7.23 (m, 4H), 7.17 – 7.08 (m, 1H), 7.06 – 6.89 (m, 4H).

^{13}C NMR (75 MHz, $CDCl_3$, 298 K) δ 157.0, 156.1, 130.0, 129.9, 128.4, 123.8, 120.2, 119.1.

Spectral data is in accordance with previous reports.[11]

Compound 17

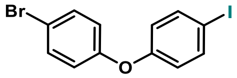

Following **Procedure A** on 0.100 mmol scale. Purification by pTLC (hexanes) afforded 34.8 mg (93%) of the title compound **17**.

Physical State: white solid.

m.p.: 92 – 94 ºC.

R_f = 0.25 (hexanes).

^{1}H NMR (300 MHz, $CDCl_3$, 298 K) δ 7.61 – 7.49 (m, 2H), 7.44 – 7.29 (m, 2H), 6.89 – 6.76 (m, 2H), 6.72 – 6.65 (m, 2H).

^{13}C NMR (75 MHz, $CDCl_3$, 298 K) δ 157.1, 156.0, 139.0, 133.0, 121.1, 120.8, 116.4, 86.7.

Spectral data is in accordance with previous report.[12]

Compound 18

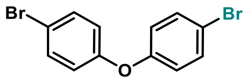

Following **Procedure A** on 0.100 mmol scale. Purification by pTLC (hexanes) afforded 30.3 mg (93%) of the title compound **18**.

Physical State: white solid.

m.p.: 57 – 59 ºC.

R_f = 0.46 (10:1 hexanes:EtOAc).

^{1}H NMR (300 MHz, $CDCl_3$, 298 K) δ 7.49 – 7.39 (m, 4H), 6.94 – 6.83 (m, 4H).
^{13}C NMR (75 MHz, $CDCl_3$, 298 K) δ 156.2, 133.0, 120.7, 116.3.
HRMS (EI-TOF): calc'd for $C_{12}H_8O_1Br_2$ $[M]^+$: 325.8937, found: 325.8941; deviation: –1.3 ppm.

Compound 19

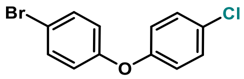

Following **Procedure A** on 0.100 mmol scale. Purification by pTLC (hexanes) afforded 23.4 mg (83%) of the title compound **19**.

Physical State: colorless oil.

R_f = 0.46 (10:1 hexanes:EtOAc).

^{1}H NMR (300 MHz, $CDCl_3$, 298 K) δ 7.49 – 7.39 (m, 2H), 7.36 – 7.26 (m, 2H), 6.99 – 6.81 (m, 4H).
^{13}C NMR (75 MHz, $CDCl_3$, 298 K) δ 156.3, 155.6, 133.0, 130.0, 128.9, 120.6, 120.3, 116.3.
HRMS (EI-TOF): calc'd for $C_{12}H_8O_1Br_1Cl_1$ $[M]^+$: 281.9442, found: 281.9447; deviation: –1.9 ppm.

Compound 20

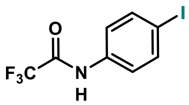

Following **Procedure A** on 0.100 mmol scale. Purification by pTLC (20:1 hexanes:EtOAc) afforded 22.4 mg (71%) of the title compound **20**.

Physical State: white solid.

m.p.: 107 – 109 ºC.

R_f = 0.65 (10:1 hexanes:EtOAc).

^{1}H NMR (300 MHz, $CDCl_3$, 298 K) δ 7.91 (br s, 1H), 7.76 – 7.65 (m, 2H), 7.40 – 7.30 (m, 2H).
^{19}F NMR (282 MHz, $CDCl_3$, 298 K) δ –75.7.
Spectral data is in accordance with previous report.[13]

Compound 21

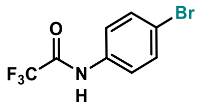

Following **Procedure A** on 0.100 mmol scale. Purification by pTLC (20:1 hexanes:EtOAc) afforded 24.3 mg (91%) of the title compound **21**.

Physical State: white solid.

m.p.: 121 – 123 ºC.

R_f = 0.27 (10:1 hexanes:EtOAc).

^{1}H NMR (300 MHz, $CDCl_3$, 298 K) δ 7.88 (br s, 1H), 7.62 – 7.39 (m, 4H).
^{19}F NMR (282 MHz, $CDCl_3$, 298 K) δ –75.7.
Spectral data is in accordance with previous report.[14]

Compound 22

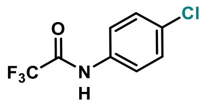

Following **Procedure A** on 0.100 mmol scale. Purification by pTLC (20:1 hexanes:EtOAc) afforded 21.0 mg (94%) of the title compound **22**.

Physical State: white solid.

m.p.: 120 – 122 ºC.

R_f = 0.27 (10:1 hexanes:EtOAc).

^{1}H NMR (300 MHz, $CDCl_3$, 298 K) δ 7.91 (br s, 1H), 7.57 – 7.47 (m, 2H), 7.42 – 7.32 (m, 2H).
^{19}F NMR (282 MHz, $CDCl_3$, 298 K) δ –75.7.
Spectral data is in accordance with previous report.[15]

Compound 23

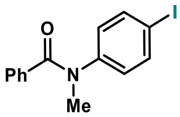

Following **Procedure A** on 0.100 mmol scale. Purification by column chromatography on silica gel (3:1 hexanes:EtOAc) afforded 17.9 mg (53%) of the title compound **23**.

Physical State: white solid.

m.p.: 100 – 102 ºC.

R_f = 0.18 (10:1 hexanes:EtOAc).

^{1}H NMR (300 MHz, $CDCl_3$, 298 K) δ 7.59 – 7.49 (m, 2H), 7.34 – 7.13 (m, 5H), 6.86 – 6.74 (m, 2H), 3.47 (s, 3H).
^{13}C NMR (75 MHz, $CDCl_3$, 298 K) δ 170.7, 144.9, 138.4, 135.7, 130.0, 128.8, 128.8, 128.1, 91.2, 38.4.
HRMS (ESI-TOF): calc'd for $C_{14}H_{12}I_1N_1O_1Na_1$ $[M+Na]^+$: 359.9856, found: 359.9853; deviation: 0.8 ppm.

Compound 24

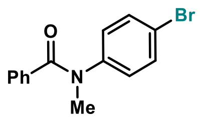

Following **Procedure A** on 0.100 mmol scale. Purification by pTLC (3:1 hexanes:EtOAc) afforded 23.7 mg (82%) of the title compound **24**.

Physical State: white solid.

m.p.: 74 – 76 ºC.

R_f = 0.11 (10:1 hexanes:EtOAc).

^{1}H NMR (300 MHz, $CDCl_3$, 298 K) δ 7.41 – 7.11 (m, 7H), 6.98 – 6.84 (m, 2H), 3.47 (s, 3H).
^{13}C NMR (75 MHz, $CDCl_3$, 298 K) δ 170.7, 144.2, 135.7, 132.4, 130.0, 128.8, 128.5, 128.1, 120.1, 38.5.
HRMS (EI-TOF): calc'd for $C_{14}H_{12}Br_1N_1O_1$ $[M]^+$: 289.0097, found: 289.0099; deviation: –0.6 ppm.

Compound 25

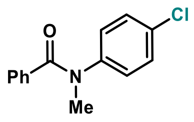

Following **Procedure A** on 0.100 mmol scale. Purification by pTLC (3:1 hexanes:EtOAc) afforded 18.9 mg (77%) of the title compound **25**.

Physical State: white solid.

m.p.: 64 – 66 ºC.

R_f = 0.11 (10:1 hexanes:EtOAc).

^{1}H NMR (300 MHz, $CDCl_3$, 298 K) δ 7.33 – 7.14 (m, 7H), 7.00 – 6.93 (m, 2H), 3.47 (s, 3H).
^{13}C NMR (75 MHz, $CDCl_3$, 298 K) δ 170.7, 143.6, 135.7, 132.2, 130.0, 129.4, 128.8, 128.2, 128.1, 38.5.

HRMS (EI-TOF): calc'd for $C_{14}H_{12}Cl_1N_1O_1$ $[M]^+$: 245.0602, found: 245.0604; deviation: –0.7 ppm.

Compound 26

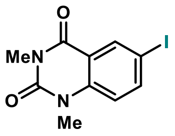

Following **Procedure A** on 0.100 mmol scale. Purification by column chromatography on silica gel (2:1 hexanes:EtOAc) afforded 25.3 mg (80%) of the title compound **26**.

Physical State: white solid.

m.p.: 163 – 165 ºC.

R_f = 0.12 (10:1 hexanes:EtOAc).

^{1}H NMR (300 MHz, $CDCl_3$, 298 K) δ 8.50 (d, *J* = 2.1 Hz, 1H), 7.92 (dd, *J* = 8.8, 2.2 Hz, 1H), 6.96 (d, *J* = 8.8 Hz, 1H), 3.57 (s, 3H), 3.47 (s, 3H).

^{13}C NMR (75 MHz, $CDCl_3$, 298 K) δ 160.8, 151.0, 143.6, 140.1, 137.5, 117.3, 115.7, 85.6, 31.0, 28.8.

HRMS (ESI-TOF): calc'd for $C_{10}H_9I_1N_2O_2Na_1$ $[M+Na]^+$: 338.9601, found: 338.9601; deviation: 0.1 ppm.

Compound 27

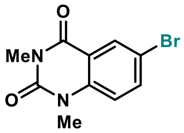

Following **Procedure A** on 0.100 mmol scale. Purification by pTLC (2:1 hexanes:EtOAc) afforded 11.3 mg (42%) of the title compound **27**.

Physical State: white solid.

m.p.: >200 ºC.

R$_f$ = 0.12 (10:1 hexanes:EtOAc).

^{1}H NMR (600 MHz, CDCl$_3$, 298 K) δ 8.34 (dd, *J* = 2.4, 0.4 Hz, 1H), 7.76 (dd, *J* = 8.8, 2.4 Hz, 1H), 7.09 (d, *J* = 8.8 Hz, 1H), 3.59 (s, 3H), 3.48 (s, 3H).
^{13}C NMR (151 MHz, CDCl$_3$, 298 K) δ 161.0, 151.0, 139.5, 138.0, 131.5, 117.1, 116.1, 115.5, 31.1, 28.9.
HRMS (ESI-TOF): calc'd for $C_{10}H_9N_2O_2Br_1Na_1$ [M+Na]$^+$: 290.9740, found: 290.9740; deviation: 0.0 ppm.

Compound 28

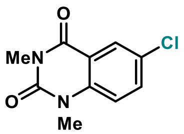

Following **Procedure A** on 0.100 mmol scale. Purification by pTLC (3:1 hexanes:EtOAc) afforded 9.0 mg (40%) of the title compound **28**.

Physical State: white solid.

m.p.: 156 – 158 ºC.

R$_f$ = 0.13 (10:1 hexanes:EtOAc).

^{1}H NMR (600 MHz, CDCl$_3$, 298 K) δ 8.19 (d, *J* = 2.5 Hz, 1H), 7.62 (dd, *J* = 8.9, 2.5 Hz, 1H), 7.15 (d, *J* = 8.9 Hz, 1H), 3.60 (s, 3H), 3.48 (s, 3H).
^{13}C NMR (151 MHz, CDCl$_3$, 298 K) δ 161.1, 151.0, 139.1, 135.2, 128.9, 128.4, 116.8, 115.3, 31.1, 28.8.
HRMS (EI-TOF): calc'd for $C_{10}H_9N_2O_2Cl_1$ [M]$^+$: 224.0347, found: 224.0350; deviation: –1.3 ppm.

Compound 29

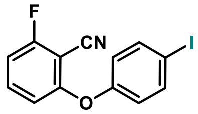

Following **Procedure A** on 0.100 mmol scale. Purification by pTLC (hexanes) afforded 20.7 mg (61%) of the title compound **29**.

Physical State: white solid.

m.p.: 90 – 92 ºC.

R_f = 0.24 (20:1 hexanes:EtOAc).

^{1}H NMR (300 MHz, CDCl$_3$, 298 K) δ 7.79 – 7.66 (m, 2H), 7.51 – 7.38 (m, 1H), 6.98 – 6.81 (m, 3H), 6.66 – 6.58 (m, 1H).
^{13}C NMR (75 MHz, CDCl$_3$, 298 K) δ 165.8, 162.4, 160.3 (d, *J* = 4.1 Hz), 154.5, 139.3, 134.9 (d, *J* = 10.4 Hz), 122.3, 112.0 (d, *J* = 3.5 Hz), 110.8, 110.2 (d, *J* = 19.6 Hz), 89.2.
^{19}F NMR (282 MHz, CDCl$_3$, 298 K) δ –104.2.
HRMS (EI-TOF): calc'd for $C_{13}H_7N_1F_1O_1I_1$ $[M]^+$: 338.9551, found: 338.9554; deviation: –1.0 ppm.

Compound 30

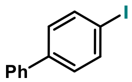

Following **Procedure A** on 0.100 mmol scale. Purification by pTLC (hexanes) afforded 11.8 mg (42%) of the title compound **30**.

Physical State: white solid.

m.p.: 112 – 114 ºC.

R_f = 0.41 (20:1 hexanes:EtOAc).

^{1}H NMR (300 MHz, CDCl$_3$, 298 K) δ 7.81 – 7.73 (m, 2H), 7.58 – 7.52 (m, 2H), 7.49 – 7.40 (m, 2H), 7.40 – 7.30 (m, 3H).
Spectral data is in accordance with previous report.[16]

Compound 31

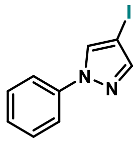

Following **Procedure A** on 0.100 mmol scale. Purification by pTLC (10:1 hexanes:EtOAc) afforded 13.2 mg (49%) of the title compound **31**.

Physical State: white solid.

m.p.: 69 – 71 ºC.

R_f = 0.80 (10:1 hexanes:EtOAc).

^{1}H NMR (300 MHz, $CDCl_3$, 298 K) δ 7.96 (s, 1H), 7.72 (s, 1H), 7.69 – 7.59 (m, 2H), 7.51 – 7.41 (m, 2H), 7.38 – 7.28 (m, 1H).

^{13}C NMR (151 MHz, $CDCl_3$, 298 K) δ 146.1, 139.7, 131.5, 129.7, 127.2, 119.3, 59.0.

HRMS (EI-TOF): calc'd for $C_9H_7N_2I_1$ $[M]^+$: 269.9648, found: 269.9650; deviation: –0.7 ppm.

Compound 32

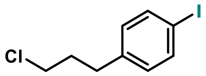

Following **Procedure A** on 0.100 mmol scale. Purification by pTLC (20:1 hexanes:EtOAc) afforded 17.7 mg (69%) of the title compound **32**.

Physical State: colorless oil.

R_f = 0.18 (hexanes).

^{1}H NMR (300 MHz, $CDCl_3$, 298 K) δ 7.61 (d, *J* = 8.3 Hz, 2H), 6.95 (d, *J* = 8.3 Hz, 2H), 3.51 (t, *J* = 6.4 Hz, 2H), 2.73 (t, *J* = 7.4 Hz, 2H), 2.16 – 1.95 (m, 2H).

^{13}C NMR (75 MHz, $CDCl_3$, 298 K) δ 140.5, 137.7, 130.8, 91.4, 44.1, 33.9, 32.4.

HRMS (ESI-TOF): calc'd for $C_9H_{10}Cl_1I_1$ $[M+Na]^+$: 279.9510, found: 279.9515; deviation: –1.6 ppm.

Compound 33

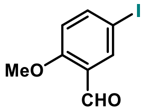

Following **Procedure A** on 0.100 mmol scale. Purification by pTLC (10:1 hexanes:EtOAc) afforded 14.4 mg (55%) of the title compound **33**.

Physical State: white solid.

m.p.: 141 – 143 ºC.

R_f = 0.55 (10:1 hexanes:EtOAc).

^{1}H NMR (300 MHz, $CDCl_3$, 298 K) δ 10.34 (s, 1H), 8.09 (d, *J* = 2.3 Hz, 1H), 7.81 (dd, *J* = 8.8, 2.4 Hz, 1H), 6.78 (d, *J* = 8.8 Hz, 1H), 3.92 (s, 3H).
^{13}C NMR (75 MHz, $CDCl_3$, 298 K) δ 188.4, 161.6, 144.3, 137.3, 126.7, 114.3, 83.1, 56.0.
HRMS (EI-TOF): calc'd for $C_8H_7O_2I_1$ $[M]^+$: 261.9485, found: 261.9486; deviation: –0.4 ppm.

Compound 34

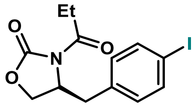

Following **Procedure A** on 0.100 mmol scale. Purification by column chromatography on silica gel (3:1 hexanes:EtOAc) afforded 24.1 mg (67%) of the title compound **34**.

Physical State: white solid.

m.p.: 105 – 107 ºC.

R_f = 0.36 (3:1 hexanes:EtOAc).

^{1}H NMR (300 MHz, CDCl$_3$, 298 K) δ 7.66 (d, *J* = 8.0 Hz, 2H), 6.96 (d, *J* = 8.0 Hz, 2H), 4.71 – 4.55 (m, 1H), 4.21 (t, *J* = 8.5 Hz, 1H), 4.11 (dd, *J* = 9.2, 2.8 Hz, 1H), 3.24 (dd, *J* = 13.4, 3.3 Hz, 1H), 3.04 – 2.88 (m, 2H), 2.73 (dd, *J* = 13.4, 9.5 Hz, 1H), 1.20 (t, *J* = 7.3 Hz, 3H).
^{13}C NMR (75 MHz, CDCl$_3$, 298 K) δ 174.2, 153.5, 138.2, 135.1, 131.5, 93.0, 66.3, 55.1, 37.7, 29.3, 8.4.
HRMS (EI-TOF): calc'd for $C_{13}H_{14}N_1O_3I_1$ $[M]^+$: 359.0013, found: 359.0020; deviation: –2.0 ppm.

Compound 35

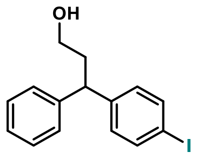

Following **Procedure A** on 0.100 mmol scale. Purification by column chromatography on silica gel (30:1 to 4:1 hexanes:EtOAc) afforded 23.4 mg (69%) of the title compound **35**.

Physical State: colorless oil.

R_f = 0.21 (4:1 hexanes:EtOAc)

^{1}H NMR (500 MHz, CDCl$_3$, 298 K) δ 7.60 (d, *J* = 8.2 Hz, 2H), 7.29 (t, *J* = 7.6 Hz, 2H), 7.24 – 7.15 (m, 3H), 7.01 (d, *J* = 8.1 Hz, 2H), 4.10 (t, *J* = 7.8 Hz, 1H), 3.60 (t, *J* = 6.4 Hz, 2H), 2.37 – 2.18 (m, 2H).

^{13}C NMR (126 MHz, CDCl$_3$, 298 K) δ 144.3, 143.8, 137.6, 130.0, 128.7, 127.8, 126.6, 91.5, 60.8, 46.8, 37.9.

HRMS (EI-TOF): calc'd for $C_{15}H_{15}O_1I_1$ $[M]^+$: 338.0164, found: 338.0162; deviation: –0.6 ppm.

Compound 36

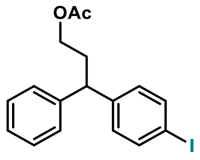

Following **Procedure A** on 0.100 mmol scale. Purification by pTLC (20:1 hexanes:EtOAc) afforded 27.0 mg (71%) of the title compound **36**.

Physical State: colorless oil.

R_f = 0.21 (10:1 hexanes:EtOAc).

^{1}H NMR (300 MHz, $CDCl_3$, 298 K) δ 7.63 – 7.57 (m, 2H), 7.35 – 7.26 (m, 2H), 7.22 – 7.16 (m, 3H), 7.03 – 6.94 (m, 2H), 4.06 – 3.93 (m, 3H), 2.41 – 2.28 (m, 2H), 2.01 (s, 3H).
^{13}C NMR (75 MHz, $CDCl_3$, 298 K) δ 171.1, 143.9, 143.4, 137.8, 130.0, 128.9, 127.8, 126.8, 91.8, 62.8, 47.4, 34.2, 21.0.
HRMS (ESI-TOF): calc'd for $C_{17}H_{17}O_2I_1Na_1$ $[M+Na]^+$: 403.0166, found: 403.0166; deviation: 0.0 ppm.

Compound 37

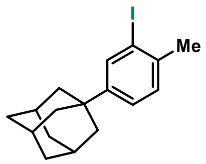

Following **Procedure A** on 0.100 mmol scale. Purification by pTLC (20:1 hexanes:EtOAc) afforded 18.3 mg (52%) of the title compound **37**.

Physical State: colorless oil.

R_f = 0.51 (20:1 hexanes:EtOAc).

^{1}H NMR (300 MHz, $CDCl_3$, 298 K) δ 7.77 (d, *J* = 2.0 Hz, 1H), 7.26 – 7.20 (m, 1H), 7.19 – 7.13 (m, 1H), 2.39 (s, 3H), 2.14 – 2.05 (m, 3H), 1.91 – 1.85 (m, 6H), 1.81 – 1.70 (m, 6H).
^{13}C NMR (75 MHz, $CDCl_3$, 298 K) δ 151.2, 138.5, 135.9, 129.5, 125.0, 101.7, 43.3, 36.8, 35.9, 29.0, 27.6.
HRMS (EI-TOF): calc'd for $C_{17}H_{21}I_1$ $[M]^+$: 352.0682, found: 352.0689; deviation: –1.7 ppm.

Compound 38

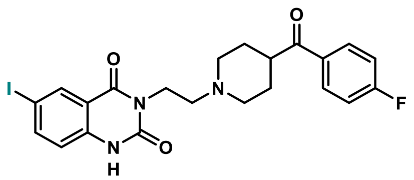

Following **Procedure A** on 0.100 mmol scale. Purification by column chromatography on silica gel (20:1 CH_2Cl_2:MeOH) afforded 21.9 mg (42%) of the title compound **38**.

Physical State: white solid.

m.p.: 155 –157 ºC.

R_f = 0.21 (1:4 hexanes:EtOAc).

^{1}H NMR (600 MHz, $CDCl_3$, 298 K) δ 10.17 (br s, 1H), 8.35 (d, *J* = 2.0 Hz, 1H), 8.00 – 7.90 (m, 2H), 7.81 (dd, *J* = 8.5, 2.0 Hz, 1H), 7.13 (dd, *J* = 8.9, 8.3 Hz, 2H), 6.81 (dd, *J* = 8.5, 0.4 Hz, 1H), 4.23 (t, *J* = 6.6 Hz, 2H), 3.28 – 3.11 (m, 3H), 2.84 – 2.69 (m, 2H), 2.23 (s, 2H), 1.95 – 1.75 (m, 4H).
^{13}C NMR (151 MHz, $CDCl_3$, 298 K) δ 201.2, 165.79 (d, *J* = 254.7 Hz) 161.2, 151.5, 143.5, 138.1, 137.1, 132.5 (d, *J* = 3.2 Hz), 131.0 (d, *J* = 9.2 Hz), 116.8, 116.4, 115.9 (d, *J* = 21.8 Hz), 85.7, 55.7, 53.5, 43.7, 38.4, 28.8.
^{19}F NMR (565 MHz, $CDCl_3$, 298 K) δ –105.4.
HRMS (ESI-TOF): calc'd for $C_{22}H_{22}F_1I_1N_3O_3$ $[M+H]^+$: 522.0684, found: 522.0686; deviation: 0.3 ppm.

Compound 39

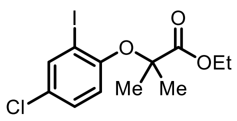

Following **Procedure A** on 0.100 mmol scale. Purification by column chromatography on silica gel (100:1 hexanes:EtOAc) afforded a mixture of the product and clofibrate. Further purification of the mixture by preparative HPLC (YMC-Actus Triart C18 (150×30.0 mmI.D: 5 µm),

CH_3CN/0.1% aqueous TFA = 65:35, flow rate = 42.5 mL/min. 35 °C) provided 18.5 mg (50%) of the title compound **39** (retention time: 12.4 min).

Physical State: colorless oil.

R_f = 0.51 (8:1 hexanes:EtOAc).

^{1}H NMR (500 MHz, $CDCl_3$, 298 K) δ 7.75 (d, *J* = 2.6 Hz, 1H), 7.17 (dd, *J* = 8.8, 2.6 Hz, 1H), 6.69 (d, *J* = 8.8 Hz, 1H), 4.25 (q, *J* = 7.1 Hz, 2H), 1.63 (s, 6H), 1.27 (t, *J* = 7.1 Hz, 3H).

^{13}C NMR (126 MHz, $CDCl_3$, 298 K) δ 173.9, 154.1, 138.9, 128.9, 128.1, 118.2, 91.6, 81.4, 61.8, 25.4, 14.2.

HRMS (EI-TOF): calc'd for $C_{12}H_{14}Cl_1I_1O_3$ $[M]^+$: 367.9672, found: 367.9671; deviation: –0.4 ppm.

Compound 40

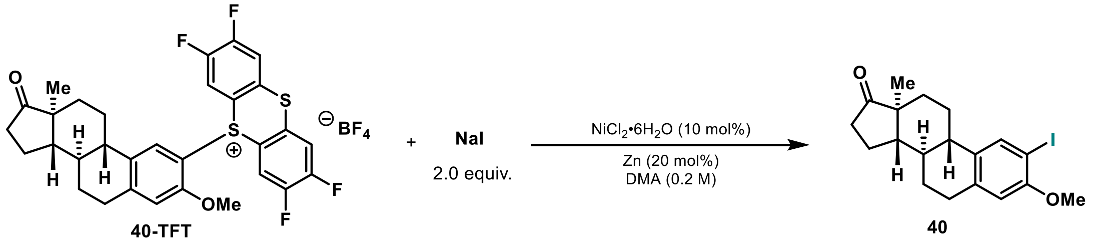

Following **Procedure A** using the corresponding **TFT** salt (65.8 mg, 0.100 mmol). Purification by pTLC (5:1 hexanes:EtOAc) afforded 23.8 mg (58%) of the title compound **40**.

Physical State: white solid.

m.p.: 154 – 157 ºC.

R_f = 0.42 (5:1 hexanes:EtOAc).

^{1}H NMR (300 MHz, $CDCl_3$, 298 K) δ 7.64 (s, 1H), 6.55 (s, 1H), 3.84 (s, 3H), 2.87 (dd, *J* = 8.7, 4.2 Hz, 2H), 2.50 (dd, *J* = 18.4, 8.4 Hz, 1H), 2.38 – 2.34 (m, 1H), 2.26 – 2.16 (m, 1H), 2.15 – 2.06 (m, 1H), 2.05 – 1.87 (m, 3H), 1.66 – 1.37 (m, 6H), 0.90 (s, 3H).

^{13}C NMR (75 MHz, CDCl$_3$, 298 K) δ 220.7, 156.2, 138.3, 136.5, 134.5, 111.5, 82.9, 56.5, 50.4, 48.1, 43.8, 38.3, 36.0, 31.6, 29.7, 26.5, 26.1, 21.7, 14.0.

HRMS (EI-TOF): calc'd for $C_{19}H_{23}O_2I_1$ $[M]^+$: 410.0737, found: 410.0745; deviation: –1.8 ppm.

Compound 41

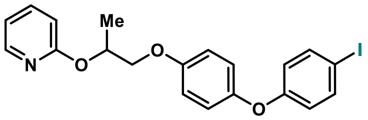

Following **Procedure A** on 0.100 mmol scale. Purification by pTLC (20:1 hexanes:EtOAc) afforded 38.9 mg (87%) of the title compound **41**.

Physical State: white solid.

m.p.: 57 –59 ºC.

R_f = 0.26 (10:1 hexanes:EtOAc).

^{1}H NMR (300 MHz, CDCl$_3$, 298 K) δ 8.19 – 8.12 (m, 1H), 7.61 – 7.52 (m, 3H), 6.97 – 6.90 (m, 4H), 6.86 (ddd, J = 5.0, 4.2, 2.1 Hz, 1H), 6.78 – 6.63 (m, 3H), 5.69 – 5.49 (m, 1H), 4.24 – 4.14 (m, 1H), 4.11 – 3.99 (m, 1H), 1.48 (d, J = 6.4 Hz, 3H).

^{13}C NMR (75 MHz, CDCl$_3$, 298 K) δ 163.3, 158.8, 155.7, 149.7, 146.9, 138.9, 138.6, 121.1, 119.8, 116.9, 116.1, 111.8, 85.0, 71.2, 69.4, 17.2.

HRMS (ESI-TOF): calc'd for $C_{20}H_{19}I_1N_1O_3$ $[M+H]^+$: 448.0404, found: 448.0404; deviation: 0.1 ppm.

Compound 42

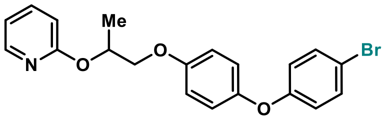

Following **Procedure A** on 0.100 mmol scale. Purification by pTLC (20:1 hexanes:EtOAc) afforded 33.1 mg (83%) of the title compound **42**.

Physical State: white solid.

m.p.: 58 – 60 ºC.

R$_f$ = 0.26 (10:1 hexanes:EtOAc).

^{1}H NMR (300 MHz, CDCl$_3$, 298 K) δ 8.15 (dd, *J* = 5.0, 1.2 Hz, 1H), 7.64 – 7.51 (m, 1H), 7.43 – 7.33 (m, 2H), 6.94 (s, 4H), 6.89 – 6.77 (m, 3H), 6.74 (d, *J* = 8.5 Hz, 1H), 5.70 – 5.47 (m, 1H), 4.27 – 4.14 (m, 1H), 4.14 – 4.00 (m, 1H), 1.48 (d, *J* = 6.5 Hz, 3H).
^{13}C NMR (75 MHz, CDCl$_3$, 298 K) δ 163.3, 157.9, 155.7, 149.9, 146.9, 138.8, 132.6, 121.0, 119.4, 116.9, 116.1, 114.9, 111.8, 71.2, 69.4, 17.2.
HRMS (ESI-TOF): calc'd for $C_{20}H_{19}Br_1N_1O_3$ [M+H]$^+$: 400.0543, found: 400.0540; deviation: 0.8 ppm.

Compound 43

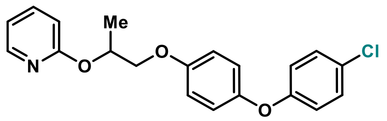

Following **Procedure A** on 0.100 mmol scale. Purification by pTLC (20:1 hexanes:EtOAc) afforded 28.8 mg (81%) of the title compound **43**.

Physical State: white solid.

m.p.: 44 – 46 ºC.

R$_f$ = 0.26 (10:1 hexanes:EtOAc).

^{1}H NMR (300 MHz, CDCl$_3$, 298 K) δ 8.18 – 8.09 (m, 1H), 7.56 (ddd, *J* = 8.4, 7.1, 2.0 Hz, 1H), 7.30 – 7.17 (m, 2H), 6.93 (s, 4H), 6.89 – 6.82 (m, 3H), 6.77 – 6.70 (m, 1H), 5.67 – 5.51 (m, 1H), 4.19 (dd, *J* = 9.9, 5.3 Hz, 1H), 4.07 (dd, *J* = 9.9, 4.9 Hz, 1H), 1.48 (d, *J* = 6.4 Hz, 3H).
^{13}C NMR (75 MHz, CDCl$_3$, 298 K) δ 163.3, 157.4, 155.6, 150.1, 146.9, 138.9, 129.7, 127.5, 120.9, 118.9, 116.9, 116.1, 111.8, 71.2, 69.4, 17.2.
HRMS (ESI-TOF): calc'd for $C_{20}H_{19}Cl_1N_1O_3$ [M+H]$^+$: 356.1048, found: 356.1050; deviation: –0.5 ppm.

Compound 44

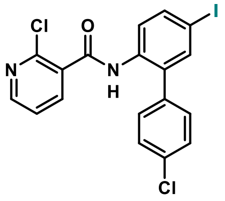

Following **Procedure A** on 0.100 mmol scale. Purification by column chromatography on silica gel (2:1 hexanes:EtOAc) afforded 36.0 mg (77%) of the title compound **44**.

Physical State: white solid.

m.p.: 162 – 164 ºC.

R_f = 0.56 (1:1 hexanes:EtOAc).

^{1}H NMR (600 MHz, $CDCl_3$, 298 K) δ 8.44 (dd, *J* = 4.7, 2.0 Hz, 1H), 8.23 (d, *J* = 8.7 Hz, 1H), 8.19 (s, 1H), 8.15 (dd, *J* = 7.6, 2.0 Hz, 1H), 7.74 (dd, *J* = 8.7, 2.1 Hz, 1H), 7.60 (dd, *J* = 2.1, 0.3 Hz, 1H), 7.45 – 7.43 (m, 2H), 7.35 (dd, *J* = 7.7, 4.7 Hz, 1H), 7.32 – 7.29 (m, 2H).
^{13}C NMR (151 MHz, $CDCl_3$, 298 K) δ 162.4, 151.6, 146.7, 140.5, 138.8, 137.9, 135.2, 134.8, 134.5, 134.1, 130.8, 129.6, 129.4, 123.6, 123.1, 89.0.
HRMS (EI-TOF): calc'd for $C_{18}H_{11}N_2O_1I_1Cl_2$ $[M]^+$: 467.9288, found: 467.9293; deviation: –1.1 ppm.

Compound 45

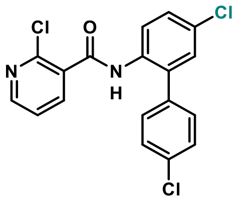

Following **Procedure A** on 0.100 mmol scale. Purification by pTLC (2:1 hexanes:EtOAc) afforded 30.5 mg (81%) of the title compound **45**.

Physical State: white solid.

m.p.: 105 – 107 ºC.

R_f = 0.56 (1:1 hexanes:EtOAc).

^{1}H NMR (600 MHz, $CDCl_3$, 298 K) δ 8.43 (dd, *J* = 4.7, 2.0 Hz, 1H), 8.38 (d, *J* = 8.8 Hz, 1H), 8.19 (s, 1H), 8.14 (dd, *J* = 7.7, 2.0 Hz, 1H), 7.45 – 7.43 (m, 2H), 7.41 (dd, *J* = 9.0, 2.5 Hz, 1H), 7.35 (dd, *J* = 7.7, 4.7 Hz, 1H), 7.33 – 7.30 (m, 2H), 7.25 (dd, *J* = 2.5, 0.4 Hz, 1H).

^{13}C NMR (151 MHz, $CDCl_3$, 298 K) δ 162.5, 151.6, 146.7, 140.5, 135.2, 135.1, 133.8, 133.2, 130.8, 130.8, 130.5, 130.1, 129.6, 128.9, 123.4, 123.1.

HRMS (EI-TOF): calc'd for $C_{18}H_{11}N_2O_1Cl_3$ $[M]^+$: 375.9931, found: 375.9936; deviation: –1.1 ppm.

Compound 46

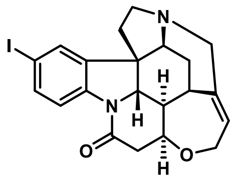

Following **Procedure A** on 0.100 mmol scale. Purification by column chromatography on silica gel (20:1 CH_2Cl_2:MeOH) afforded 27.6 mg (60%) of the title compound **46**.

Physical State: colorless oil.

R_f = 0.35 (10:1 CH_2Cl_2:MeOH).

^{1}H NMR (600 MHz, $CDCl_3$, 298 K) δ 7.86 (dd, *J* = 8.4, 0.4 Hz, 1H), 7.54 (dd, *J* = 8.5, 1.8 Hz, 1H), 7.42 (d, *J* = 1.8 Hz, 1H), 5.89 (td, *J* = 6.3, 3.4 Hz, 1H), 4.27 (dt, *J* = 8.4, 3.3 Hz, 1H), 4.14 (dd, *J* = 13.8, 7.0 Hz, 1H), 4.08 – 4.01 (m, 1H), 3.86 (s, 1H), 3.84 (d, *J* = 10.6 Hz, 1H), 3.68 (dd, *J* = 14.8, 2.0 Hz, 1H), 3.19 (dd, *J* = 10.0, 6.8 Hz, 1H), 3.16 – 3.08 (m, 2H), 2.91 – 2.81 (m, 1H), 2.70 (d, *J* = 14.8 Hz, 1H), 2.64 (dd, *J* = 17.5, 3.3 Hz, 1H), 2.36 (dt, *J* = 14.5, 4.3 Hz, 1H), 1.93 – 1.81 (m, 2H), 1.45 (dt, *J* = 14.5, 2.1 Hz, 1H), 1.24 (dt, *J* = 10.5, 3.2 Hz, 1H).

^{13}C NMR (151 MHz, $CDCl_3$, 298 K) δ 169.6, 142.2, 140.5, 137.6, 135.7, 131.5, 127.4, 118.3, 87.1, 77.6, 64.8, 60.5, 60.4, 52.8, 51.9, 50.4, 48.3, 43.0, 42.6, 31.7, 27.0.

HRMS (ESI-TOF): calc'd for $C_{21}H_{22}I_1N_2O_2$ $[M+H]^-$: 461.0721, found: 461.0724; deviation: –0.7 ppm.

Compound 47

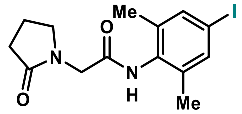

Following **Procedure A** on 0.100 mmol scale. Purification by column chromatography on silica gel (20:1 CH_2Cl_2:MeOH) afforded 17.9 mg (48%) of the title compound **47**.

Physical State: white solid.

m.p.: >200 ºC.

R_f = 0.18 (10:1 CH_2Cl_2:MeOH).

^{1}H NMR (600 MHz, $CDCl_3$, 298 K) δ 7.74 (br s, 1H), 7.42 (s, 2H), 4.08 (s, 2H), 3.65 – 3.54 (m, 2H), 2.47 (dd, J = 8.6, 7.6 Hz, 2H), 2.19 – 2.10 (m, 8H).
^{13}C NMR (151 MHz, $CDCl_3$, 298 K) δ 176.5, 167.1, 137.6, 137.2, 133.5, 93.0, 49.0, 48.2, 30.5, 18.4, 18.1.
HRMS (EI-TOF): calc'd for $C_{14}H_{17}N_2O_2I_1$ $[M]^+$: 372.0329, found: 372.0333; deviation: –1.1 ppm.

Compound 48

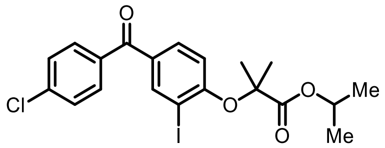

Following **Procedure A** on 0.100 mmol scale. Purification by column chromatography on silica gel (30:1 hexanes:EtOAc) afforded 39.8 mg (82%) of the title compound **48**.

Physical State: colorless oil.

Rf = 0.36 (8:1 hexanes:EtOAc)

^{1}H NMR (500 MHz, CDCl$_3$, 298 K) δ 8.25 (d, *J* = 2.1 Hz, 1H), 7.69 (d, *J* = 8.5 Hz, 2H), 7.66 (dd, *J* = 8.6, 2.2 Hz, 1H), 7.46 (d, *J* = 8.5 Hz, 2H), 6.72 (d, *J* = 8.6 Hz, 1H), 5.08 (hept, *J* = 6.3 Hz, 1H), 1.71 (s, 6H), 1.21 (d, *J* = 6.2 Hz, 6H).

^{13}C NMR (126 MHz, CDCl$_3$, 298 K) δ 193.0, 172.8, 158.8, 141.9, 138.9, 135.9, 132.1, 131.4, 131.3, 128.8, 115.2, 89.6, 81.4, 69.6, 25.5, 21.7.

HRMS (ESI-TOF): calc'd for $C_{20}H_{20}Cl_1I_1Na_1O_4$ $[M+Na]^+$: 508.9985, found: 508.9987; deviation: 0.4 ppm.

Compound 49

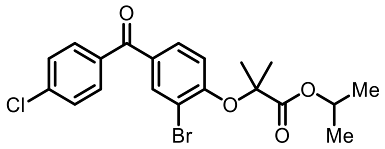

Following **Procedure A** on 0.100 mmol scale. Purification by column chromatography on silica gel (30:1 hexanes:EtOAc) afforded 42.5 mg (97%) of the title compound **49**.

Physical State: colorless oil.

R_f = 0.36 (8:1 hexanes:EtOAc)

^{1}H NMR (500 MHz, CDCl$_3$, 298 K) δ 8.02 (d, *J* = 2.2 Hz, 1H), 7.69 (d, *J* = 8.2 Hz, 2H), 7.63 (dd, *J* = 8.7, 2.2 Hz, 1H), 7.46 (d, *J* = 8.3 Hz, 2H), 6.81 (d, *J* = 8.6 Hz, 1H), 5.09 (hept, *J* = 6.3 Hz, 1H), 1.69 (s, 6H), 1.22 (d, *J* = 6.3 Hz, 6H).

^{13}C NMR (126 MHz, CDCl$_3$, 298 K) δ 193.2, 172.8, 156.5, 138.9, 135.9, 135.7, 131.6, 131.3, 130.3, 128.9, 116.7, 115.0, 81.3, 69.6, 25.4, 21.7.

HRMS (ESI-TOF): calc'd for $C_{20}H_{20}Cl_1Br_1Na_1O_4$ $[M+Na]^+$: 461.0124, found: 461.0126; deviation: 0.5 ppm.

Compound 50

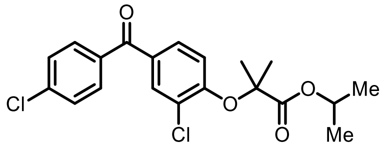

Following **Procedure A** on 0.100 mmol scale. Purification by column chromatography on silica gel (35:1 hexanes:EtOAc) afforded 22.9 mg (58%) of the title compound **50**.

Physical State: colorless oil.

R_f = 0.37 (8:1 hexanes:EtOAc)

^{1}H NMR (500 MHz, $CDCl_3$, 298 K) δ 7.86 (d, *J* = 2.1 Hz, 1H), 7.70 (d, *J* = 8.5 Hz, 2H), 7.59 (dd, *J* = 8.6, 2.2 Hz, 1H), 7.47 (d, *J* = 8.4 Hz, 2H), 6.85 (d, *J* = 8.6 Hz, 1H), 5.10 (hept, *J* = 6.3 Hz, 1H), 1.69 (s, 6H), 1.22 (d, *J* = 6.3 Hz, 6H).

^{13}C NMR (126 MHz, $CDCl_3$, 298 K) δ 193.3, 172.9, 155.6, 139.0, 135.9, 132.6, 131.3, 129.6, 128.9, 125.7, 117.0, 81.2, 69.7, 29.8, 25.4, 21.7.

HRMS (ESI-TOF): calc'd for $C_{20}H_{20}Cl_2Na_1O_4$ $[M+Na]^+$: 417.0629, found: 417.0631; deviation: 0.6 ppm.

Compound 51

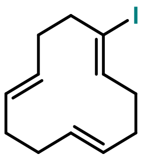

Following **Procedure A** on 0.100 mmol scale. Purification by pTLC (hexanes) afforded 24.5 mg (85%) of the title compound **51**.

Physical State: colorless oil.

R_f = 0.58 (hexanes)

^{1}H NMR (300 MHz, $CDCl_3$, 298 K) δ 5.27 – 5.10 (m, 3H), 5.07 – 4.85 (m, 2H), 2.53 – 2.39 (m, 2H), 2.33 – 2.24 (m, 2H), 2.23 – 2.09 (m, 4H), 2.07 – 1.99 (m, 4H).

^{13}C NMR (75 MHz, $CDCl_3$, 298 K) δ 135.8, 132.2, 131.7, 130.1, 129.3, 110.9, 44.1, 35.8, 32.7, 32.0, 32.0, 31.1.
HRMS (EI-TOF): calc'd for $C_{12}H_{17}I_1$ $[M]^+$: 288.0369, found: 288.0371; deviation: –0.6 ppm.

Compound 52

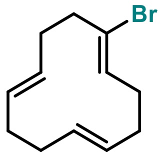

Following **Procedure A** on 0.100 mmol scale. Purification by pTLC (hexanes) afforded 22.1 mg (92%) of the title compound **52**.

Physical State: colorless oil.

R_f = 0.63 (hexanes)

^{1}H NMR (300 MHz, $CDCl_3$, 298 K) δ 5.32 (t, *J* = 7.4 Hz, 1H), 5.22 – 5.07 (m, 2H), 5.06 – 4.94 (m, 2H), 2.47 – 2.39 (m, 2H), 2.33 – 2.17 (m, 4H), 2.14 – 1.99 (m, 6H).
^{13}C NMR (75 MHz, $CDCl_3$, 298 K) δ 132.4, 131.6, 130.4, 129.7, 129.3, 128.6, 41.1, 32.7, 32.1, 32.1, 30.7, 30.2.
HRMS (EI-TOF): calc'd for $C_{12}H_{17}Br_1$ $[M]^+$: 240.0508, found: 240.0505; deviation: 1.4 ppm.

Compound 53

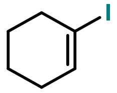

Following **Procedure A** on 0.100 mmol scale. Due to the compound is volatile, the yield (88%) is determined by ^{1}H NMR using 1,3,5-trimethoxybenzene as the internal standard. Purification by pTLC (hexanes) afforded the title compound **53**.

Physical State: colorless oil.

R_f = 0.67 (hexanes)

^{1}H NMR (300 MHz, CDCl$_3$, 298 K) δ 6.34 (tt, *J* = 4.0, 1.8 Hz, 1H), 2.55 – 2.43 (m, 2H), 2.21 – 2.09 (m, 2H), 1.70 – 1.64 (m, 4H).

HRMS (EI-TOF): calc'd for $C_6H_9I_1$ $[M]^+$: 207.9743, found: 207.9744; deviation: –0.3 ppm.

Spectral data is in accordance with previous report.[17]

Compound 54

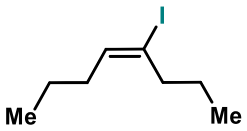

Following **Procedure A** on 0.100 mmol scale. Due to the compound is volatile, the yield (69%) is determined by ^{1}H NMR using 1,3,5-trimethoxybenzene as the internal standard. Purification by pTLC (hexanes) afforded the title compound **54**.

Physical State: colorless oil.

R_f = 0.48 (hexanes)

^{1}H NMR (300 MHz, CDCl$_3$, 298 K) δ 6.27 – 6.09 (m, 1H), 2.40 – 2.28 (m, 2H), 2.03 (q, *J* = 7.4 Hz, 2H), 1.61 – 1.46 (m, 2H), 1.46 – 1.32 (m, 2H), 0.91 (t, *J* = 7.4 Hz, 6H).

^{13}C NMR (75 MHz, CDCl$_3$, 298 K) δ 141.7, 103.6, 40.4, 33.1, 22.6, 22.5, 13.8, 12.9.

HRMS (ESI-TOF): calc'd for $C_8H_{15}I_1$ $[M]^+$: 238.0213, found: 238.0216; deviation: –1.1 ppm.

Compound 55

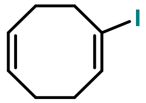

Following **Procedure A** on 0.100 mmol scale. Due to the compound is volatile, the yield (41%) is determined by ^{1}H NMR using 1,3,5-trimethoxybenzene as the internal standard. Purification by pTLC (hexanes) afforded the title compound **55**.

Physical State: colorless oil.

R_f = 0.65 (hexanes)

^{1}H NMR (300 MHz, $CDCl_3$, 298 K) δ 6.42 – 6.24 (m, 1H), 5.63 – 5.50 (m, 2H), 2.96 – 2.86 (m, 2H), 2.40 – 2.28 (m, 6H).
^{13}C NMR (75 MHz, $CDCl_3$, 298 K) δ 139.4, 128.4, 128.1, 100.9, 43.2, 30.6, 27.9, 27.5.

HRMS (EI-TOF): calc'd for $C_8H_{11}I_1$ $[M]^+$: 233.9900, found: 233.9901; deviation: –0.3 ppm.

Compound 56

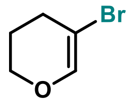

Following **Procedure A** on 0.100 mmol scale. Due to the compound is volatile, the yield (67%) is determined by ^{1}H NMR using 1,3,5-trimethoxybenzene as the internal standard. Purification by pTLC (hexanes) afforded the title compound **56**.

Physical State: colorless oil.

R_f = 0.73 (hexanes)

^{1}H NMR (300 MHz, $CDCl_3$, 298 K) δ 6.53 (t, *J* = 1.8 Hz, 1H), 3.89 – 3.82 (m, 2H), 2.29 (td, *J* = 6.4, 1.8 Hz, 2H), 1.91 – 1.84 (m, 2H).
Spectral data is in accordance with previous report.[18]

Compound 58

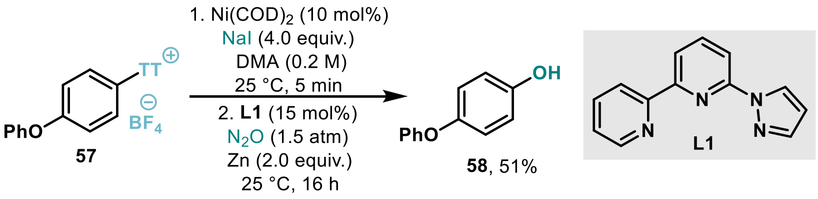

An oven- and heatgun-dried pressure Schlenk tube with a Teflon screw-cap equipped with a Teflon-coated stir bar were used. The Schlenk tube was brought into an argon-filled glovebox. In an argon-filled glovebox, the Schlenk tube was charged with aryl thianthrenium salt **57** (47.2 mg, 0.100 mmol, 1.00 equiv.), $Ni(COD)_2$ (2.8 mg, 0.010 mmol, 10 mol%) and NaI (60.0 mg, 0.400 mmol, 4.00 equiv.). Then, DMA (0.5 mL) was added and the mixture was stirred for 5 min at 25 °C. After that, **L1** (3.3 mg, 0.015 mmol, 15 mol%), zinc (140-325 mesh) (13.0 mg, 0.200 mmol, 2.00 equiv.) were added to the reaction mixture. Then, outside the glovebox, freezing the reaction mixture under –78 °C, and using a T-connection, the Schlenk was evacuated and backfilled with N_2O (three times). The Schlenk was then closed, and the pressure of N_2O was increased to 1.5 atm. The reaction mixture was kept stirring at 1000 rpm, for 16 h, at room temperature. After the completion of reaction, the mixture was diluted with MTBE, washed with aqueous solution of HCl (1 M) and brine, and dried over Na_2SO_4. Upon filtration, the organic layer was concentrated under reduced pressure (water bath at 40 °C) and purified by preparative TLC (pTLC) (5:1 hexanes:EtOAc) to afford 9.5 mg (51%) of the desired product **58**.

Physical State: colorless oil.

R_f = 0.47 (5:1 hexanes:EtOAc)

^{1}H NMR (300 MHz, $CDCl_3$, 298 K) δ 7.35 – 7.26 (m, 2H), 7.09 – 7.00 (m, 1H), 6.98 – 6.89 (m, 4H), 6.86 – 6.77 (m, 2H), 4.72 (br s, 1H).

HRMS (EI-TOF): calc'd for $C_{12}H_{10}O_2$ $[M]^+$: 186.0675, found: 186.0677; deviation: –0.7 ppm.

Spectral data is in accordance with previous report.[19]

Compound 61

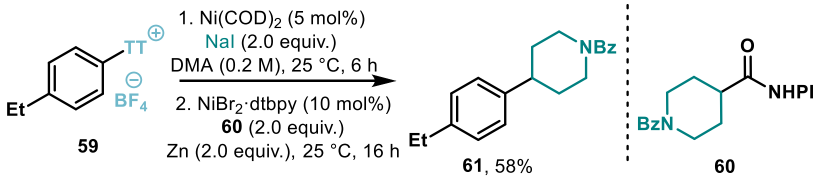

In an argon-filled glovebox, a culture tube equipped with a Teflon-coated stir bar was charged with aryl thianthrenium salt **59** (40.8 mg, 0.100 mmol, 1.00 equiv.), Ni(COD)$_2$ (1.4 mg, 0.0050 mmol, 5 mol%) and NaI (30.0 mg, 0.20 mmol, 2.0 equiv.). Then, DMA (0.5 mL) was added, and the mixture was stirred for 6 h at 25 °C. After that, NiBr$_2$·dtbpy (4.8 mg, 0.010 mmol, 10 mol%), redox-active ester **60** (75.6 mg, 0.200 mmol, 2.00 equiv.), zinc (325 mesh) (13.0 mg, 0.200 mmol, 2.00 equiv.) were added to the reaction mixture. Then, outside the glovebox, the reaction mixture was kept stirring at 1000 rpm, for 16 h, at room temperature. After the completion of reaction, the mixture was diluted with MTBE, washed with aqueous solution of HCl (1 M) and brine, and dried over Na_2SO_4. Upon filtration, the organic layer was concentrated under reduced pressure (water bath at 40 °C) and purified by preparative TLC (pTLC) (3:1 hexanes:EtOAc) to afford 17.0 mg (58%) of the desired product **61**.

Physical State: colorless oil.

R_f = 0.41 (3:1 hexanes:EtOAc)

^{1}H NMR (600 MHz, CDCl$_3$, 253 K) δ 7.49 – 7.37 (m, 5H), 7.20 – 7.12 (m, 4H), 4.89 (dt, *J* = 13.1, 2.2 Hz, 1H), 3.93 – 3.83 (m, 1H), 3.13 (td, *J* = 13.1, 2.6 Hz, 1H), 2.86 (td, *J* = 13.0, 2.8 Hz, 1H), 2.77 (ddd, *J* = 12.2, 8.6, 3.6 Hz, 1H), 2.63 (q, *J* = 7.6 Hz, 2H), 2.01 – 1.94 (m, 1H), 1.82 – 1.72 (m, 2H), 1.67 – 1.55 (m, 1H), 1.22 (t, *J* = 7.6 Hz, 3H).

^{13}C NMR (151 MHz, CDCl$_3$, 253 K) δ 170.5, 142.6, 142.4, 136.0, 129.7, 128.6, 128.1, 126.9, 126.8, 48.5, 42.8, 42.3, 33.9, 33.1, 28.5, 15.9.

HRMS (EI-TOF): calc'd for $C_{20}H_{23}N_1O_1$ $[M]^+$: 293.1774, found: 293.1777; deviation: –0.9 ppm.

13 References

[1] a) Berger, F.; Plutschack, M. B.; Riegger, J.; Yu, W.; Speicher, S.; Ho, M.; Frank, N.; Ritter, T., Site-selective and versatile aromatic C−H functionalization by thianthrenation. *Nature* **2019,** *567*, 223-228; b) Engl, P. S.; Häring, A. P.; Berger, F.; Berger, G.; Pérez-Bitrián, A.; Ritter, T., C–N Cross-Couplings for Site-Selective Late-Stage Diversification via Aryl Sulfonium Salts. *J. Am. Chem. Soc.* **2019,** *141*, 13346-13351; c) Sang, R.; Korkis, S. E.; Su, W.; Ye, F.; Engl, P. S.; Berger, F.; Ritter, T., Site-Selective C−H Oxygenation via Aryl Sulfonium Salts. *Angew. Chem., Int. Ed.* **2019,** *58*, 16161-16166; d) Ye, F.; Berger, F.; Jia, H.; Ford, J.; Wortman, A.; Börgel, J.; Genicot, C.; Ritter, T., Aryl Sulfonium Salts for Site-Selective Late-Stage Trifluoromethylation. *Angew. Chem., Int. Ed.* **2019,** *58*, 14615-14619; e) Li, J.; Chen, J.; Sang, R.; Ham, W.-S.; Plutschack, M. B.; Berger, F.; Chabbra, S.; Schnegg, A.; Genicot, C.; Ritter, T., Photoredox catalysis with aryl sulfonium salts enables site-selective late-stage fluorination. *Nat. Chem.* **2020,** *12*, 56-62; f) Lansbergen, B.; Granatino, P.; Ritter, T., Site-Selective C–H alkylation of Complex Arenes by a Two-Step Aryl Thianthrenation-Reductive Alkylation Sequence. *J. Am. Chem. Soc.* **2021,** *143*, 7909-7914.

[2] Schwab, M. M.; Himmel, D.; Kacprzak, S.; Kratzert, D.; Radtke, V.; Weis, P.; Ray, K.; Scheidt, E.-W.; Scherer, W.; de Bruin, B.; Weber, S.; Krossing, I., [Ni(cod)$_2$][Al(ORF)$_4$], a Source for Naked Nickel(I) Chemistry. *Angew. Chem., Int. Ed.* **2015**, *54*, 14706-14709.

[3] Engl, P. S.; Häring, A. P.; Berger, F.; Berger, G.; Pérez-Bitrián, A.; Ritter, T., C–N Cross-Couplings for Site-Selective Late-Stage Diversification via Aryl Sulfonium Salts. *J. Am. Chem. Soc.* **2019,** *141*, 13346-13351.

[4] Sousa, S. C. A.; Fernandes, T. A.; Fernandes, A. C., Highly Efficient Deoxygenation of Aryl Ketones to Arylalkanes Catalyzed by Dioxidomolybdenum Complexes. *Eur. J. Org. Chem.* **2016,** *18*, 3109-3112.

[5] Nagarjun, N.; Arthy, K.; Dhakshinamoorthy, A., Copper (II)-Doped ZIF-8 as a Reusable and Size Selective Heterogeneous Catalyst for the Hydrogenation of Alkenes using Hydrazine Hydrate. *Eur. J. Inorg.Chem.* **2021**, *22*, 2108-2119.

[6] Han, B.; Zhang, M.; Jiao, H.; Ma, H.; Wang, J.; Zhang, Y. J. R. a., Ligand-enabled and magnesium-activated hydrogenation with earth-abundant cobalt catalysts. *RSC Adv.* **2021,** *11*, 39934-39939.

[7] Cella, R.; Cunha, R. L. O. R.; Reis, A. E. S.; Pimenta, D. C.; Klitzke, C. F.; Stefani, H. A., Suzuki−Miyaura Cross-Coupling Reactions of Aryl Tellurides with Potassium Aryltrifluoroborate Salts. *J. Org. Chem.* **2006**, *71*, 244-250.

[8] Gieuw, M. H.; Ke, Z.; Yeung, Y.-Y., Lewis Base-Promoted Ring-Opening 1,3-Dioxygenation of Unactivated Cyclopropanes Using a Hypervalent Iodine Reagent. *Angew. Chem., Int. Ed.* **2018,** *57*, 3782-3786.

[9] Leas, D. A.; Dong, Y.; Vennerstrom, J. L.; Stack, D. E., One-Pot, Metal-Free Conversion of Anilines to Aryl Bromides and Iodides. *Org. Lett.* **2017,** *19*, 2518-2521.

[10] Chan, L.; McNally, A.; Toh, Q. Y.; Mendoza, A.; Gaunt, M. J., A counteranion triggered arylation strategy using diaryliodonium fluorides. *Chem. Sci.* **2015,** *6*, 1277-1281.

[11] Düsel, S. J. S.; König, B., Oxidative Photochlorination of Electron-Rich Arenes via in situ Bromination. *Eur. J. Org. Chem.* **2020,** *10*, 1491-1495.

[12] Narobe, R.; Düsel, S. J. S.; Iskra, J.; König, B., Photocatalytic Oxidative Iodination of Electron-Rich Arenes. *Adv. Synth.Catal.* **2019,** *361*, 3998-4004.

[13] Barluenga, J.; Álvarez-Gutiérrez, J. M.; Ballesteros, A.; González, J. M., Direct ortho Iodination of β- and γ-Aryl Alkylamine Derivatives. *Angew. Chem., Int. Ed.* **2007,** *46*, 1281-1283.

[14] Tao, C.-Z.; Li, J.; Fu, Y.; Liu, L.; Guo, Q.-X., Copper-catalyzed synthesis of primary arylamines from aryl halides and 2,2,2-trifluoroacetamide. *Tetrahedron Lett.* **2008,** *49*, 70-75.

[15] Salazar, J.; López, S. E.; Rebollo, O., Direct microwave promoted trifluoroacetylation of aromatic amines with trifluoroacetic acid. *J. Fluor. Chem.* **2003,** *12*, 111-113.

[16] Kulbitski, K.; Nisnevich, G.; Gandelman, M., Metal-Free Efficient, General and Facile Iododecarboxylation Method with Biodegradable Co-Products. *Adv. Synth. Catal.* **2011,** *353*, 1438-1442.

[17] Skotnitzki, J.; Kremsmair, A.; Keefer, D.; Gong, Y.; de Vivie-Riedle, R.; Knochel, P., Stereoselective Csp^3–Csp^2 Cross-Couplings of Chiral Secondary Alkylzinc Reagents with Alkenyl and Aryl Halides. *Angew. Chem., Int. Ed.* **2020,** *59*, 320-324.

[18] Van Lommel, R.; Bock, J.; Daniliuc, C. G.; Hennecke, U.; De Proft, F., A dynamic picture of the halolactonization reaction through a combination of *ab initio* metadynamics and experimental investigations. *Chem. Sci.* **2021,** *12*, 7746-7757.

[19] Shigeno, M.; Hayashi, K.; Korenaga, T.; Nozawa-Kumada, K.; Kondo, Y., Organic superbase *t*-Bu-P4-catalyzed demethylations of methoxyarenes. *Org. Chem. Front.*, **2022,** *9*, 3656-3663.

14 NMR Spectra

Compound 2 ^{1}H NMR in $CDCl_3$, 298 K

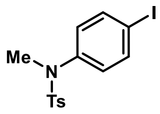

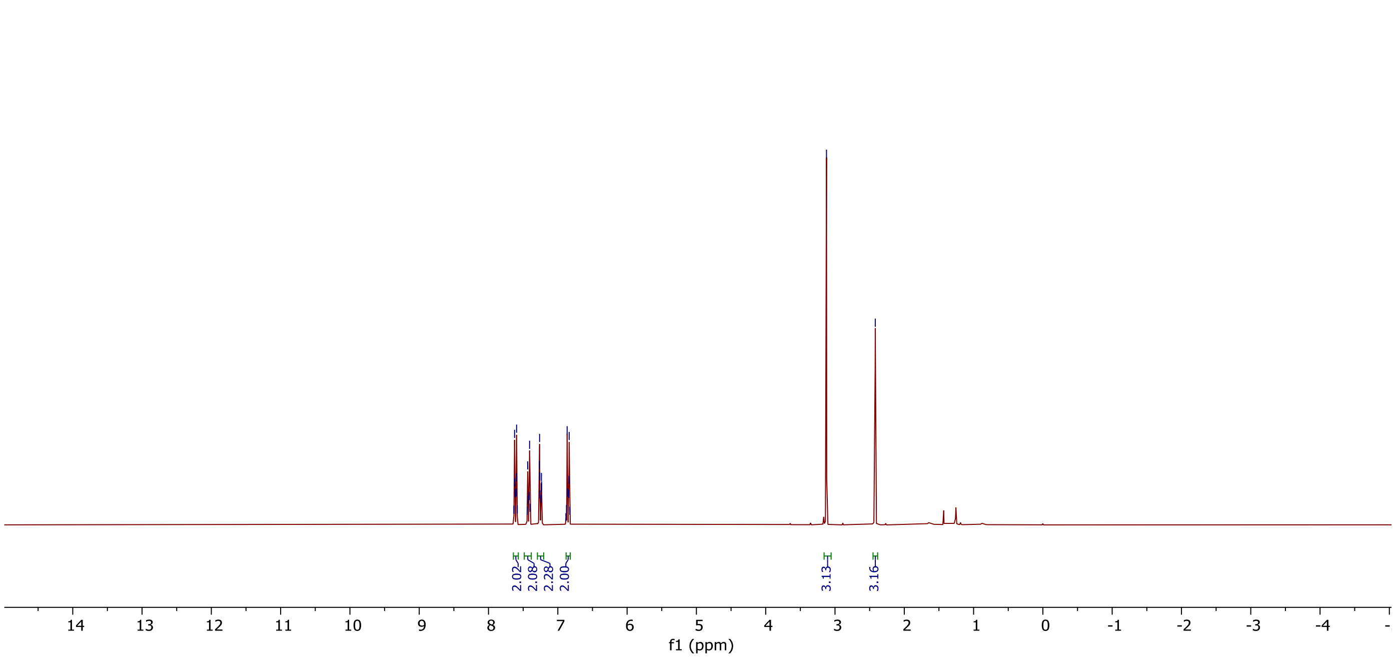

Compound 2 ^{13}C NMR in $CDCl_3$, 298 K

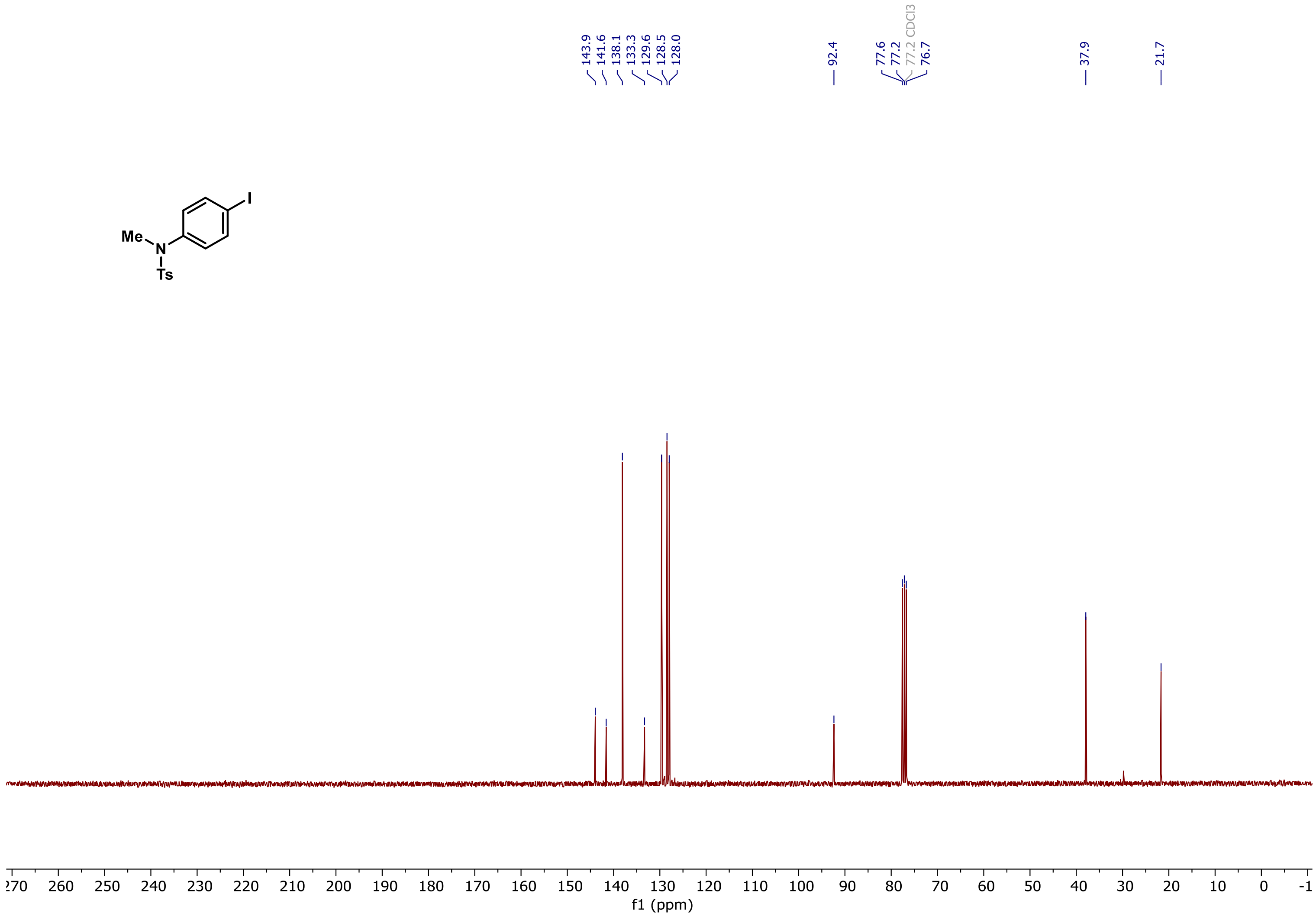

Compound 3 ^{1}H NMR in $CDCl_3$, 298 K

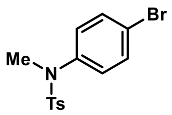

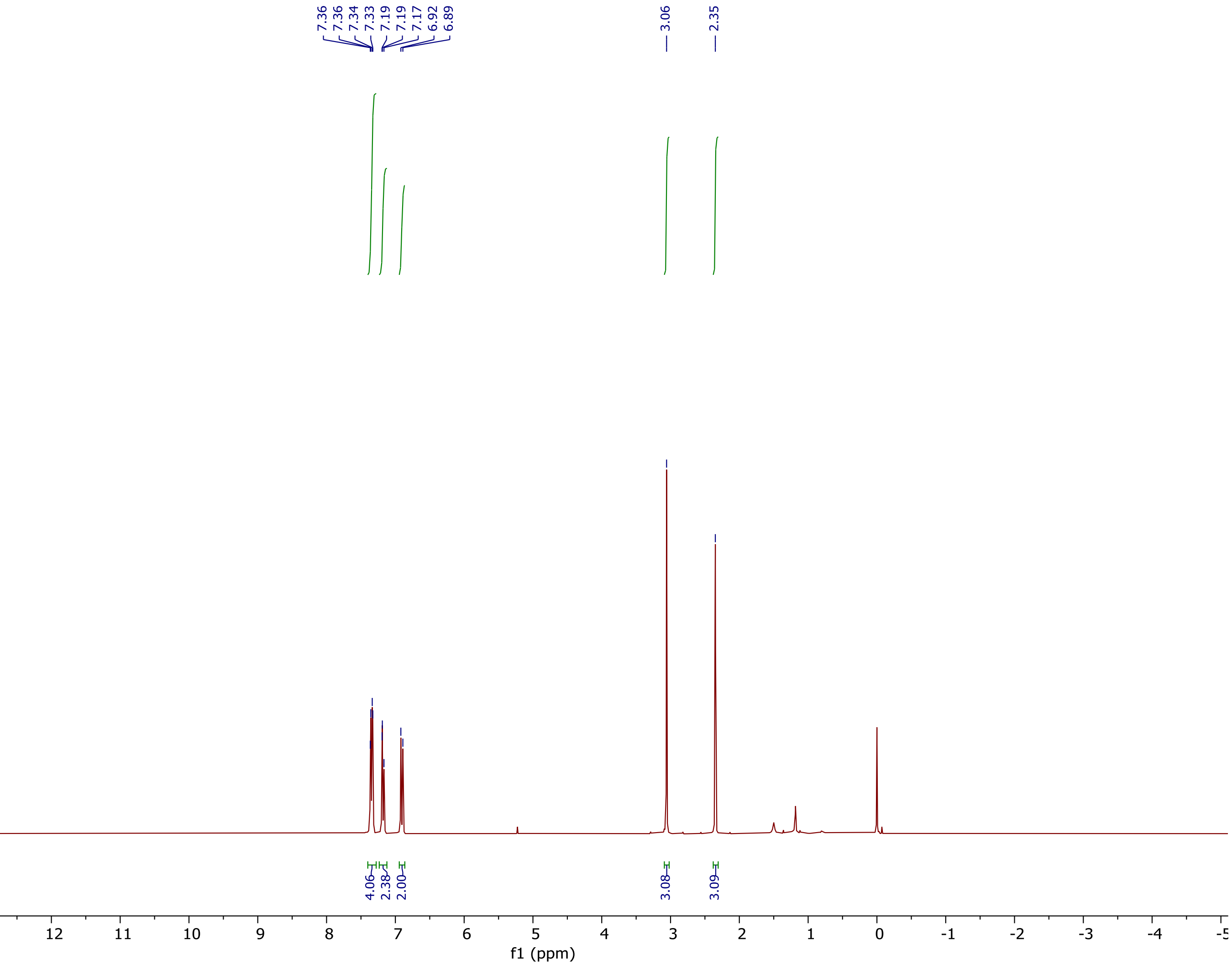

Compound 3 ^{13}C NMR in $CDCl_3$, 298 K

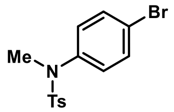

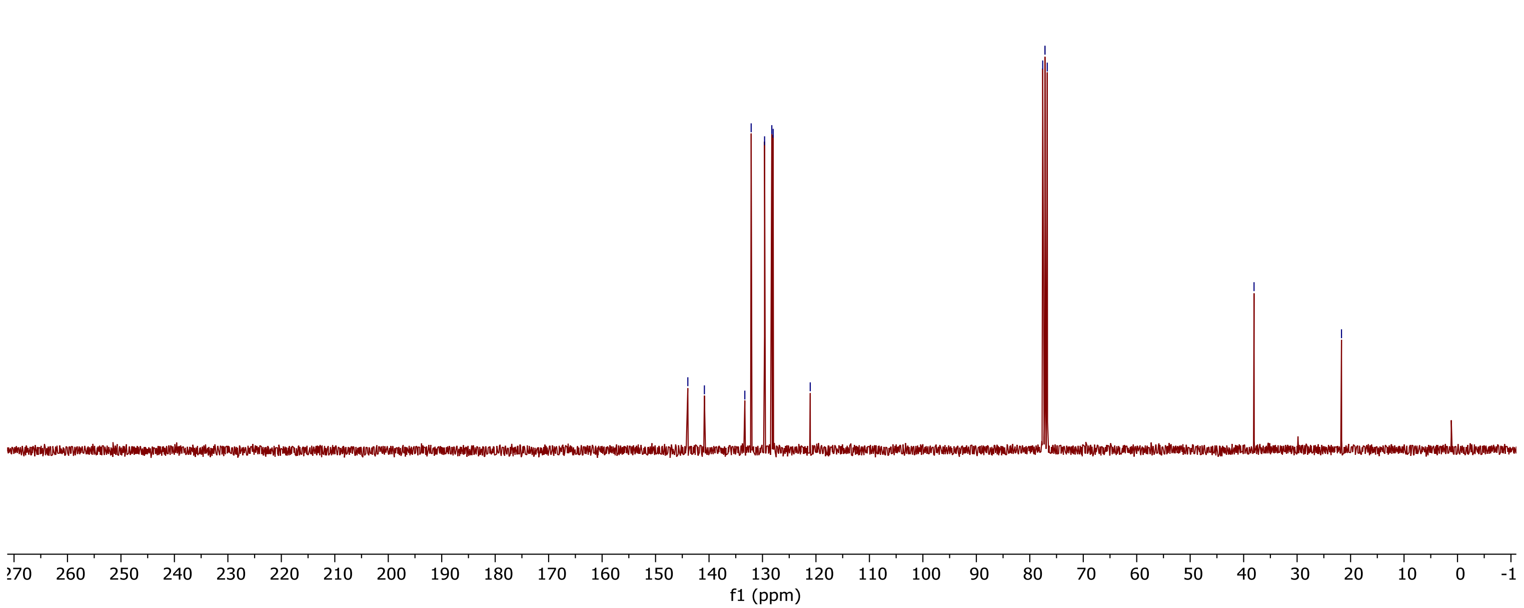

Compound 4 ^{1}H NMR in $CDCl_3$, 298 K

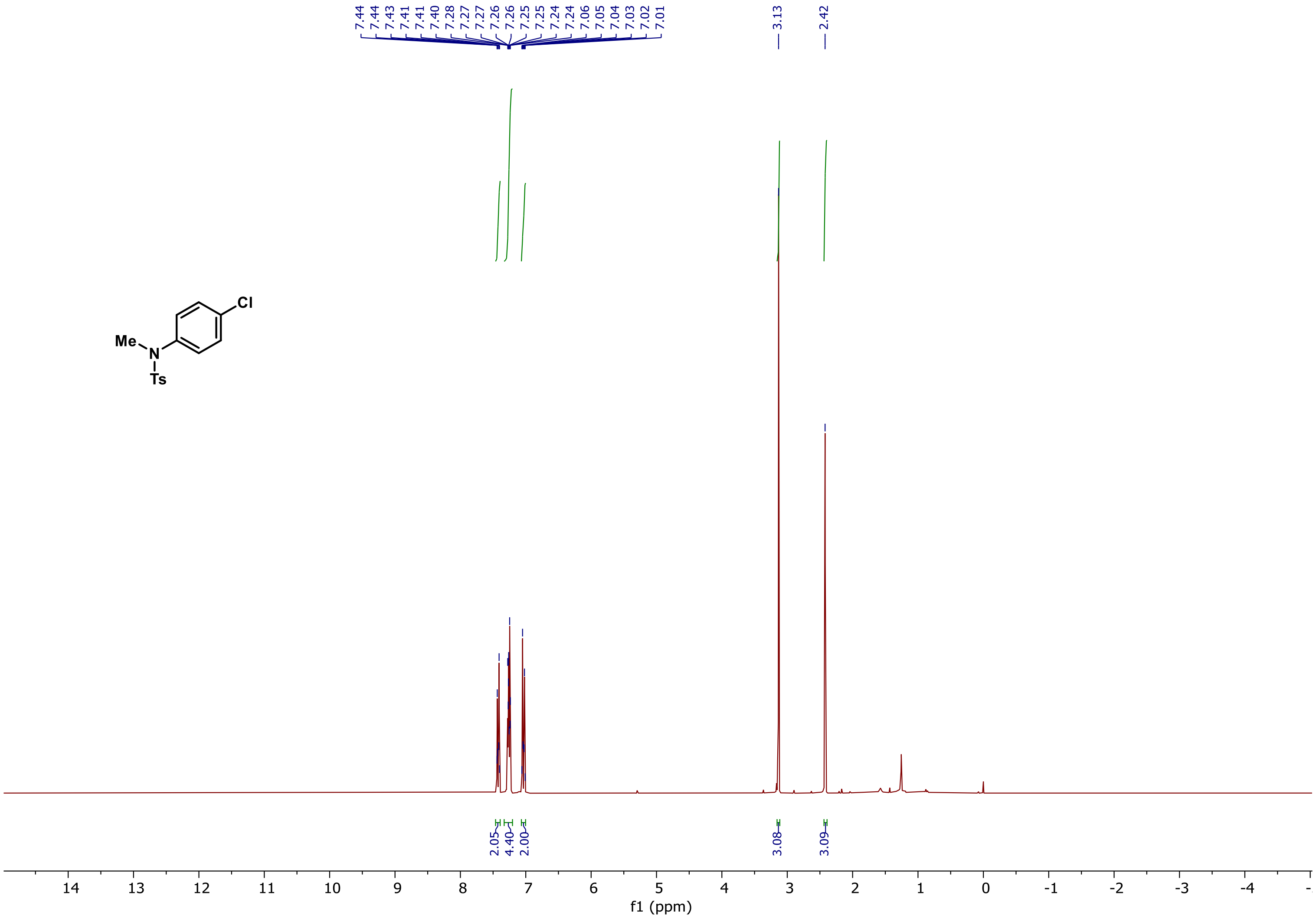

Compound 4 ^{13}C NMR in $CDCl_3$, 298 K

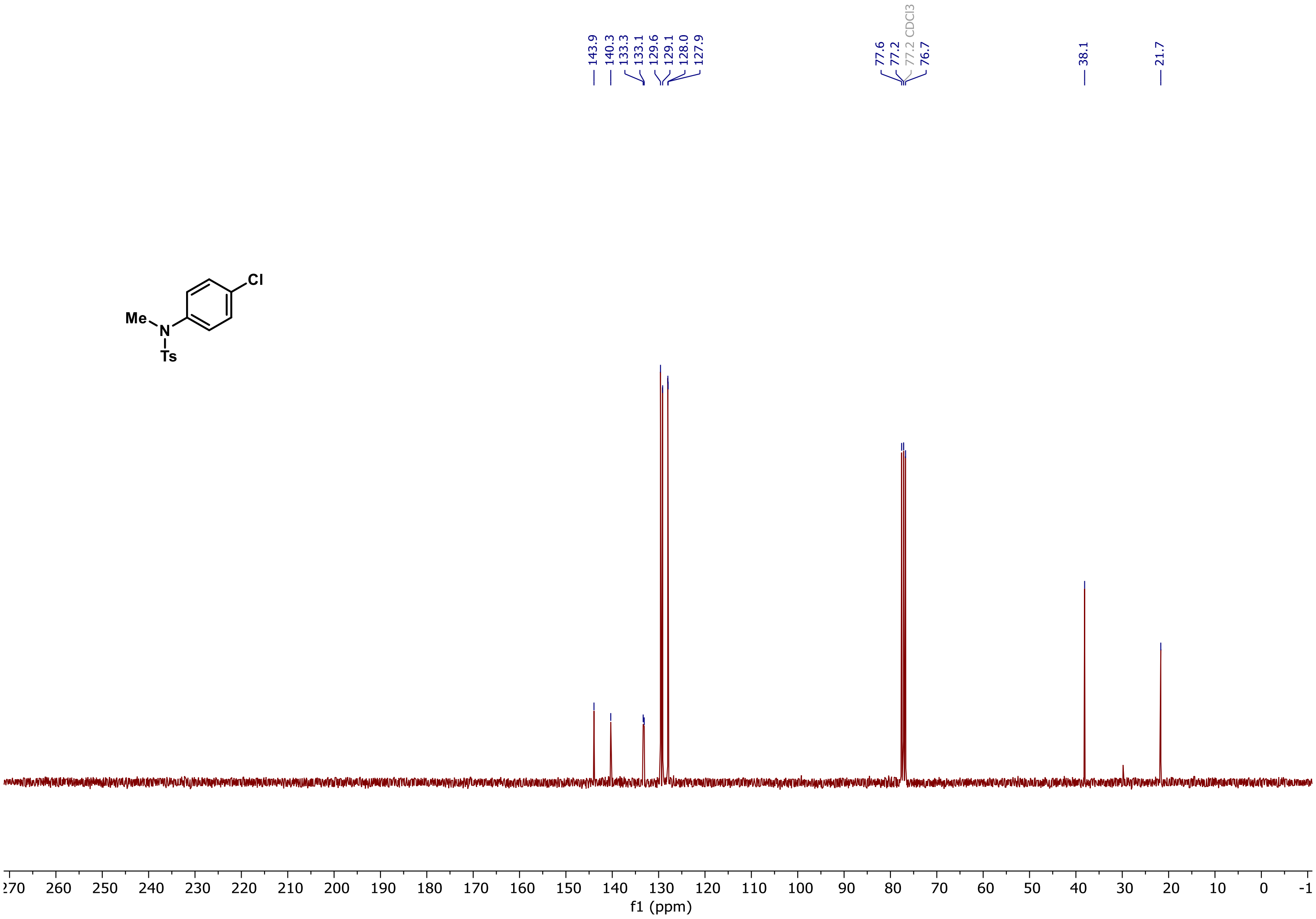

Compound 8 ^{1}H NMR in $CDCl_3$, 298 K

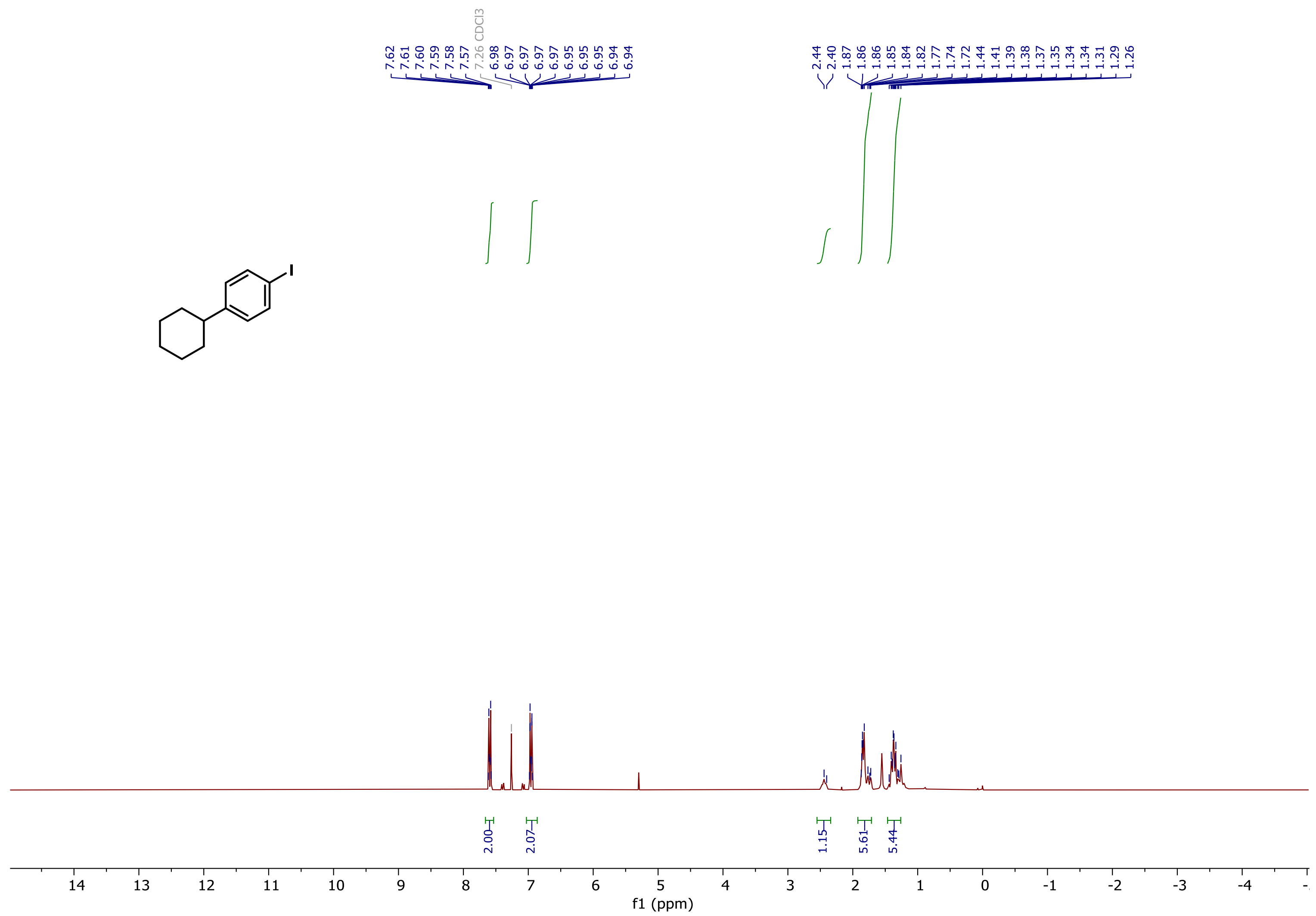

Compound 8 ^{13}C NMR in $CDCl_3$, 298 K

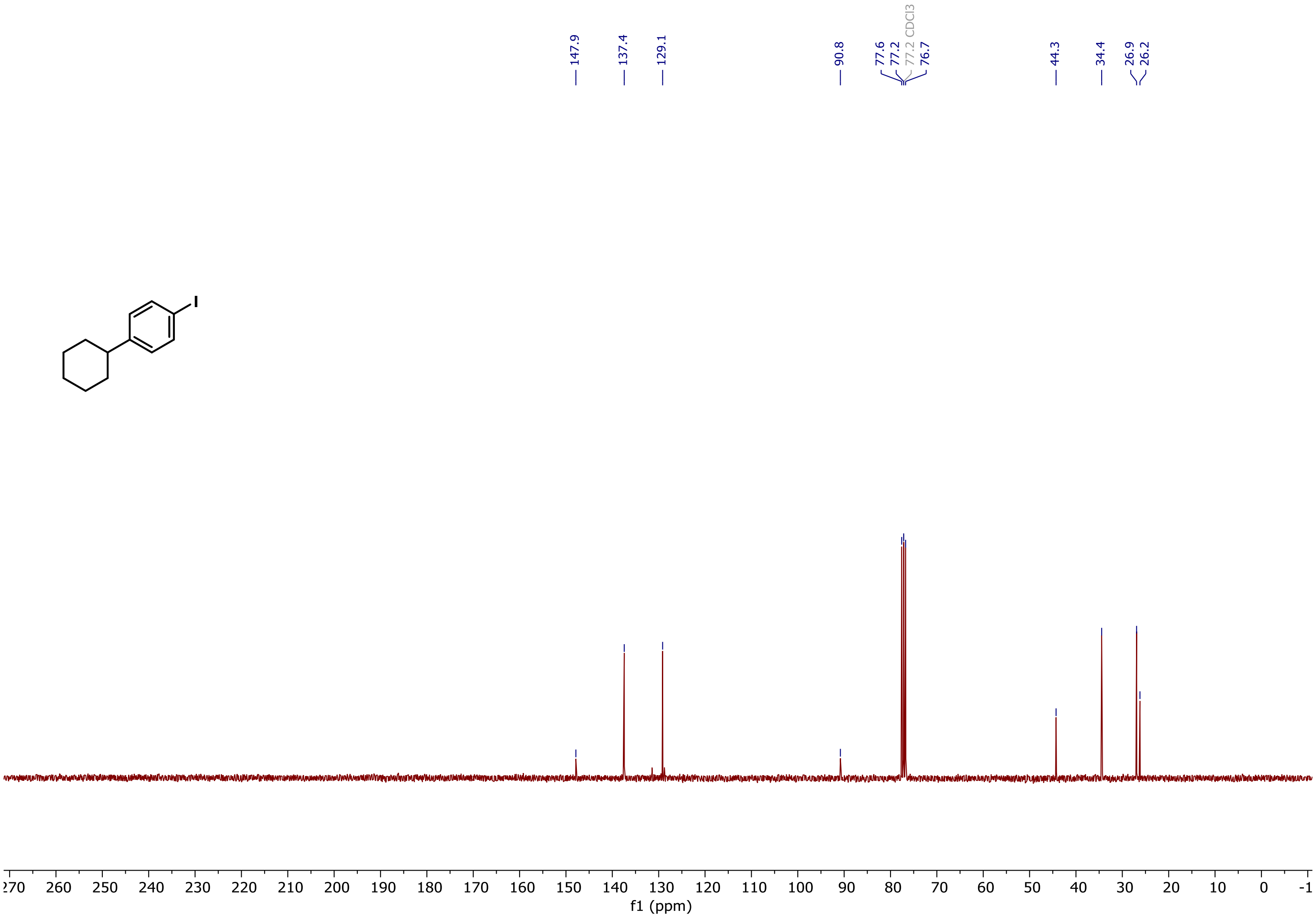

Compound 9 ^{1}H NMR in $CDCl_3$, 298 K

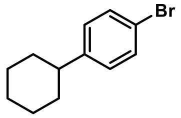

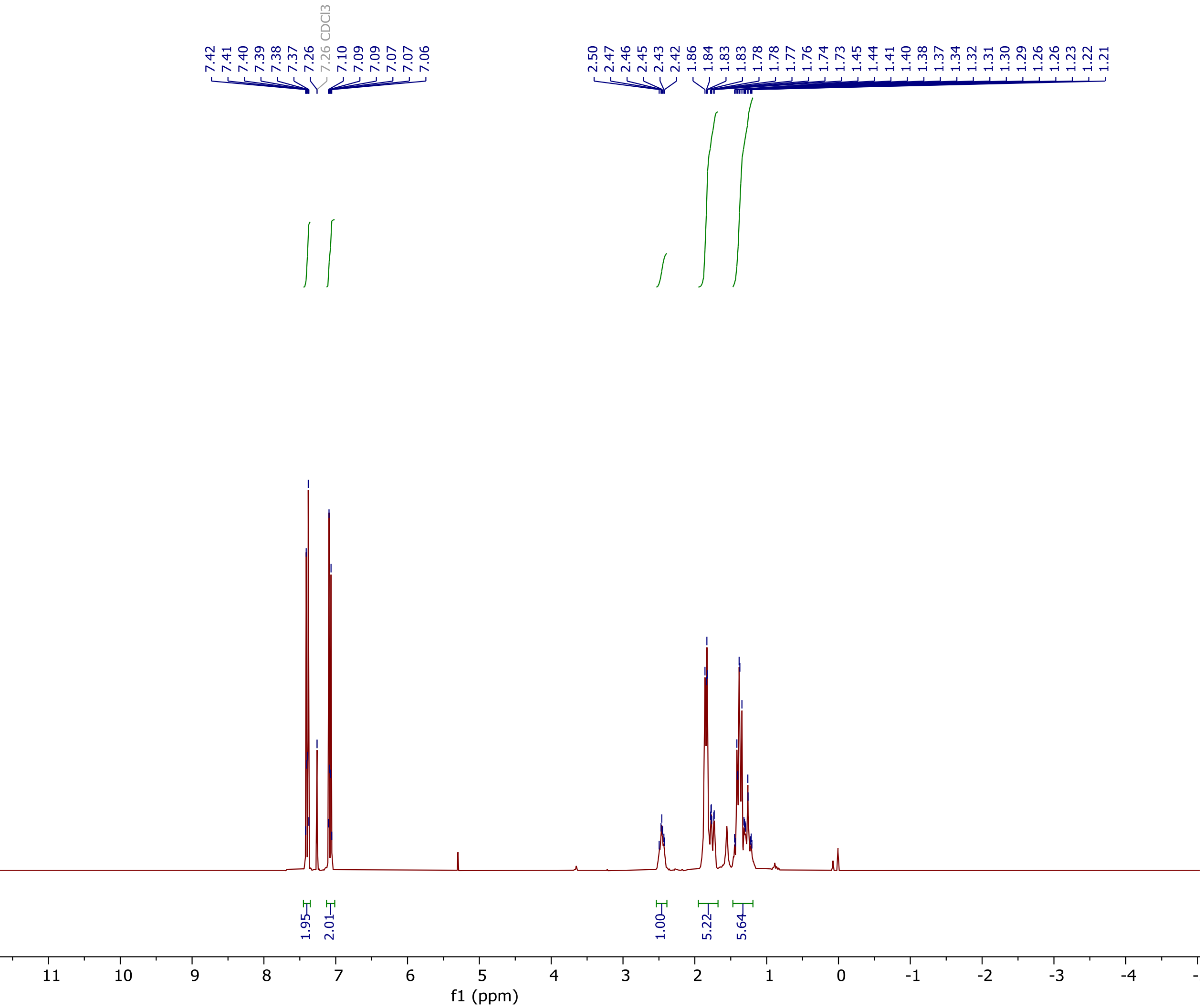

Compound 9 ^{13}C NMR in $CDCl_3$, 298 K

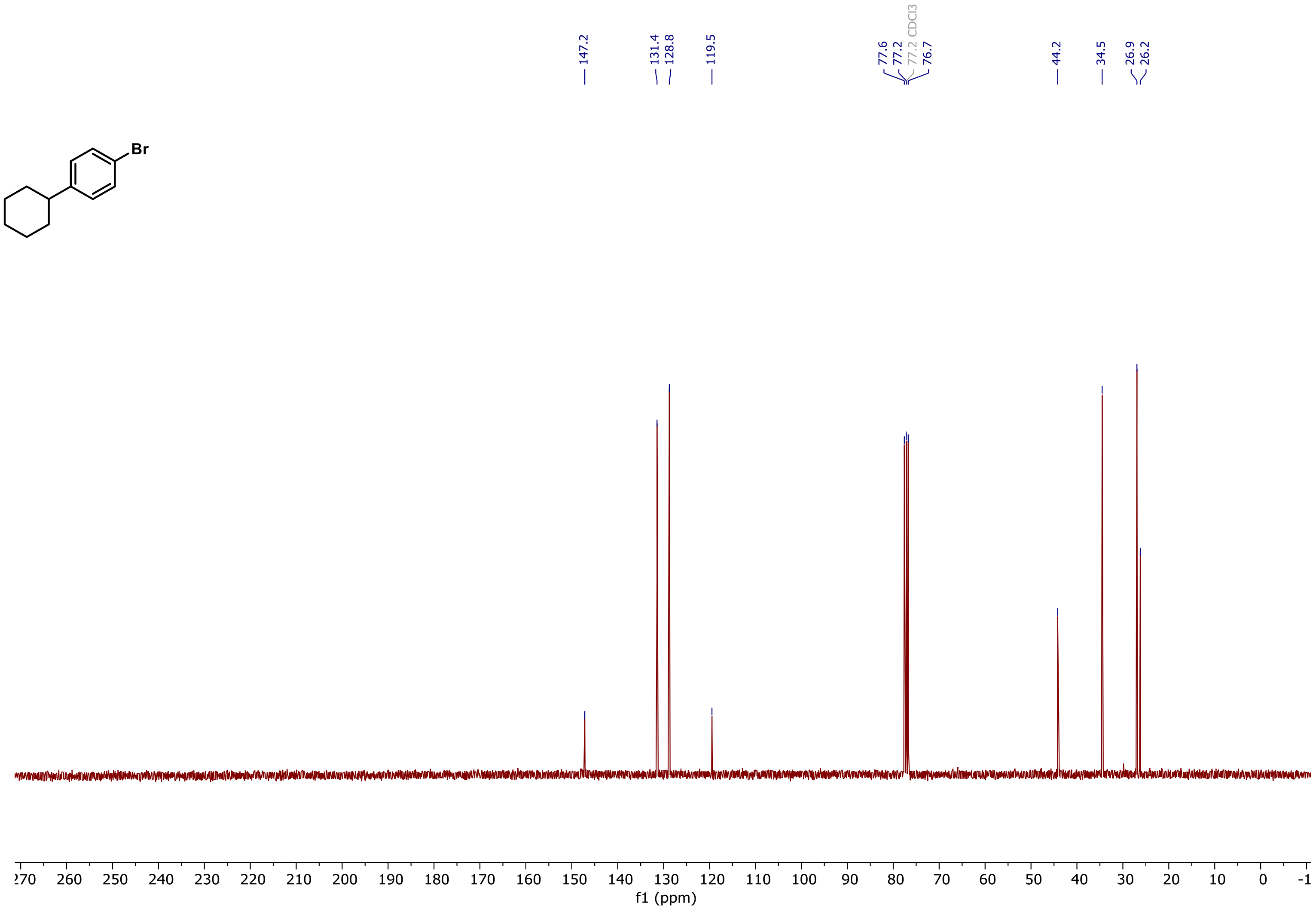

Compound 10 ^{1}H NMR in $CDCl_3$, 298 K

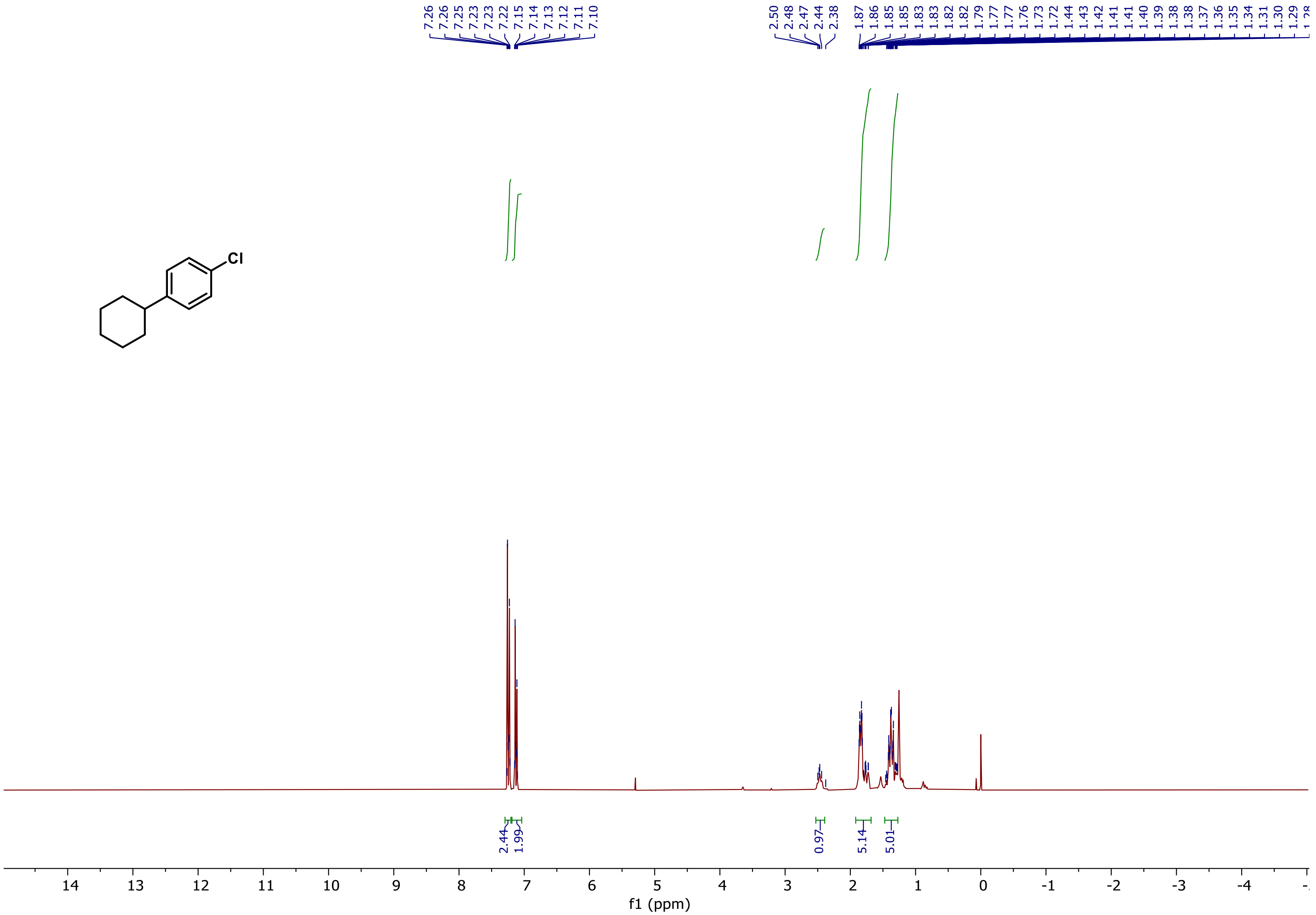

Compound 10 ^{13}C NMR in $CDCl_3$, 298 K

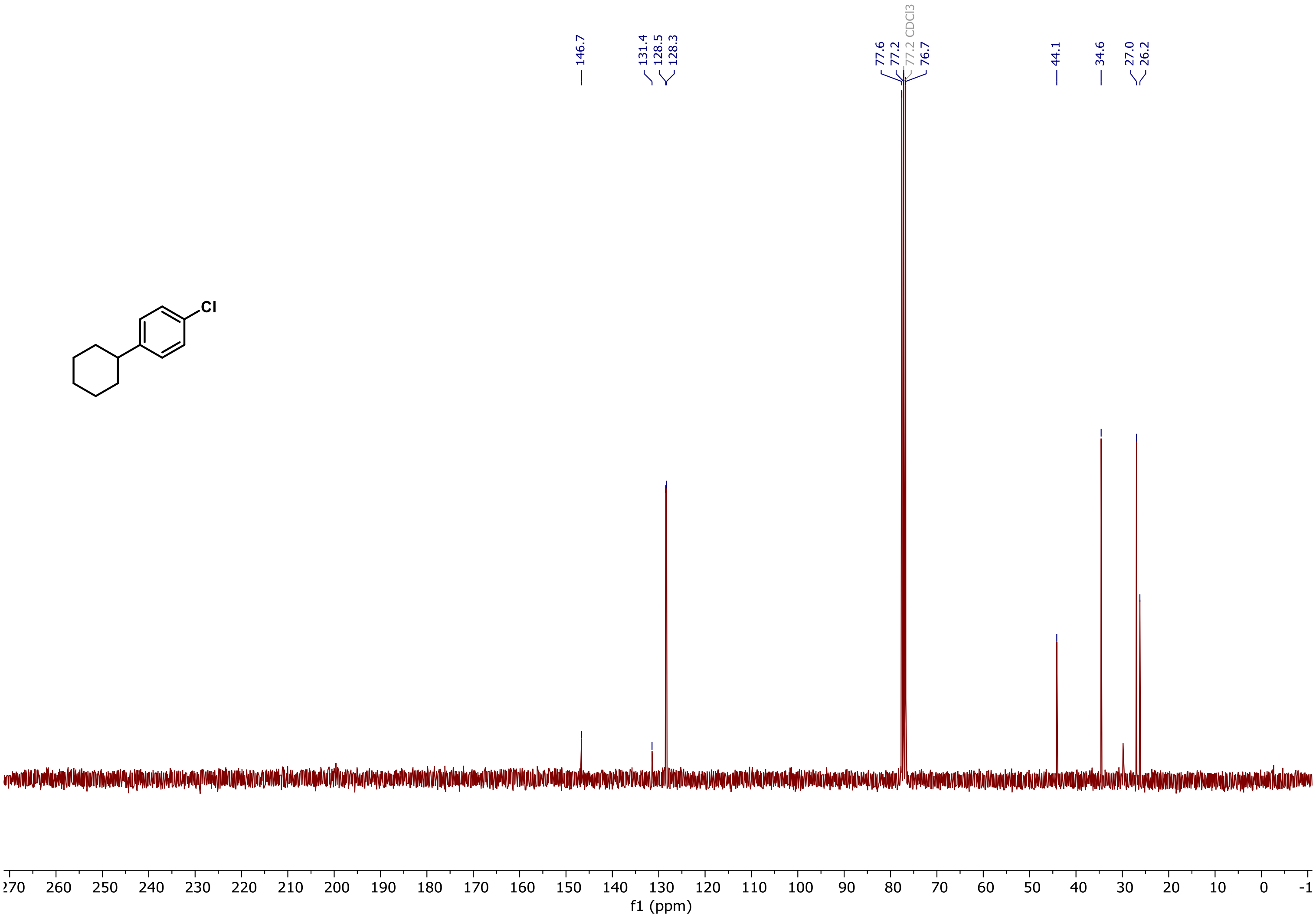

Compound 11 ^{1}H NMR in $CDCl_3$, 298 K

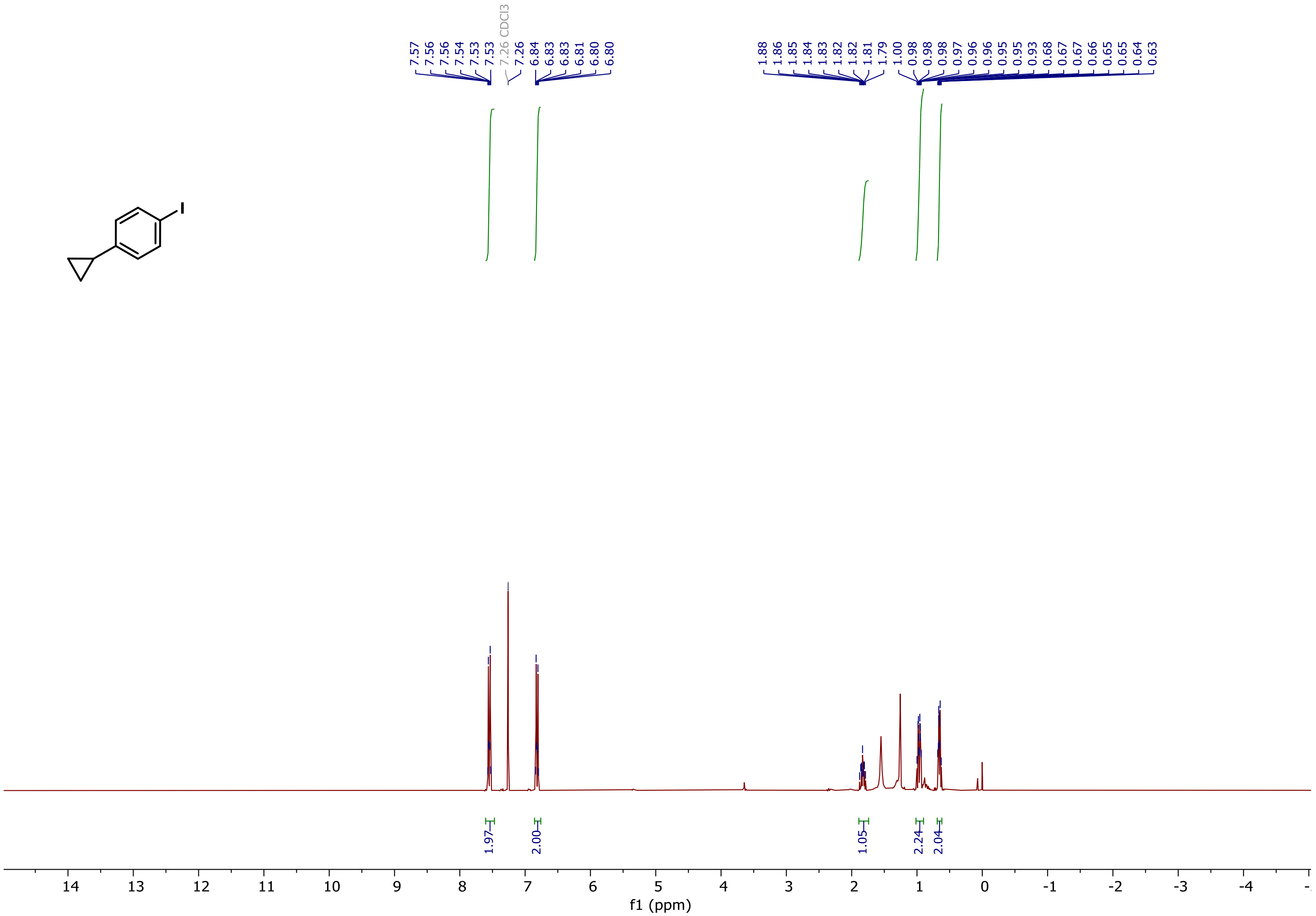

Compound 11 ^{13}C NMR in $CDCl_3$, 298 K

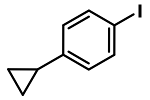

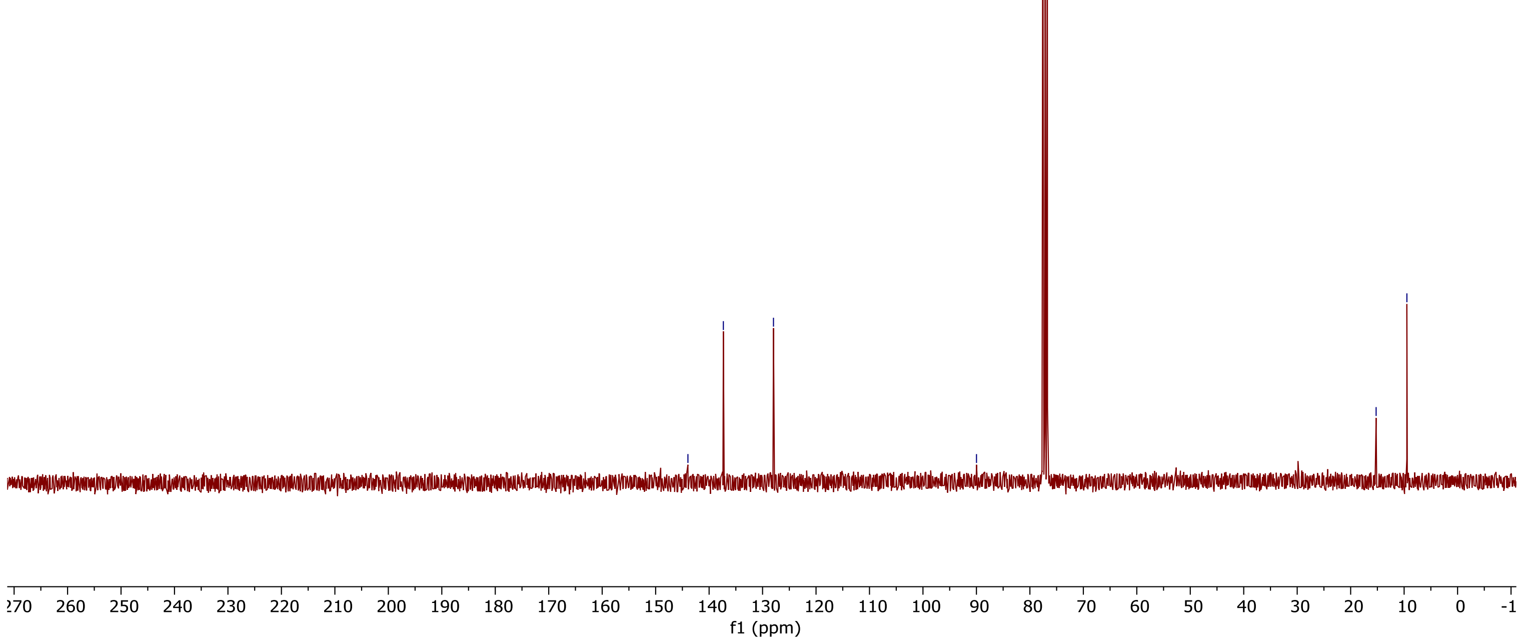

Compound 14 ^{1}H NMR in $CDCl_3$, 298 K

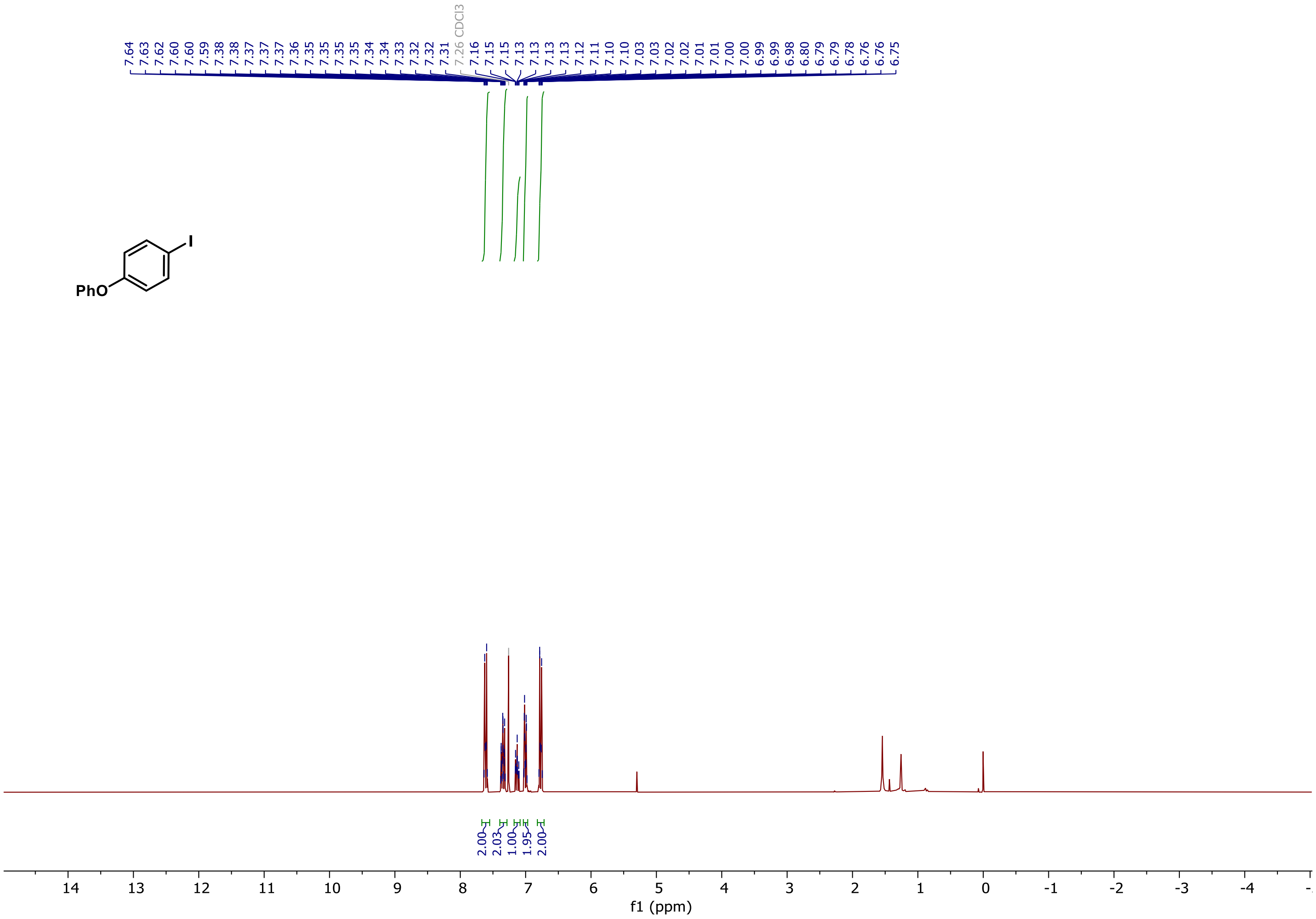

Compound 14 ^{13}C NMR in $CDCl_3$, 298 K

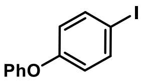

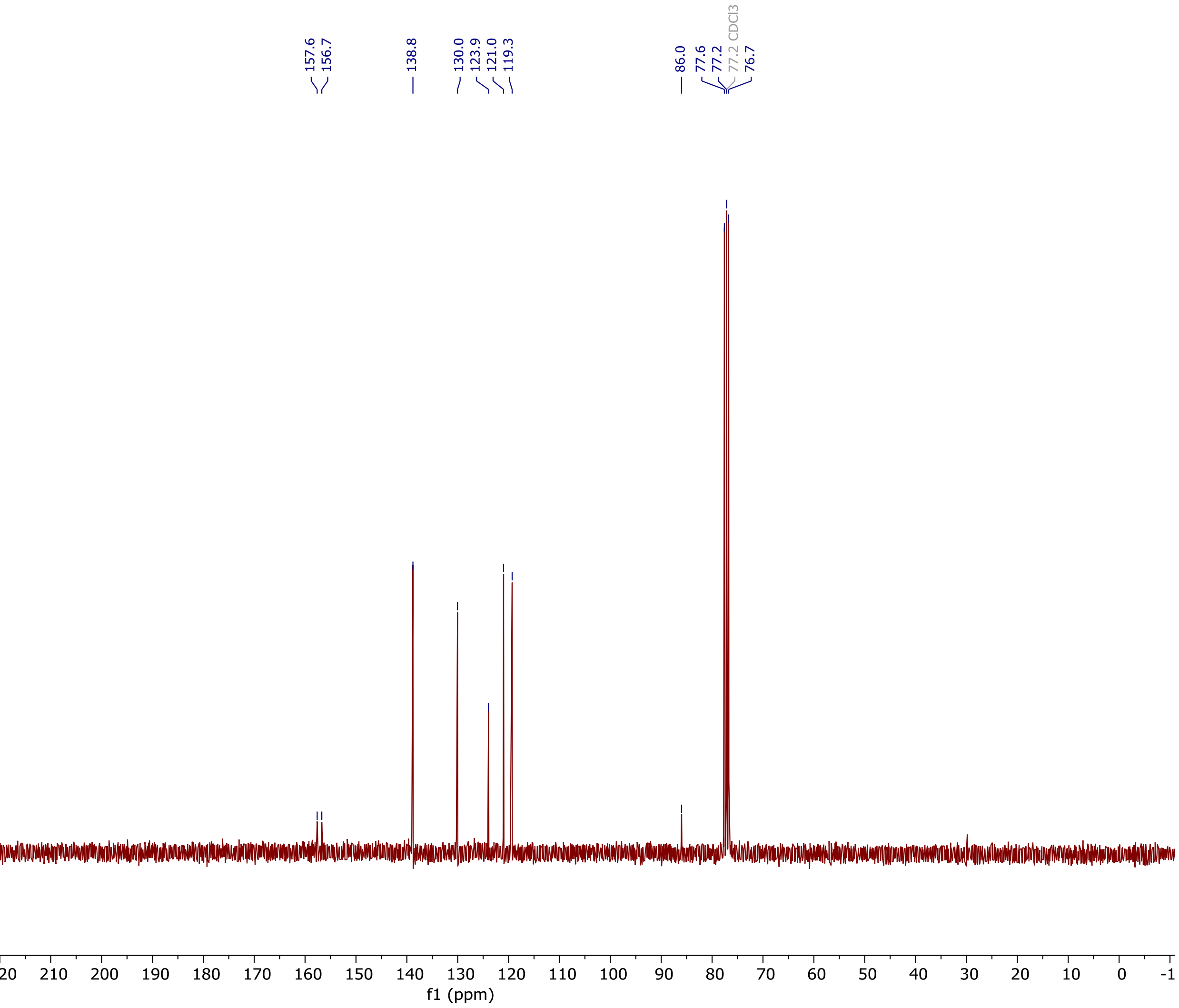

Compound 16 ^{1}H NMR in $CDCl_3$, 298 K

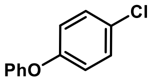

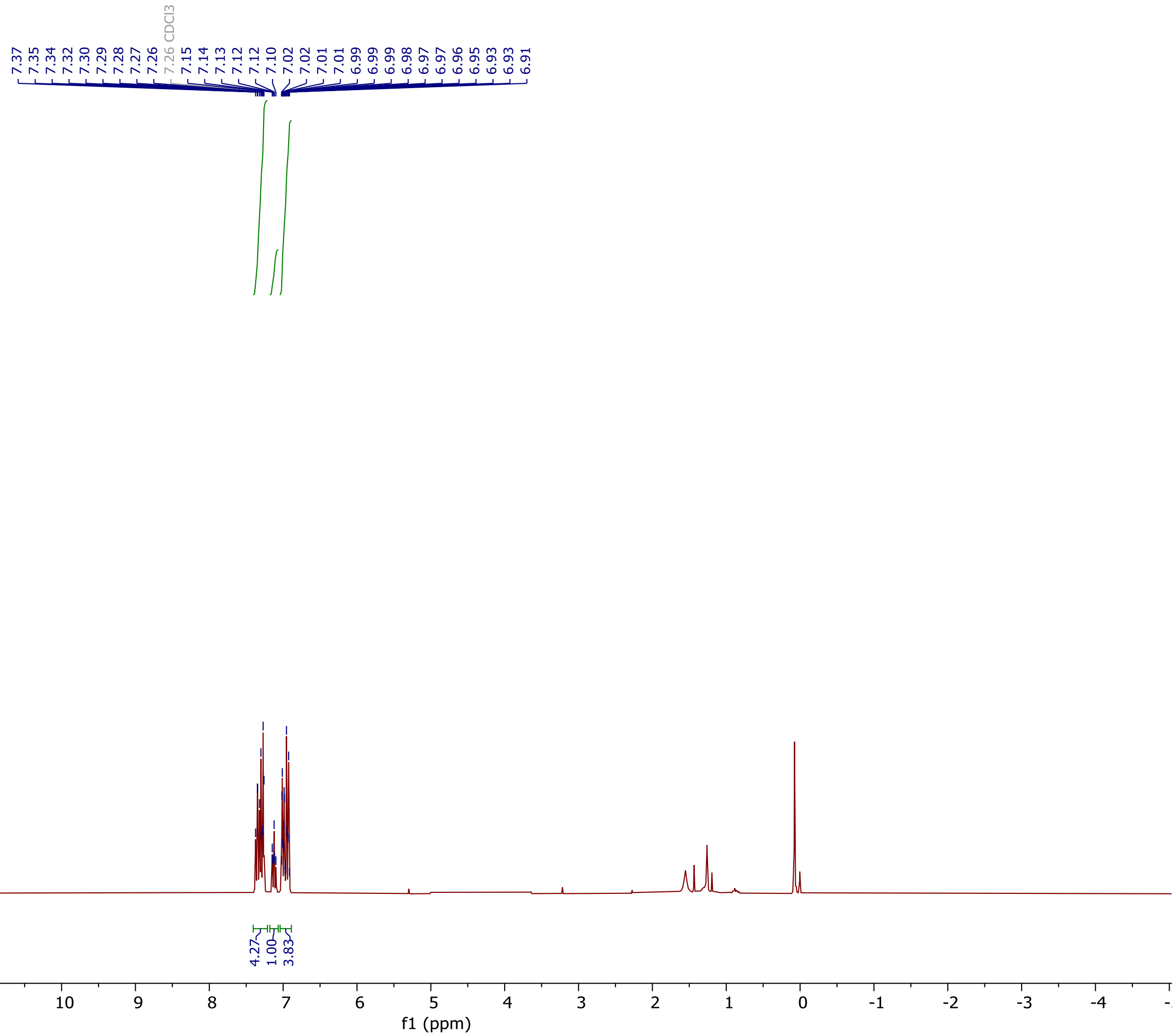

Compound 16 ^{13}C NMR in $CDCl_3$, 298 K

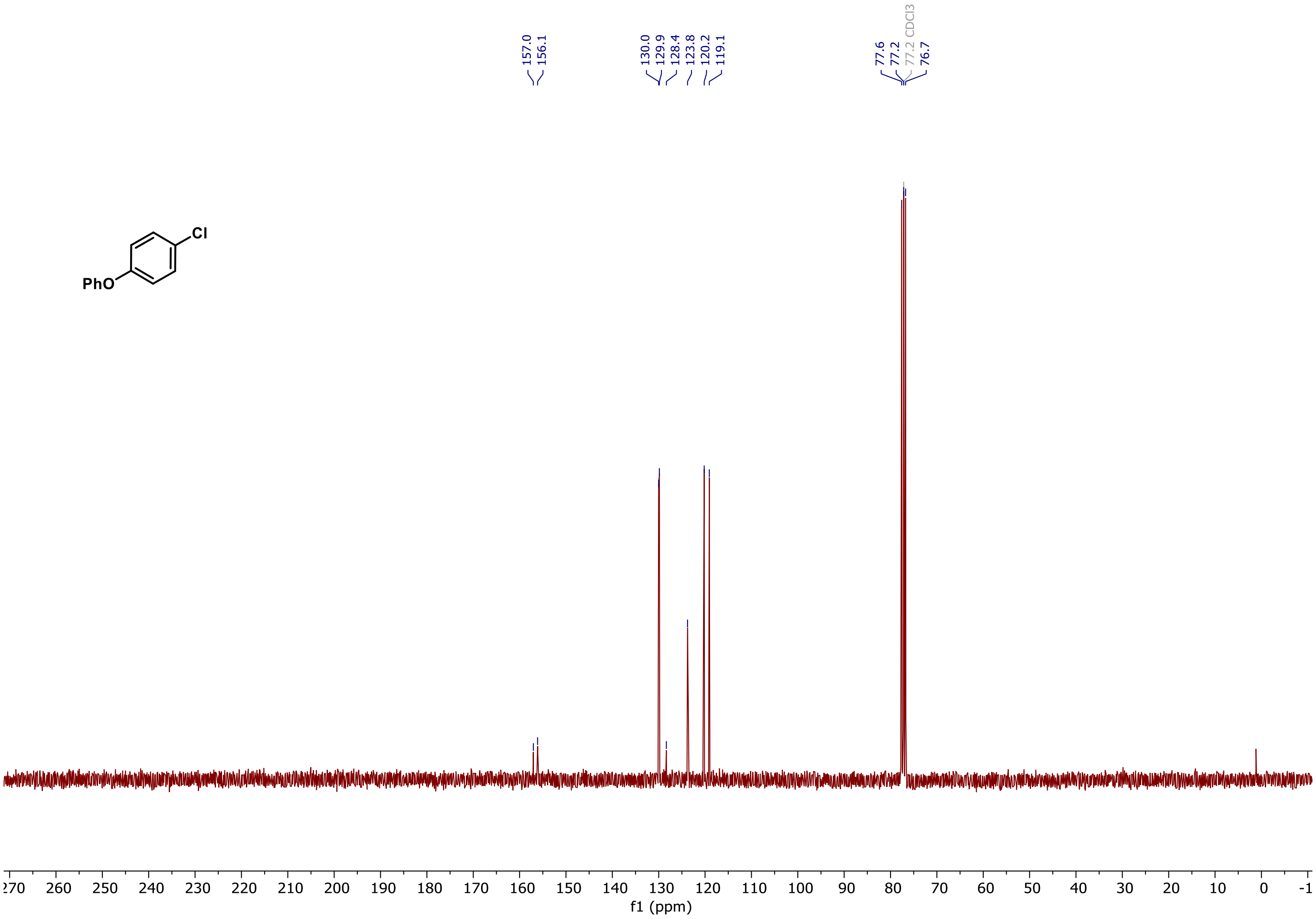

Compound 17 ^{1}H NMR in $CDCl_3$, 298 K

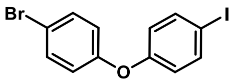

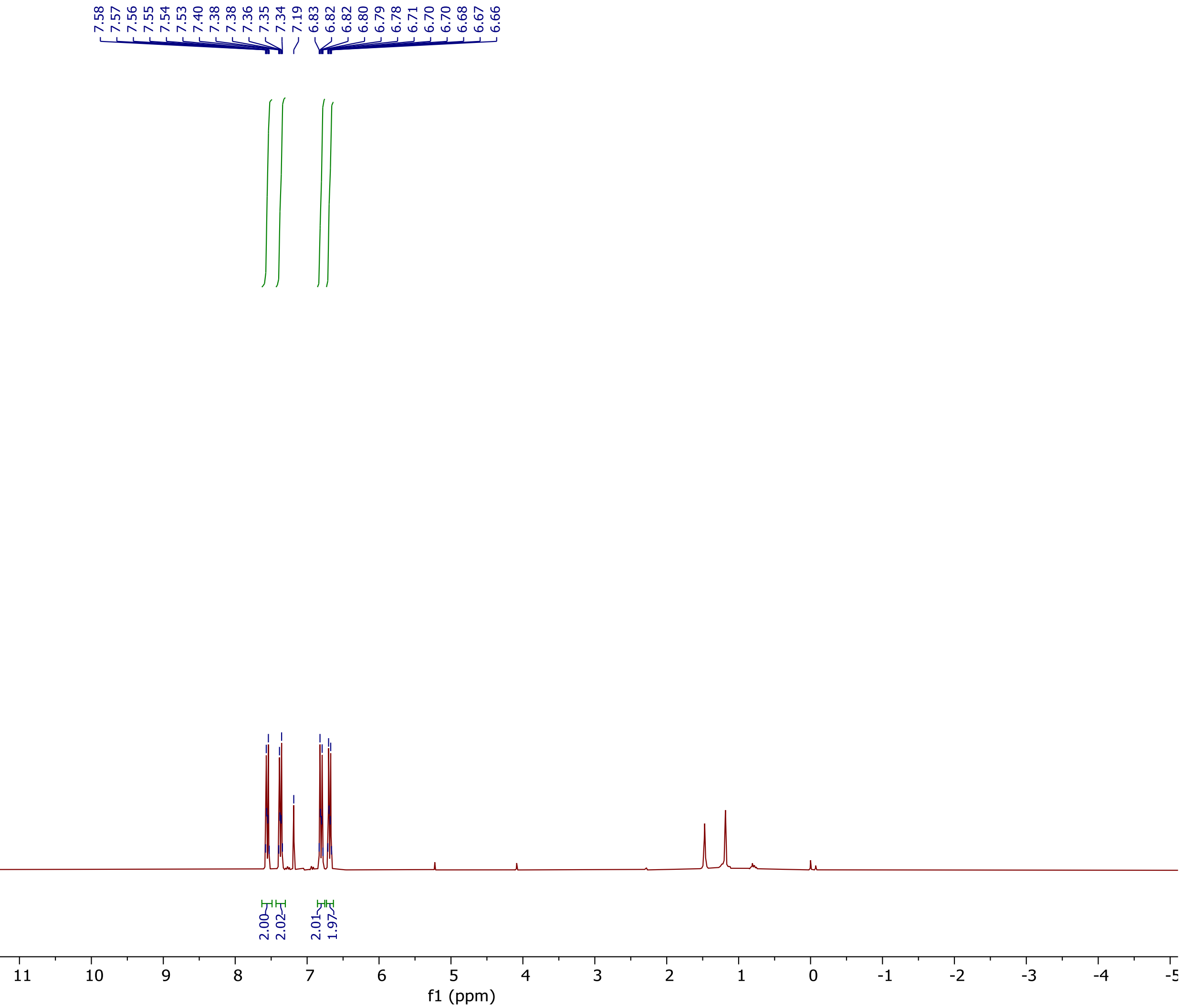

Compound 17 ^{13}C NMR in $CDCl_3$, 298 K

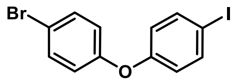

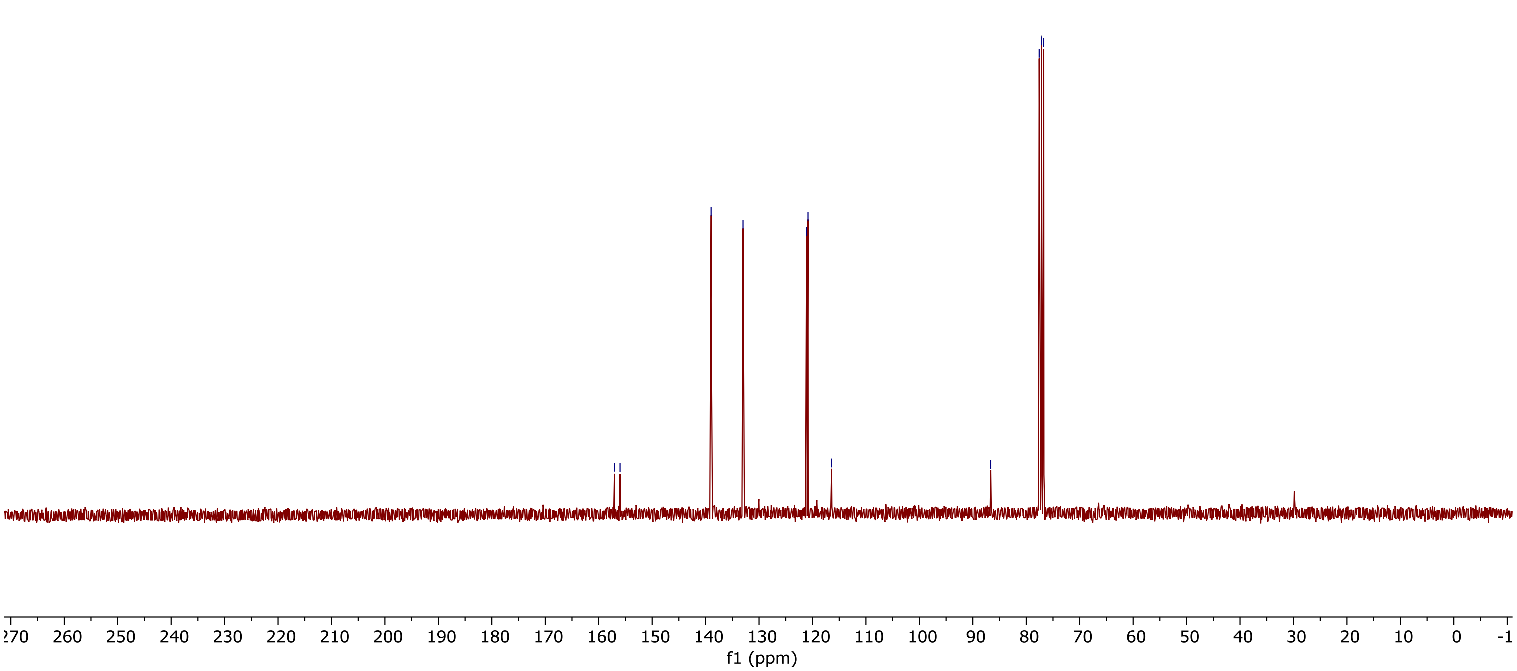

Compound 18 ^{1}H NMR in $CDCl_3$, 298 K

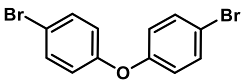

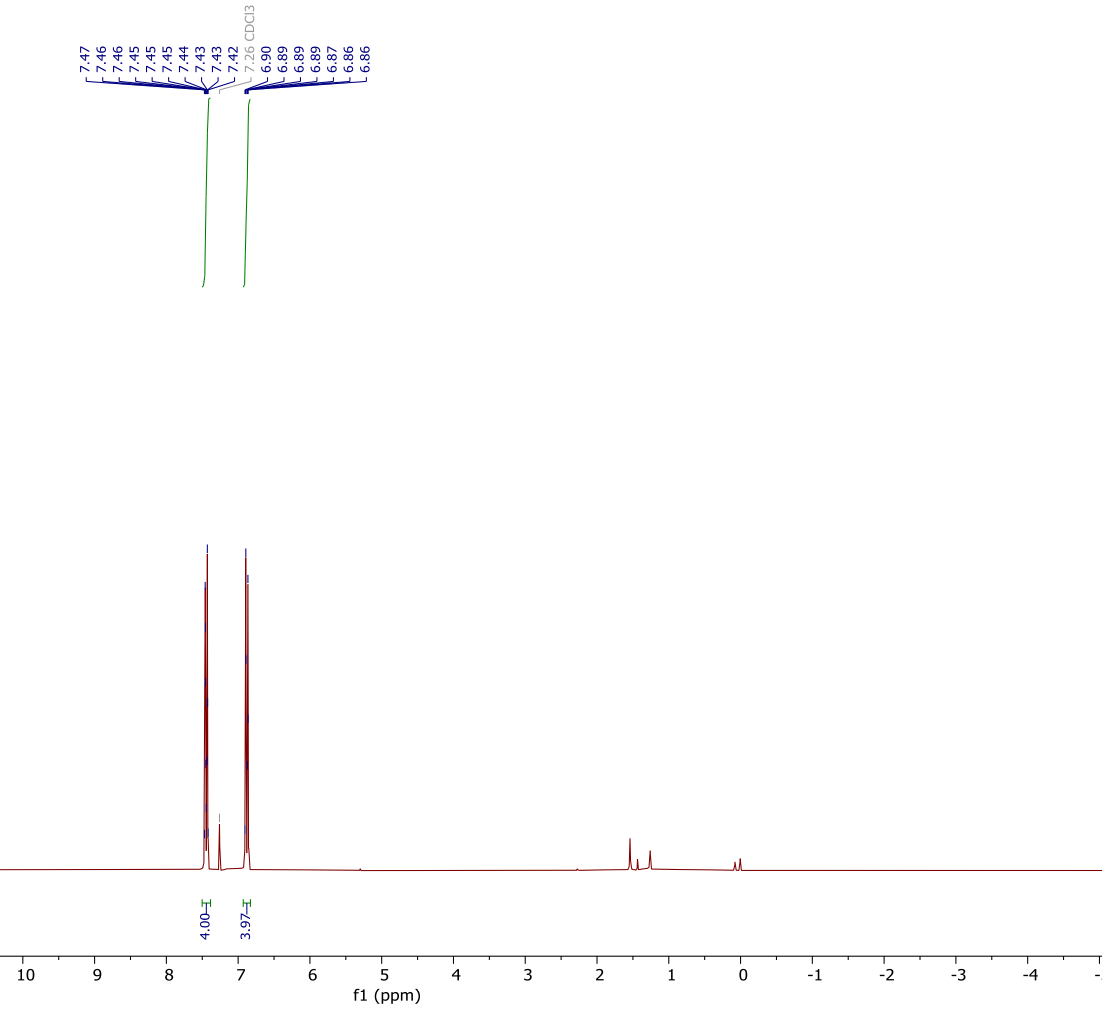

Compound 18 ^{13}C NMR in $CDCl_3$, 298 K

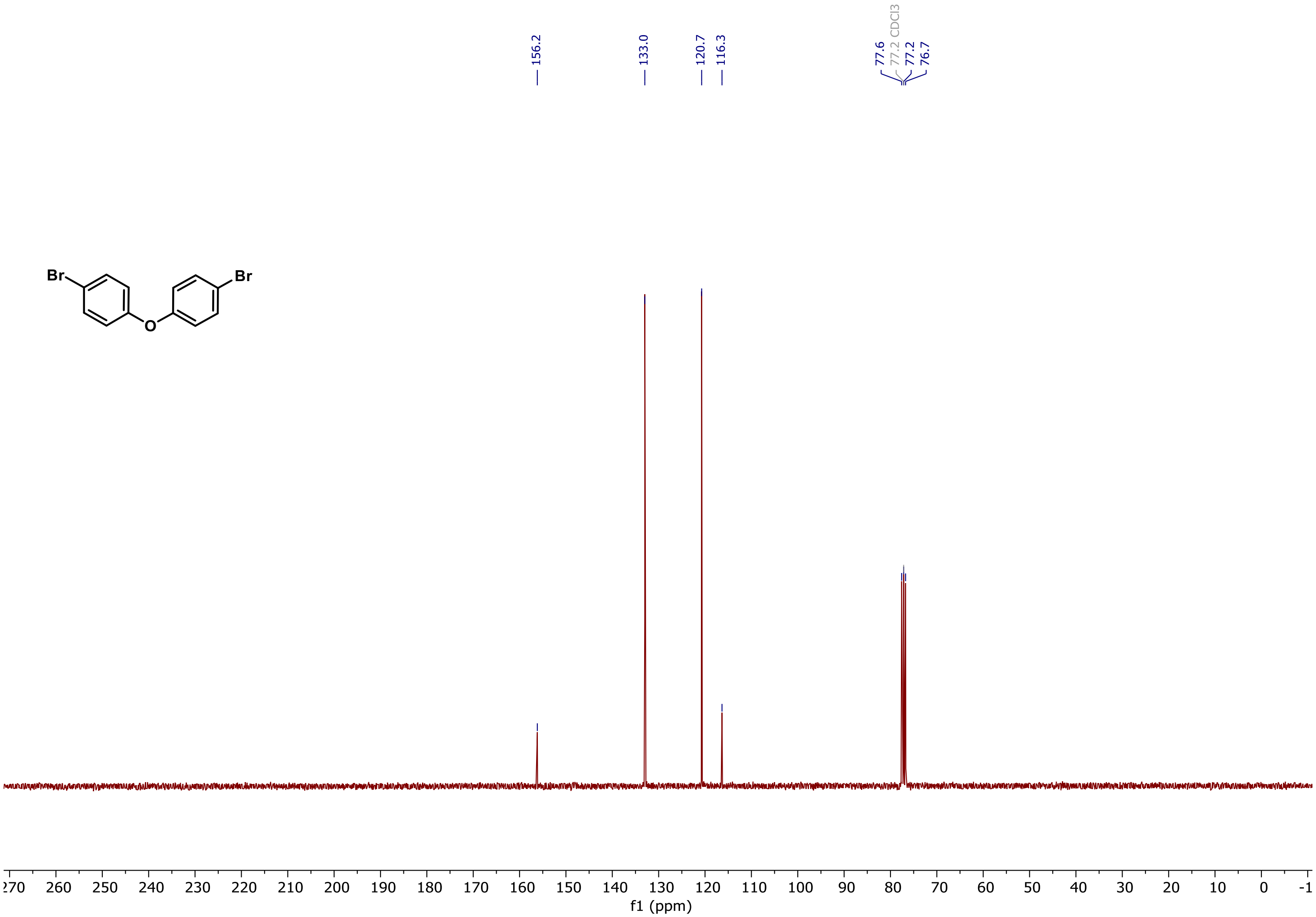

Compound 19 ^{1}H NMR in $CDCl_3$, 298 K

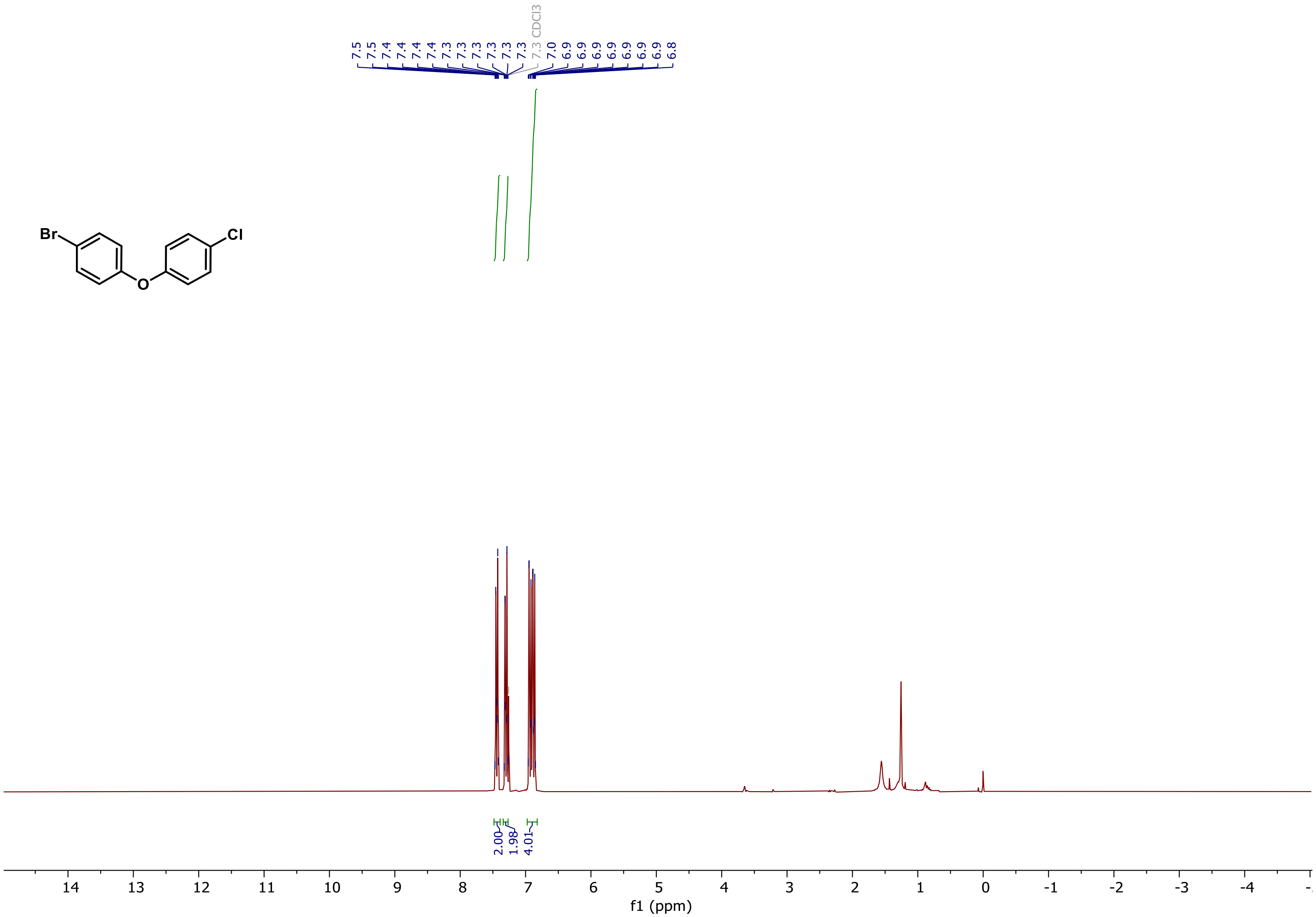

Compound 19 ^{13}C NMR in $CDCl_3$, 298 K

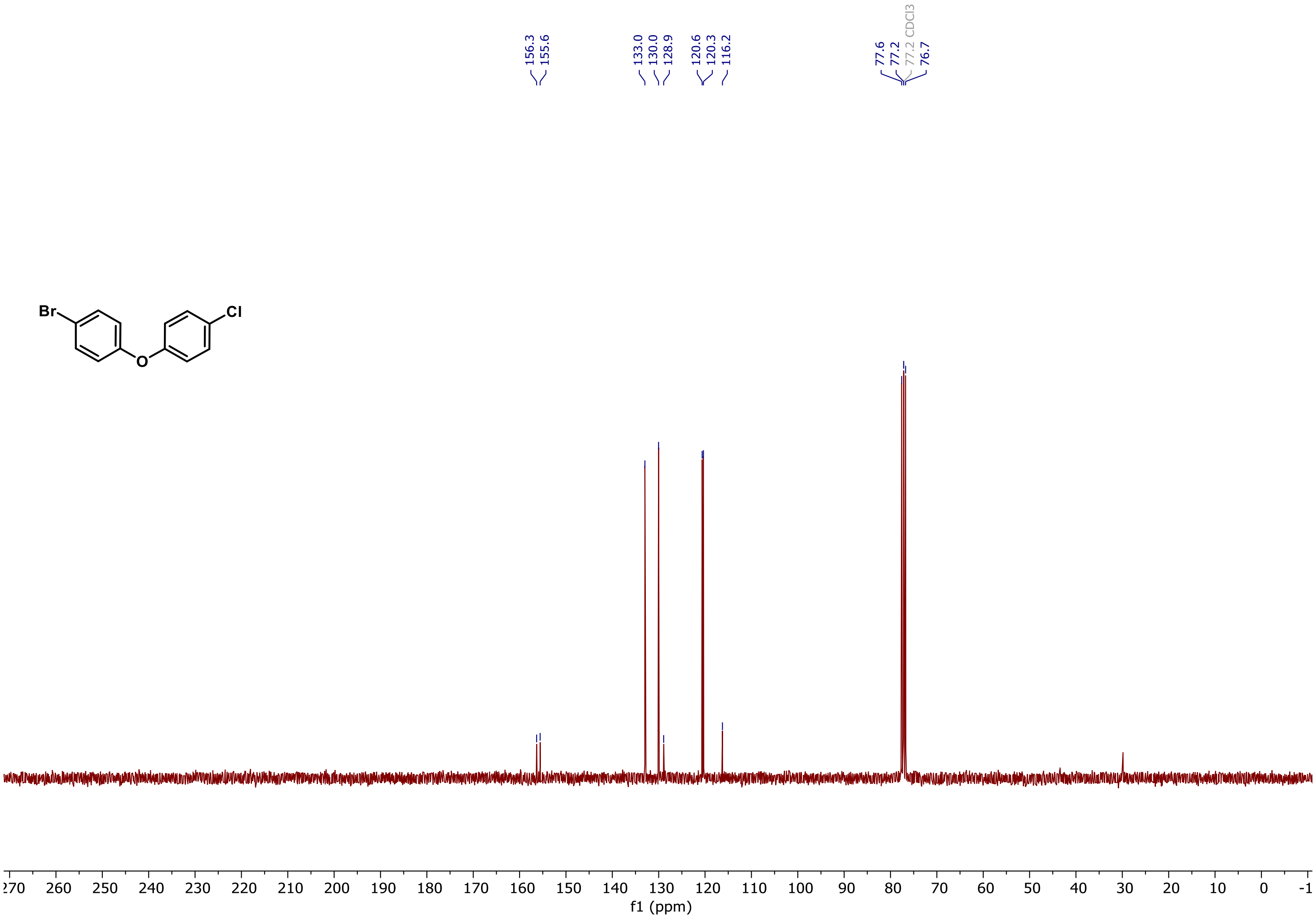

Compound 20 ^{1}H NMR in $CDCl_3$, 298 K

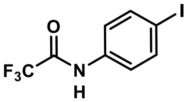

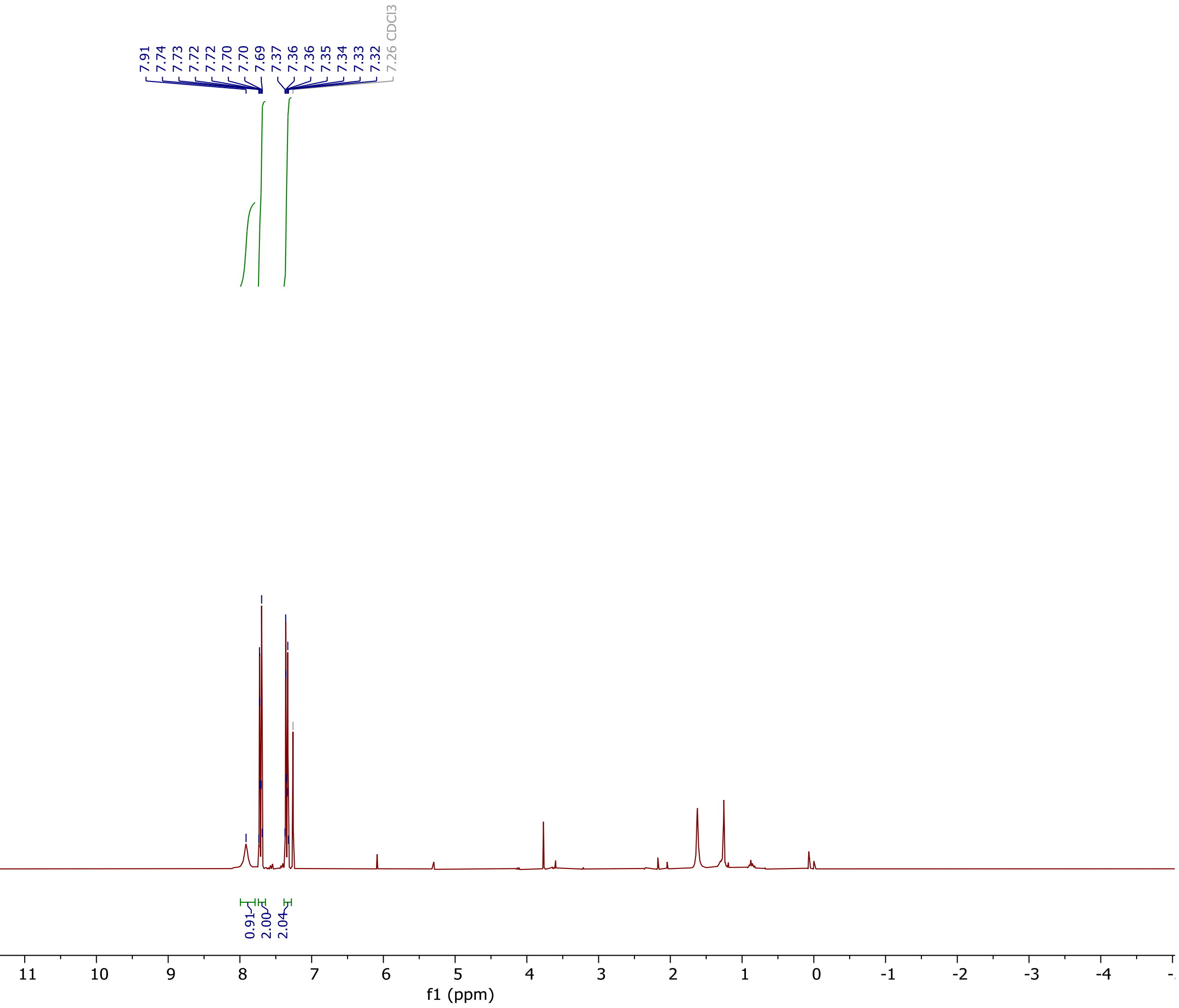

Compound 20 ^{19}F NMR in $CDCl_3$, 298 K

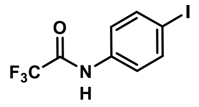

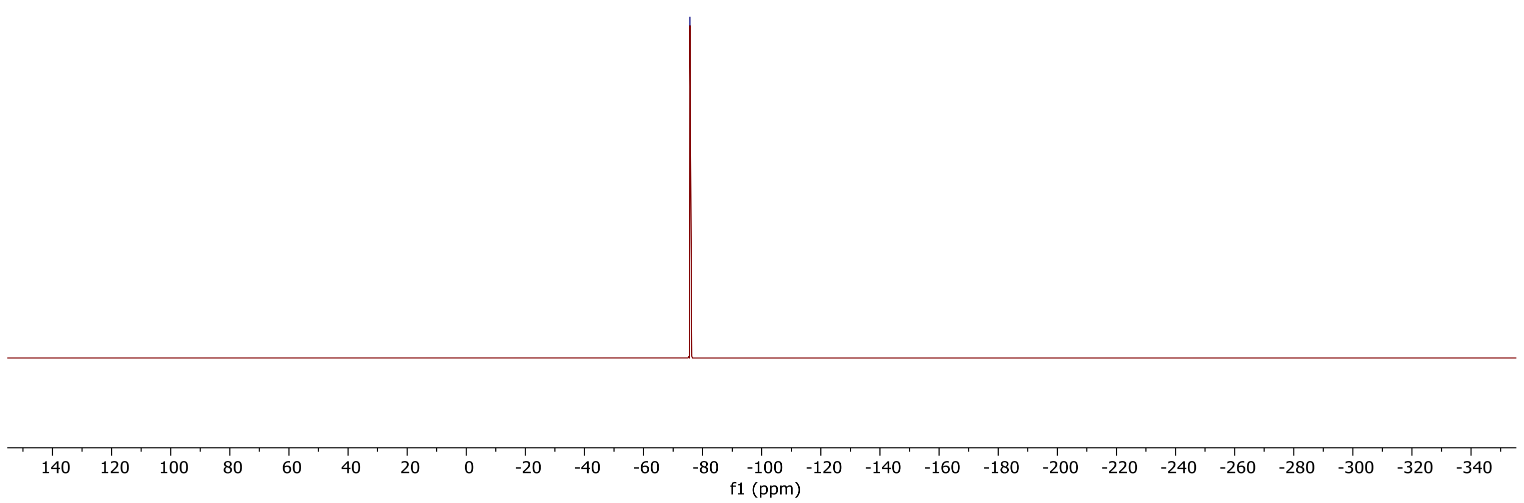

Compound 23 ^{1}H NMR in $CDCl_3$, 298 K

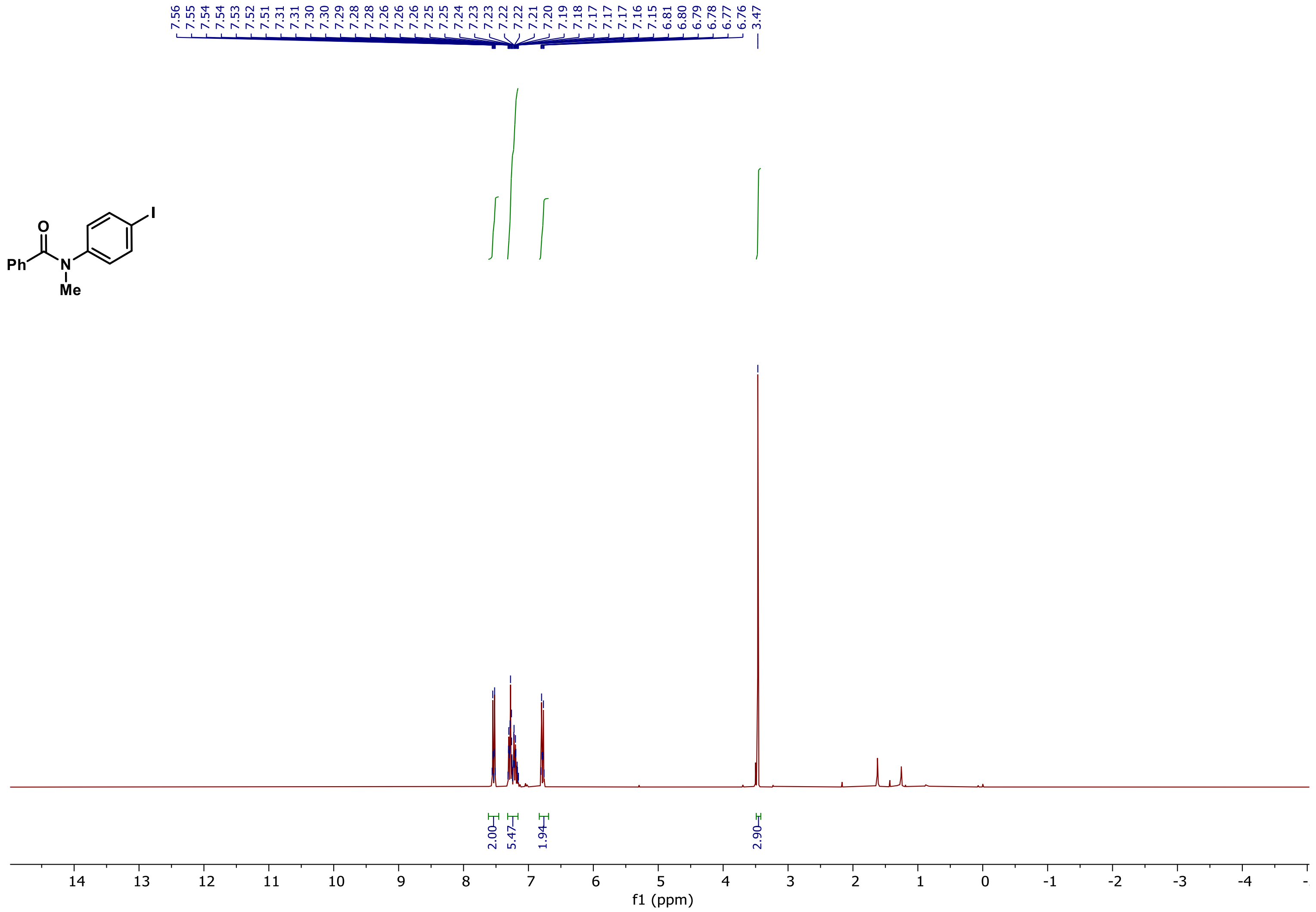

Compound 23 ^{13}C NMR in $CDCl_3$, 298 K

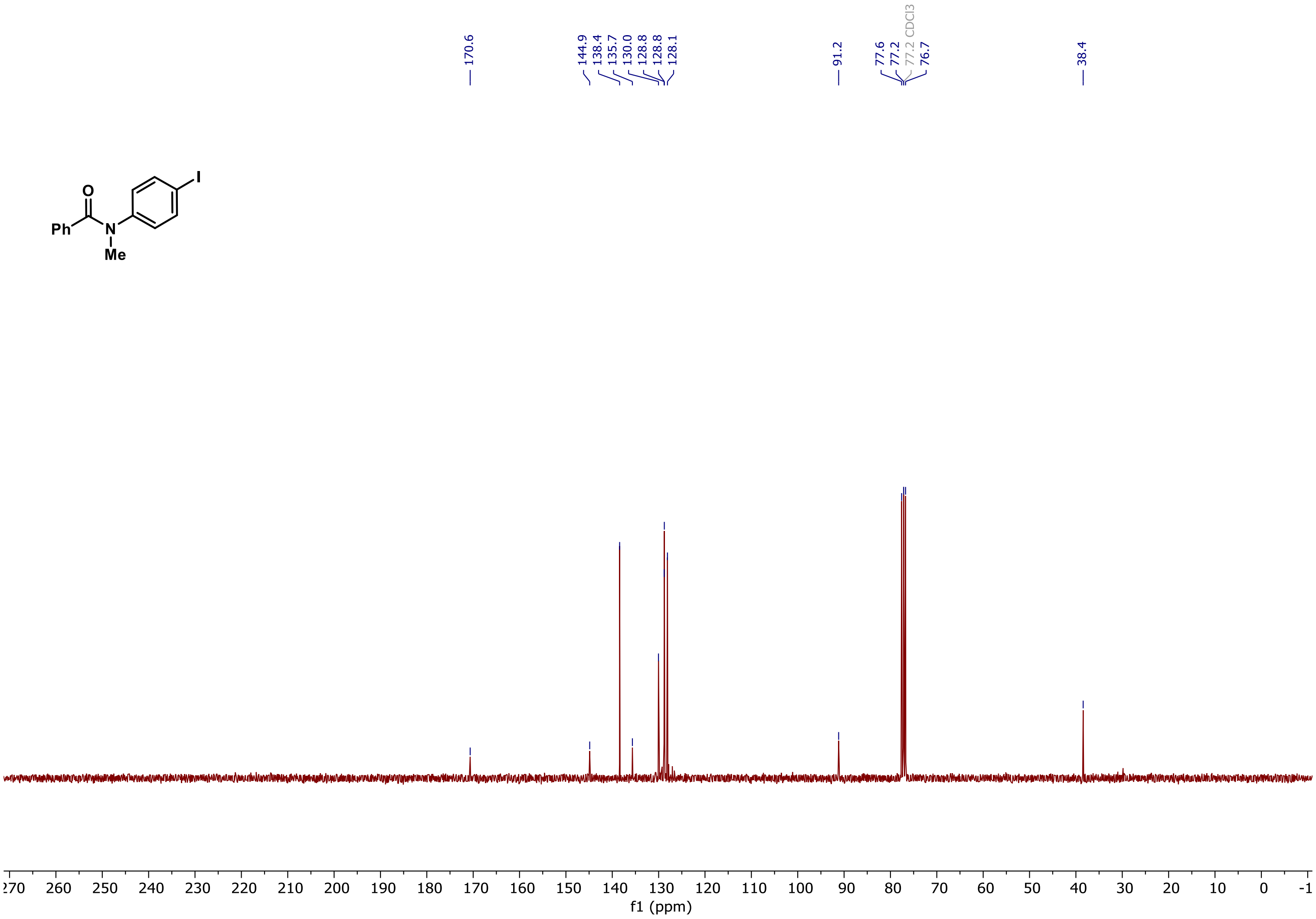

Compound 24 ^{1}H NMR in $CDCl_3$, 298 K

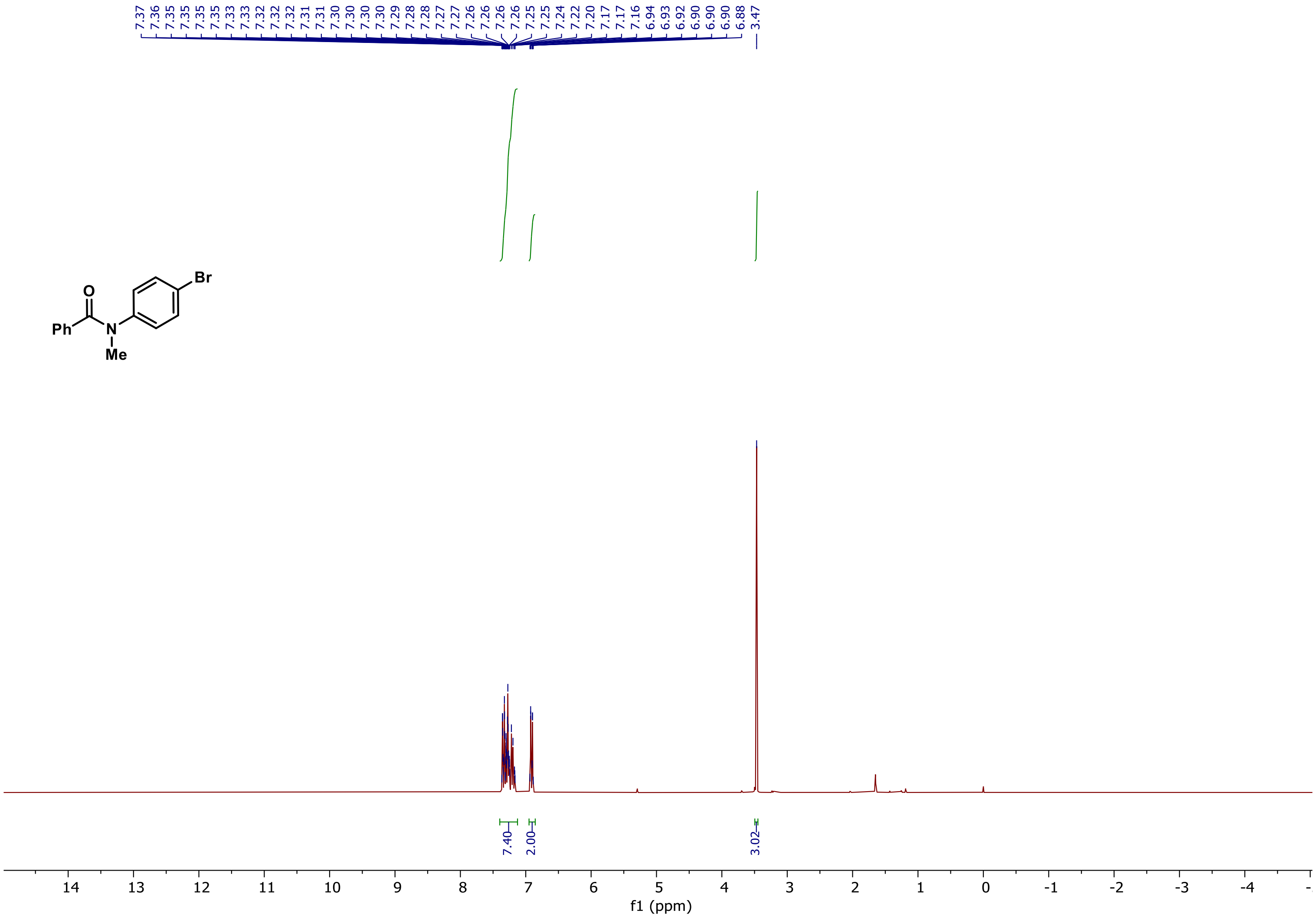

Compound 24 ^{13}C NMR in $CDCl_3$, 298 K

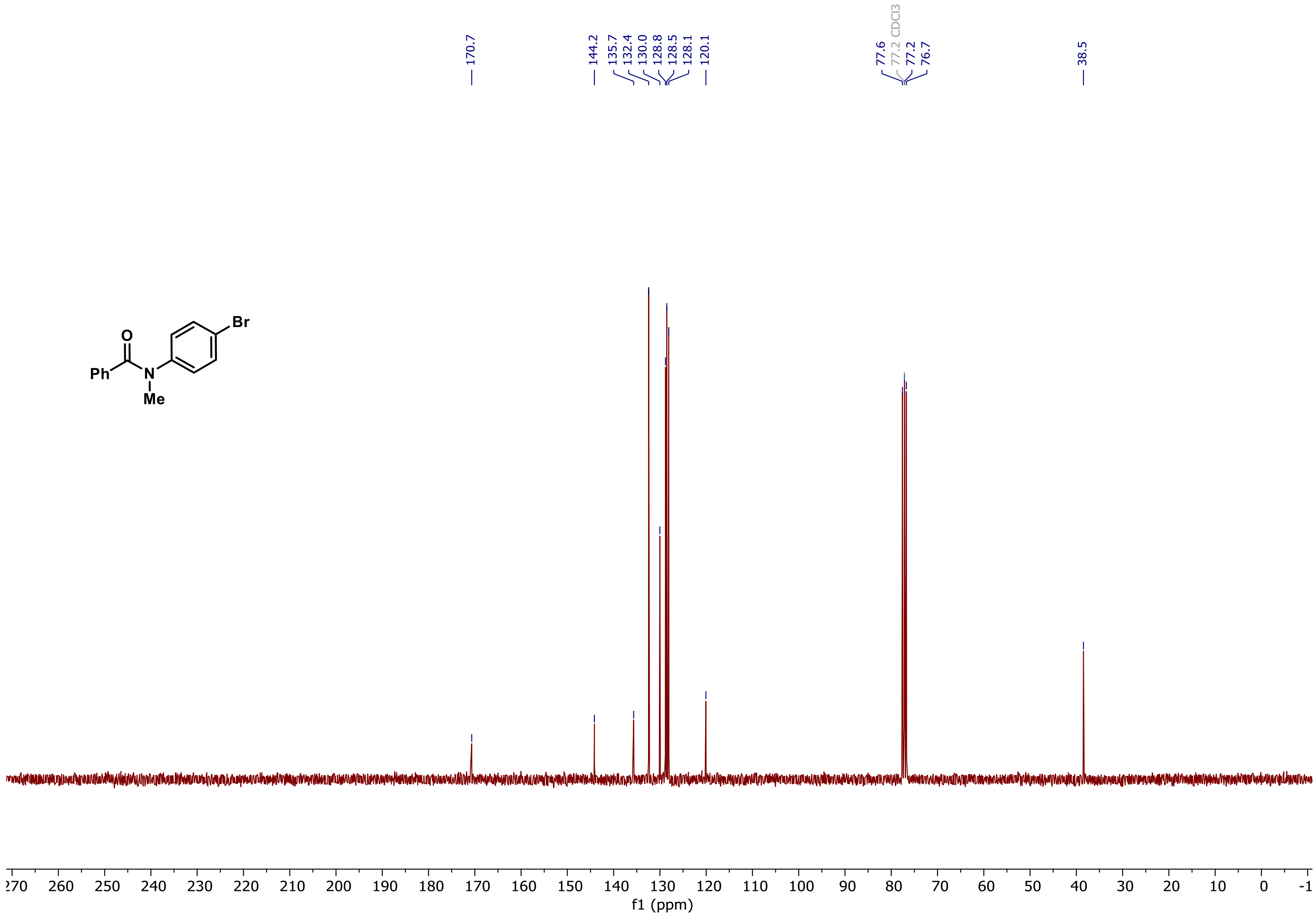

Compound 25 ^{1}H NMR in $CDCl_3$, 298 K

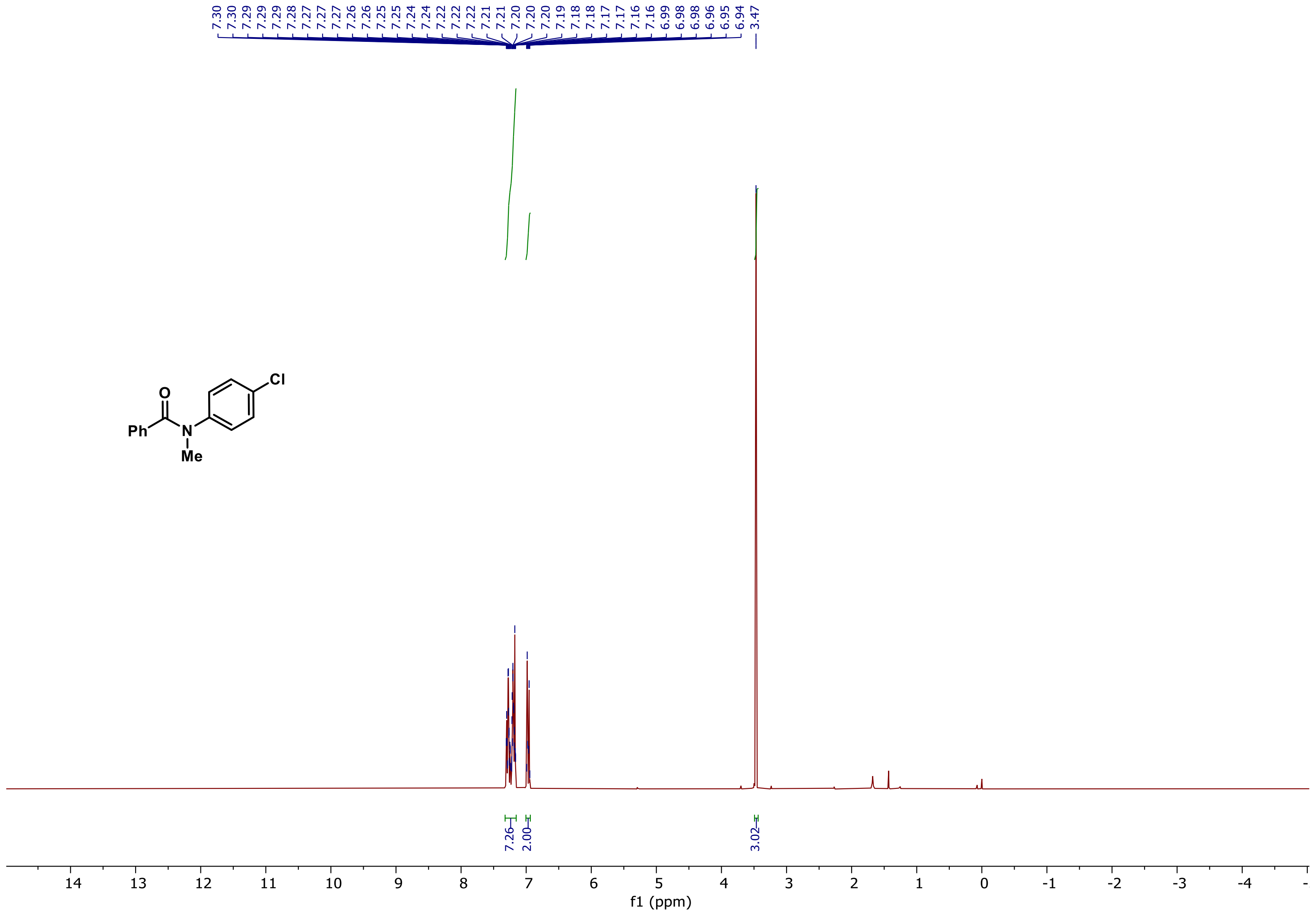

Compound 25 ^{13}C NMR in $CDCl_3$, 298 K

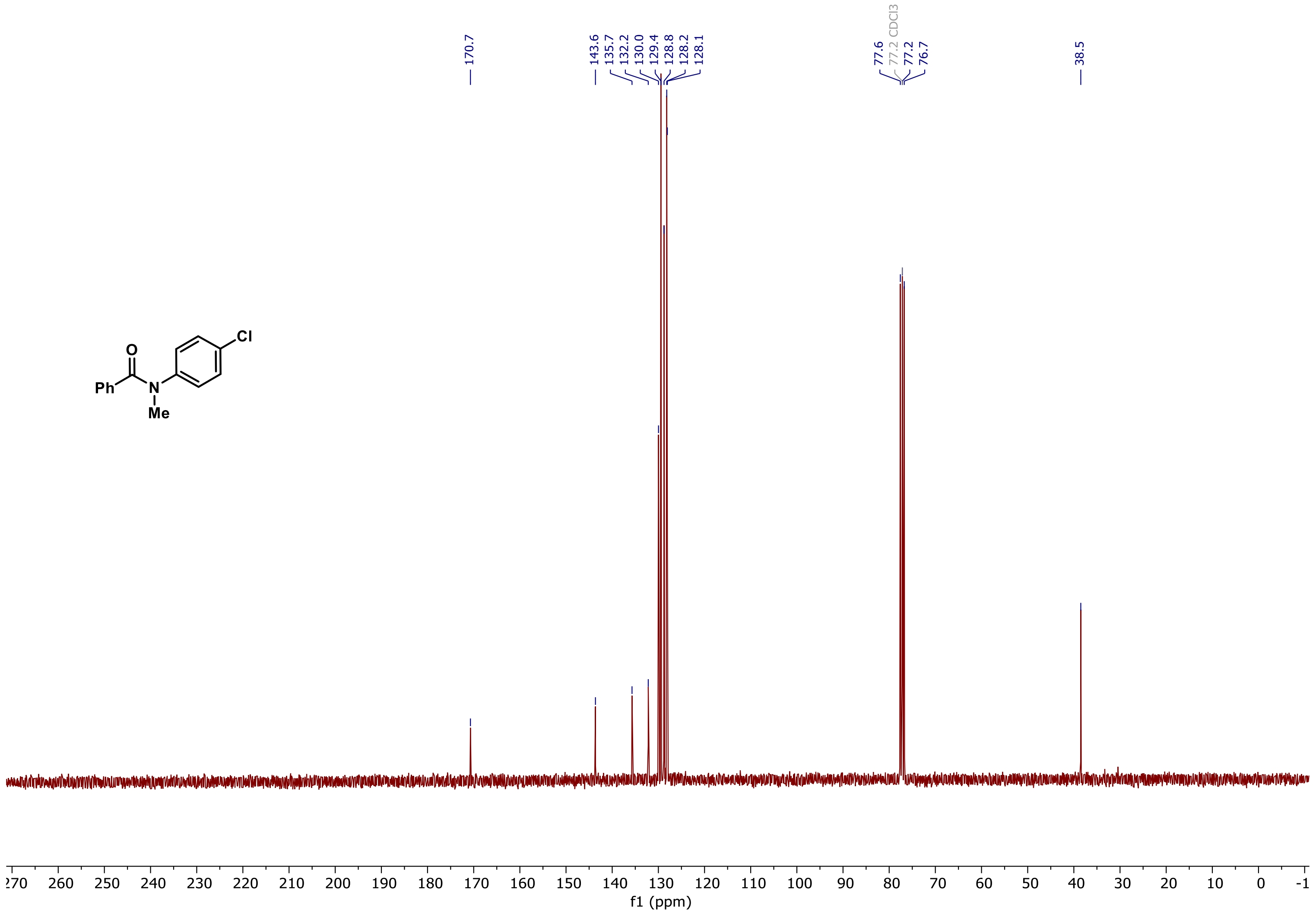

Compound 26 ^{1}H NMR in $CDCl_3$, 298 K

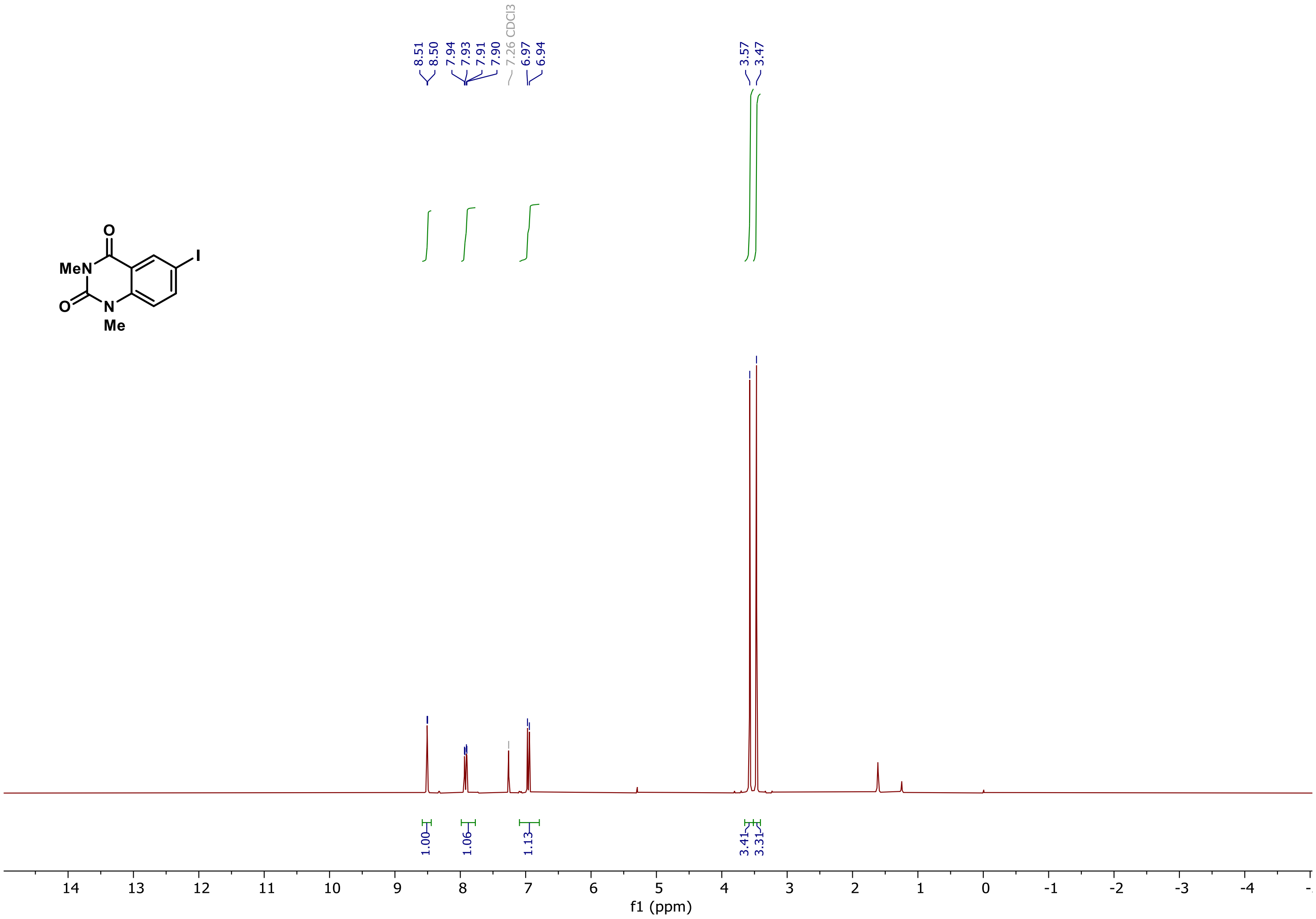

Compound 26 ^{13}C NMR in $CDCl_3$, 298 K

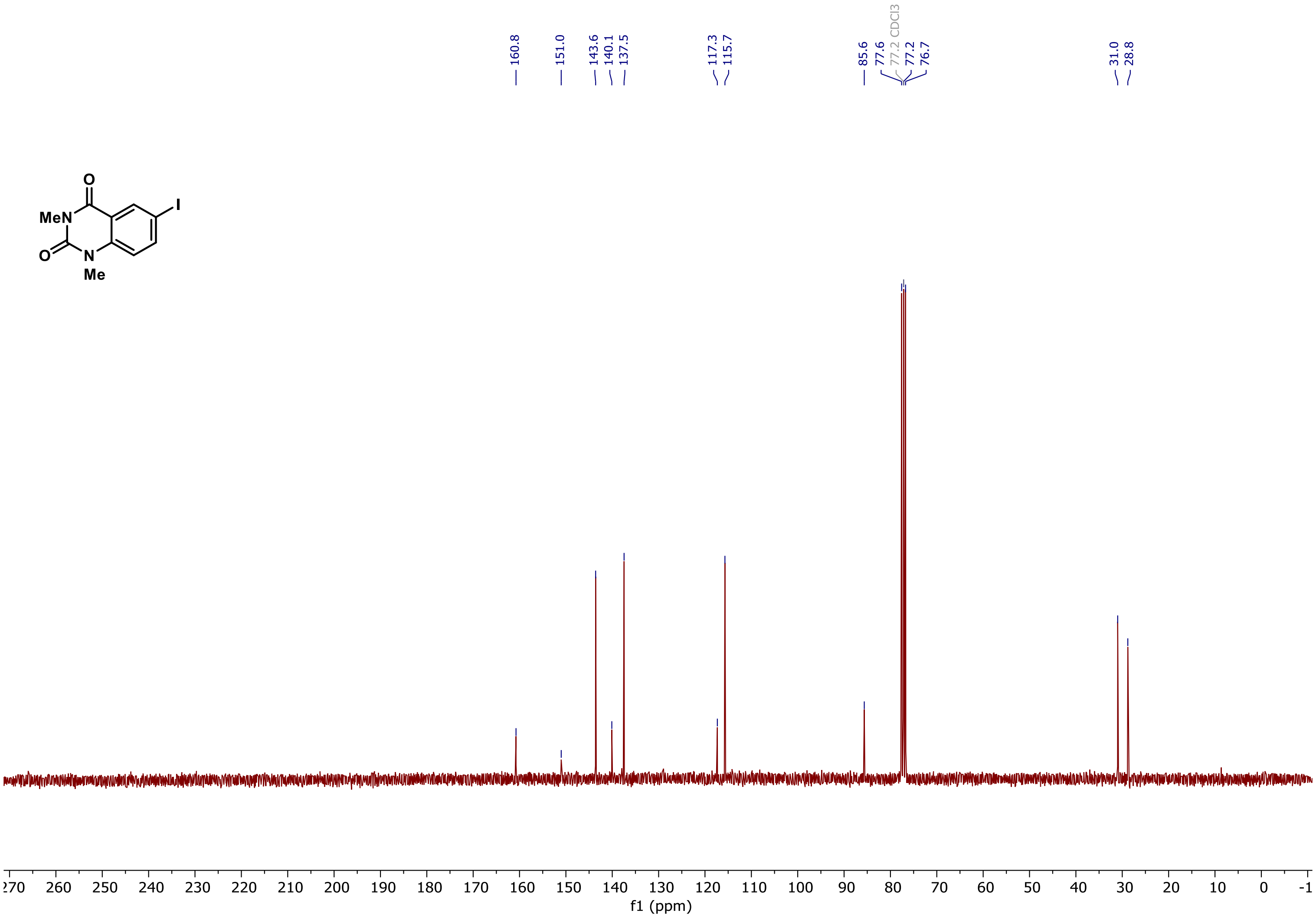

Compound 27 ^{1}H NMR in $CDCl_3$, 298 K

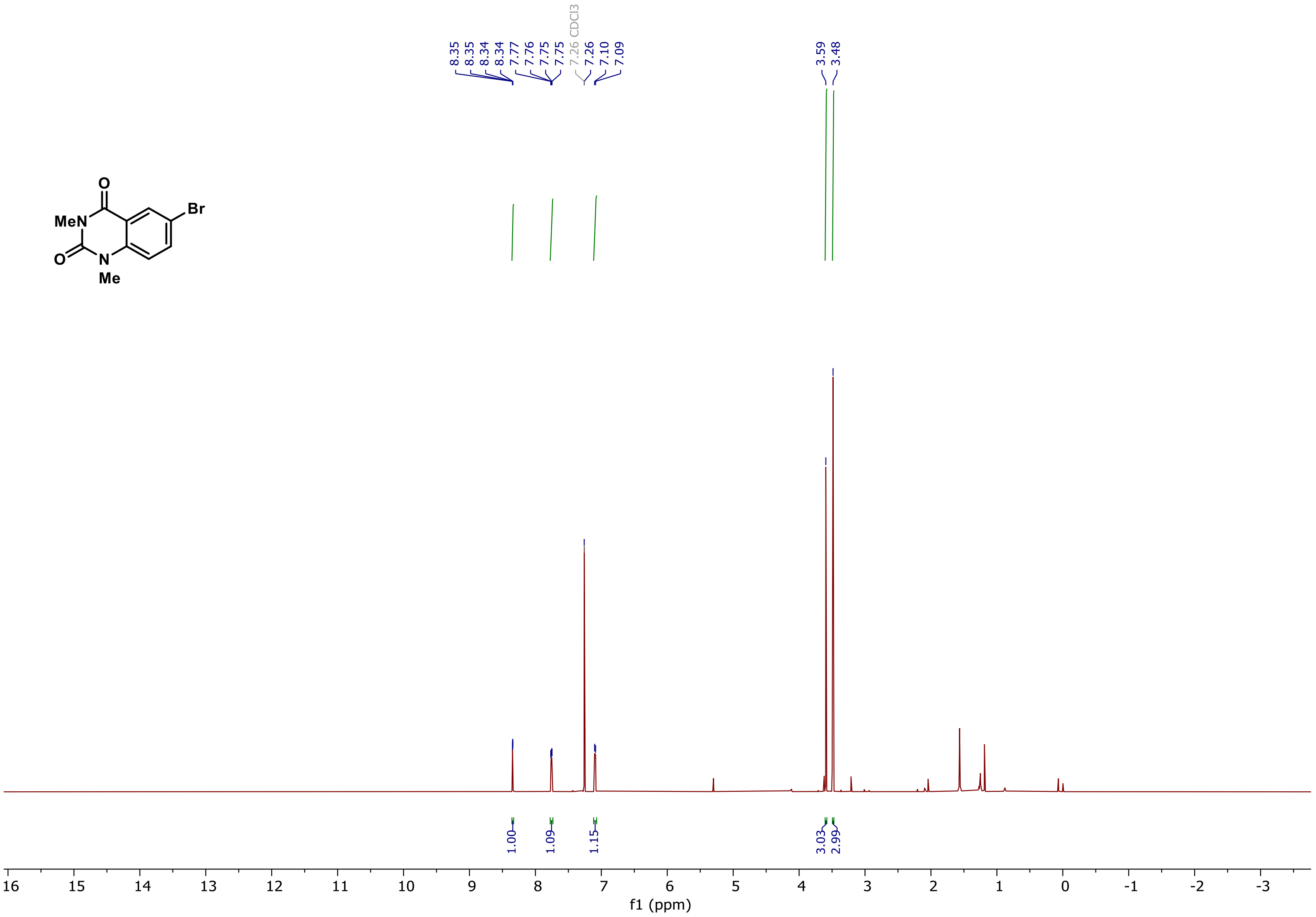

Compound 27 ^{13}C NMR in $CDCl_3$, 298 K

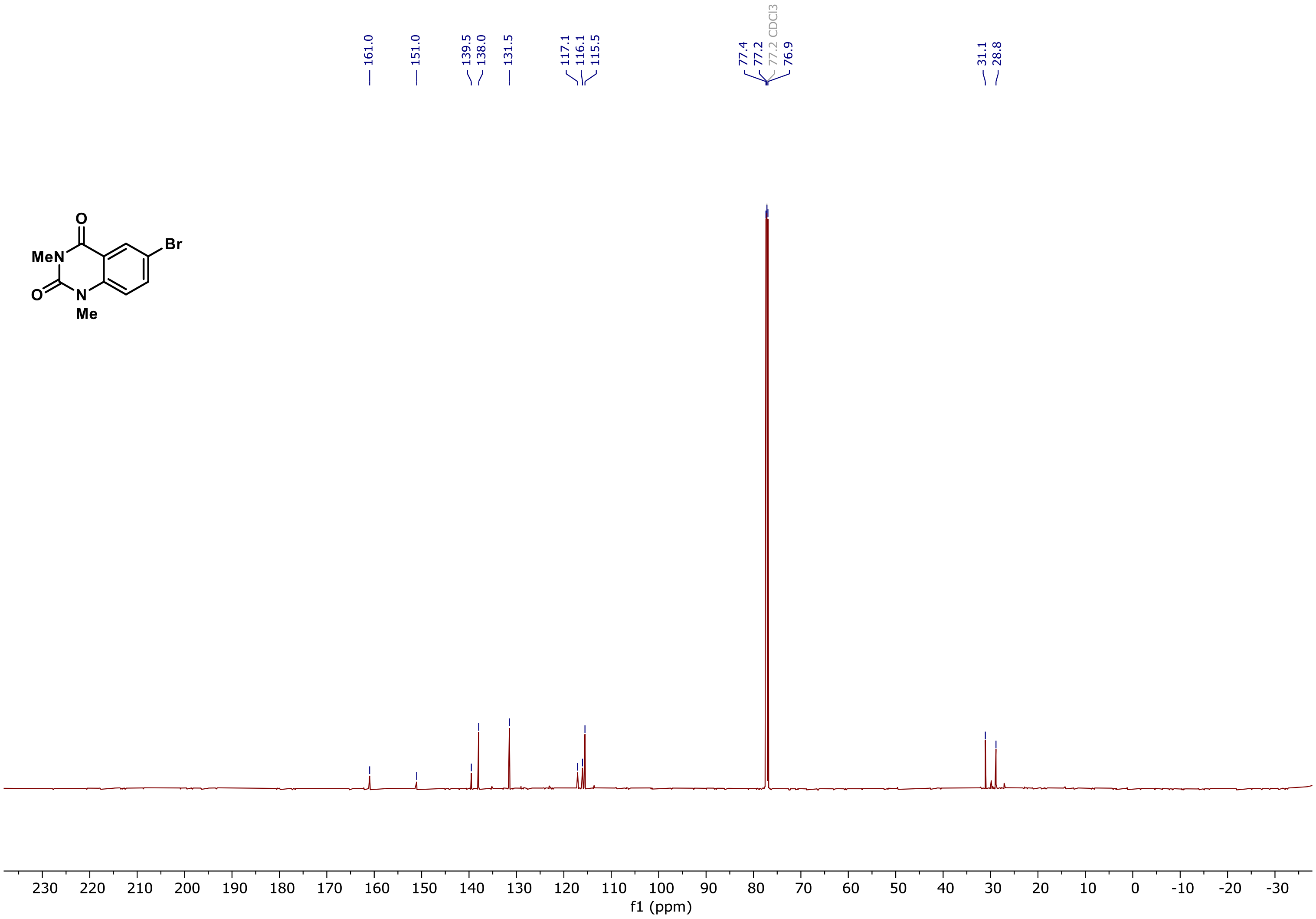

Compound 28 ^{1}H NMR in $CDCl_3$, 298 K

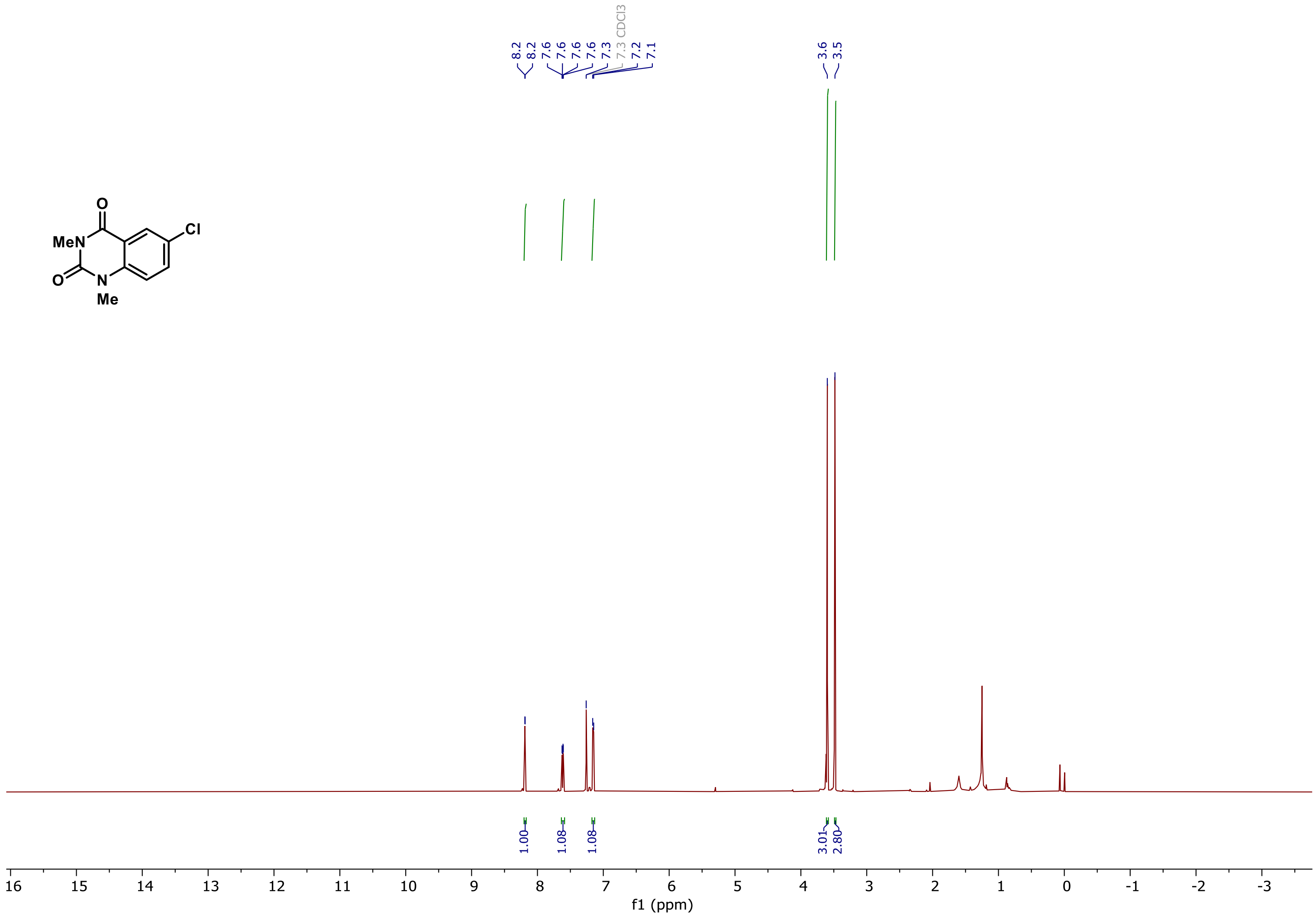

Compound 28 ^{13}C NMR in $CDCl_3$, 298 K

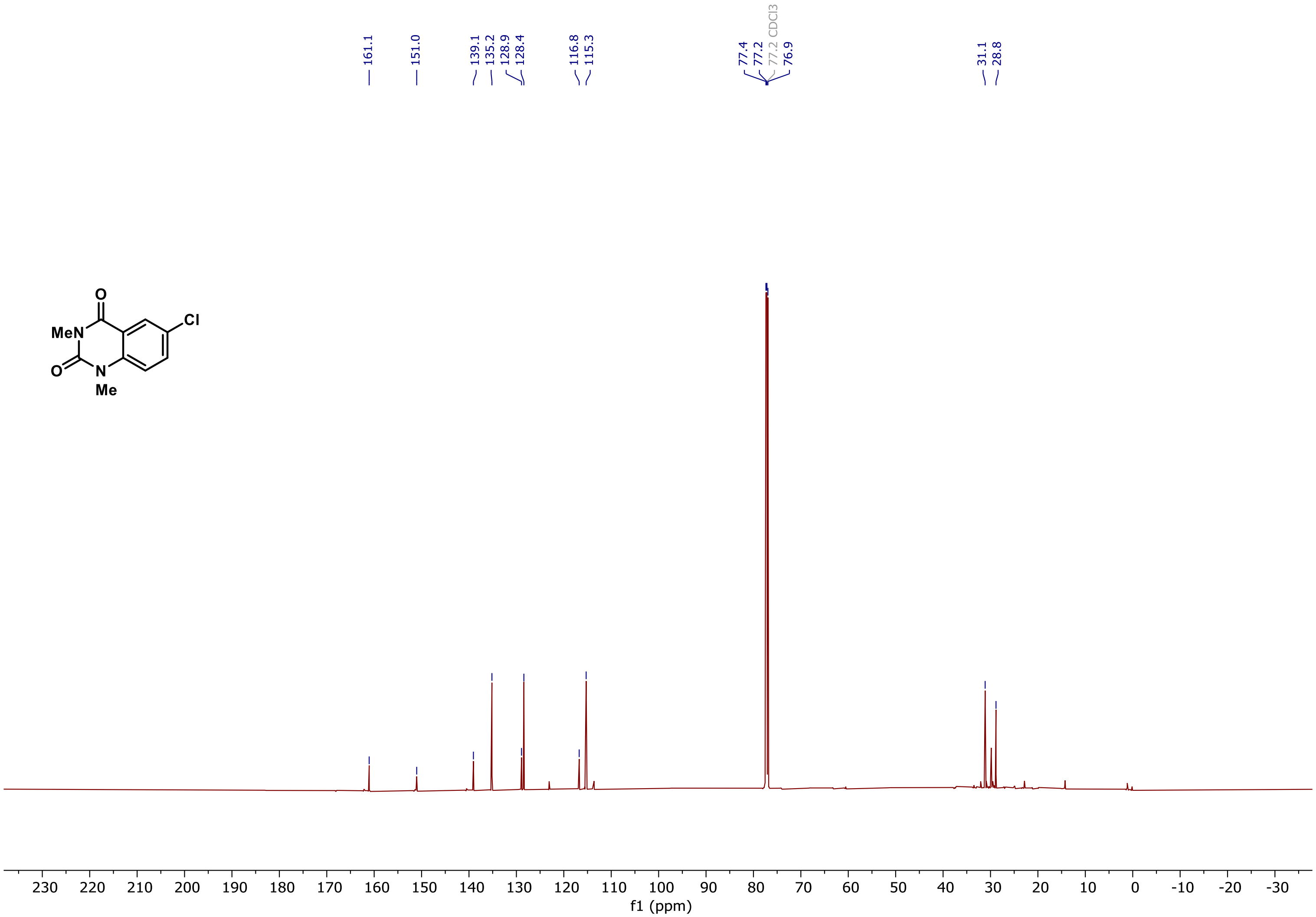

Compound 29 ^{1}H NMR in $CDCl_3$, 298 K

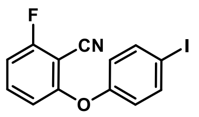

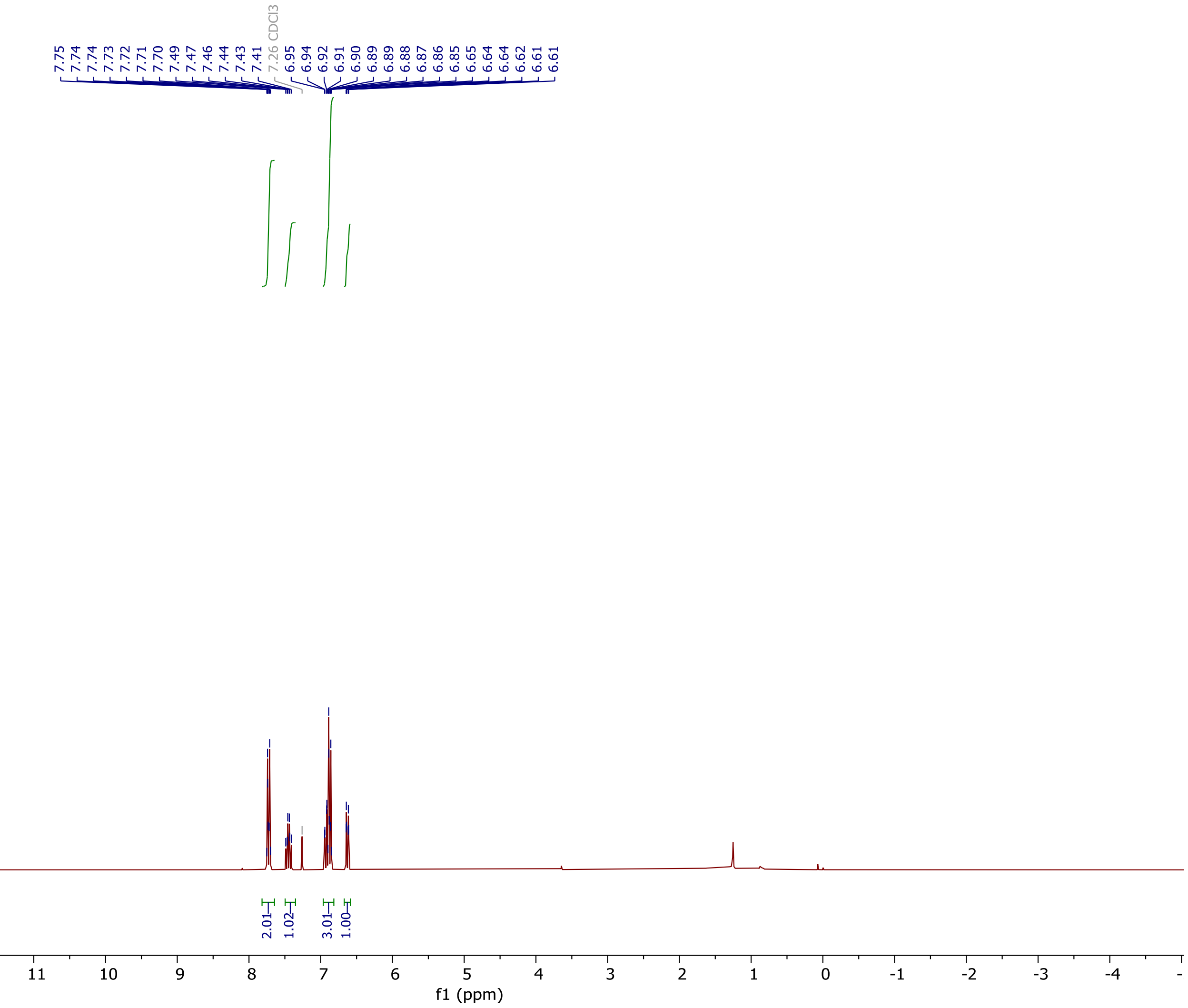

Compound 29 ^{13}C NMR in $CDCl_3$, 298 K

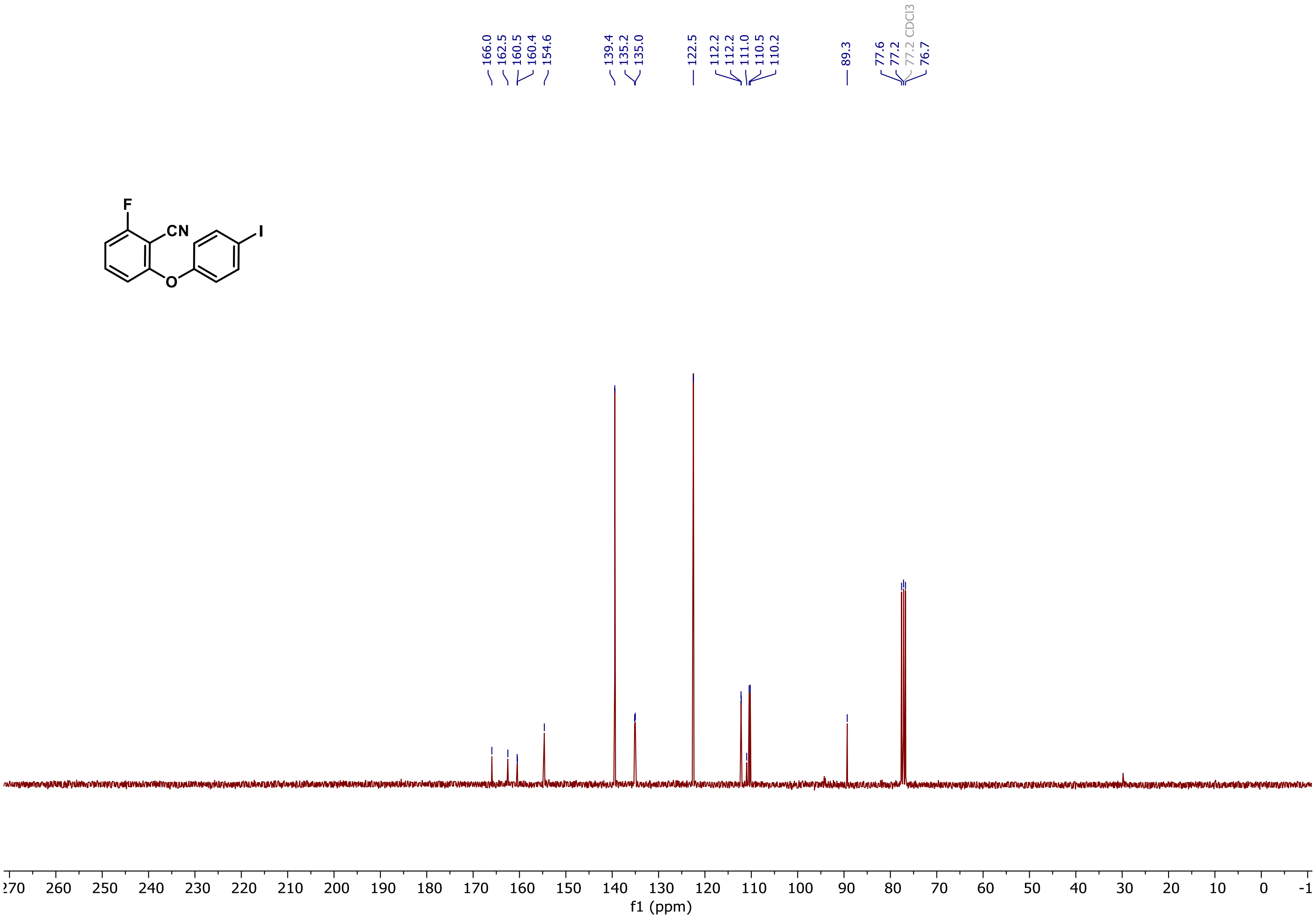

Compound 29 ^{19}F NMR in $CDCl_3$, 298 K

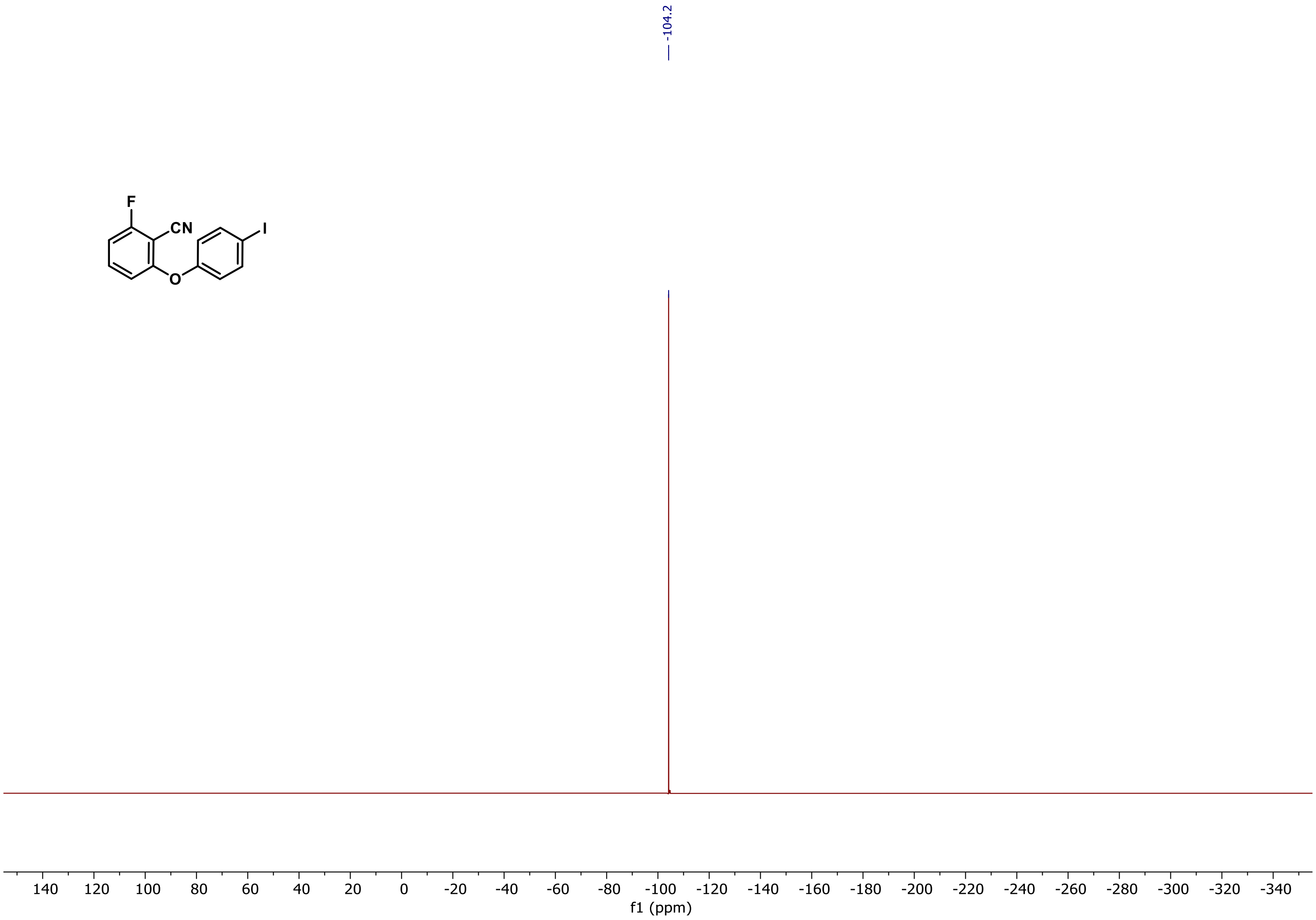

Compound 31 ^{1}H NMR in $CDCl_3$, 298 K

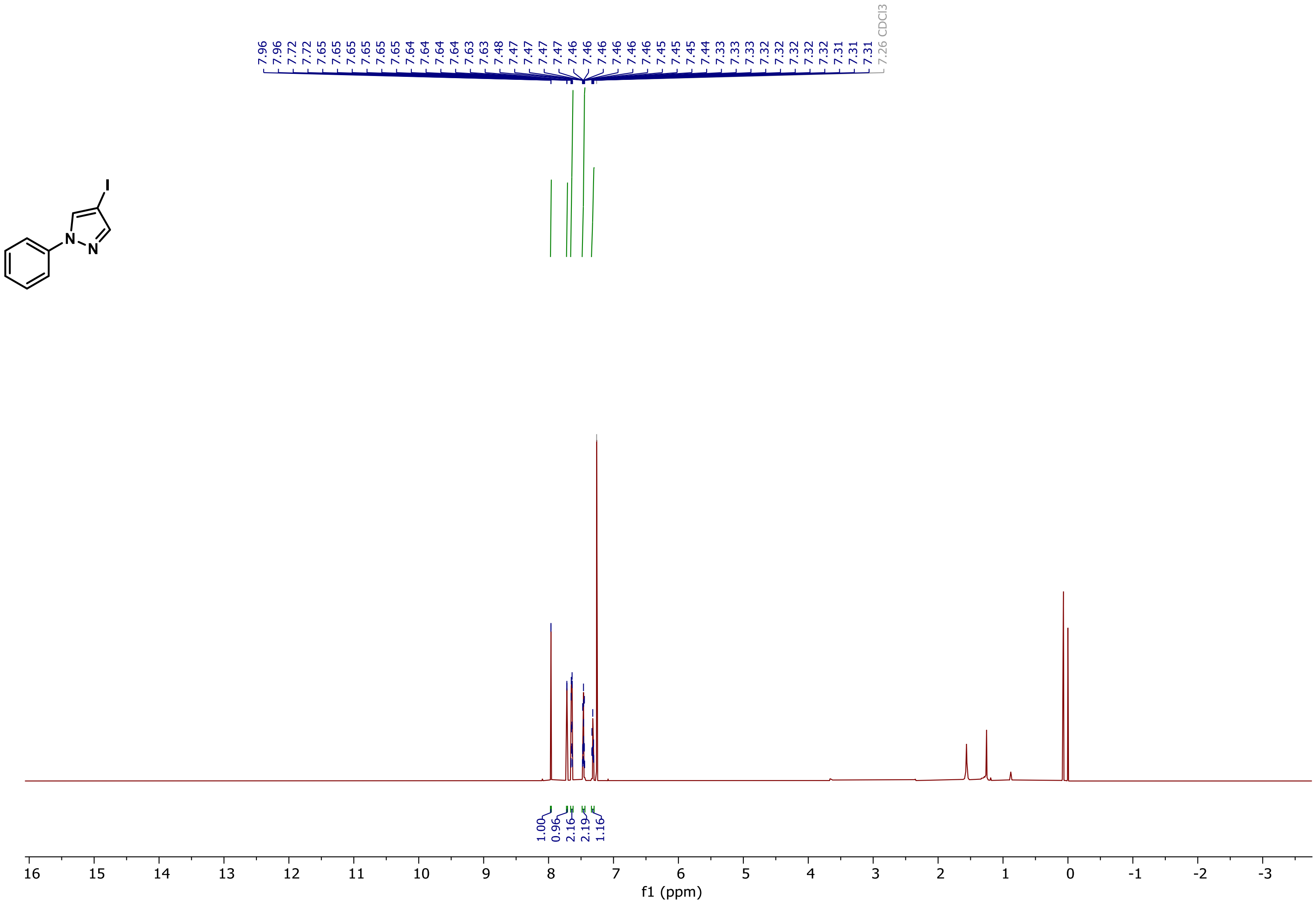

Compound 31 ^{13}C NMR in $CDCl_3$, 298 K

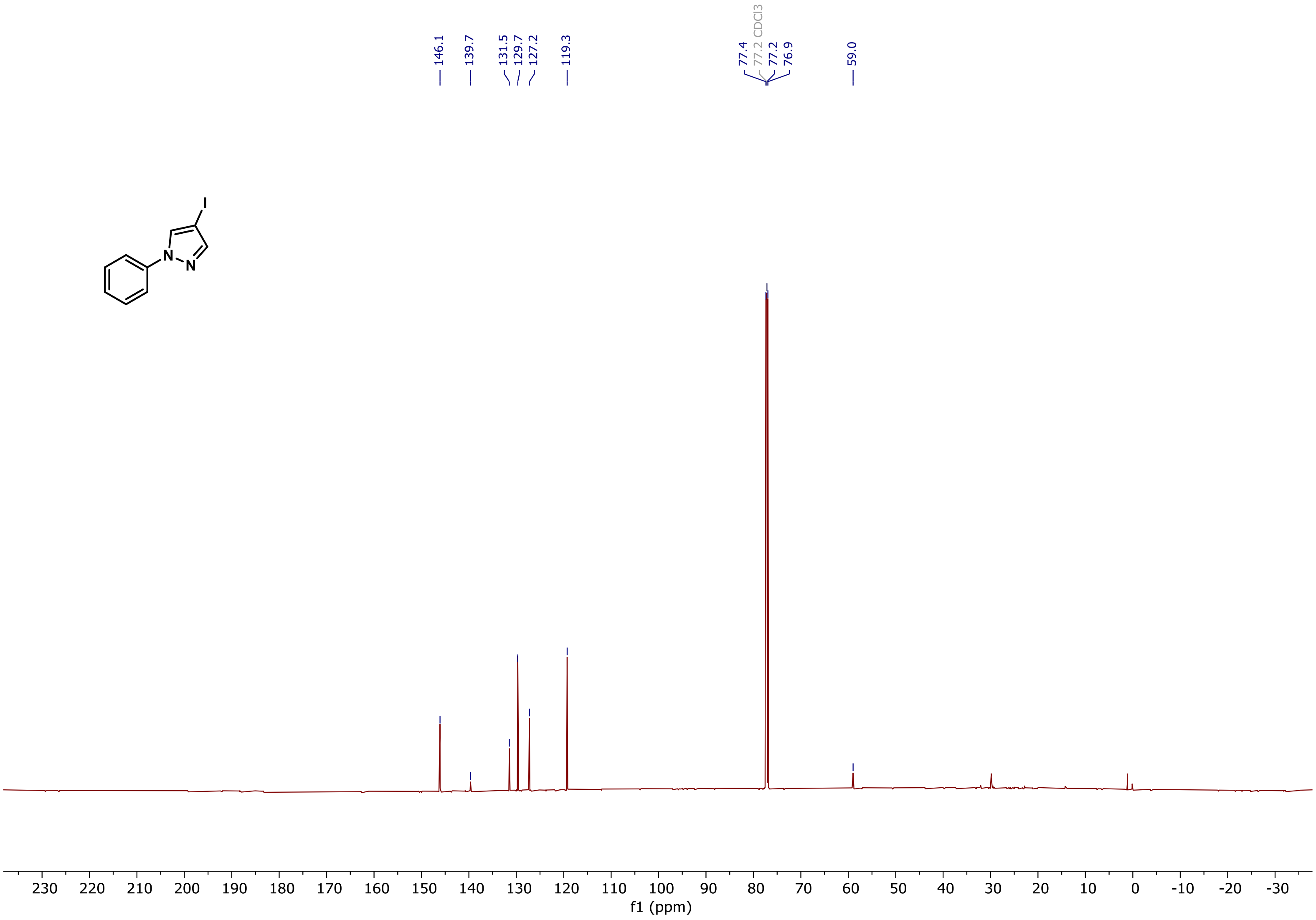

Compound 32 ^{1}H NMR in $CDCl_3$, 298 K

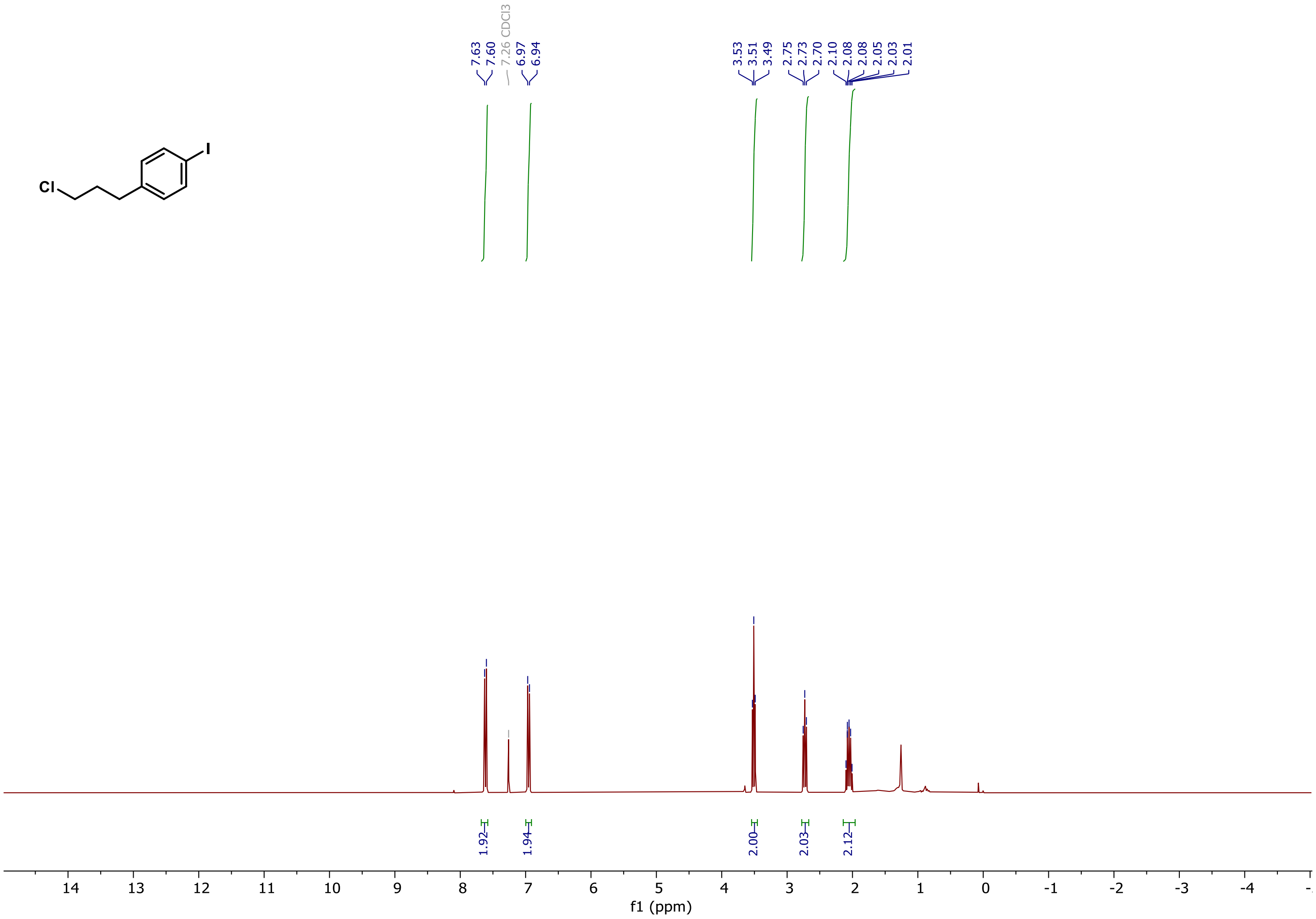

Compound 32 ^{13}C NMR in $CDCl_3$, 298 K

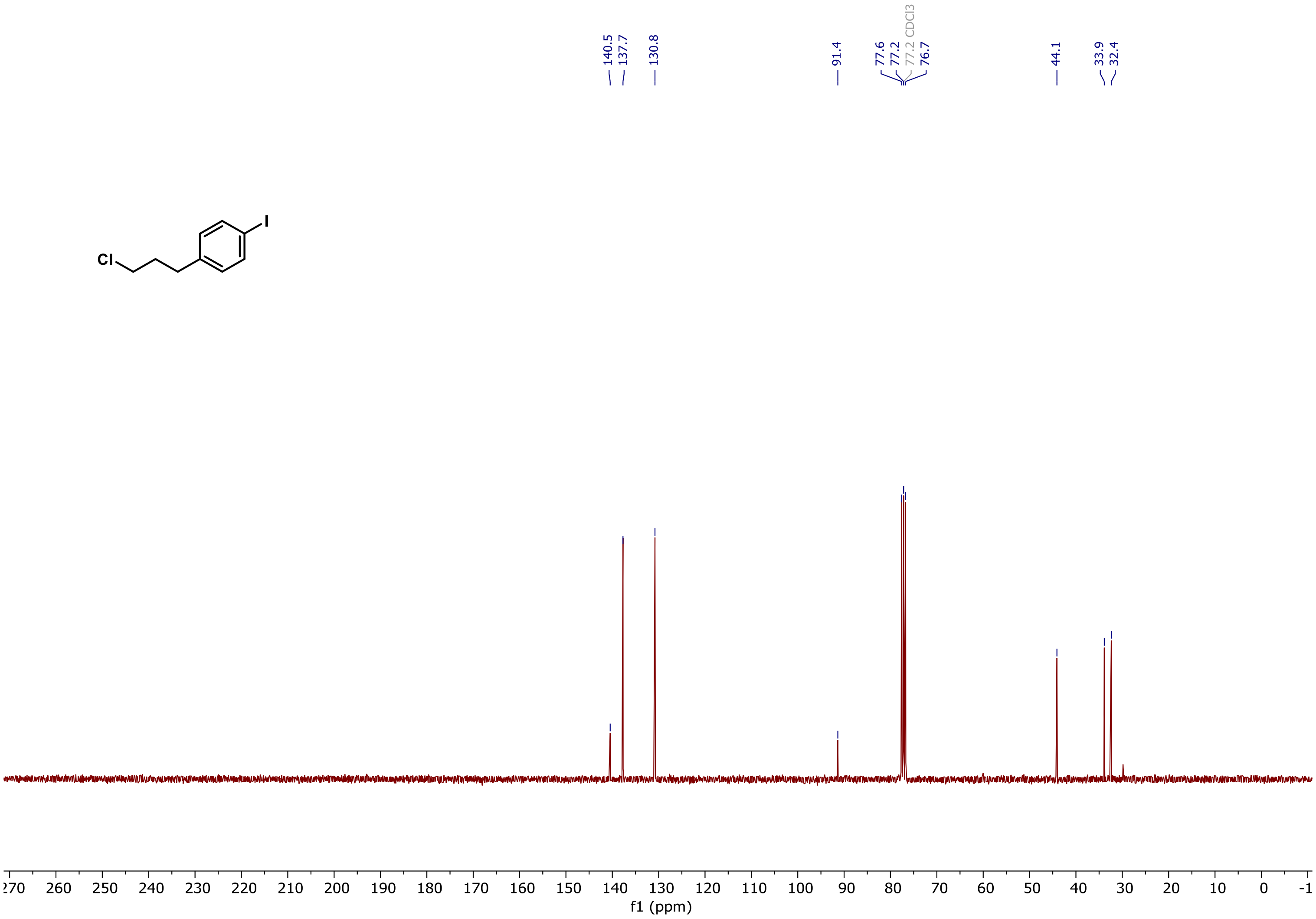

Compound 33 ^{1}H NMR in $CDCl_3$, 298 K

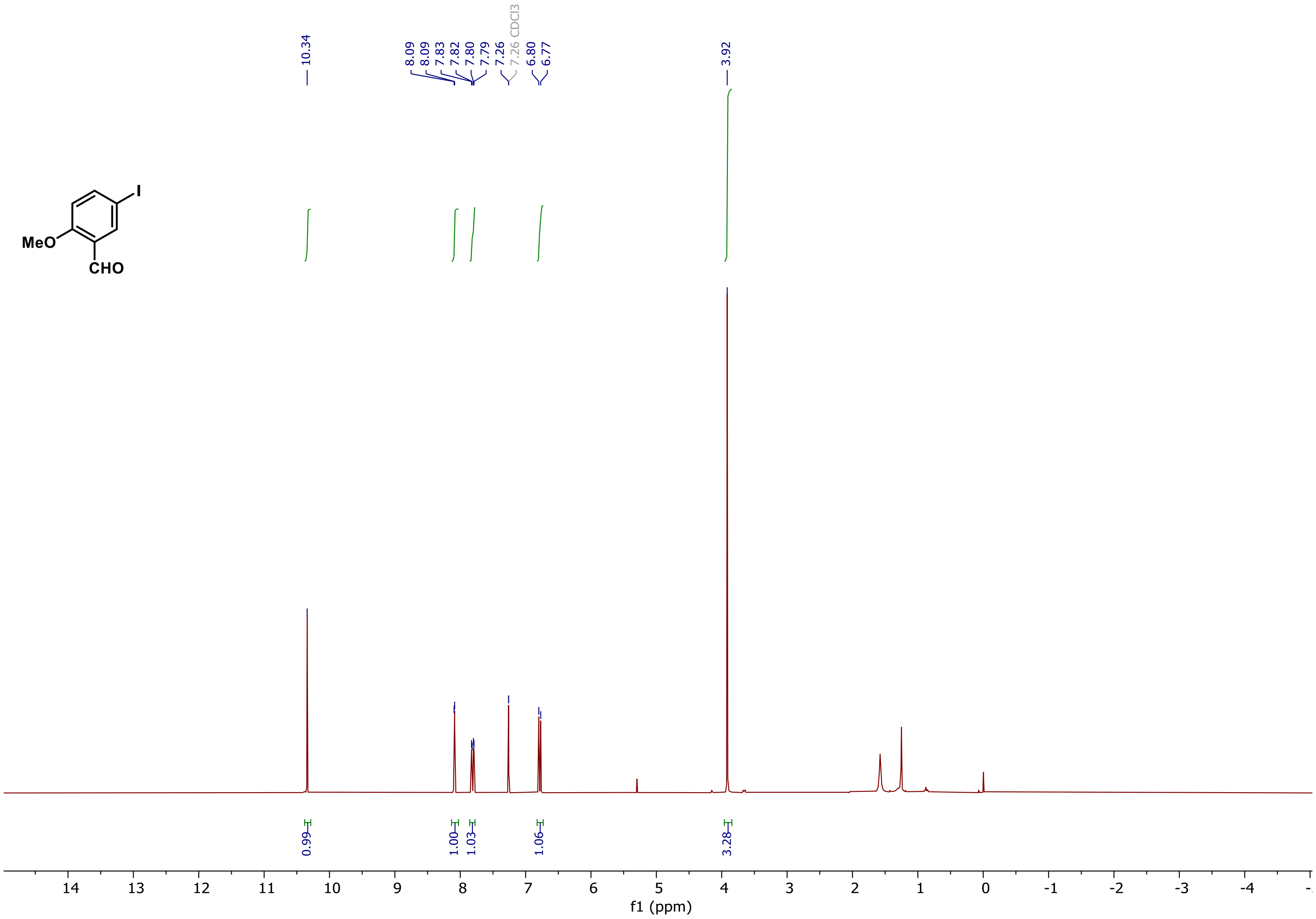

Compound 33 ^{13}C NMR in $CDCl_3$, 298 K

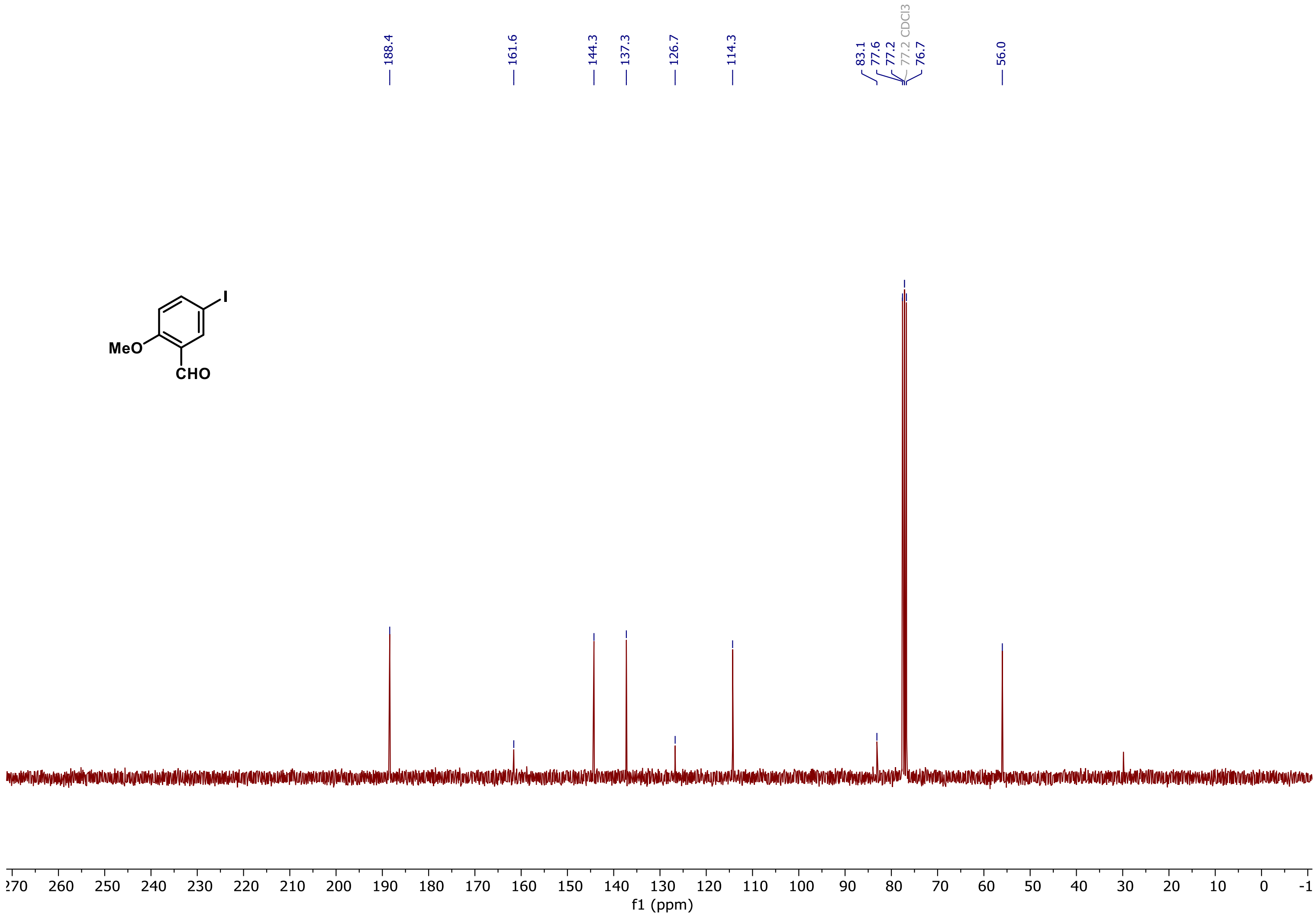

Compound 34 ^{1}H NMR in $CDCl_3$, 298 K

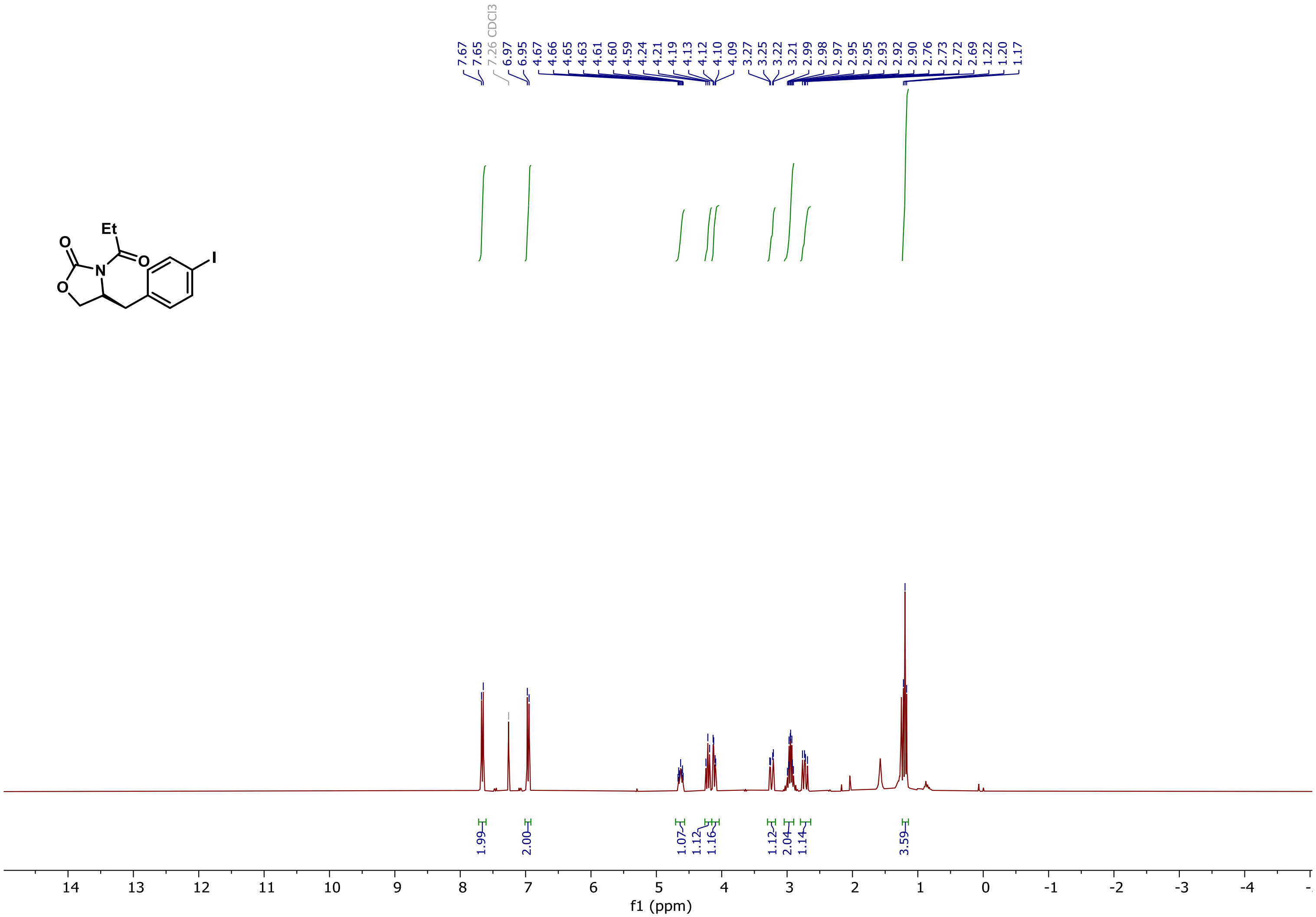

Compound 34 ^{13}C NMR in $CDCl_3$, 298 K

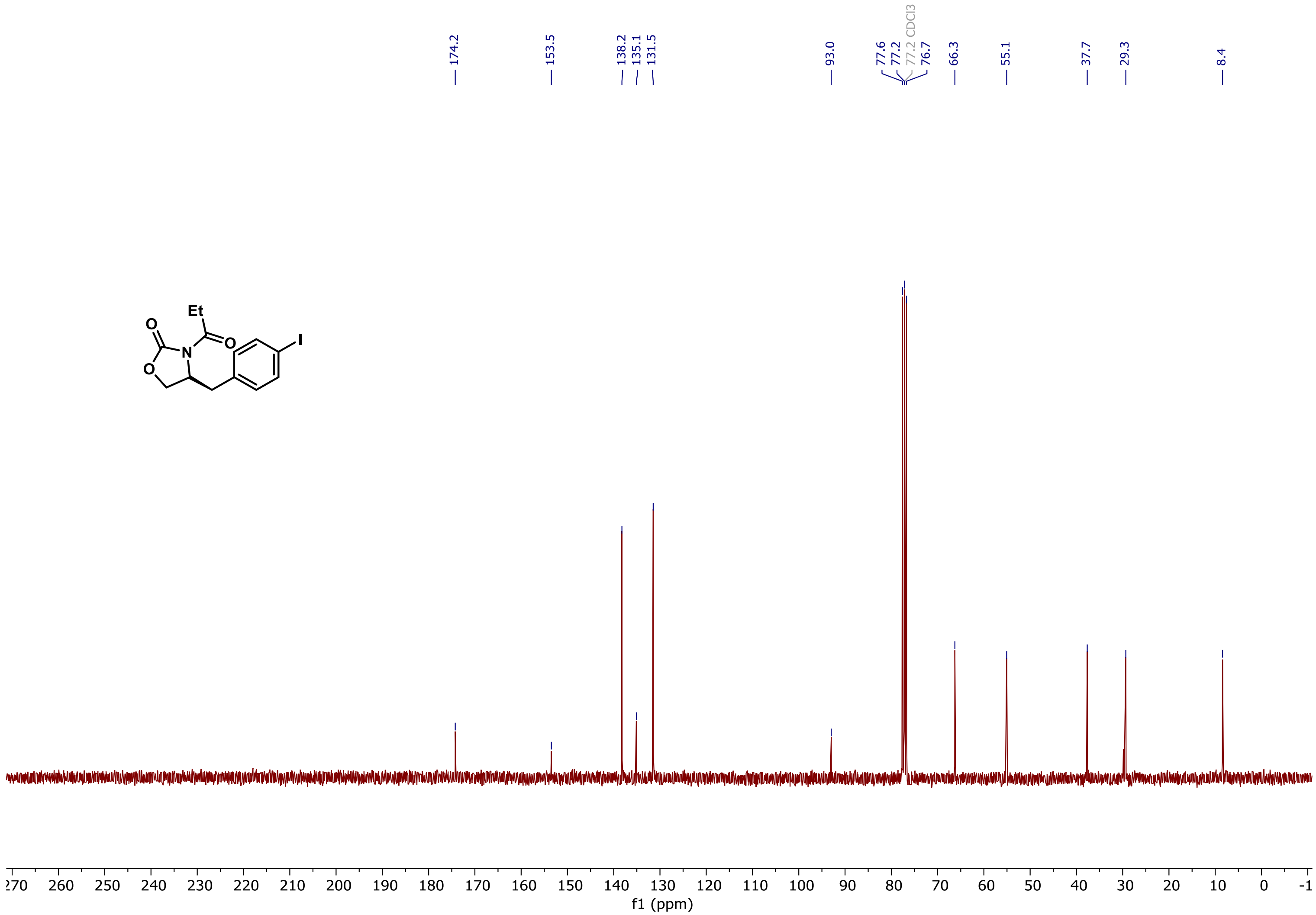

Compound 35 ^{1}H NMR in $CDCl_3$, 298 K

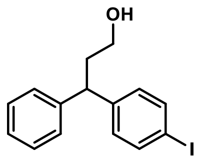

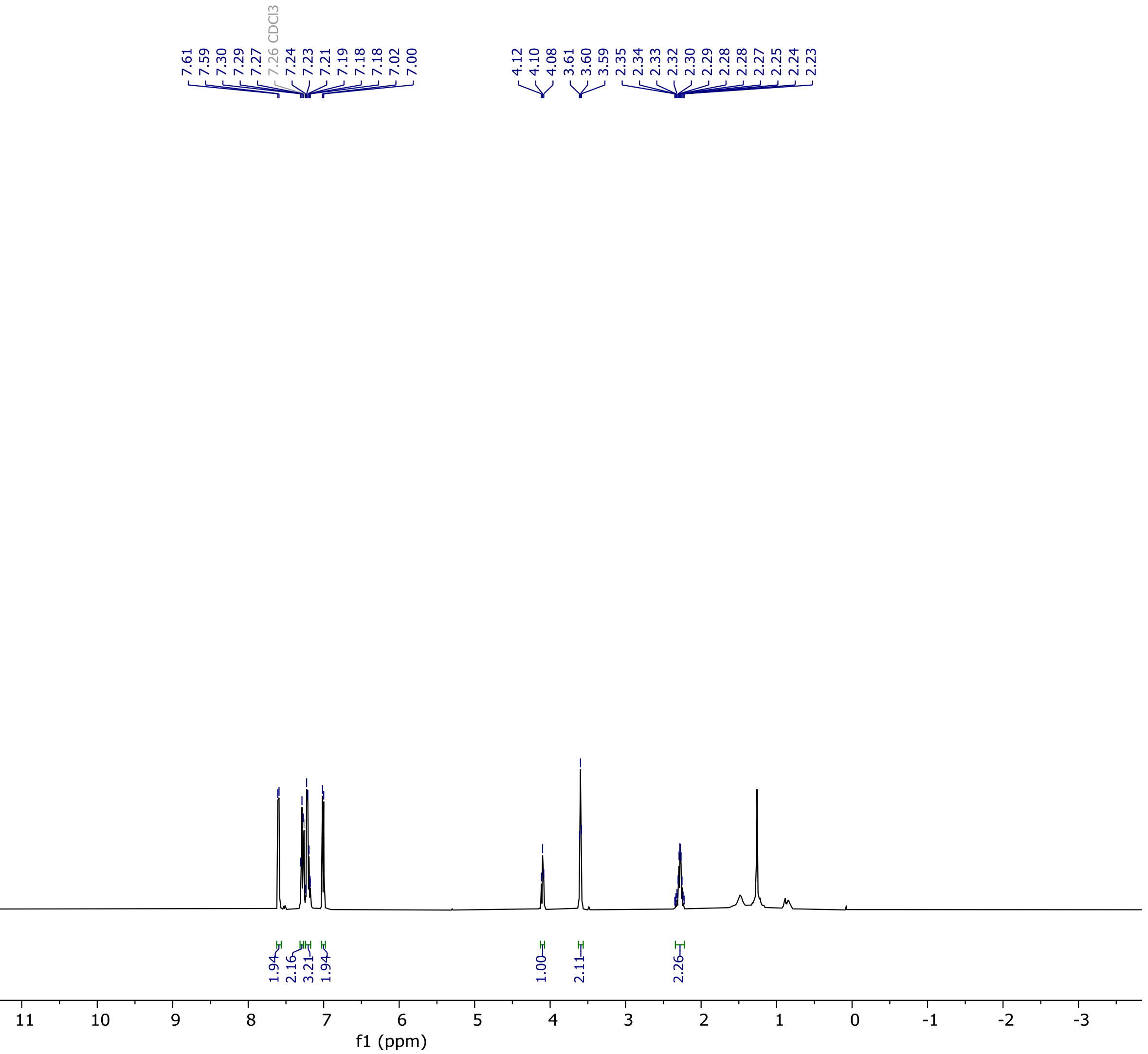

Compound 35 ^{13}C NMR in $CDCl_3$, 298 K

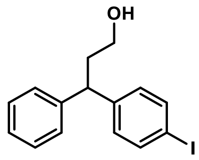

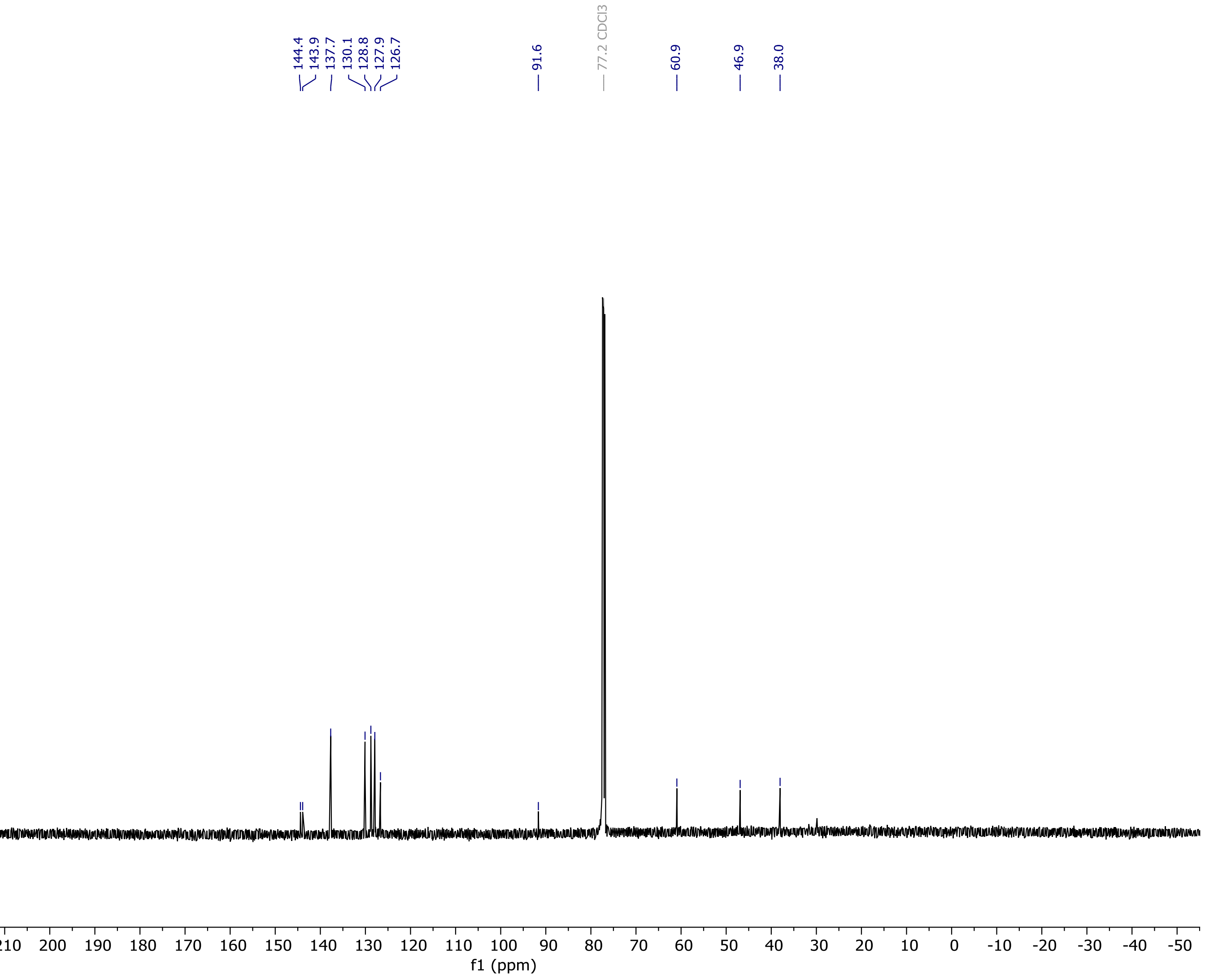

Compound 36 ^{1}H NMR in $CDCl_3$, 298 K

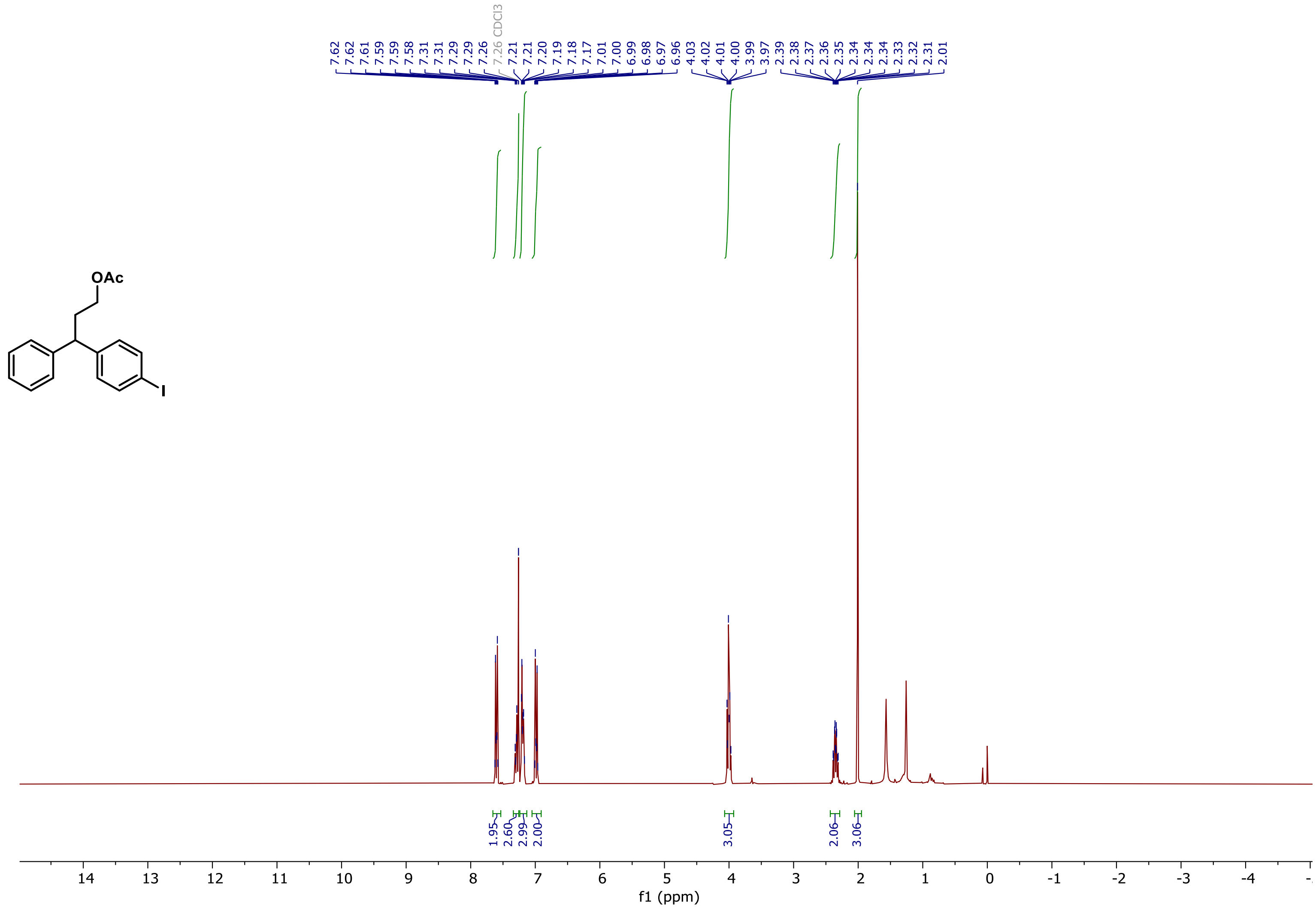

Compound 36 ^{13}C NMR in $CDCl_3$, 298 K

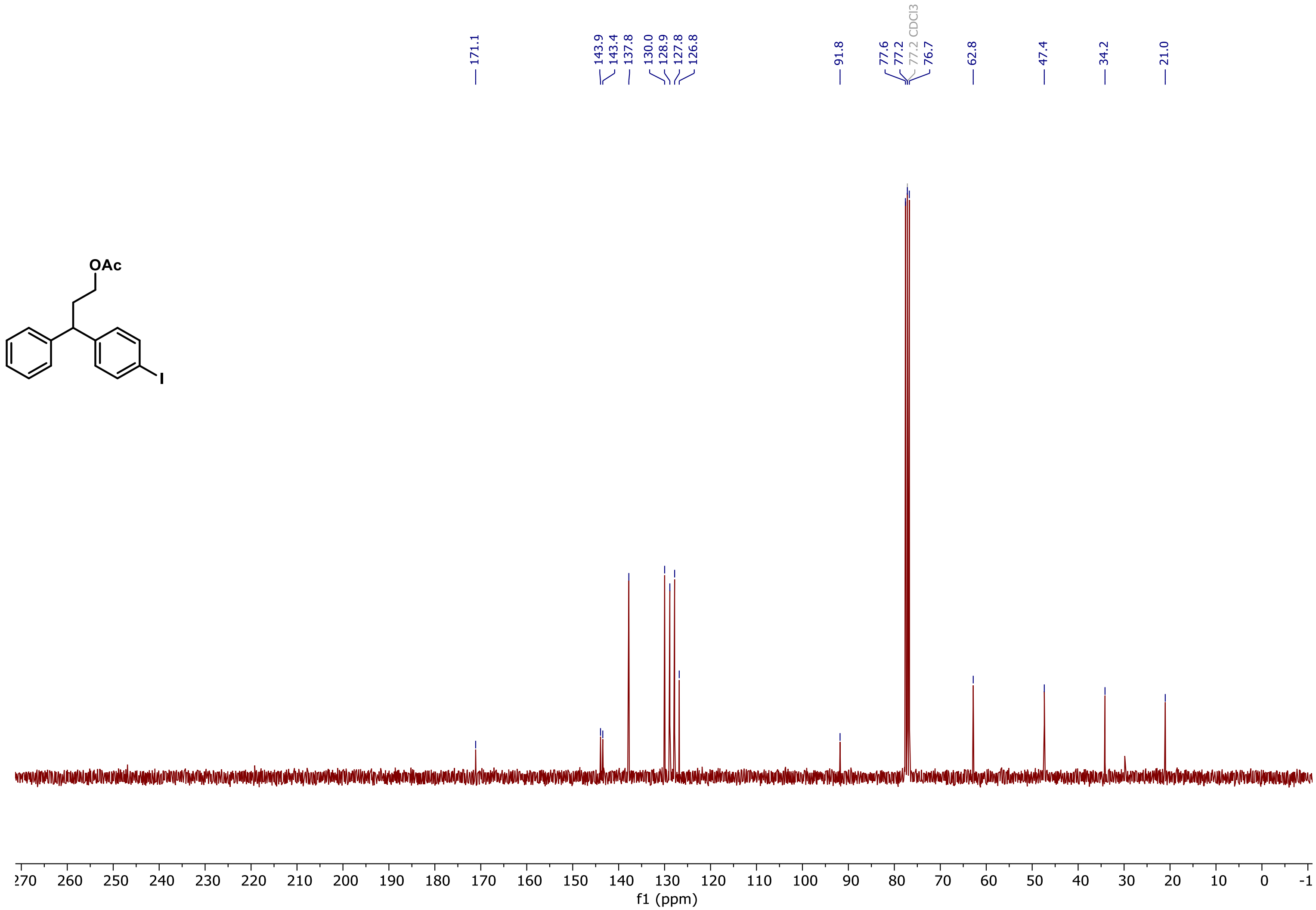

Compound 37 ^{1}H NMR in $CDCl_3$, 298 K

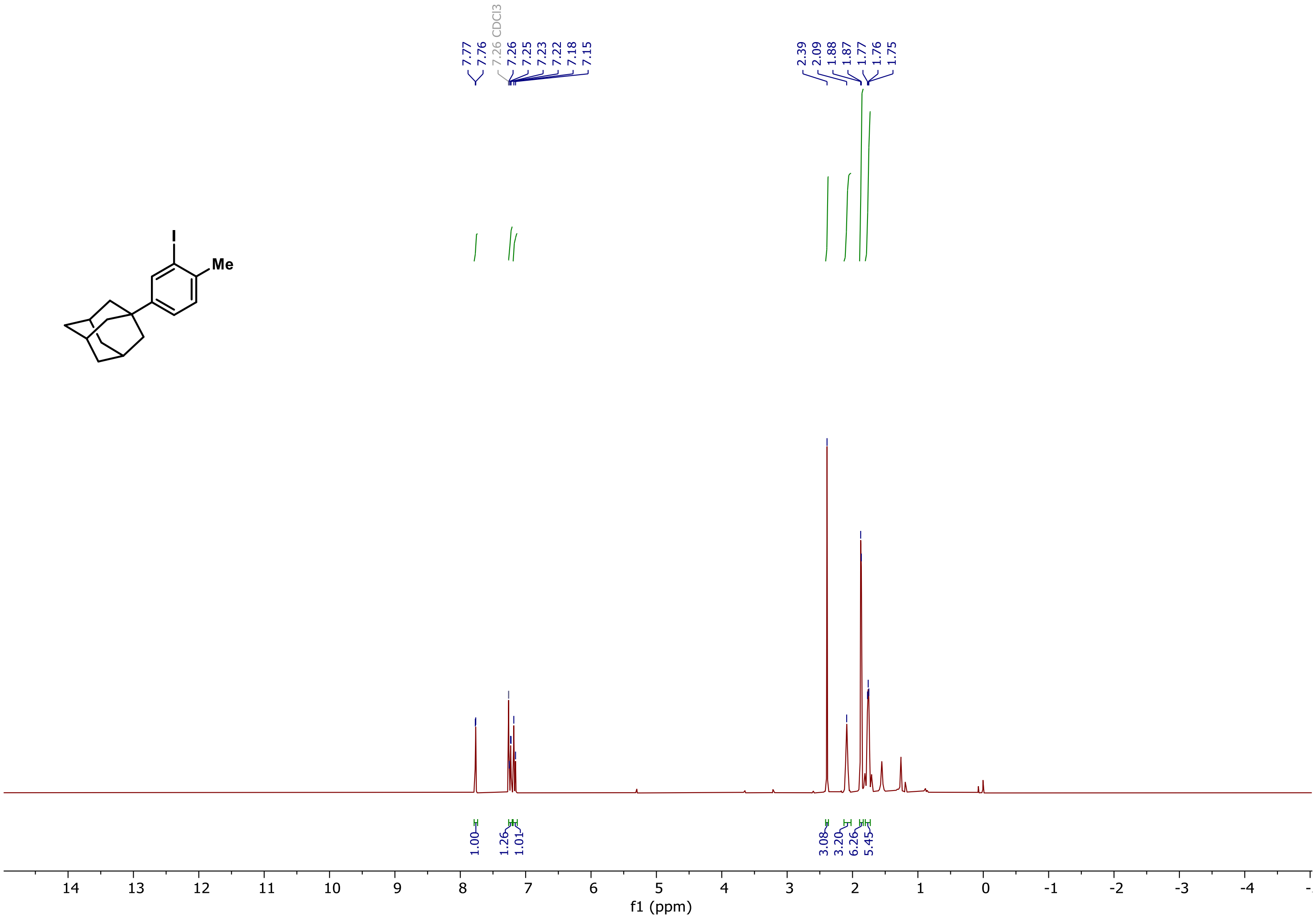

Compound 37 ^{13}C NMR in $CDCl_3$, 298 K

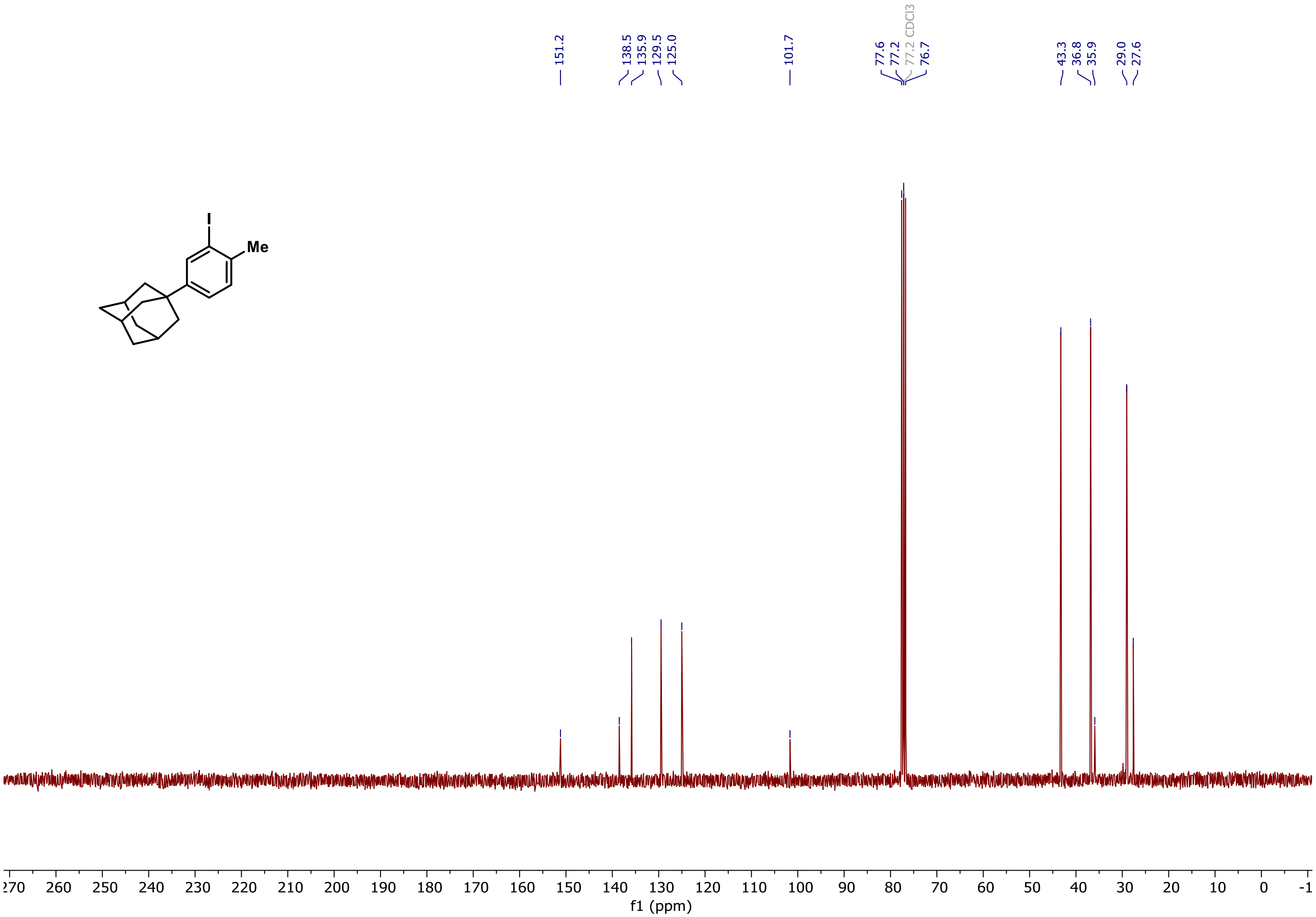

Compound 38 ^{1}H NMR in $CDCl_3$, 298 K

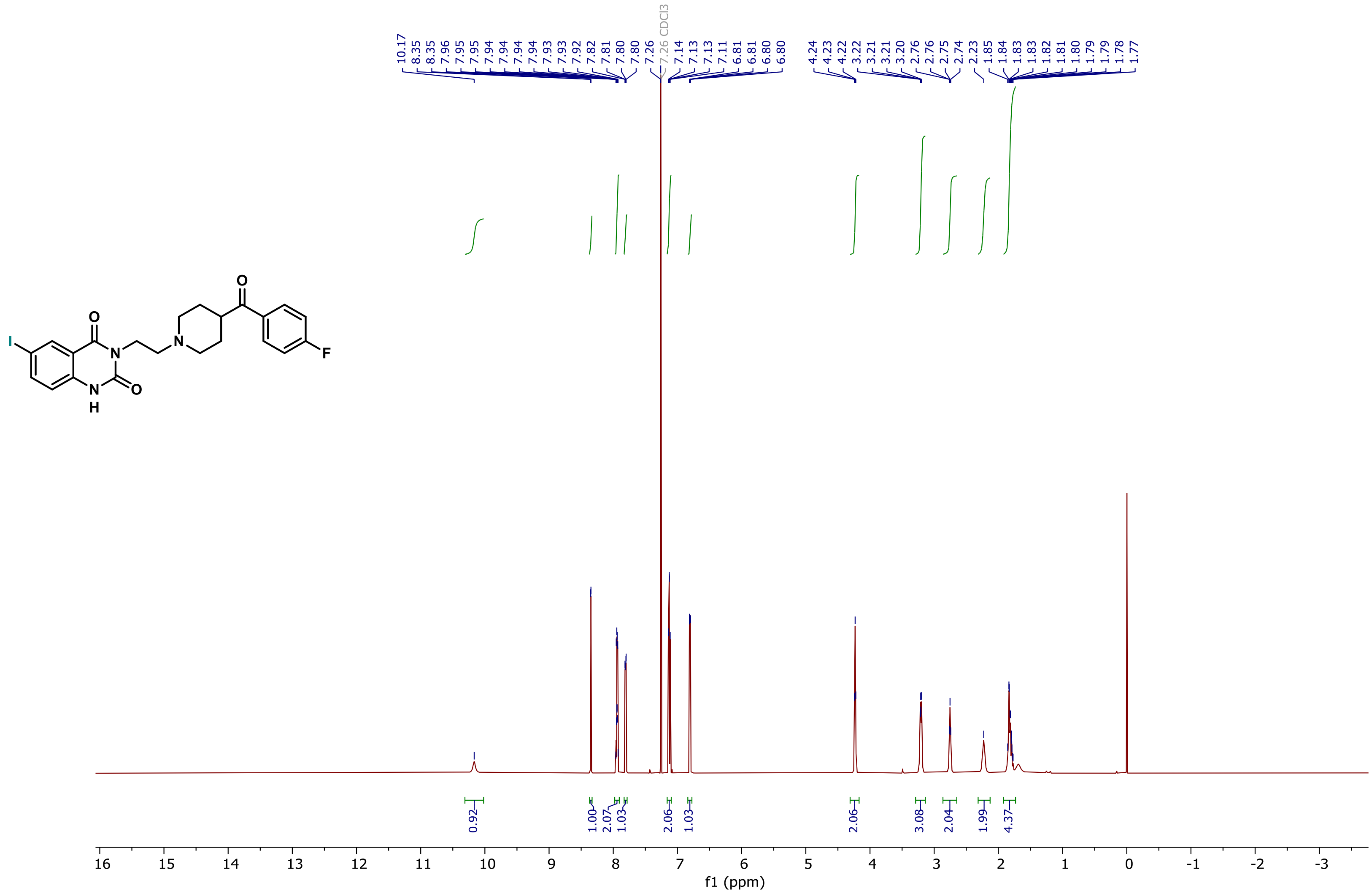

Compound 38 ^{13}C NMR in $CDCl_3$, 298 K

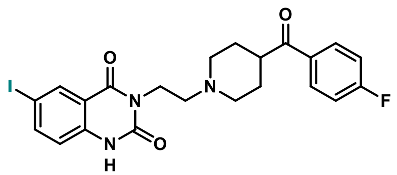

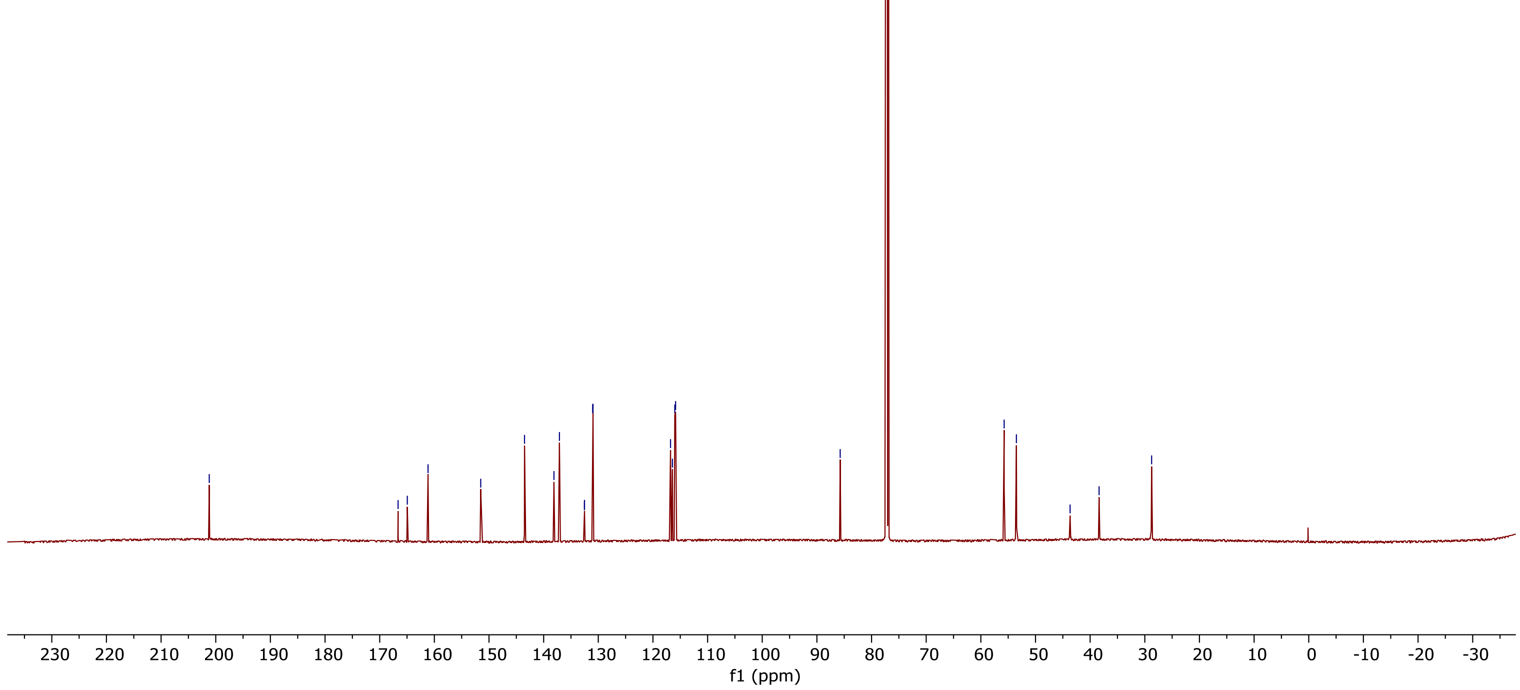

Compound 38 ^{19}F NMR in $CDCl_3$, 298 K

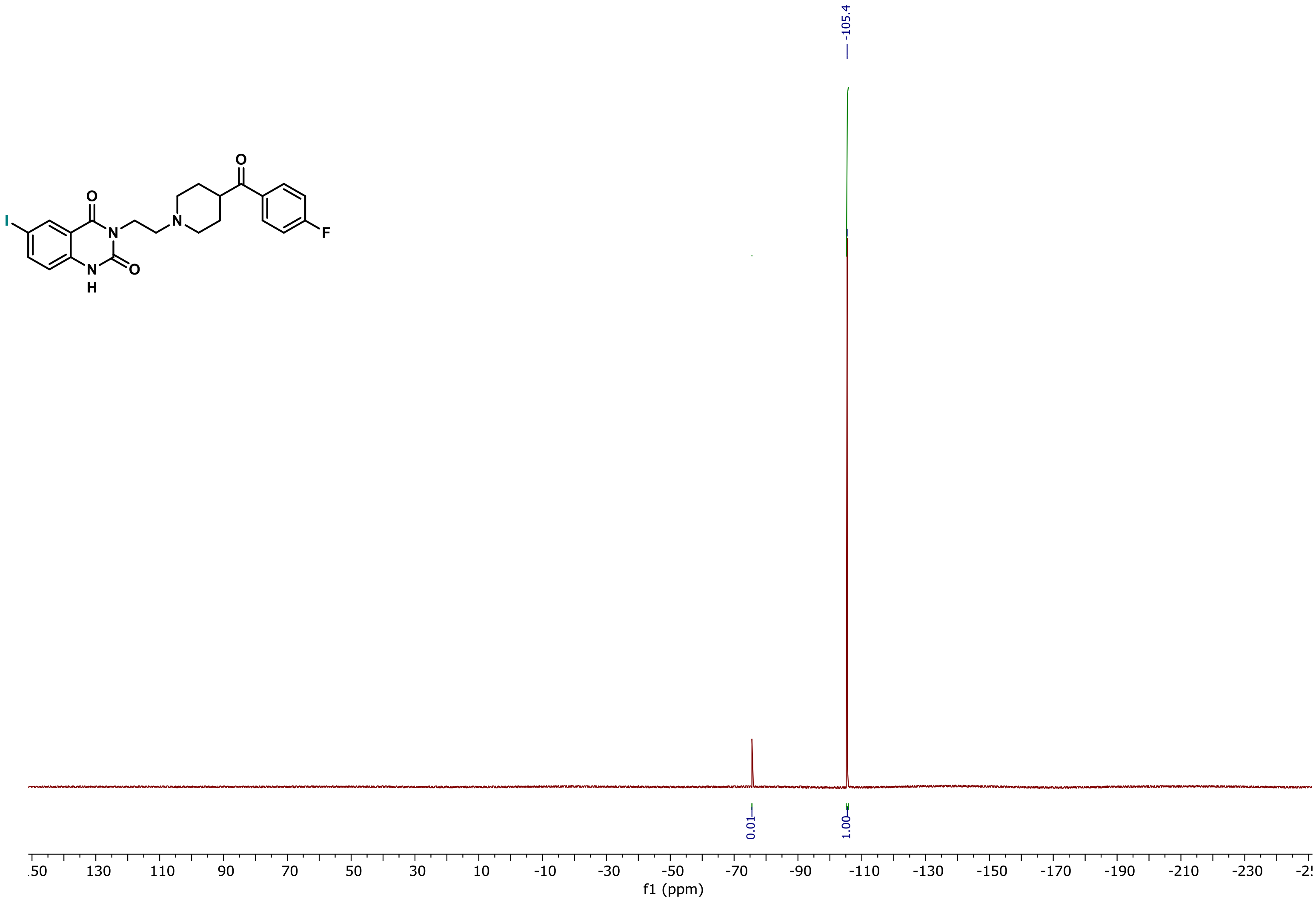

Compound 39 ^{1}H NMR in $CDCl_3$, 298 K

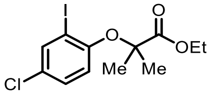

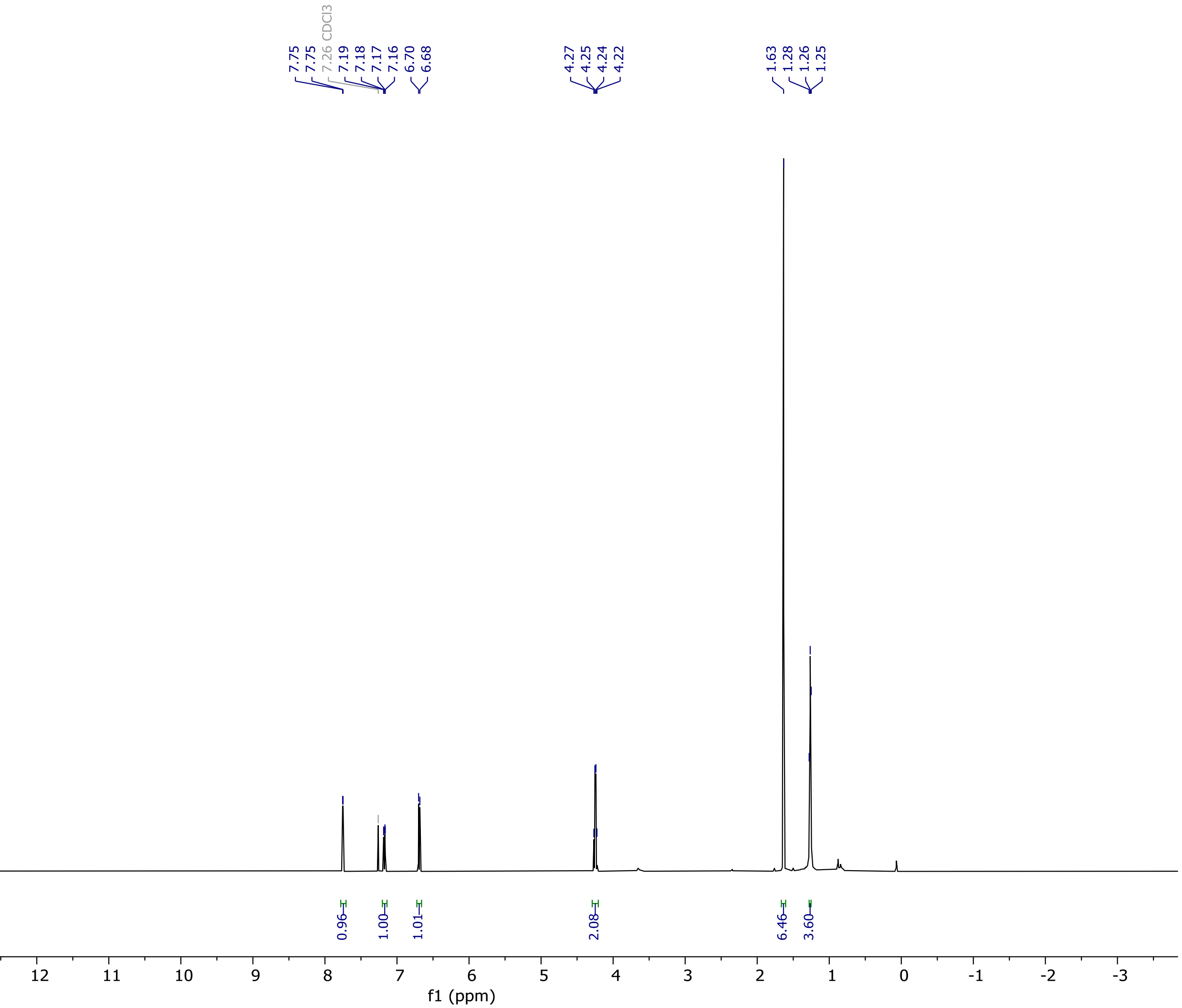

Compound 39 ^{13}C NMR in $CDCl_3$, 298 K

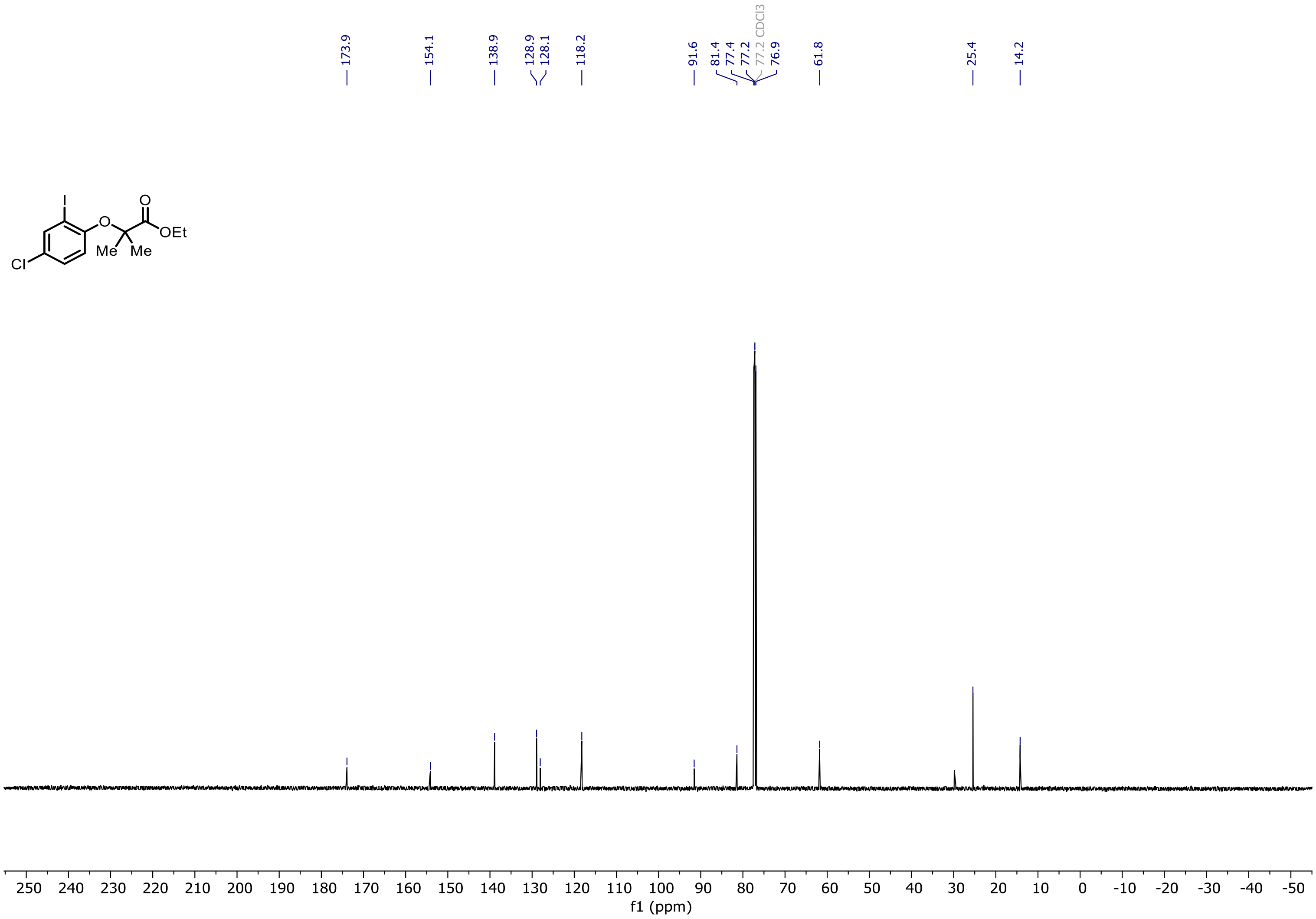

Compound 40 ^{1}H NMR in $CDCl_3$, 298 K

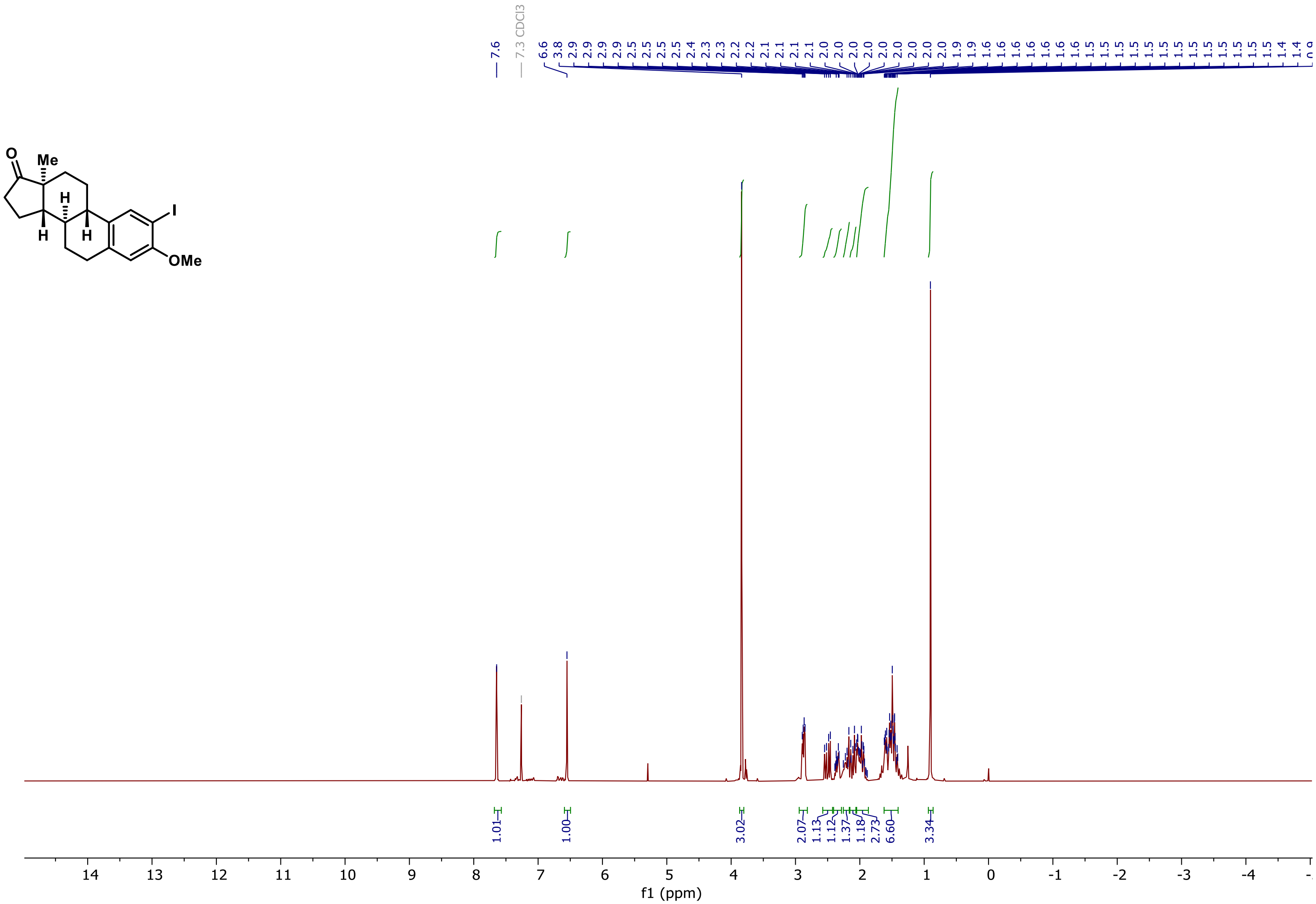

Compound 40 ^{13}C NMR in $CDCl_3$, 298 K

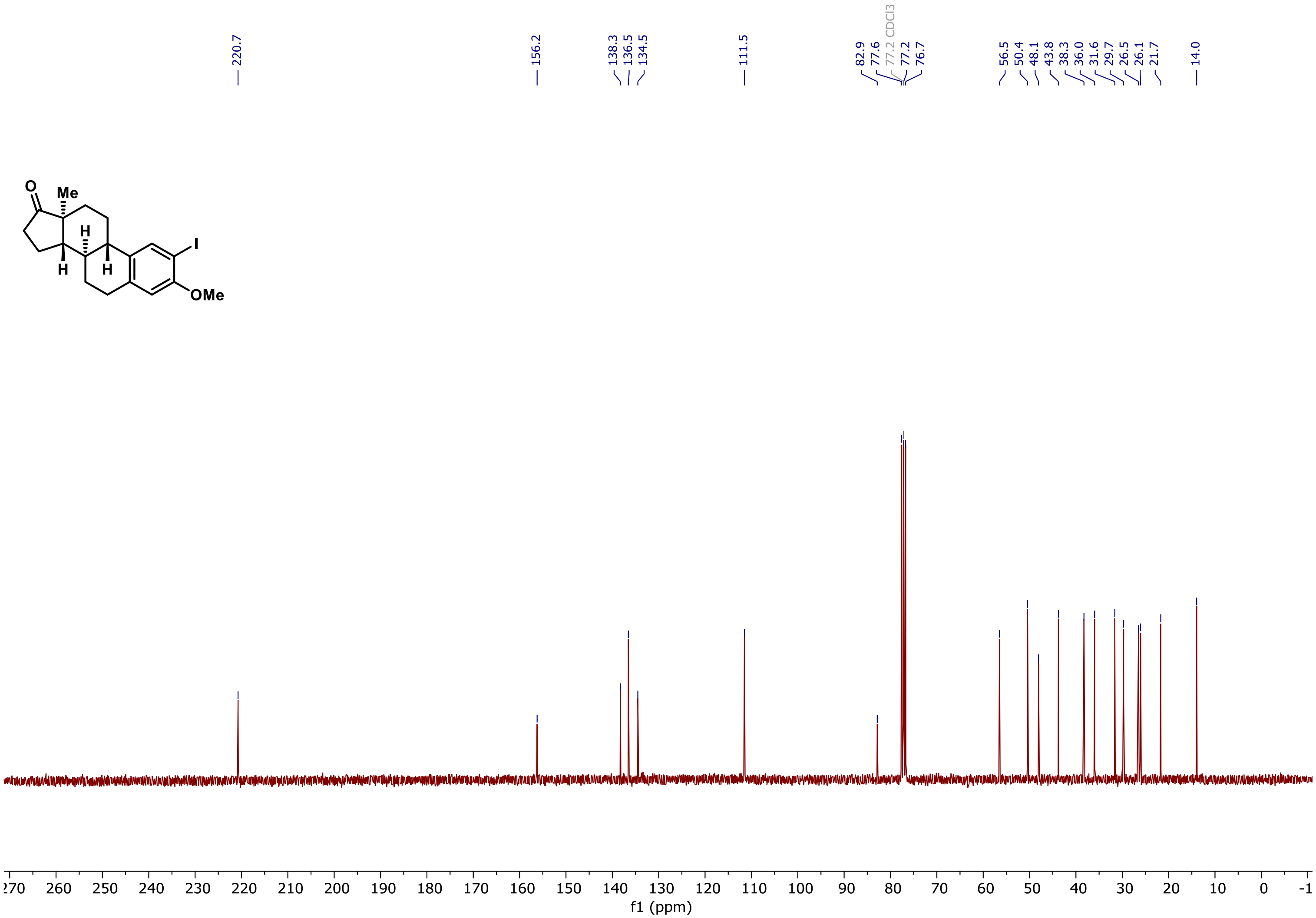

Compound 41 ^{1}H NMR in $CDCl_3$, 298 K

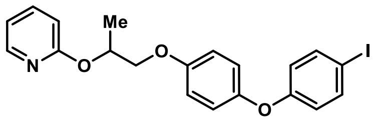

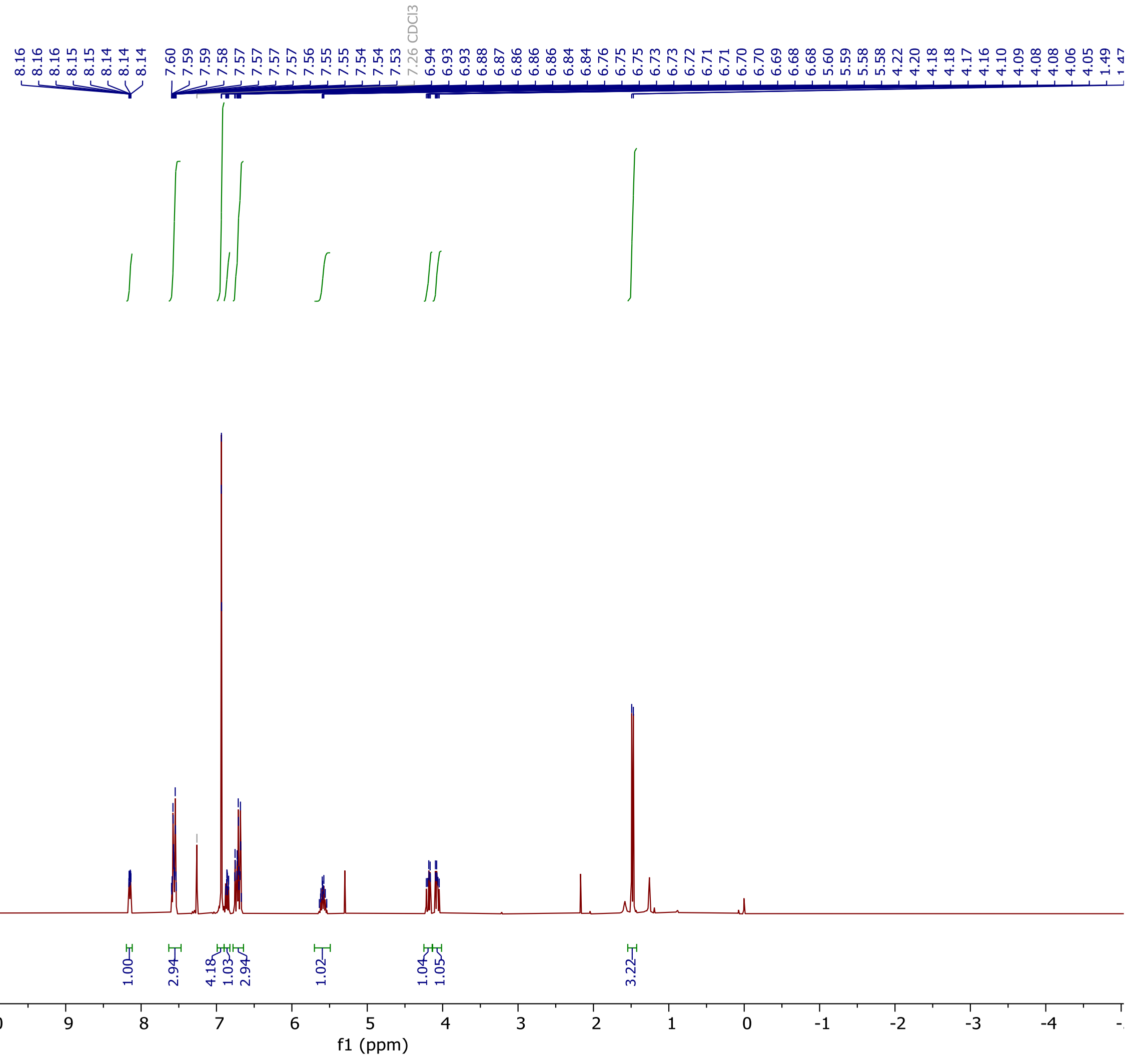

Compound 41 ^{13}C NMR in $CDCl_3$, 298 K

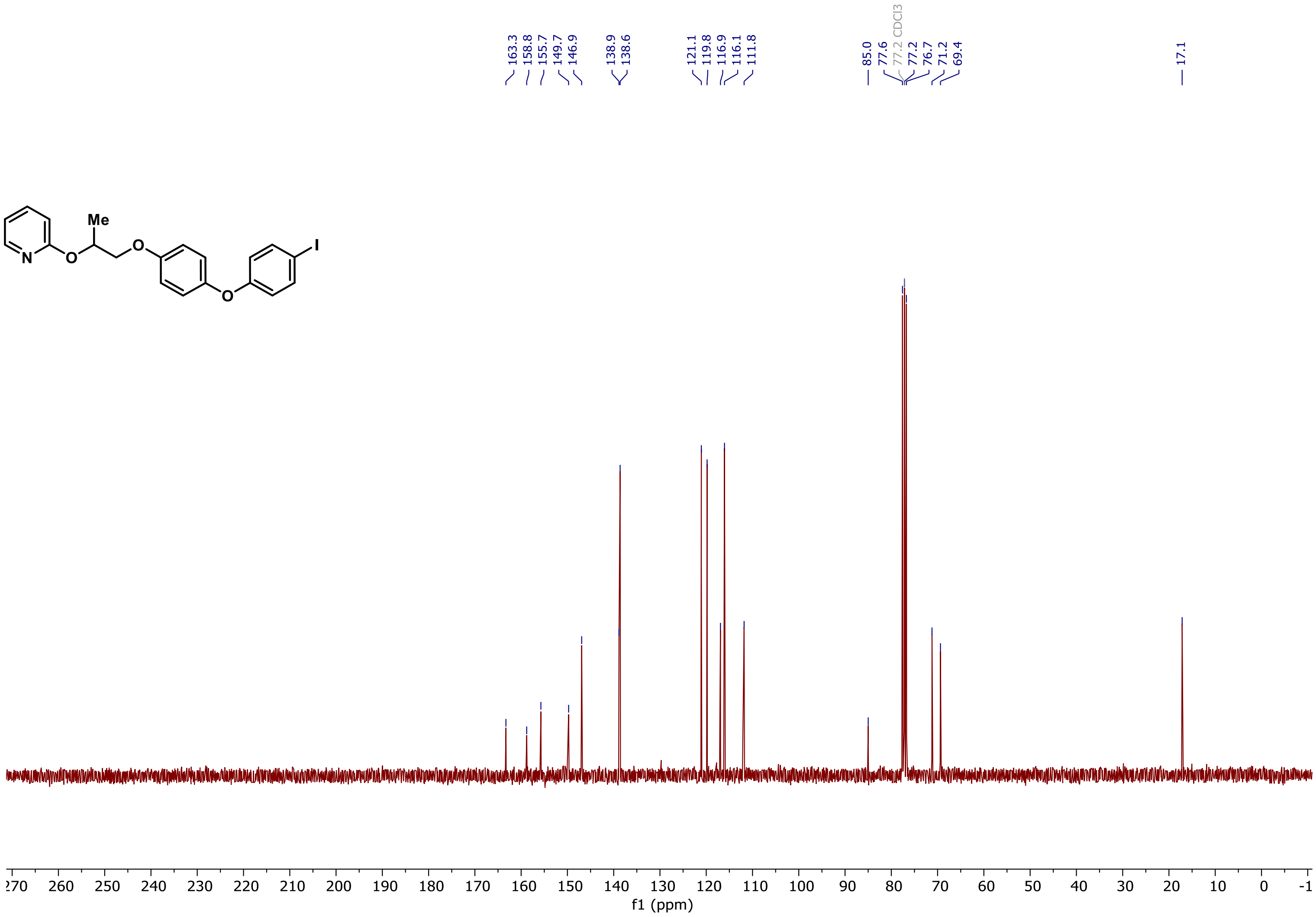

Compound 42 ^{1}H NMR in $CDCl_3$, 298 K

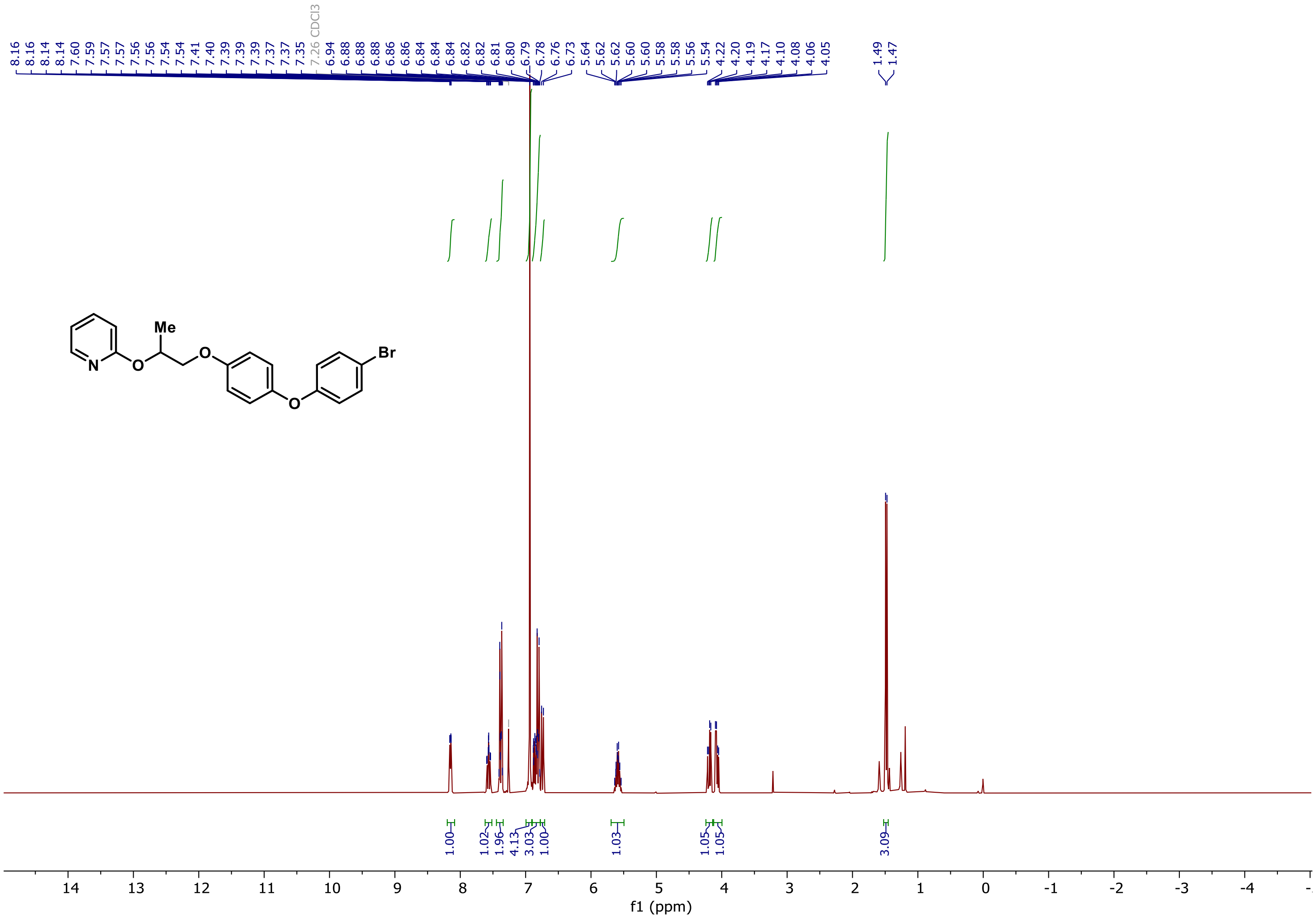

Compound 42 ^{13}C NMR in $CDCl_3$, 298 K

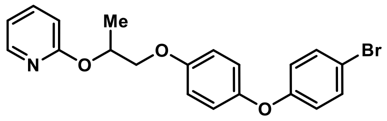

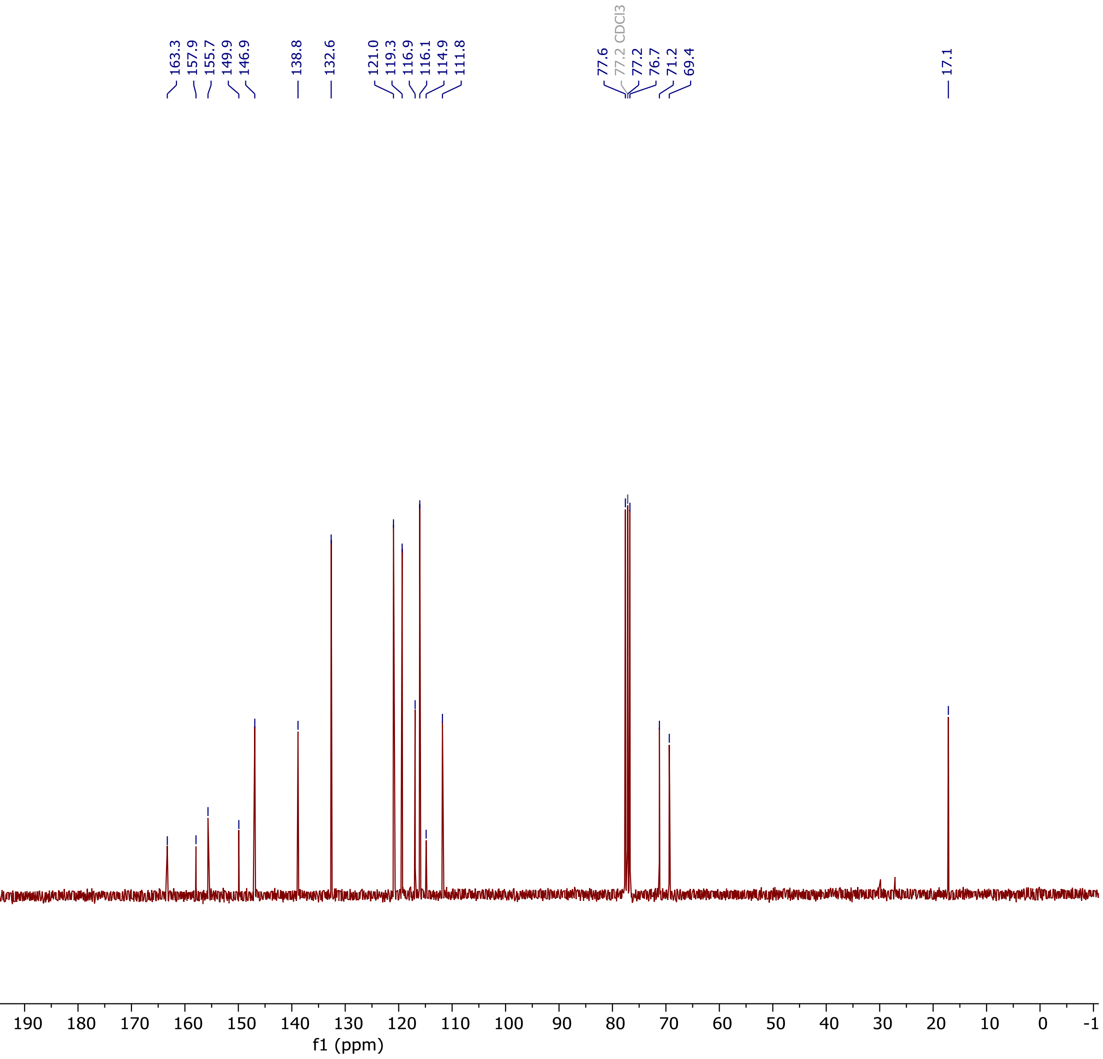

Compound 43 ¹H NMR in $CDCl_3$, 298 K

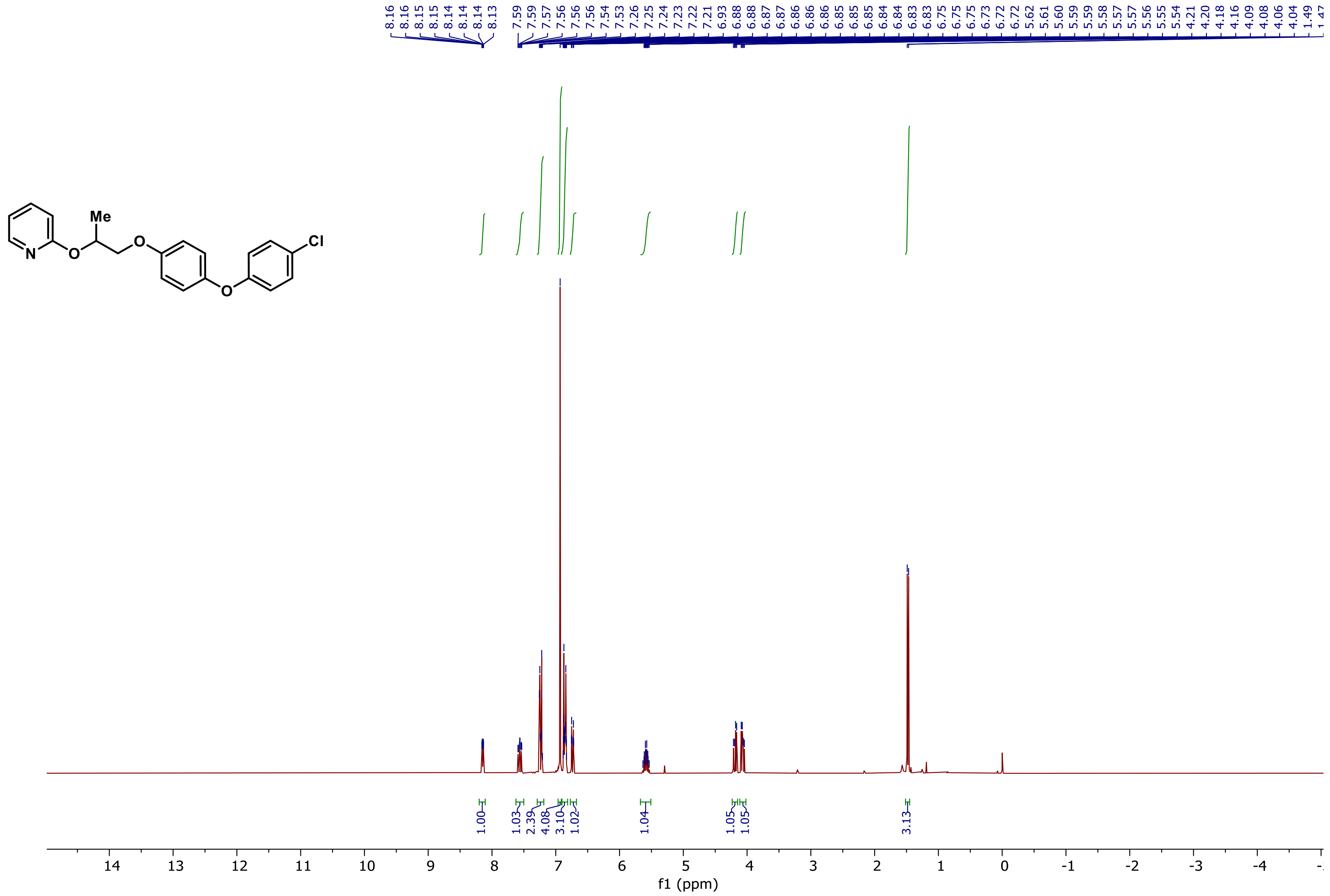

Compound 43 ^{13}C NMR in $CDCl_3$, 298 K

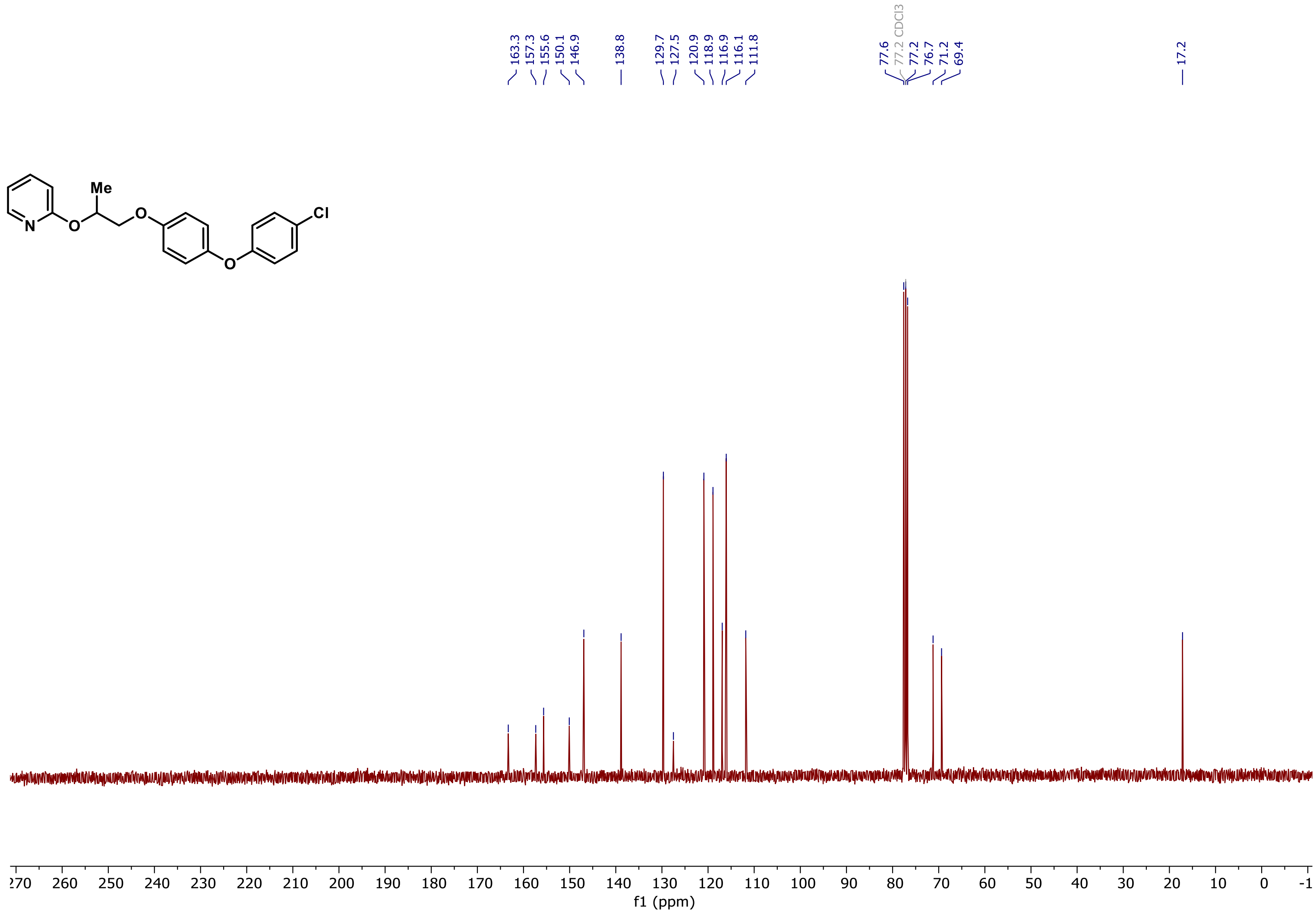

Compound 44 ^{1}H NMR in $CDCl_3$, 298 K

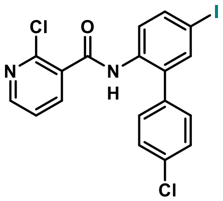

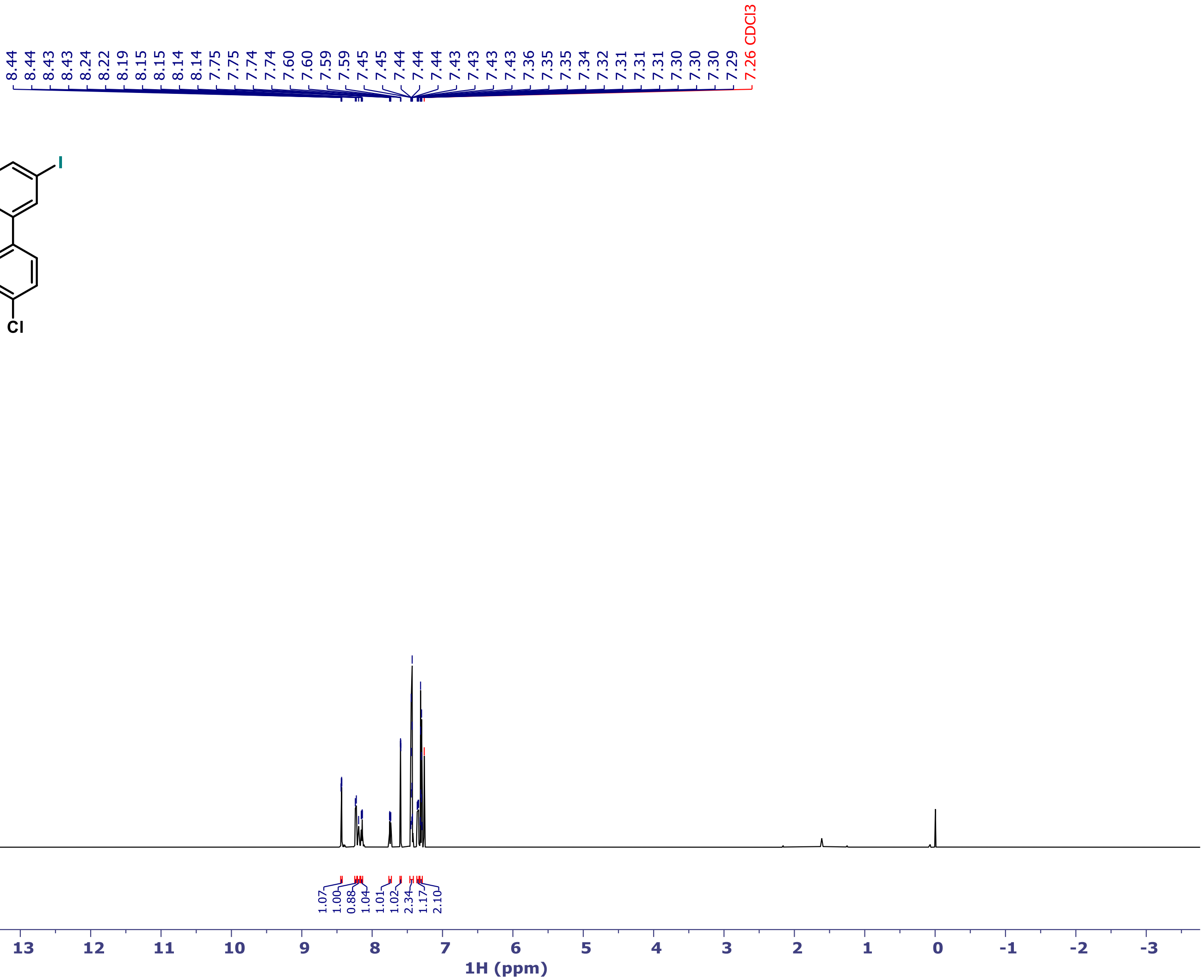

Compound 44 ^{13}C NMR in $CDCl_3$, 298 K

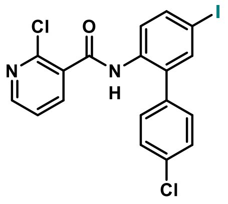

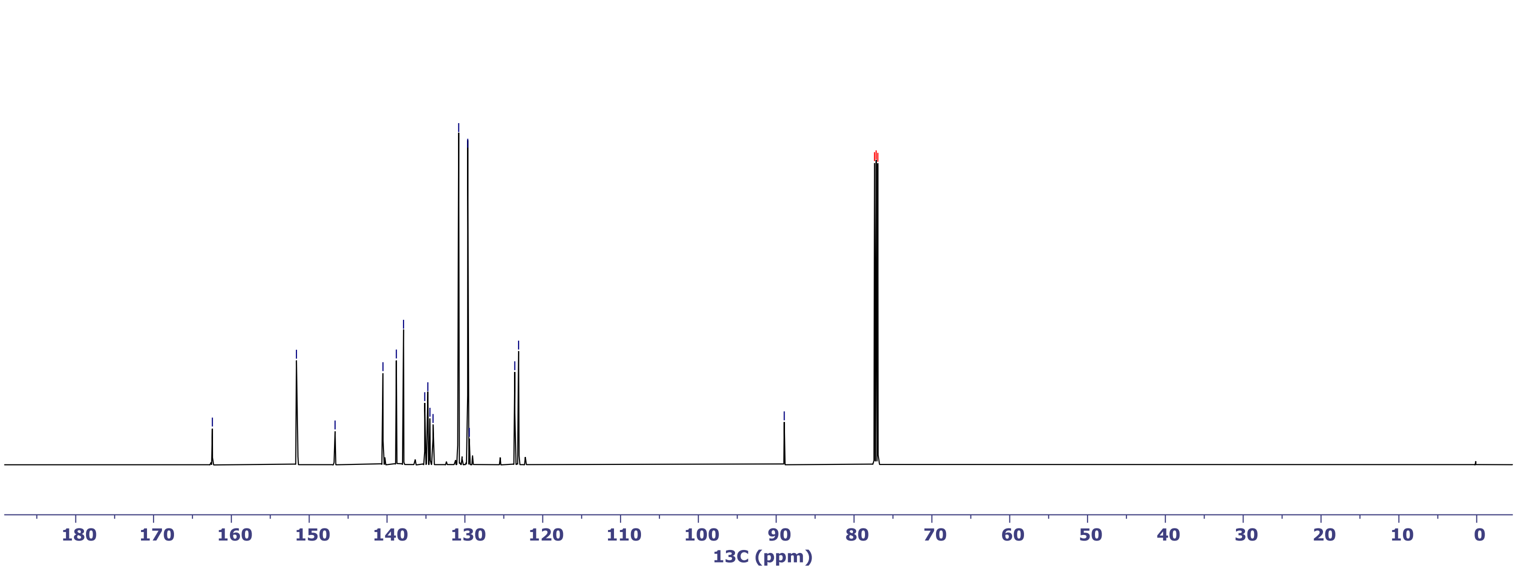

Compound 45 ¹H NMR in $CDCl_3$, 298 K

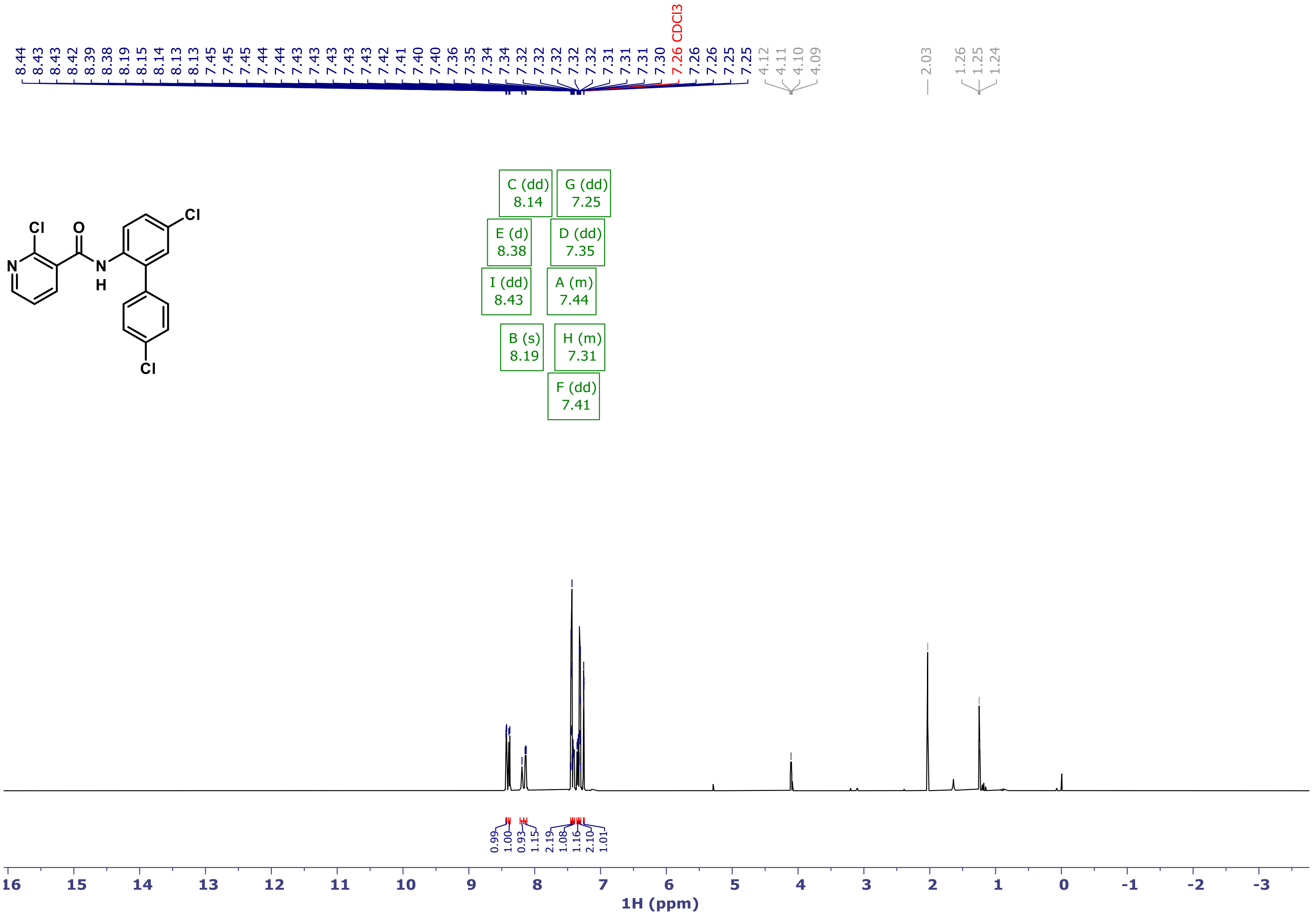

Compound 45 ^{13}C NMR in $CDCl_3$, 298 K

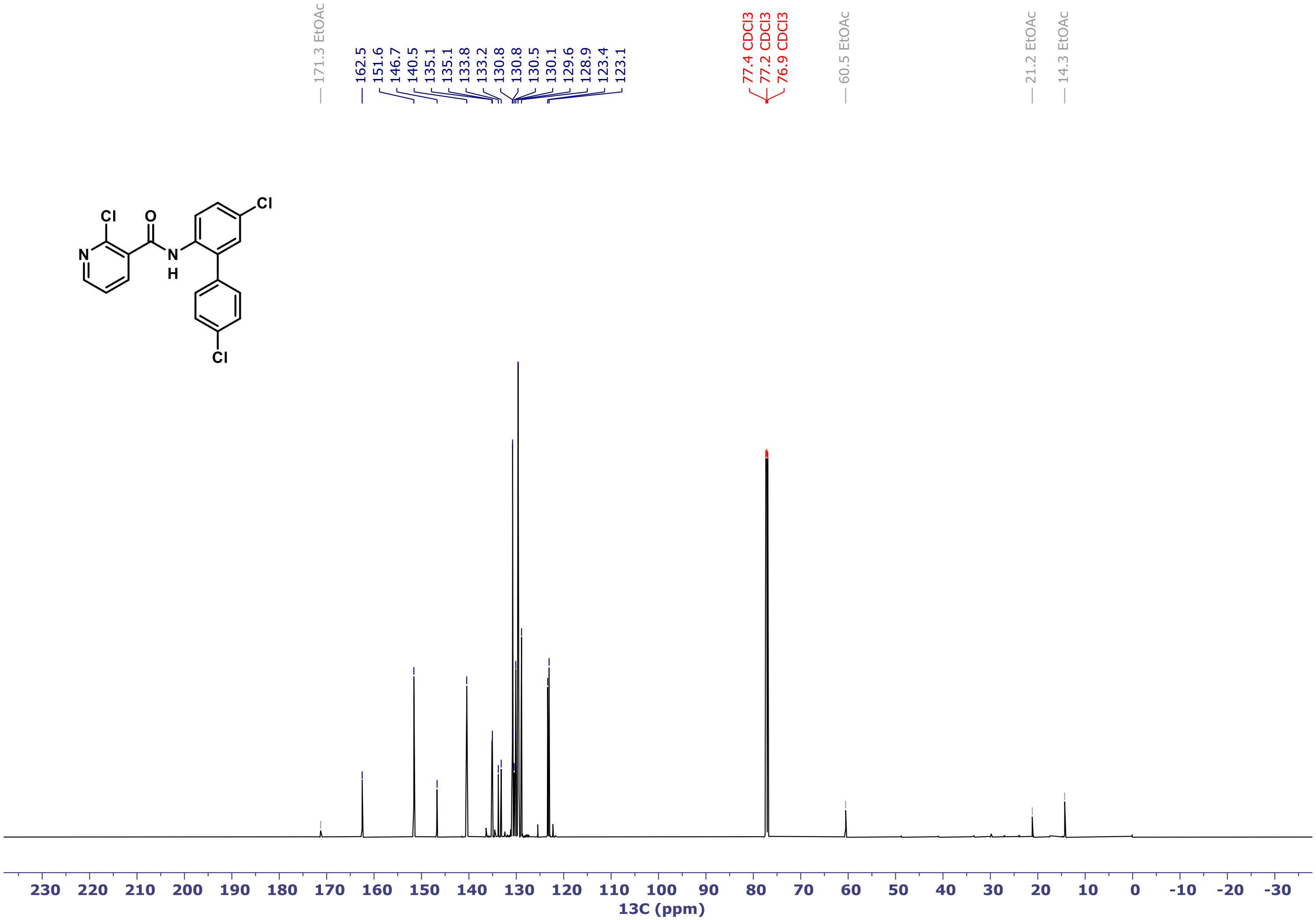

Compound 45 HSQC in $CDCl_3$, 298 K

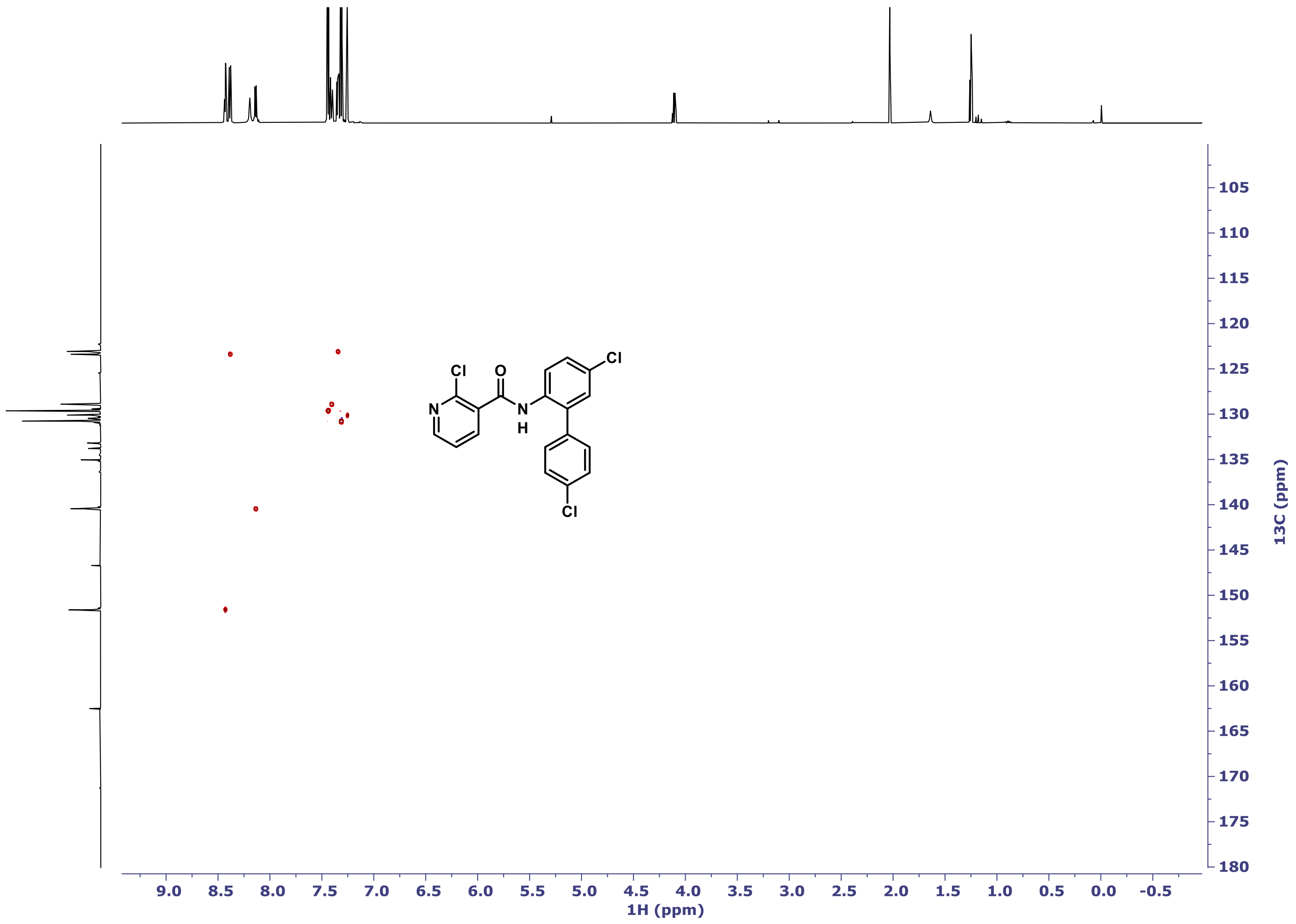

Compound 45 HMBC in $CDCl_3$, 298 K

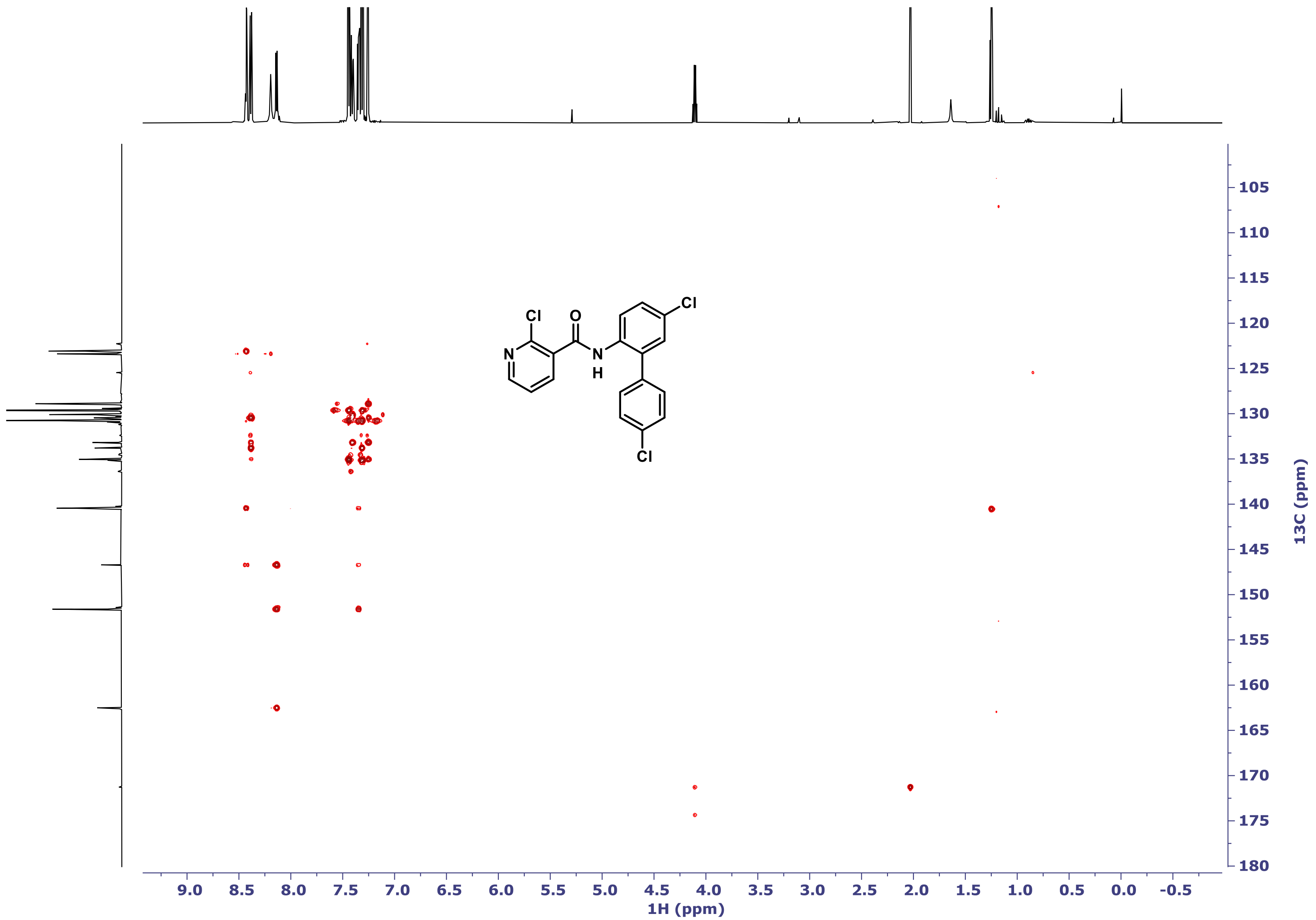

Compound 45 COSY in $CDCl_3$, 298 K

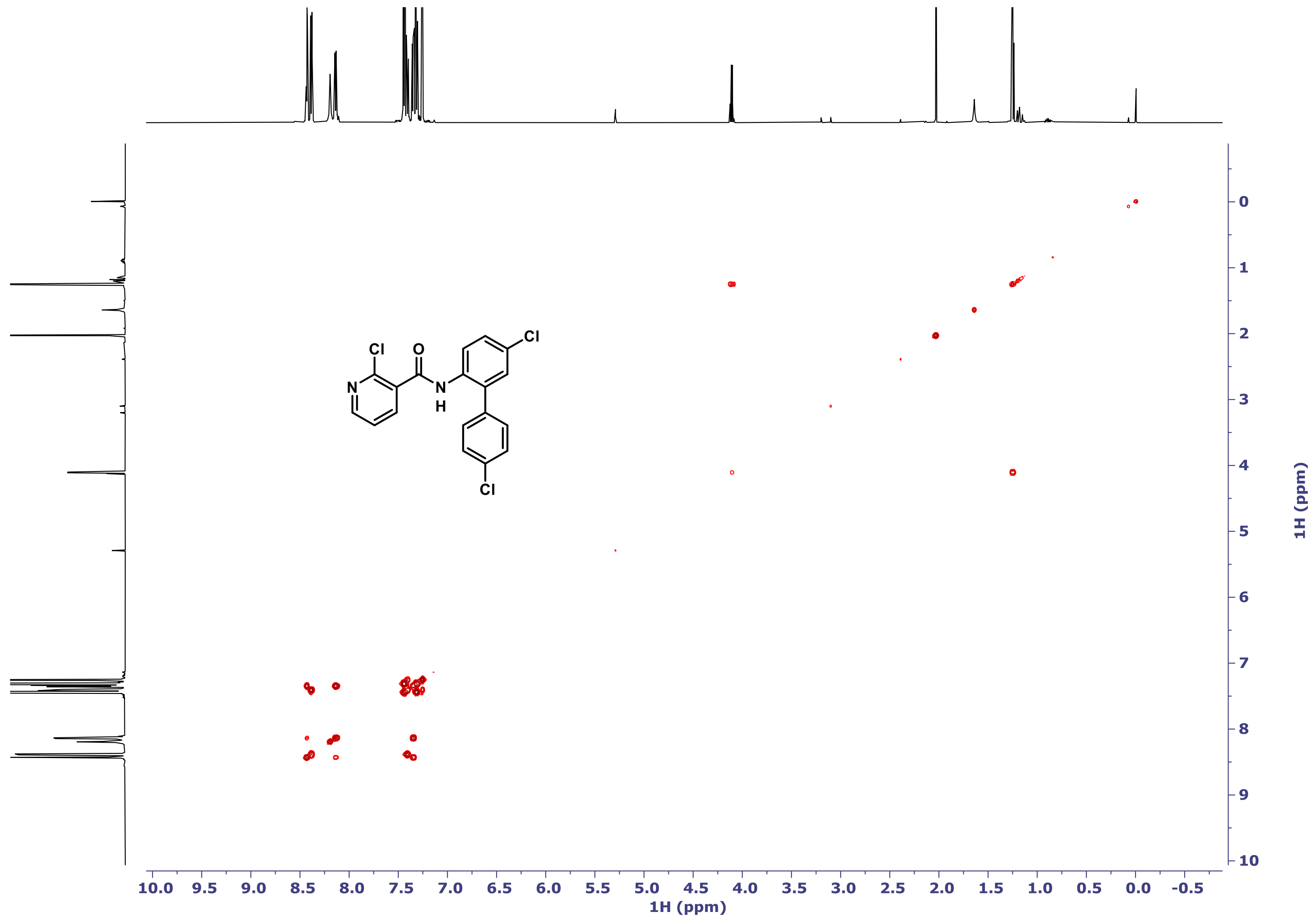

Compound 45 HMBC C-N in $CDCl_3$, 298 K

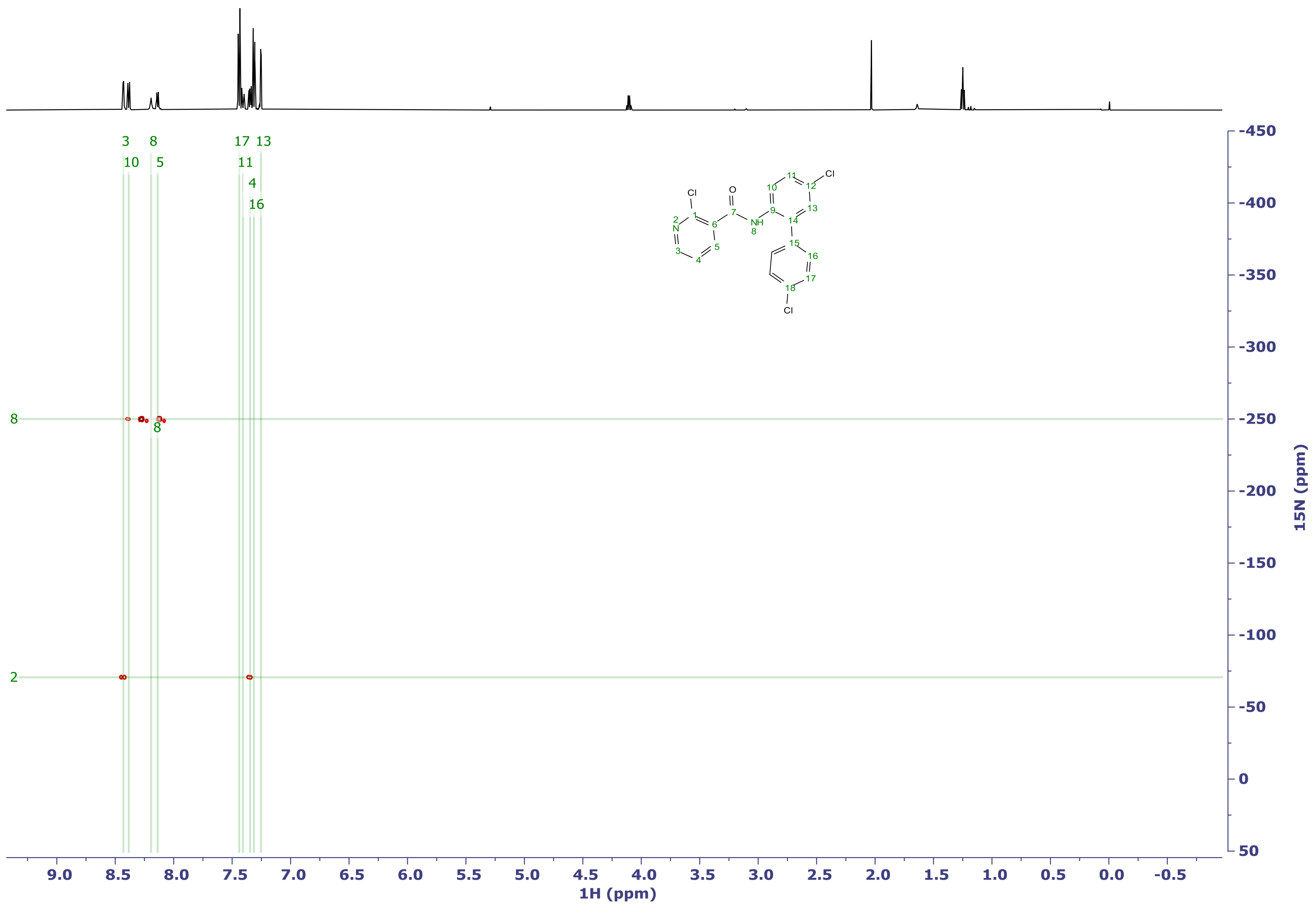

Compound 46 ^{1}H NMR in $CDCl_3$, 298 K

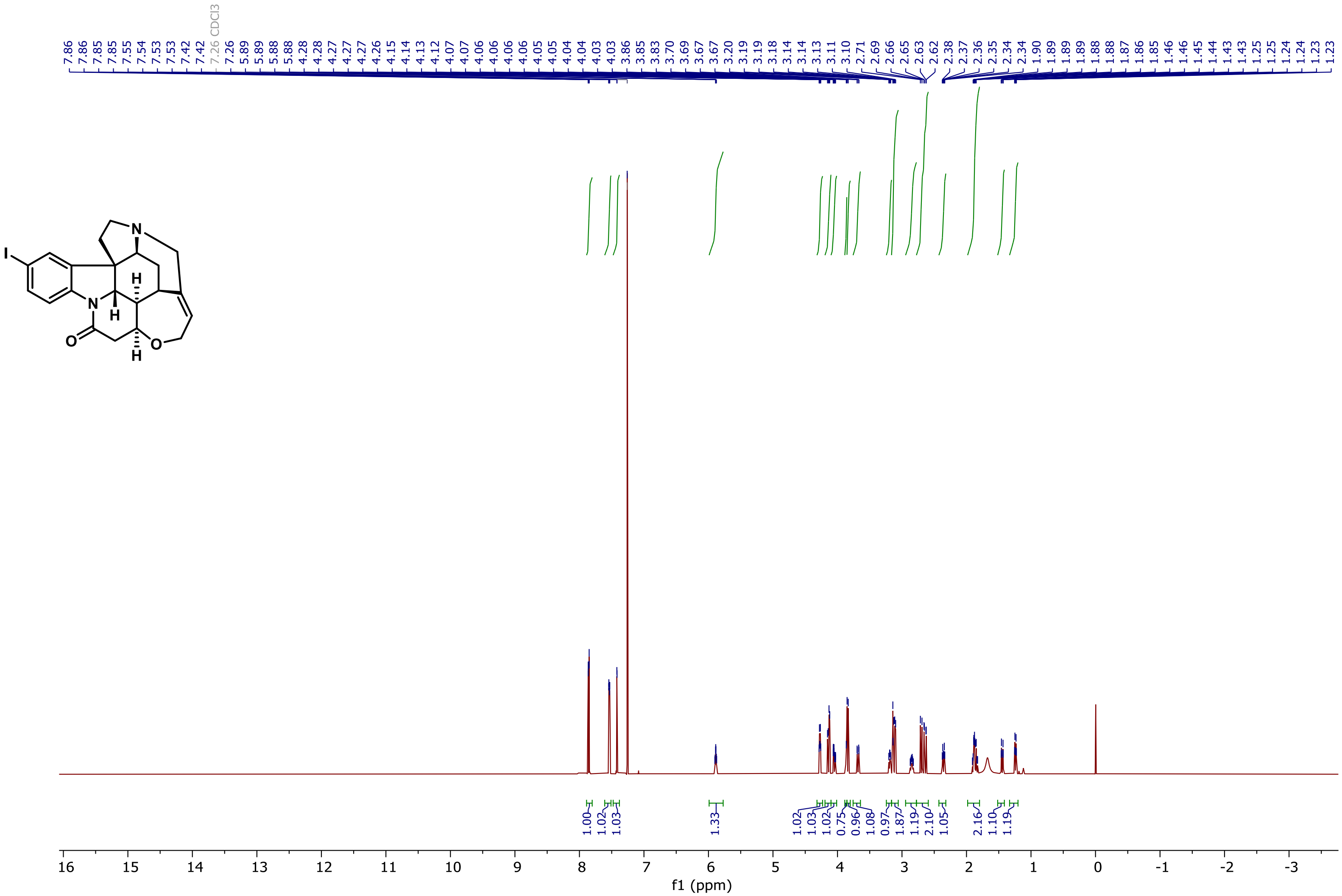

Compound 46 ^{13}C NMR in $CDCl_3$, 298 K

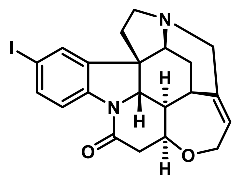

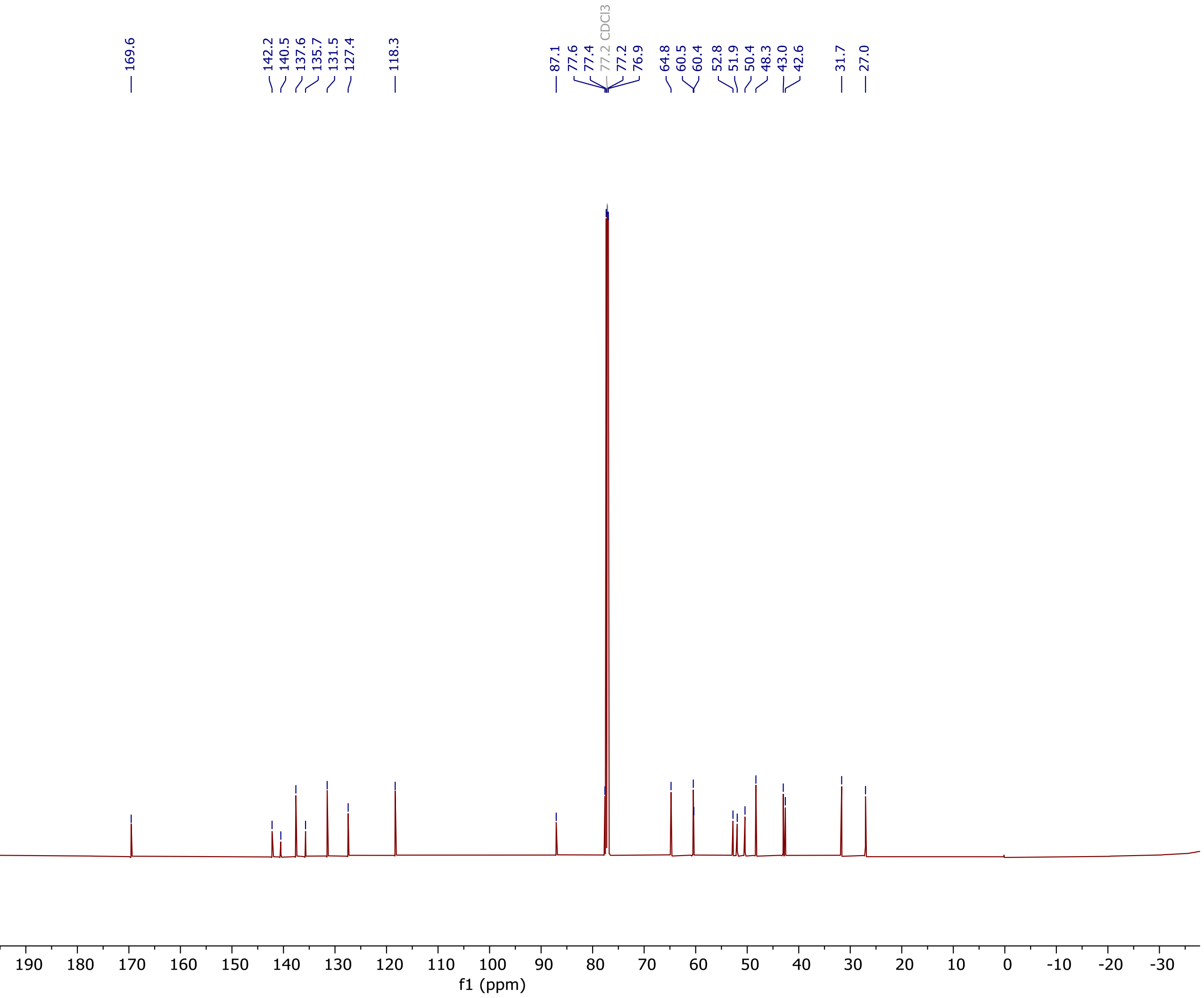

Compound 47 ^{1}H NMR in $CDCl_3$, 298 K

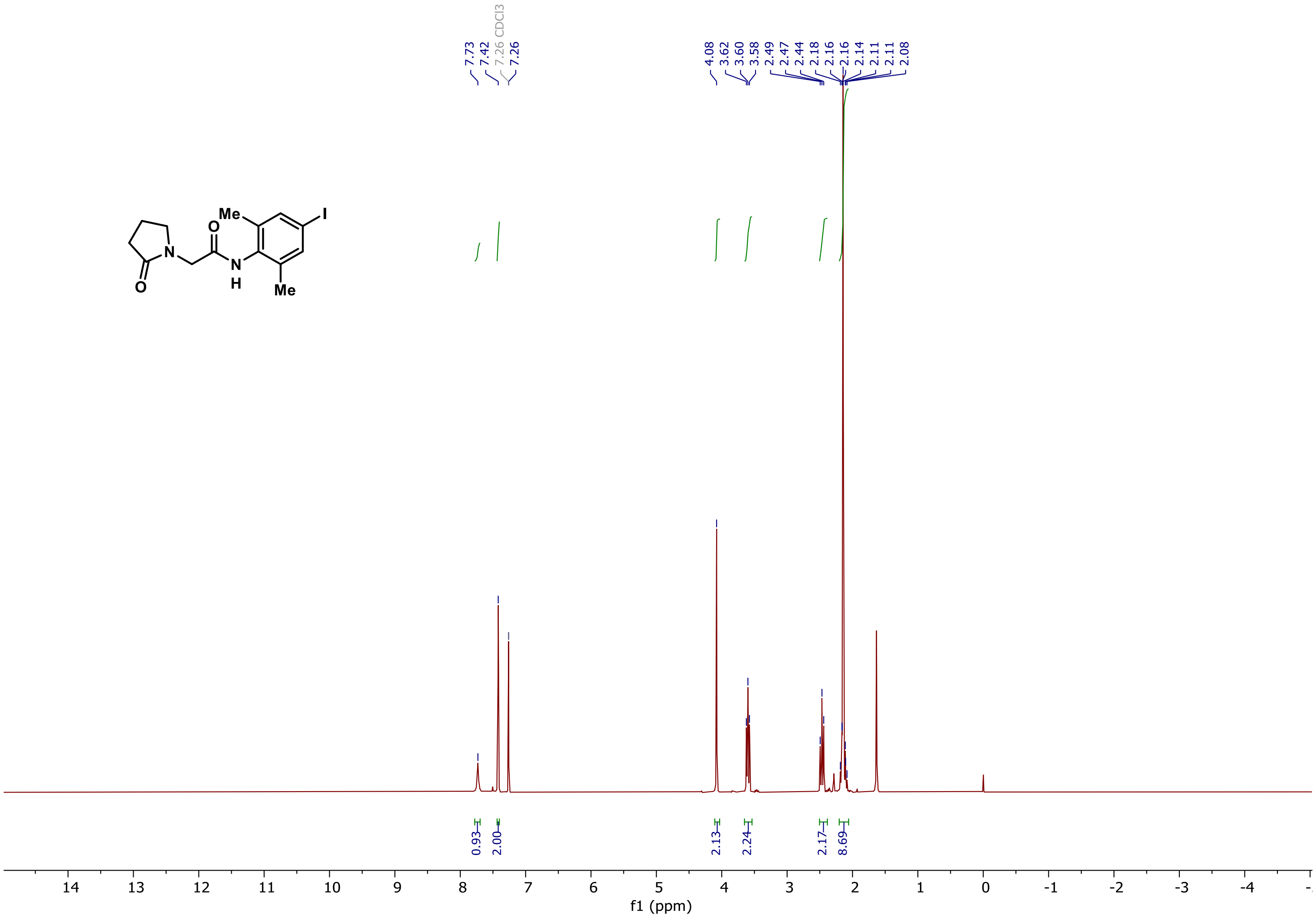

Compound 47 ^{13}C NMR in $CDCl_3$, 298 K

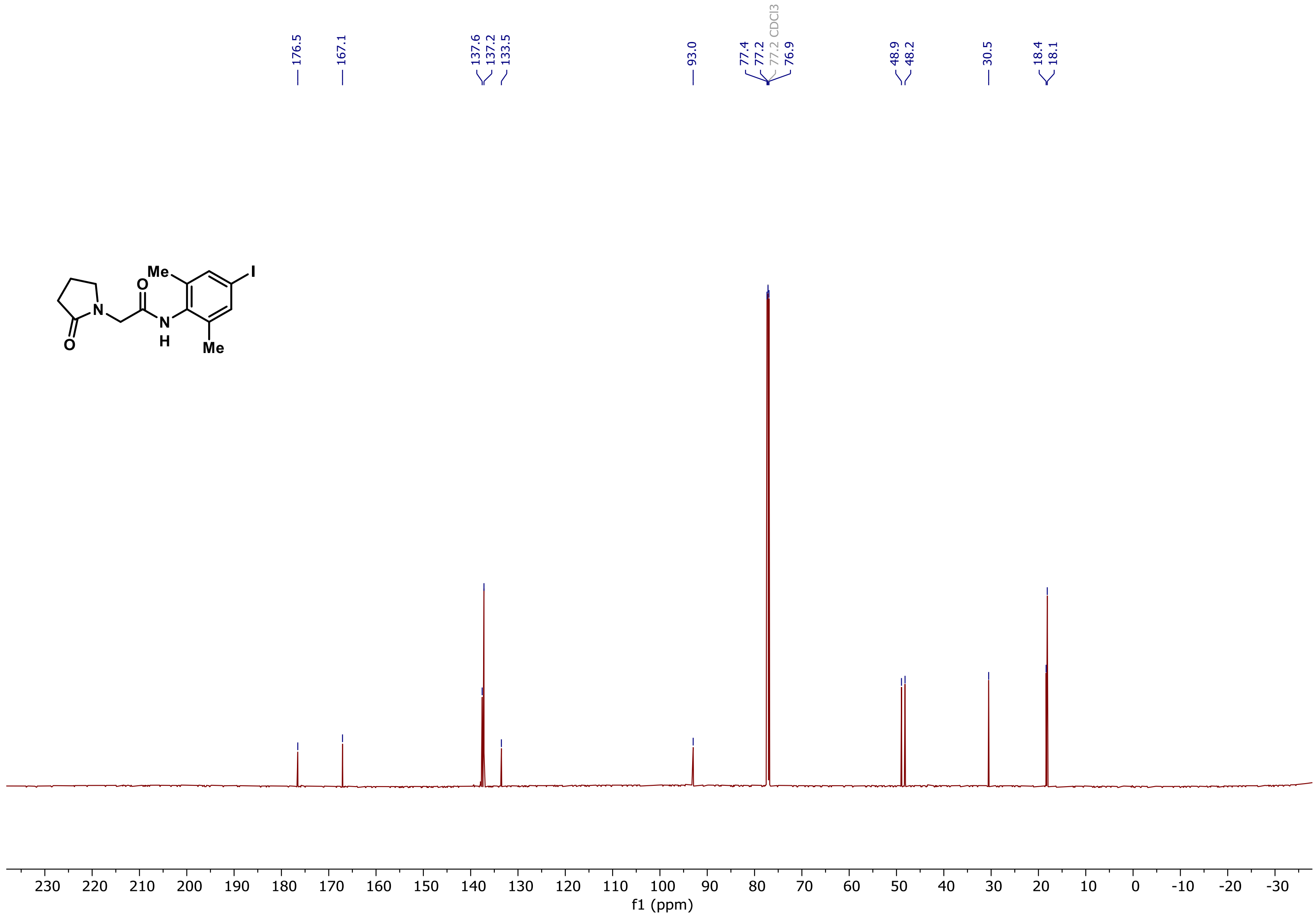

Compound 48 ^{1}H NMR in $CDCl_3$, 298 K

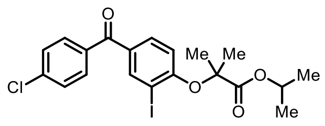

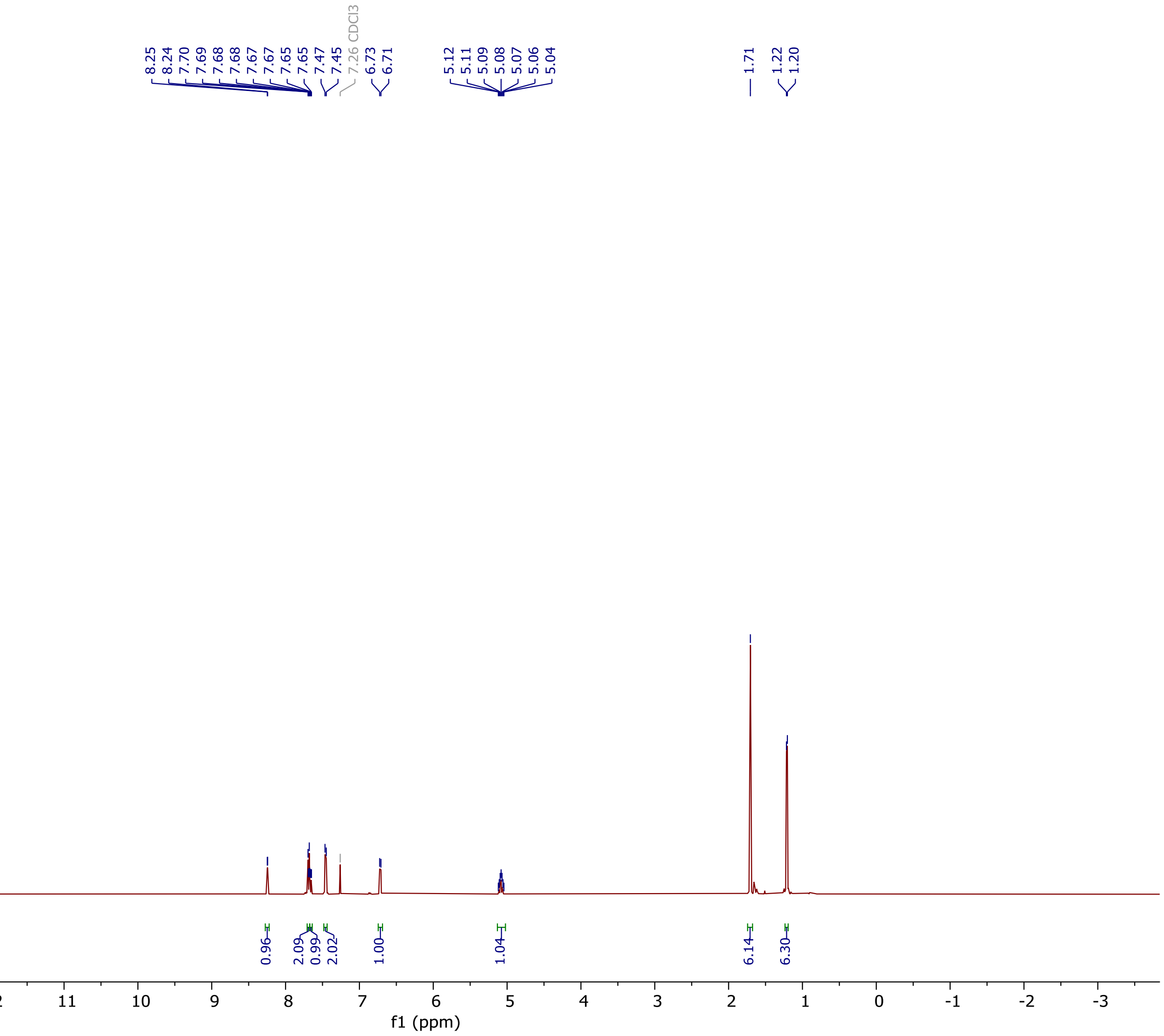

Compound 48 ^{13}C NMR in $CDCl_3$, 298 K

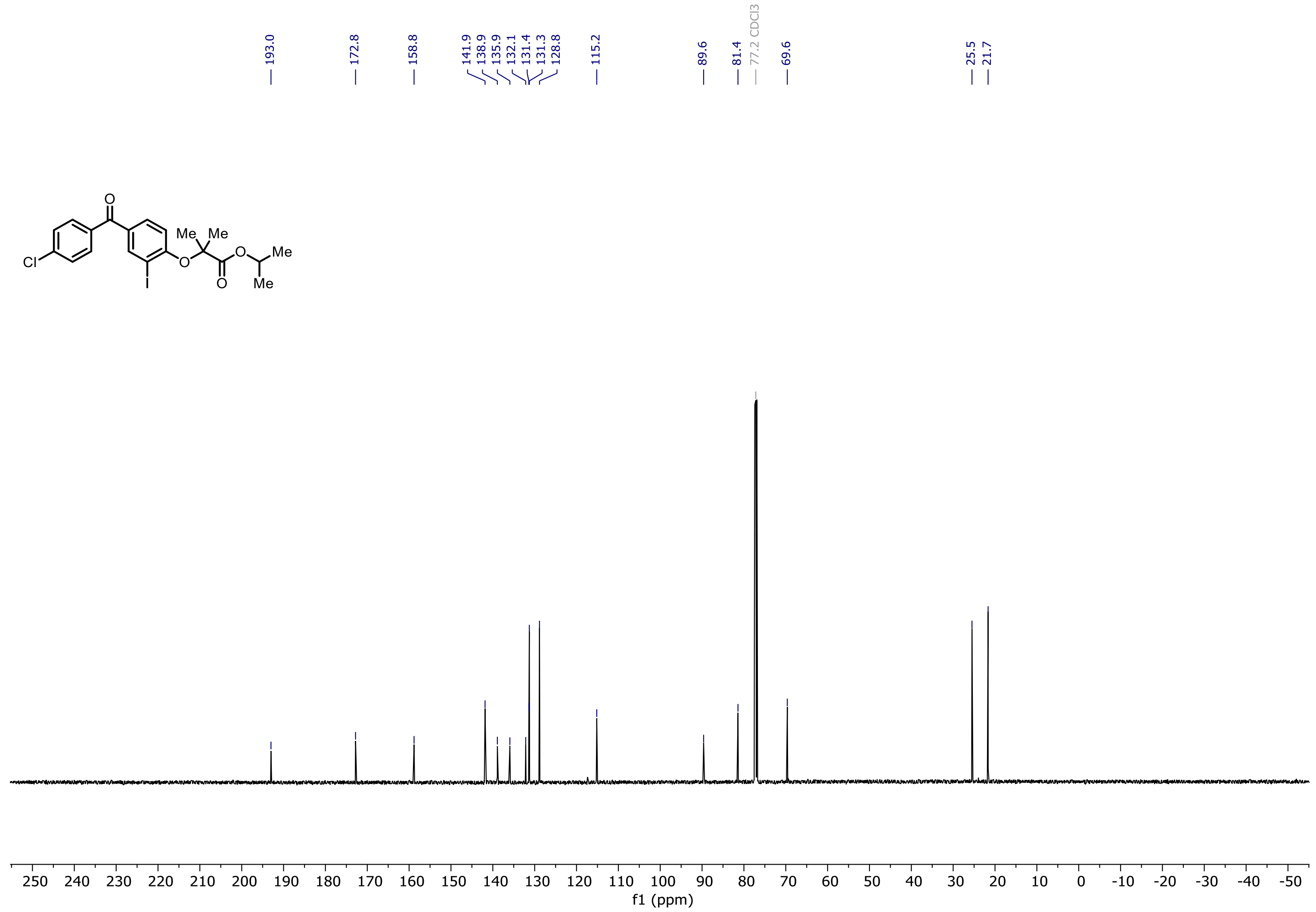

Compound 49 ^{1}H NMR in $CDCl_3$, 298 K

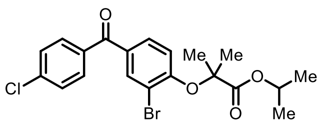

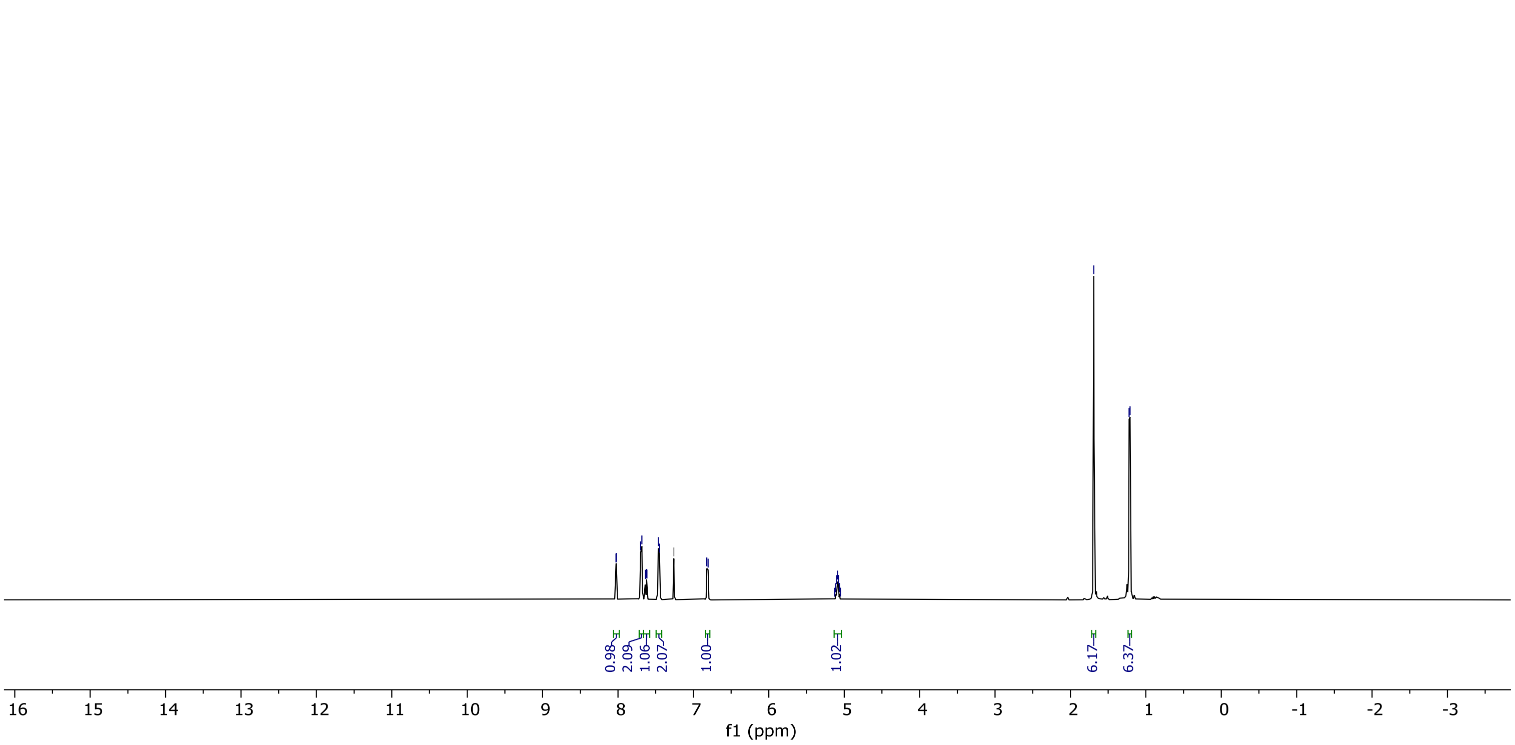

Compound 49 ^{13}C NMR in $CDCl_3$, 298 K

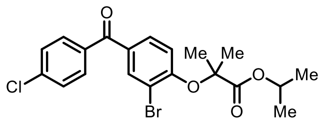

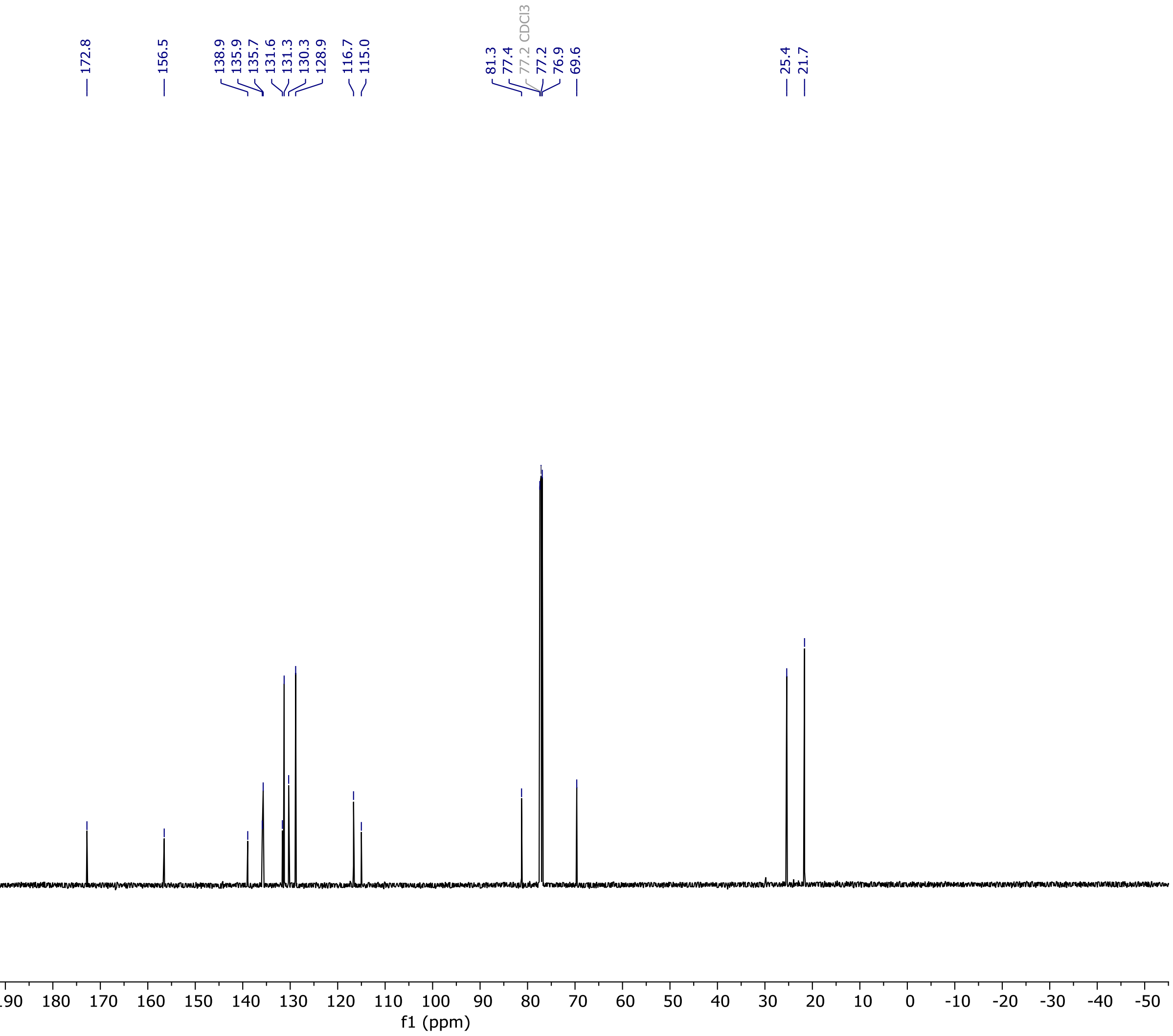

Compound 50 ¹H NMR in $CDCl_3$, 298 K

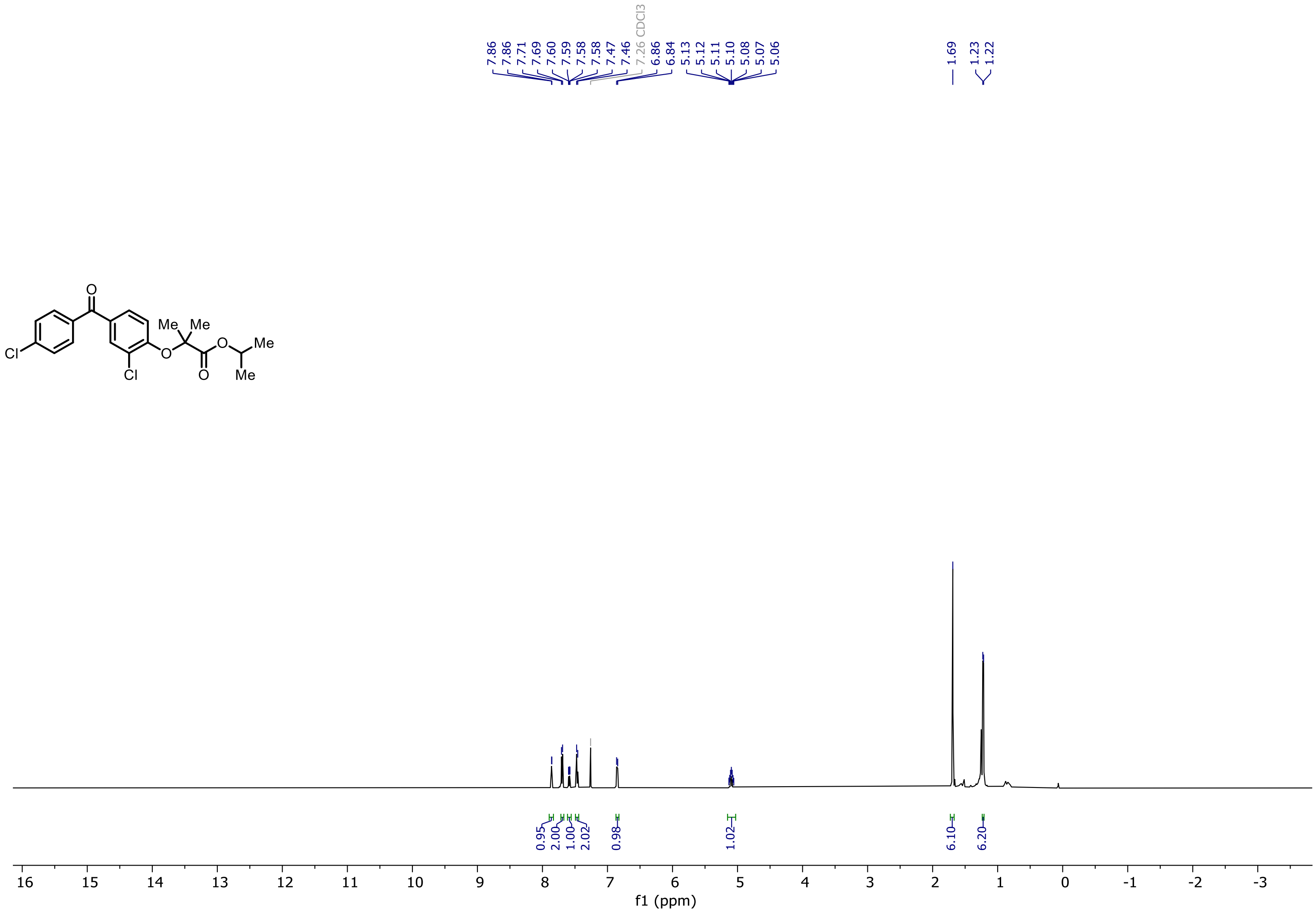

Compound 50 ^{13}C NMR in $CDCl_3$, 298 K

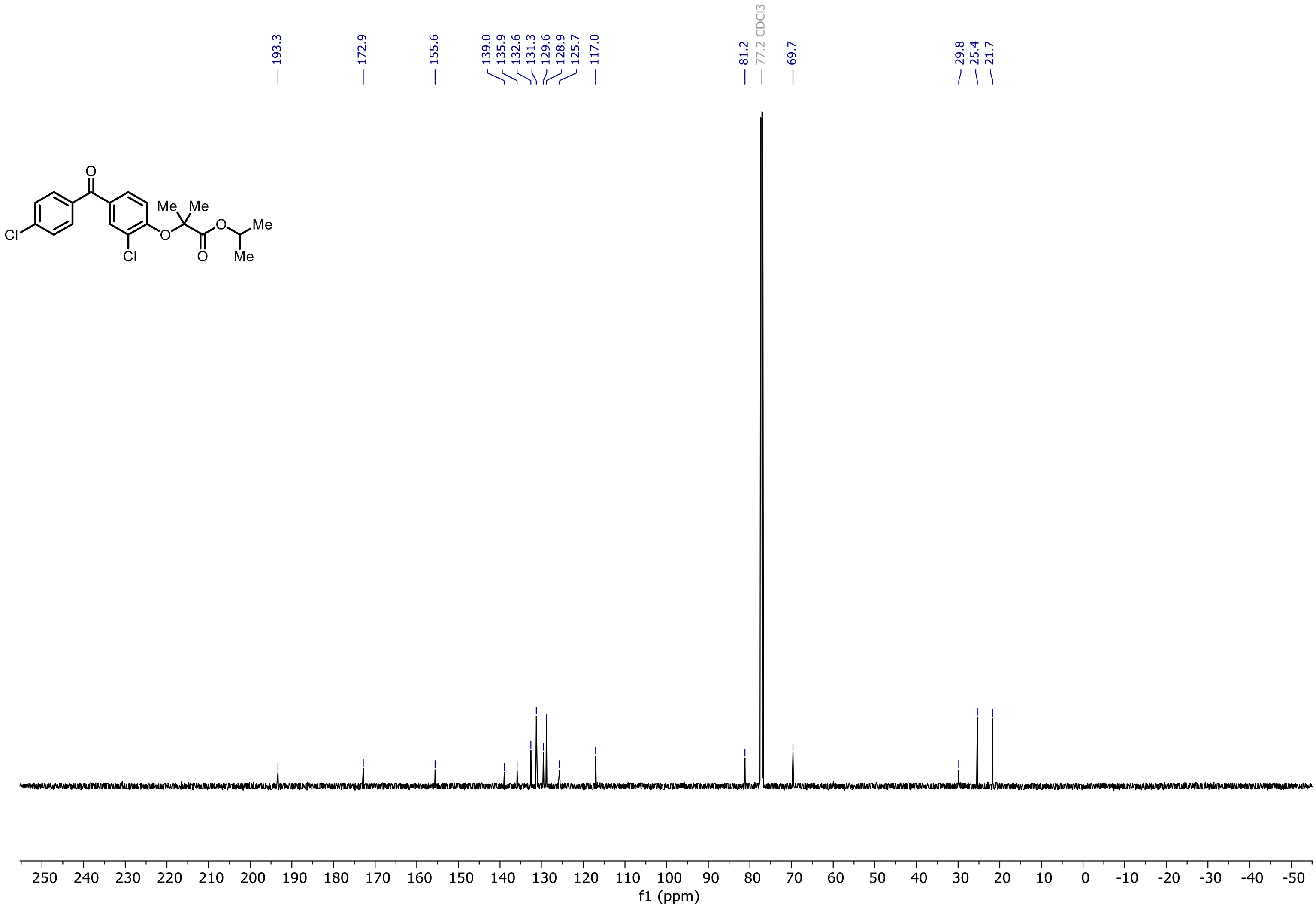

Compound 51 ^{1}H NMR in $CDCl_3$, 298 K

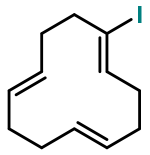

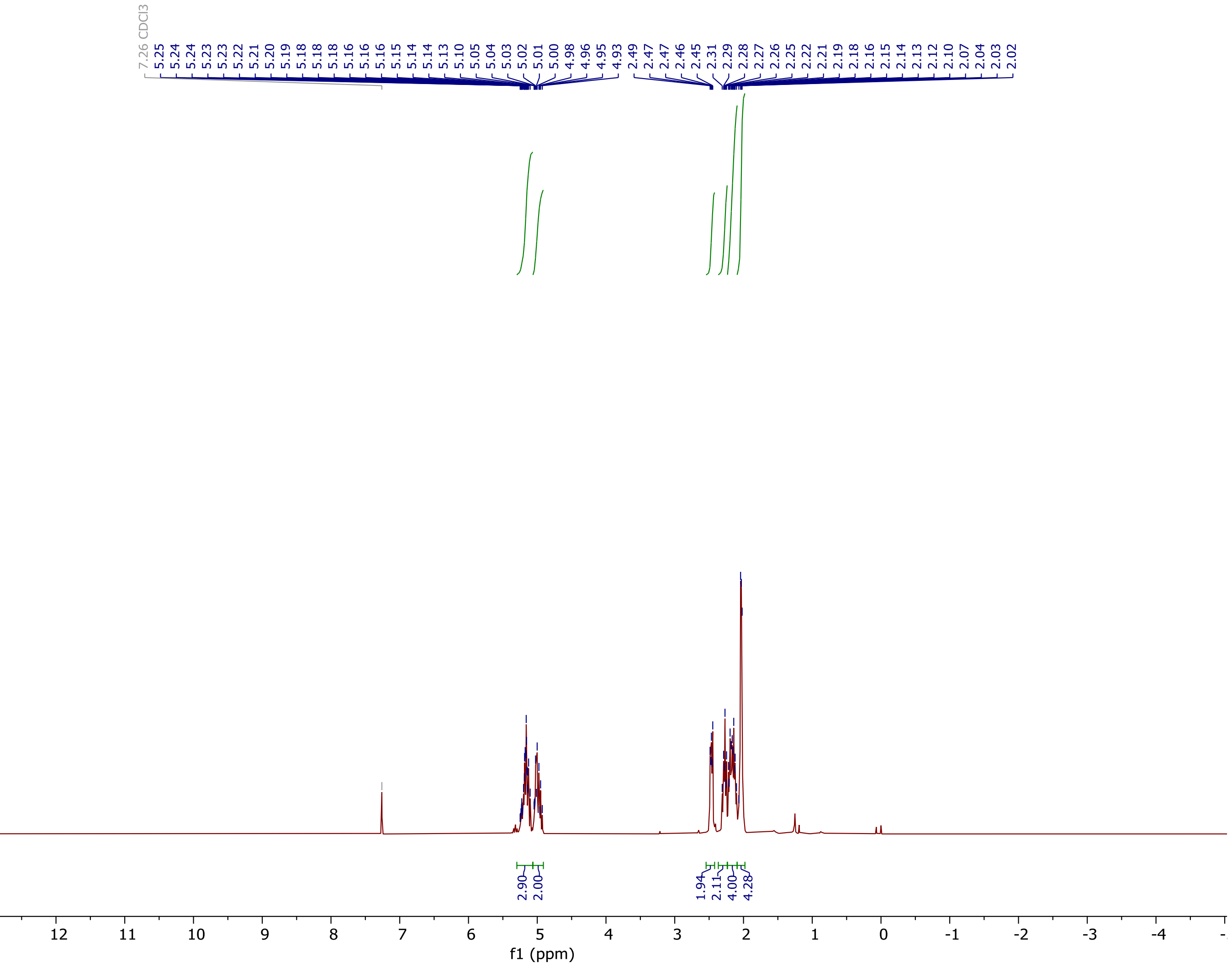

Compound 51 ^{13}C NMR in $CDCl_3$, 298 K

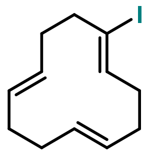
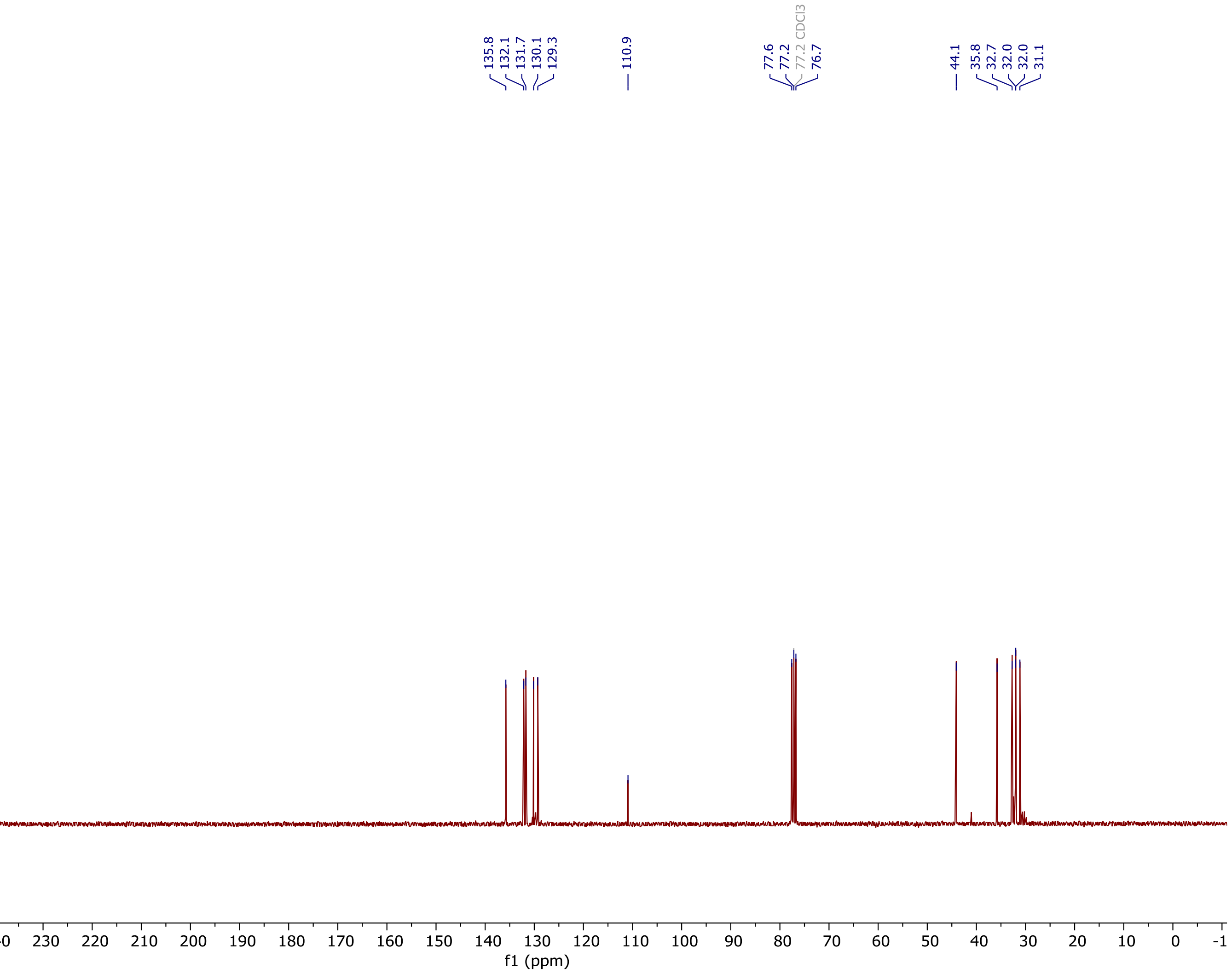

Compound 52 ^{1}H NMR in $CDCl_3$, 298 K

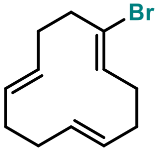

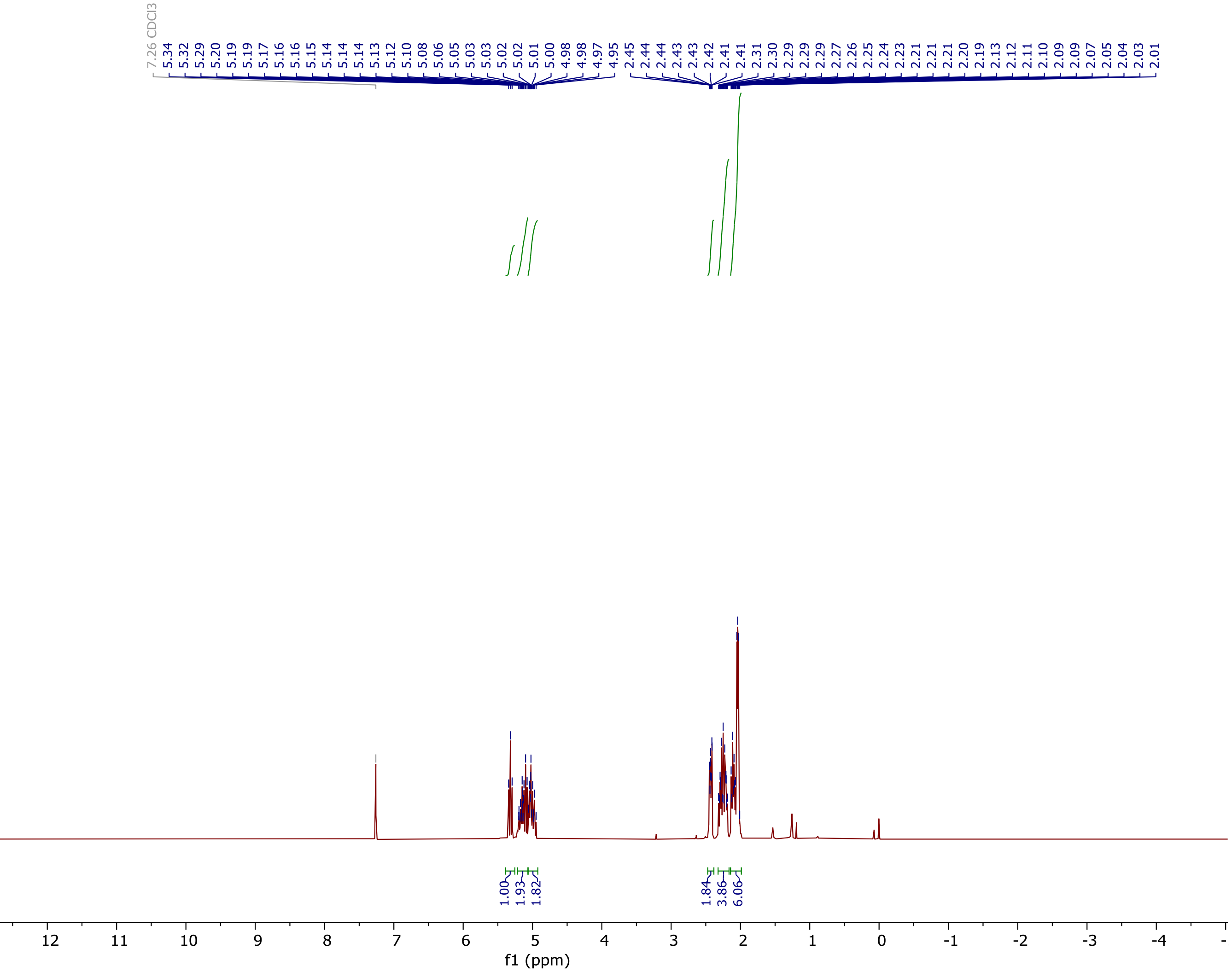

Compound 52 ^{13}C NMR in $CDCl_3$, 298 K

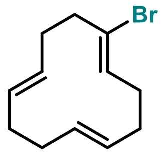

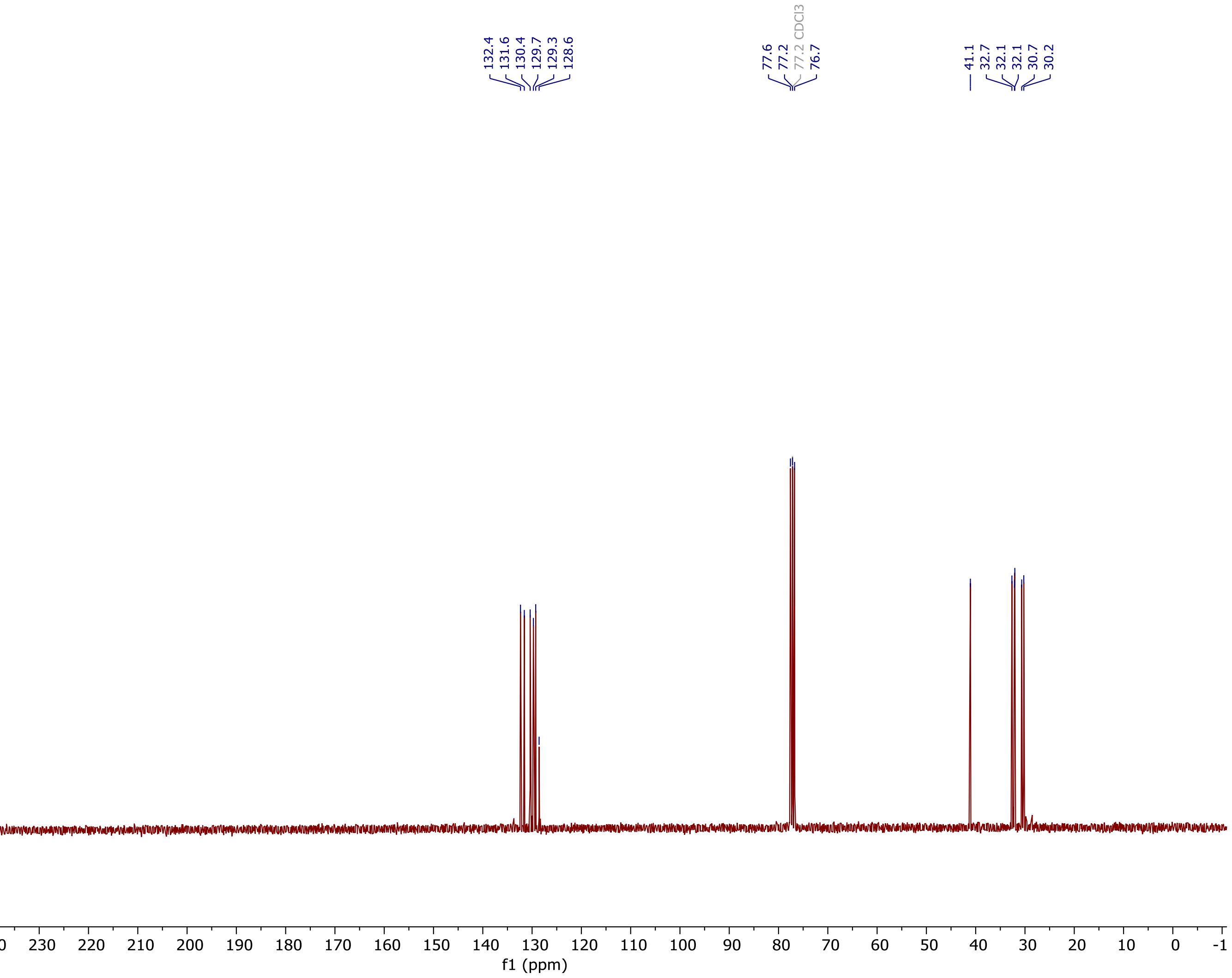

Compound 54 ^{1}H NMR in $CDCl_3$, 298 K

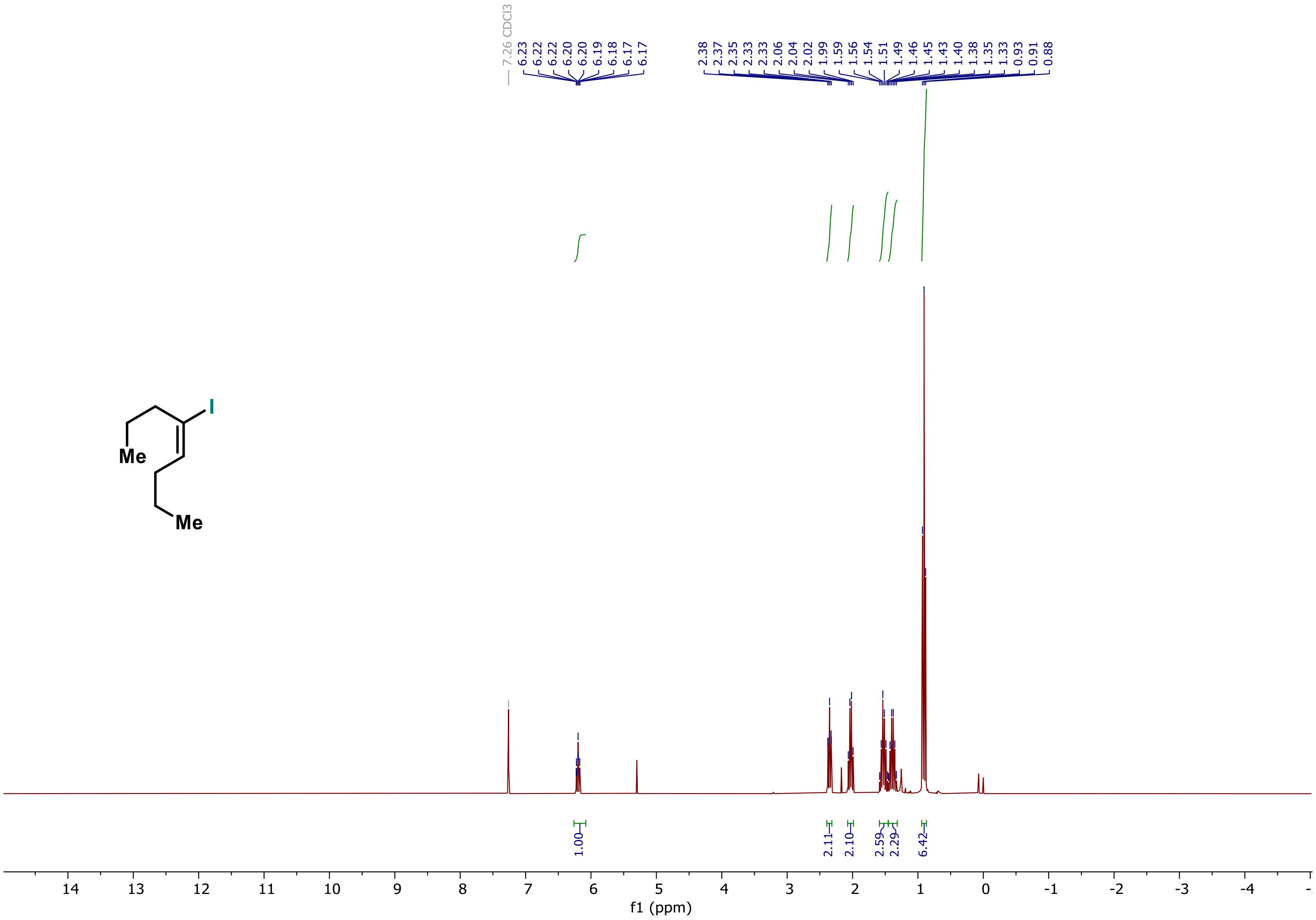

Compound 54 ^{13}C NMR in $CDCl_3$, 298 K

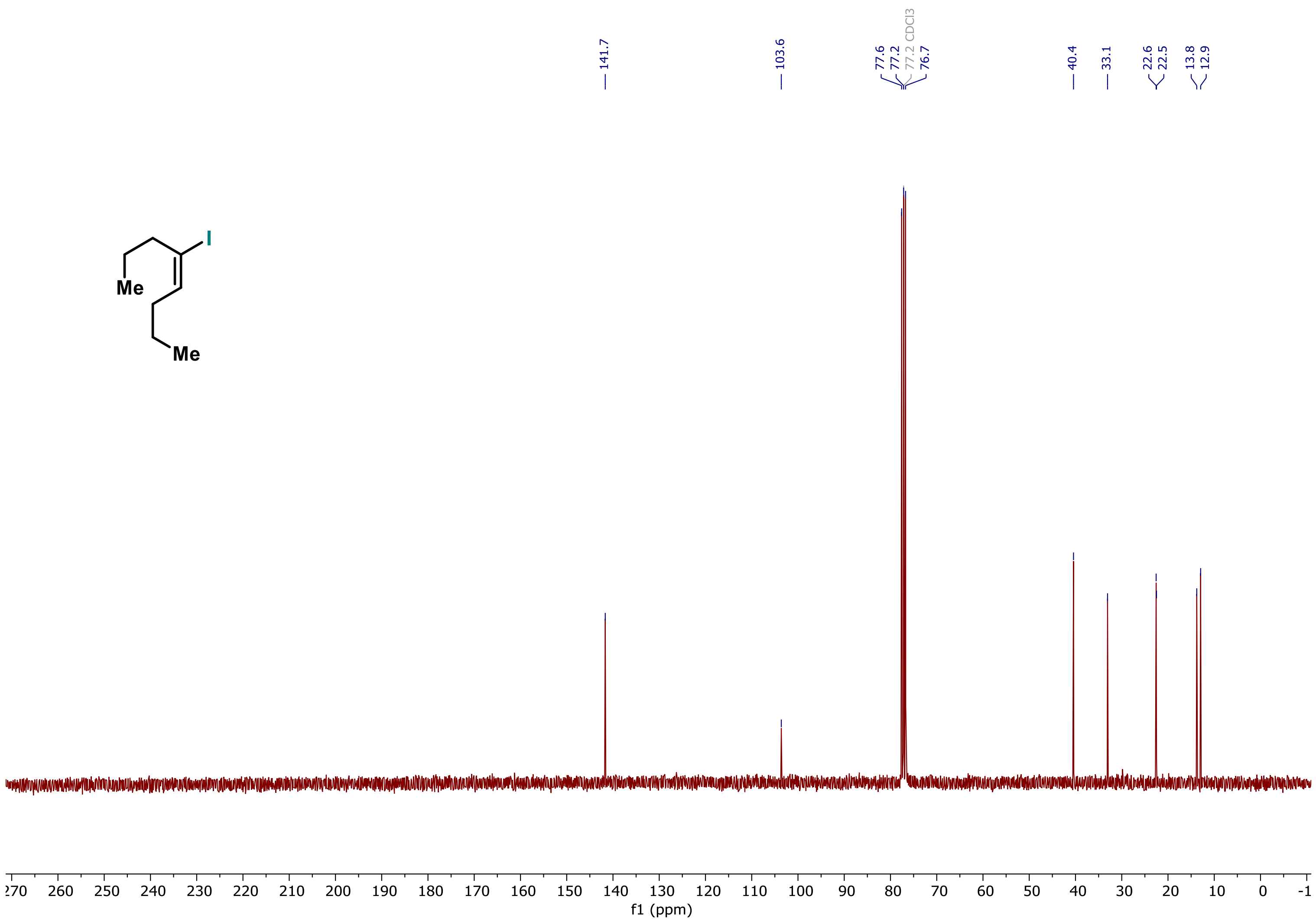

Compound 55 ^{1}H NMR in $CDCl_3$, 298 K

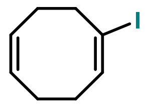

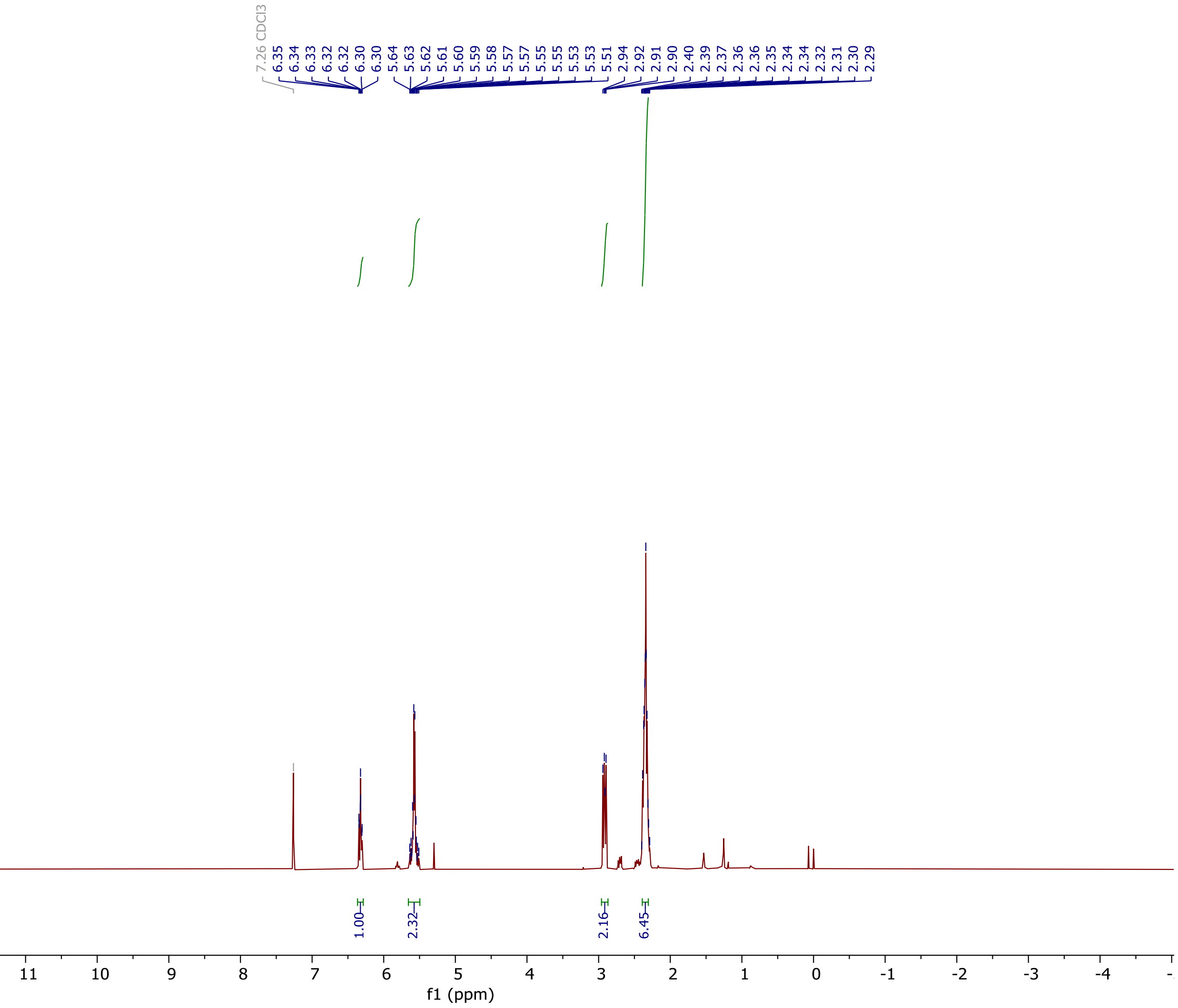

Compound 55 ^{13}C NMR in $CDCl_3$, 298 K

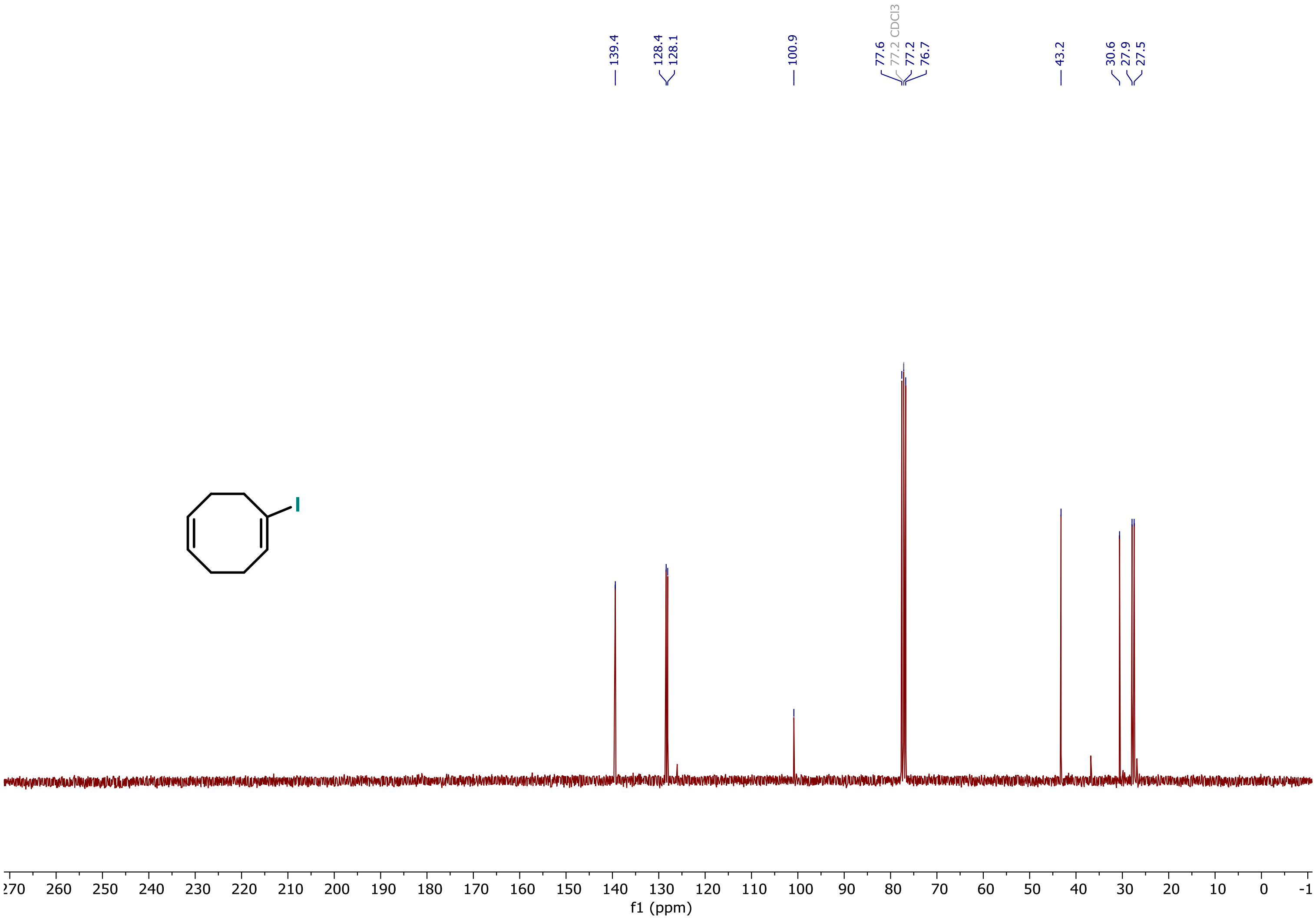

Compound 61 ^{1}H NMR in $CDCl_3$, 253 K

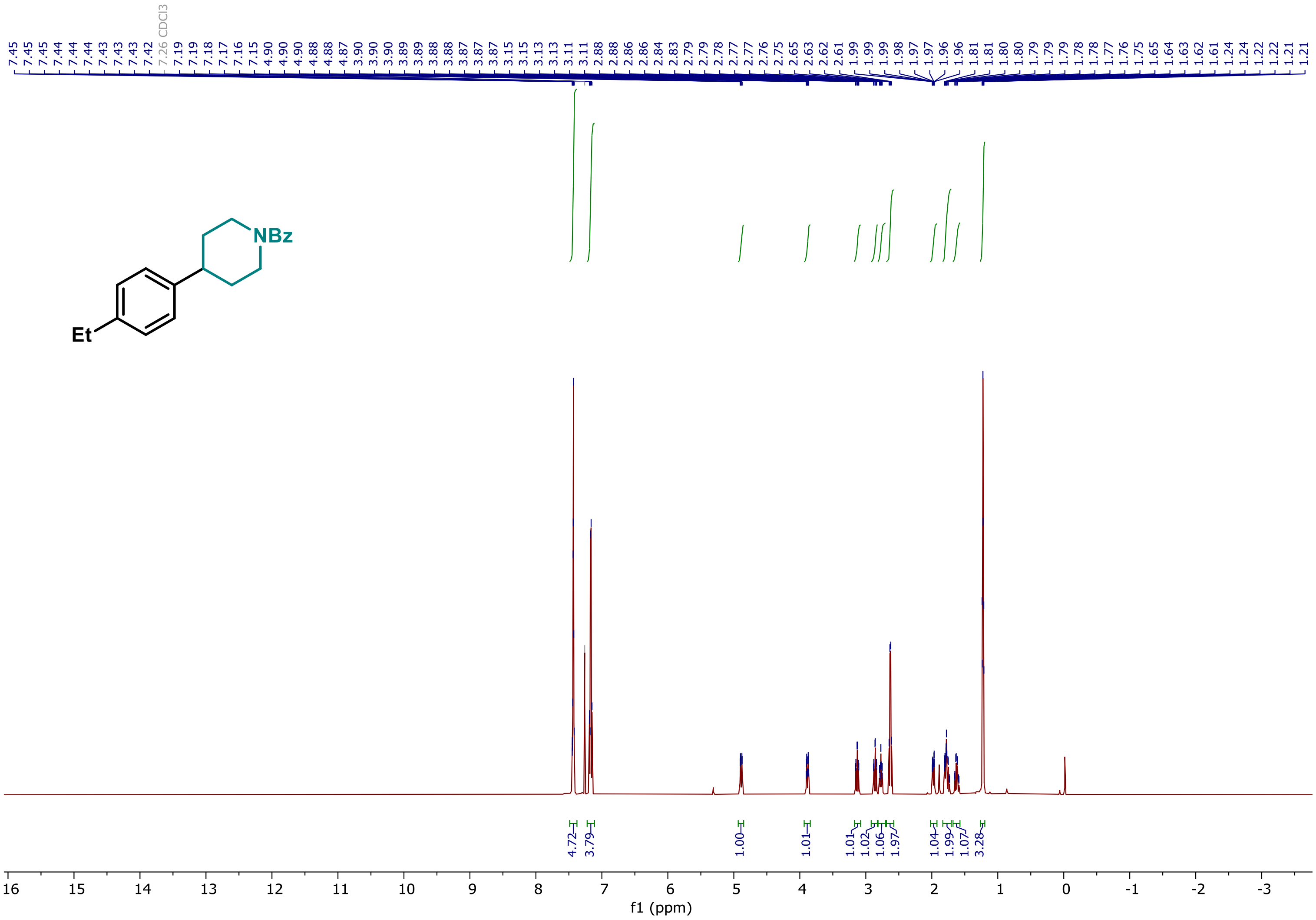

Compound 61 ^{13}C NMR in $CDCl_3$, 253 K

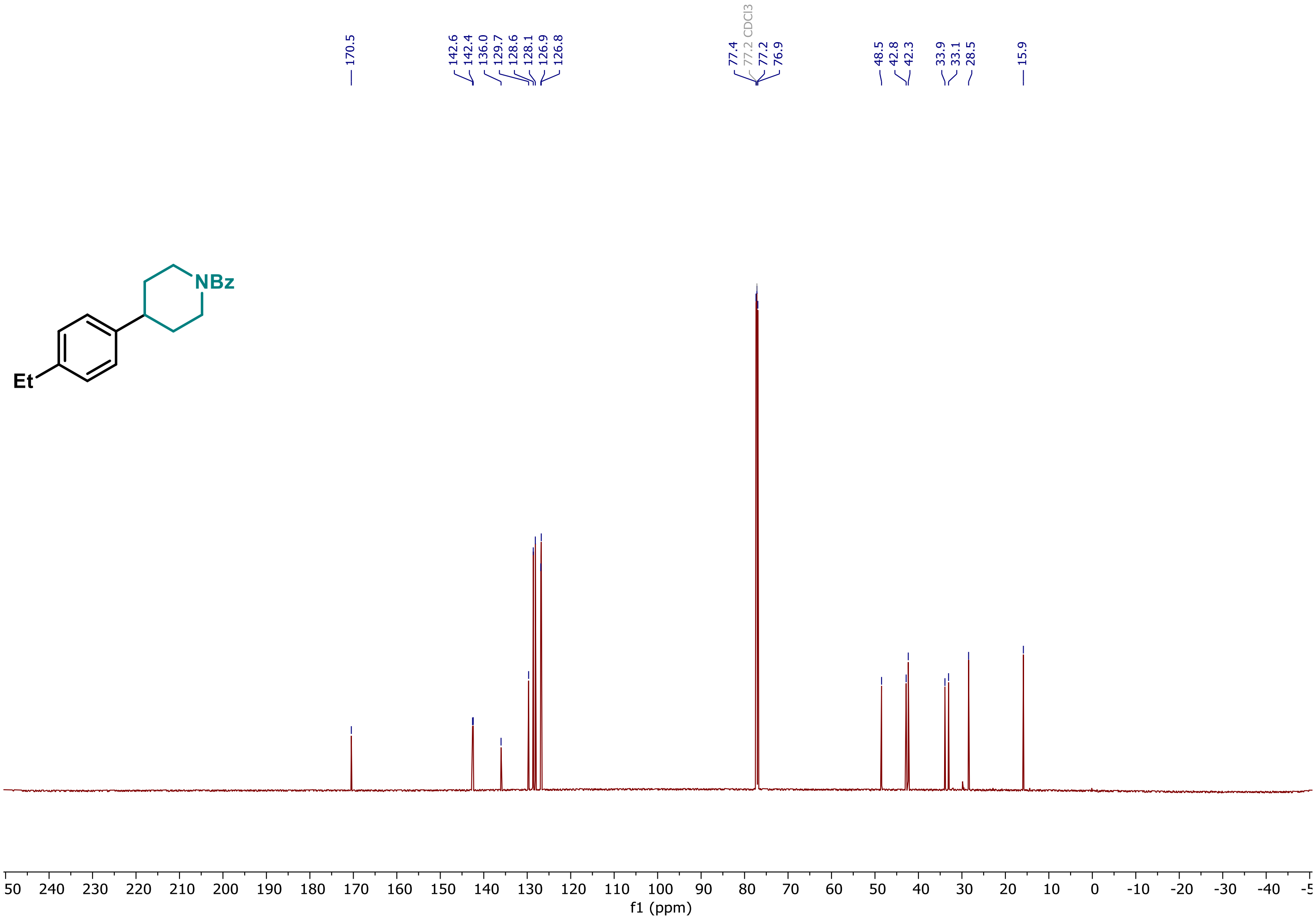

Supplement: Supplementary file 1 — ja3c02611_si_001.pdf [file ja3c02611_si_001.pdf]
